# Genome mining and biosynthesis of kitacinnamycins as a STING activator†
†Electronic supplementary information (ESI) available. See DOI: 10.1039/c9sc00815b


**DOI:** 10.1039/c9sc00815b

**Published:** 2019-04-02

**Authors:** Jing Shi, Cheng Li Liu, Bo Zhang, Wen Jie Guo, Jiapeng Zhu, Chin-Yuan Chang, Er Juan Zhao, Rui Hua Jiao, Ren Xiang Tan, Hui Ming Ge

**Affiliations:** a State Key Laboratory of Pharmaceutical Biotechnology , Institute of Functional Biomolecules , School of Life Sciences , Nanjing University , 210023 , P. R. China . Email: rxtan@nju.edu.cn ; Email: hmge@nju.edu.cn; b State Key Laboratory Cultivation Base for TCM Quality and Efficacy , Nanjing University of Chinese Medicine , Nanjing 210023 , P. R. China; c Department of Biological Science and Technology , National Chiao Tung University , Hsinchu , Republic of China

## Abstract

Cinnamoyl-containing nonribosomal peptides (CCNPs) are a small group of secondary metabolites with potent biological activities produced by actinobacteria. Two remarkable features in the biosynthesis of CCNPs include the nonribosomal peptide synthases (NRPSs) for assembly of the depsipeptide backbone and the type II polyketide synthases (PKSs) for N-terminal cinnamoyl moiety construction. Here, we present a genome mining approach targeting both NRPS and type II PKS for discovery of new CCNPs, which led to the identification of 51 putative CCNP gene clusters from public bacterial genome databases. After strain prioritization, a novel class of CCNP-type glycopeptides named kitacinnamycins, one of which showing potent activation ability towards the stimulator of interferon genes (STING) protein, was identified. Bioinformatic, genetic and biochemical analysis revealed the use of the NRPS assembly line to form the macrocyclic peptide backbone, followed by a P450 monooxygenase to generate terminal oxidized groups. A glycosyltransferase with relatively broad substrate specificity transfers sugars to the newly generated OH/COOH group. The protein crystallographic study further provided structural insights into this glycosylation. Our results not only demonstrated the feasibility of genome mining and strain prioritization for the discovery of new bioactive natural products but also disclosed the biosynthetic pathway for kitacinnamycins.

## Abstract 2

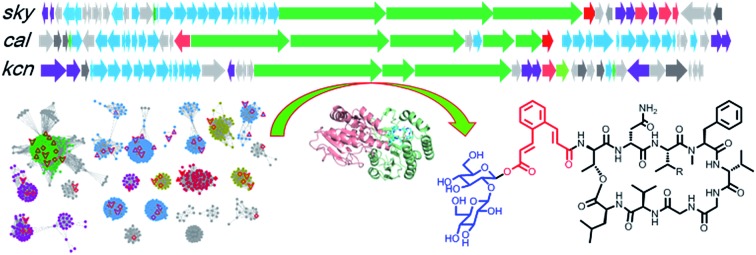
Genome mining targeting unique type II PKS and NRPS led to the identification of a novel class of glycopeptides named kitacinnamycins.

## Introduction

Nonribosomal peptides (NRPs) are one of the most structurally diverse classes of natural products that exhibit a wide range of biological activities and include important therapeutic drugs such as vancomycin and cyclosporine A.^1^,^2^ Many NRPs possess N-terminal acylation, which is important for protecting the N-terminus from degradation or modulating specific properties. N-terminal acylation is commonly achieved through coupling with an activated fatty acid^3^–^5^ or acyl residues derived from amino acid precursors.^6^,^7^ Different to this N-terminal modification, skyllamycin, a potent inhibitor of the platelet-derived growth factor (PDGF) signaling pathway, contains a unique 2-[1-(*Z*)-propenyl]-cinnamoyl moiety.^8^ The N-terminal cinnamoyl moiety is rarely observed in NRPs and so far has only been reported in WS9326A,^9^ mohangamides,^10^ and coprisamides (Fig. S1†),^11^ all of which possess interesting structural features. Importantly, WS3926A is a potent agonist of the tachykinin receptor, mohangamides show strong inhibitory activity against isocitrate lyase from *Candida albicans*, and coprisamides display significant activity towards induction of quinone reductase.^9^–^11^ Thus, CCNPs represent a small but very unique class of NRPs, not only due to their interesting structural architectures but also due to their potent and diverse biological activities.

Biosynthetic gene clusters (BGCs) for skyllamycin (*sky*) and WS9326A (*cal*) have already been identified, revealing that the backbones of both compounds were assembled through multimodule NRPSs.^8^,^12^ In addition, unusual type II polyketide synthases (PKSs) and accessory enzymes, which are conserved among the *sky* and *cal* clusters,^8^,^12^ are predicted to synthesize the cinnamoyl moiety. Briefly, the KS_α_–KS_β_, KR, and DH in type II PKSs catalyze the repeating cycles of decarboxylative Claisen condensation, β-keto reduction and dehydration, respectively, to afford a polyene precursor. An isomerase that may reverse the 6*E*-polyene to 6*Z*-configuration is hypothesized to bring the carbon atoms close enough for the subsequent 6π-electrocyclization, followed by a desaturation step to give the required cinnamoyl residue (Fig. S2†).^8^ In the present work, we utilized a genome-mining strategy targeting both NRPSs and discrete enzymes of type II PKSs involved in cinnamoyl biosynthesis to mine the potential BGCs of CCNPs from bacterial genomes, leading to the identification of 51 putative CCNP BGCs. After strain prioritization and dereplication, we discovered 14 novel CCNPs, kitacinnamycins A–N (**1–14**), with **8** showing potent STING activation activity. The biosynthetic pathway for kitacinnamycins was revealed through a series of gene deletions, biochemical reactions, and protein crystallographic analyses.

## Results and discussion

### Genome mining reveals 51 CCNP biosynthetic gene clusters from the genomic database

**Scheme 1:**
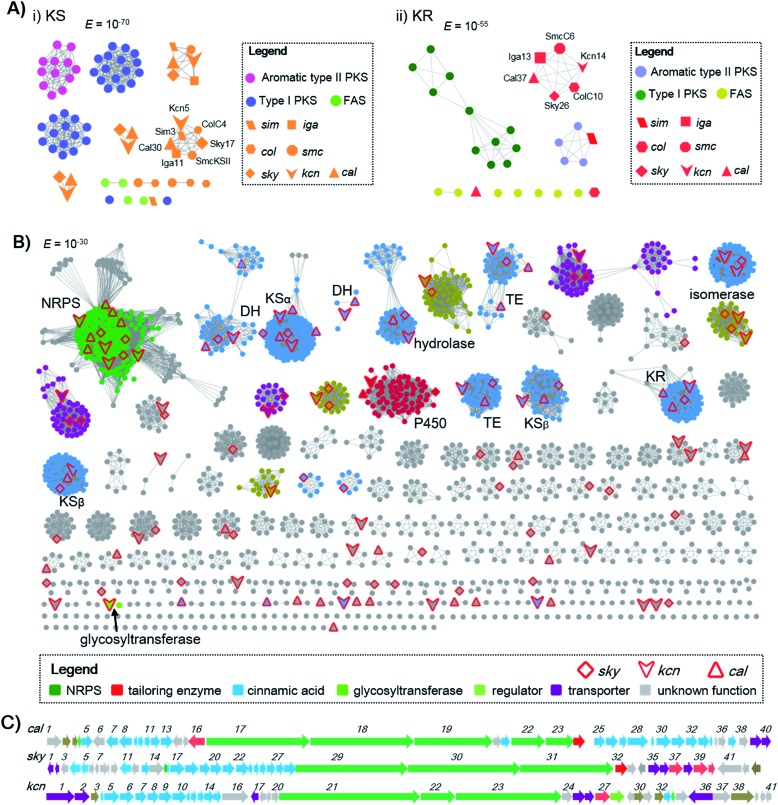
Genome mining of CCNPs. (A) The SSN analysis of representative KSs and KRs. (i) KSs are displayed at an *E* value threshold of 10^–70^. KS proteins (Sim3, Cal30, Iga11, SmcKSII, Sky17 and ColC4) from polyene type II PKSs are clustered together; (ii) KRs are displayed at an *E* value threshold of 10^–55^. KR proteins (SimJ2, Cal37, Iga13, SmcC6, Sky26 and ColC10) from polyene type II PKSs are clustered together. (B) The GNN for CCNPs consisting of two known (*sky* and *cal* clusters) and 51 newly identified CCNP clusters. The GNN is displayed at an *E* value threshold of 10^–30^. (C) BGCs for WS9326 (*cal*), skyllamycin (*sky*) and kitacinnamycin (*kcn*). The GNN and BGCs are color-coded based on the proposed function.

Genome mining to discover structurally specific natural products relies on the use of conserved probes that are in the targeted BGCs but not present in others. The cinnamoyl residue in CCNPs is proposed to be synthesized through a polyene precursor, which is likely formed by a type II PKS.^8^,^13^,^14^ To gain insight into whether the type II PKSs in polyene biosynthesis can be separated from other PKS systems, we first collected discrete enzymes of polyene type II PKSs [skyllamycin (*sky*),^8^ WS9326 (*cal*),^12^ ishigamide (*iga*),^15^ colabomycins (*col*)^13^ and simocyclinone (*sim* and *smc*)] (Fig. S1 and S3†),^14^,^16^ type I PKSs, aromatic type II PKSs, and type II FASs. Then, we generated a protein sequence similarity network (SSN) to visualize and analyze the diversity of type II PKSs using the Enzyme Function Initiative-Enzyme Similarity Tool (EFI-EST).^17^ A preliminary *E* value threshold of 1.0 × 10^–10^ was set to gather the related protein families together. When the *E* value was smaller (*i.e.*, higher threshold value), it would allow the separation of the related enzymes and result in smaller subfamilies. At an *E* value of 10^–70^, the KSs from different PKS systems are indeed separated, but KSs from polyene type II PKSs are also distributed in several distinct clusters. However, one KS_α_ (some polyene BGCs contain more than one pair of KS_α_–KS_β_) from each polyene type II PKS BGC can be clustered together (Scheme 1A), indicating that this class of KS_α_ is conserved and unique among all KSs and could be selected as a probe for genome mining. Also, KRs from polyene type II PKSs can also be separated from others at an *E* value threshold of 10^–55^, whereas DHs do not cluster at low *E* values (*e.g.*, 10^–15^). In addition, at least one isomerase, which is expected to reverse the geometry of alkene in polyene, is present in both the *cal* and *sky* BGCs but not observed in other type II PKSs. Thus, homology searches in the bacterial genome NCBI and JGI databases utilizing the skyllamycin proteins KS (Sky17, *E* value ≤10^–70^), KR (Sky26, *E* value ≤10^–55^) and isomerase (Sky27) as queries afforded a total of 192 genomes in which all three genes were present. To further analyze if these genes are physically adjacent to NRPS genes, we subjected these genomes data to antiSMASH analysis.^18^ This led to the identification of 51 putative BGCs that have an intact type II PKS system (including KS, KR, DH, ACP, and isomerase) and NRPS genes clustered together (Scheme 1 and Fig. S4†). To further annotate these potential CCNP BGCs and prioritize the hits, a genome neighbourhood network (GNN) consisting of 3196 proteins from 51 putative CCNP BGCs, together with *cal* and *sky*, was generated.^19^ The GNN revealed that two BGCs from *Streptomyces* sp. Root63 and *Streptomyces griseus* BIG105 showed high similarities to *sky* and *cal* (identity > 90%), respectively, and may also encode skyllamycin or WS3926A (Fig. S4†). It is therefore significant that the remaining 49 BGCs are diverse and possess many functionally different enzymes, indicating that these hits represent potential novel CCNP producers. Importantly, two glycosyltransferases were observed only in *Kitasatospora* sp. CGMCC 16924 and *Streptomyces* sp. LZ35 (Scheme 1B), suggesting that glycosylation tailoring steps likely occur in the biosynthesis of their corresponding final products.

### Characterization of kitacinnamycins from *Kitasatospora* sp. CGMCC 16924 as new CCNP-type glycopeptides

**Fig. 1:**
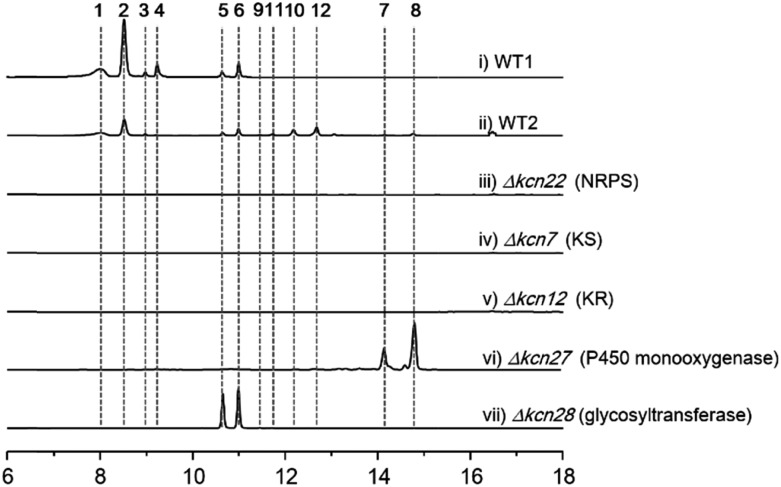
HPLC profiles (300 nm) of metabolites extracted from *K.* sp. CGMCC 16924 and mutant strains. WT1, WT strain fermented for 10 days; WT2, WT strain fermented for 4 days; all mutant strains were fermented for 10 days.

We were interested in identifying the putative CCNP-type glycopeptides in CGMCC 16924 (*S.* sp. LZ35 was not available). To facilitate this process, we constructed a NRPS gene deletion strain (Δ*kcn22*). The wild-type (WT) strain was then fermented under several fermentation conditions using Δ*kcn22* as a control. Metabolic extracts were then analyzed by HPLC. Six peaks with UV absorbance at 260 and 300 nm from the WT strain were observed when fermented in SCAS medium. In contrast, the production in the Δ*kcn22* strain was completely abolished, indicating that these peaks are correlated with the *kcn* BGC (Fig. 1). A large scale (20 L) fermentation of the WT strain was then conducted, which resulted in the isolation of six compounds named kitacinnamycins A–F (**1–6**). The planar structures formula of **1–6** were determined based on the extensive analyses of spectroscopic data including high-resolution mass (HRMS) and 1D (^1^H and ^13^C) and 2D (HSQC, HMBC, ^1^H–^1^H COSY, and ^1^H–^15^N HMBC) NMR data (Tables S5–S10†). The gross structures were also supported by MS/MS fragmentation analysis (Fig. S5†). The absolute configurations of amino acid residues in **1** and **2** were assigned on the basis of the advanced Marfey's method (Fig. S6†), and the sugar moieties were determined as d-glucose through a NOESY experiment and GC/MS analysis of the trimethylsilyl derivatives of the hydrolyzed compound **1** (Fig. S7†). Structures of **1–4** consist of a 9-mer macrocyclic peptide with a cinnamoyl unit, in which a β-d-glucose-(1 → 2)-β-d-glucose disaccharide or *N*-acetylglucosamine moiety was attached. To our delight, structures of **1–4** indeed represent a new class of CCNP-type glycopeptides.

**Scheme 2:**
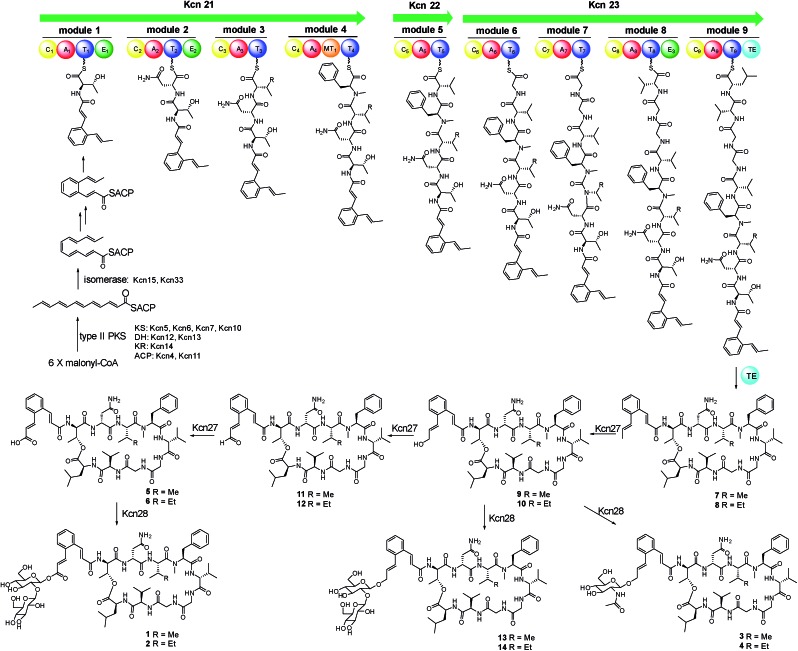
Proposed biosynthetic pathway for kitacinnamycins A–N (**1–14**).

As these structures are unusual, we set out to understand the molecular basis for the assembly of **1–6**. The *kcn* BGC spans a *ca.* 55 kb DNA segment consisting of 41 open reading frames (Scheme 1C), whose putative functions were assigned according to BLAST analysis (Table S3†). Consistent with the 9-mer macrocyclic peptide backbone, three NRPS enzymes (Kcn21, Kcn22, and Kcn23) encoded in the *kcn* BGC comprise nine modules that were expected to incorporate nine amino acid building blocks to form a linear nonapeptide (Table S4†), with the terminal thioesterase (TE) domain in Kcn23 catalyzing macrocyclization. The cluster also encodes a set of enzymes (*kcn4–kcn15* and *kcn33*) showing moderate homology to the type II PKS in the *sky* BGC, which is expected to biosynthesize the N-terminal cinnamic group. Knocking out either KS (*kcn7*) or KR (*kcn14*) gene completely eliminated the production of all metabolites (Fig. 1),^8^ confirming their involvement in kitacinnamycin biosynthesis (Scheme 2).

### Kcn27 is a P450 monooxygenase catalyzing three successive oxidation steps

In contrast to the terminal methyl group of the cinnamoyl moiety in skyllamycin or other CCNPs,^8^–^11^ **1–6** harbor a terminal carboxylic acid or hydroxyl group. We postulated that the original terminal methyl group is first hydroxylated, with subsequent transfer of an *N*-acetylglucosamine moiety. Alternatively, the hydroxyl group can be further oxidized to the carboxyl acid through an aldehyde intermediate. Cytochrome P450 enzymes that carry out successive oxidation steps on methyl carbons have been reported in several biosynthetic pathways of natural products.^20^–^22^ Indeed, only one P450 monooxygenase (Kcn27), showing 45% sequence identity to PldB, a P450 hydroxylase in pladienolide biosynthesis,^23^ was observed in the *kcn* BGC. Knocking out *kcn27* abolished the production of **1–6** but clearly led to the accumulation of a pair of new products **7** and **8** with [M + Na]^+^ 1093.5688 and 1107.5845, respectively. The structures of **7** and **8** were determined as the expected macrocyclic peptides with an N-terminal 2-[1-(*E*)-propenyl]-cinnamoyl moiety, suggesting the role of Kcn27 in catalyzing successive oxidation steps at the terminal methyl group.

**Fig. 2:**
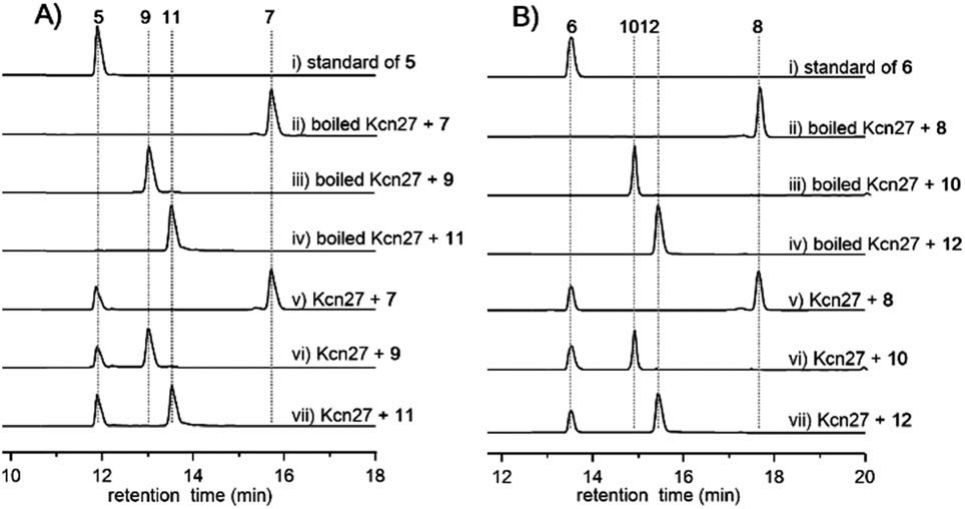
HPLC analysis (300 nm) of the Kcn27 catalyzed enzymatic reaction. Each enzymatic reaction was supplemented with Fdr, Fdx and NADPH.

To further verify the function of Kcn27, the recombinant P450 enzyme was overproduced and purified to homogeneity from *Escherichia coli* BL21(DE3) (Fig. S9†). Its cognate ferredoxin (Fdx) and ferredoxin reductase (Fdr) were also purified. Individual incubation of **7** and **8** with Kcn27 together with the required Fdx and Fdr and NADPH in 50 mM MES buffer (pH 5.8) led to the formation of a new product identical to **5** and **6** in retention time and mass, respectively (Fig. 2). In addition, the putative hydroxyl (**9**/**10**) and aldehyde (**11**/**12**) intermediates with the expected molecular weight were detected in small but reproducible amounts by LC-MS (Fig. S10†). To further verify this, we performed a fermentation time-course using the WT strain to see if these intermediates can be observed in the early time point. Gratifyingly, after 4 d of cultivation four new peaks with expected molecular ions of **9–12** can be detected (Fig. 1,ii). Compounds **9–12** were thus isolated and characterized from a large-scale fermentation. Individual incubation of **9** or **11** and **10** or **12** with Kcn27 under the same enzymatic reaction conditions led to the production of **5** and **6**, respectively (Fig. 2). These *in vivo* and *in vitro* results collectively indicate that Kcn27 is a P450 oxidase responsible for three successive two-electron oxidation steps at the terminal methyl group of the cinnamoyl moiety.

### *In vivo* and *in vitro* characterization of Kcn28 as a glycosyltransferase

The glycosyltransferase Kcn28, which is unique among all CCNP BGCs, was expected to transfer a glycosyl group onto either the terminal COOH (**5** and **6**) or OH group (**9** and **10**). To examine its role, *kcn28* was knocked out through in-frame deletion. The Δ*kcn28* mutant completely eliminated the production of **1–4**, only leaving **5** and **6** as two dominant products (Fig. 1). This result suggested that Kcn28 could be responsible for introducing the glycosyl group at the cinnamoyl moiety.

**Fig. 3:**
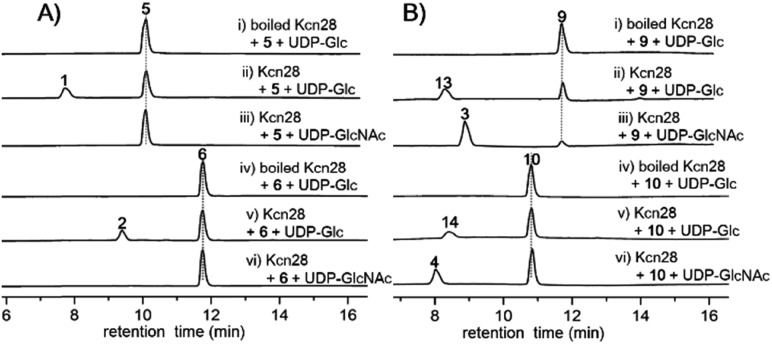
HPLC analysis (300 nm) of the Kcn28 catalyzed enzymatic reaction.

To confirm the function of Kcn28, the recombinant enzyme was overproduced from *E. coli* BL21(DE3) (Fig. S9†). The sugar donor UDP-d-glucose was then incubated with **5** or **6** in the presence of Kcn28, which leads to the formation of **1** or **2**, respectively (Fig. 3). When the hydroxyl compound **9** or **10** was used as the sugar acceptor and incubated with UDP-d-GlcNAc and Kcn28, as expected the corresponding product **3** or **4** was detected (Fig. 3). To further generate structural diversity, **9** or **10** was assayed with UDP-d-glucose. Interestingly, the putative disaccharide product **13** (*m*/*z* 1433.7 [M + Na]^+^) or **14** (*m*/*z* 1447.6 [M + Na]^+^) was observed based on its molecular ions (Fig. 3 and S11†). In contrast, no reaction occurred when **5** or **6** was assayed with UDP-d-GlcNAc in the presence of Kcn28 (Fig. 3).

### Crystal structure of Kcn28 reveals the structural basis of glycosylation

**Fig. 4:**
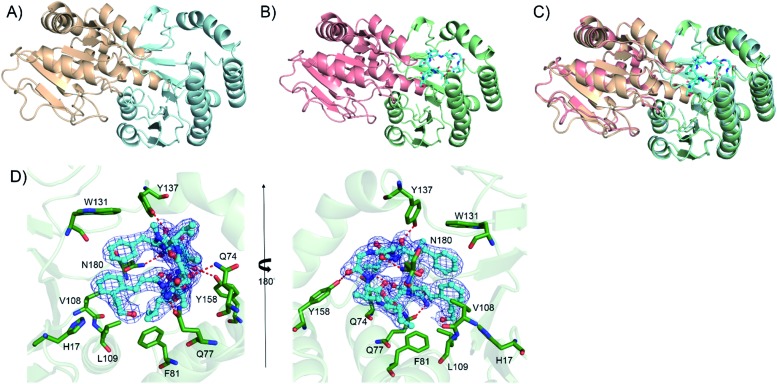
The crystal structure of the glycosyltransferase Kcn28. (A) The structure of free Kcn28. The substrate binding (N-terminal) domain is shown in pale cyan, while the UDP-sugar binding (C-terminal) domain is shown in wheat. (B) The structure of Kcn28 in complex with **9**. The substrate binding (N-terminal) domain is shown in pale green, while the UDP-sugar binding (C-terminal) domain is shown in salmon; substrate **9** is shown in cyan. (C) The superimposed image of the two forms; (D) local view of the catalytic pocket highlighting possible key residues (green) involved in the interaction between Kcn28 with **9** (cyan). Hydrogen bonds are shown as red dashed lines.

We further attempted to understand the structural basis of the Kcn28-catalyzed enzymatic reaction. Crystal structures of Kcn28 as a free enzyme and in a binary complex (with **9**) were obtained at 2.50 and 2.24 Å resolution, respectively (Fig. 4A and B and Table S17†). The monomeric Kcn28 contains two Rossmann-like β/α/β domains. The N-terminal domain from residues 6–208 is a substrate binding domain consisting of seven parallel β-strands that are flanked by seven α-helices, while the C-terminal domain comprising residues 229–392 is a UDP-sugar binding domain with six parallel β-strands surrounded by seven α-helices. The overall structure of Kcn28 is similar to that of other glycosyltransferases in natural product biosynthesis, including OleD (PDB: ; 2IYF) (Fig. S12A†)^24^ and CalG3 (PDB: ; 3OTI) (Fig. S12B†)^25^ from oleandomycin and calicheamicin biosynthesis, respectively.

The model of **9** was confidently built into well-defined electron densities. Superposition of free and complexed structures revealed a global conformational change (Fig. 4C). The substrate **9** is tightly encapsulated in four α-helices with an orientation directed by insertion of the macrocyclic peptide part into the cavity and exposure of the cinnamic acid portion to the UDP part. Specifically, the side chains of F81, V108, and L109 create a hydrophobic environment to accommodate the cinnamic moiety of **9**. In addition, the side chain of W131 provides hydrophobic interaction with the Phe residue of the macrocyclic peptide (Fig. 4D). The side chains of Q74, Q77, Y137, Y158 and N180 provide hydrogen bond interactions with the macrocyclic peptide backbone. Site-directed mutagenesis of the above key residues revealed diminished or abolished enzyme activities (Fig. S13†), supporting their roles in substrate binding. Additionally, H17, which is conserved among structurally similar glycosyltransferase (Fig. S14†) and adjacent to the hydroxyl group of the cinnamic moiety of **9**, is proposed as the catalytic base by abstracting a proton in the terminal OH/COOH group.^24^ Consistent with the hypothesis, the H17A mutant renders the enzyme inactive.

### Kitacinnamycin H is an activator of the STING signalling pathway

**Fig. 5:**
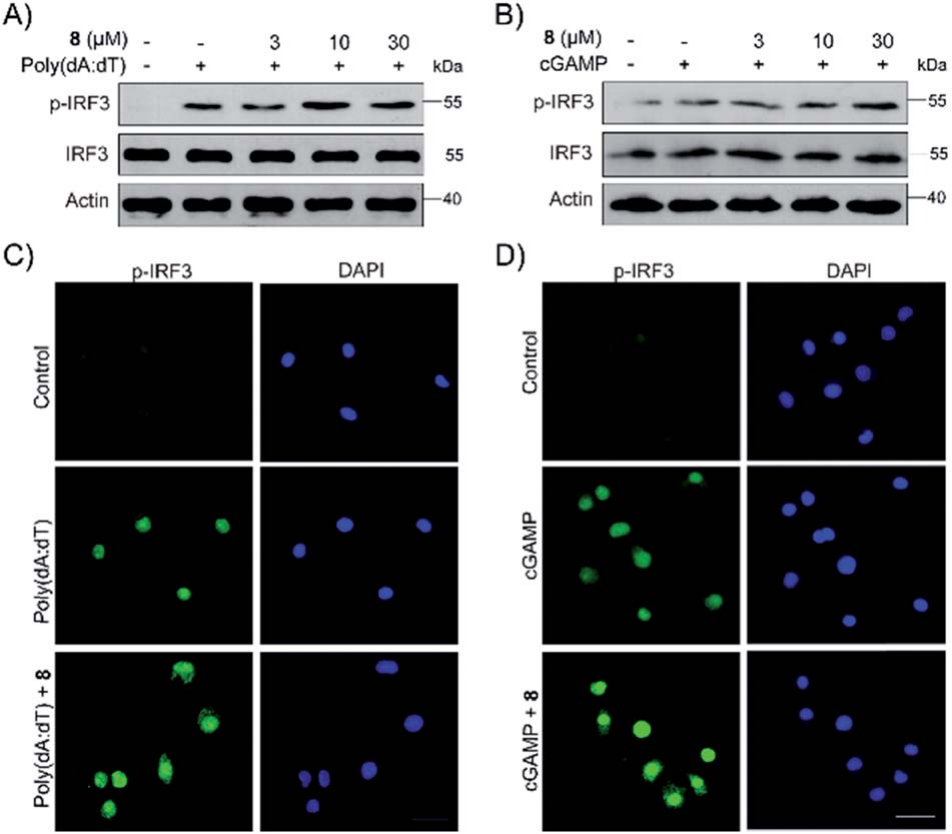
Compound **8** enhanced IRF3 phosphorylation and nuclei localization induced by poly(dA:dT) and cGAMP. (A and B) PMA-differentiated THP1 cells were treated with **8** with or without poly(dA:dT) (2 μg mL^–1^) or cGAMP (1 μg mL^–1^) for 6 h. Proteins of cells were extracted and subjected to immunoblot analysis. (C, D) PMA-differentiated THP1 cells were treated with compound **8** with or without poly(dA:dT) (2 μg mL^–1^) or cGAMP (1 μg mL^–1^) for 3 h. IRF3 expression was examined by immunofluorescence.

Finally, the biological activity of **1**, **2** and **5–11** was tested towards the STING protein, which is a central signalling molecule of the intracellular DNA sensing pathway and is now considered as an effective target for therapeutic intervention.^26^–^28^ As shown in Fig. S15A,† compound **8** significantly promoted IFN-β production induced by poly(dA:dT), whereas **9–11** could inhibit that, and the remaining compounds **1**, **2**, and **5–7** have no or a weak effect on IFN-β production. The STING agonists (cyclic dinucleotides or non-dinucleotides) have demonstrated therapeutic effects in multiple mouse tumor models.^29^,^30^ Moreover, **8** also dose-dependently increased the production of IFN-β induced by poly(dA:dT) as well as cGAMP (Fig. S15B† and C†). Different from cGAMP or amidobenzimidazole STING receptor agonists, **8** itself cannot trigger IFN-β production. To further confirm the effect of **8** on the STING signaling pathway, the phosphorylation of IRF3 was examined by Western blot and immunofluorescence. Compound **8** dose-dependently enhanced the IRF3 phosphorylation induced by poly(dA:dT) as well as cGAMP (Fig. 5Aand B). Moreover, immunofluorescence showed that phosphorylation and nuclei localization of IRF3 was markedly increased after compound 8 treatment (Fig. 5C and D). Taken together, our results showed that 8 is an activator of the STING signalling pathway.

## Conclusions

The recent explosion in genome sequence data clearly indicated that the biosynthetic potential of microorganisms to produce new/bioactive natural products is greatly underappreciated. However, discovery of secondary metabolites has historically been a tedious and laborious process. Here, by employing a protein sequence similarity network, we systematically analyzed KS, KR and DH enzymes in type II polyene PKSs together with other PKSs and FASs. Our analysis indicated that KSs and KRs in type II polyene PKSs are distinct from those in other systems at certain *E* value thresholds and can be used as probes for genome mining. Using these unique KS, KR and isomerases as probes, we identified 192 gene clusters from the genomic database, 51 of which are putative CCNP BGCs. Further analysis through the GNN not only indicated the chemical diversity of these encoded CCNPs but also prioritized the strain hits.

The successful identification of kitacinnamycins as a new class of CCNP-type glycopeptides validates the power of our genome mining strategy in natural product discovery. The biosynthetic machinery of kitacinnamycins was elucidated through gene inactivation studies, *in vitro* biochemical reaction. The X-ray crystal structure of a glycosyltransferase (Kcn28) and site-directed mutagenesis analysis provide structural insight into this glycosylation step. Notably, kitacinnamycin H (**8**), which is produced in a high titer from the Δ*kcn27* strain, exhibited promising STING activation activity. Future studies on the other hits identified here will promise the discovery of more new CCNPs and expand the chemical space of CCNP-type natural products.

## Conflicts of interest

The authors declare no conflict of interest.

## Supplementary Material

Supplementary information
