# Supplementary material for: Genome mining and biosynthesis of kitacinnamycins as a STING activator
Source: Chem Sci. 2019 Apr 2;10(18):4839–46. doi: 10.1039/c9sc00815b (PMC6510318; doi:10.1039/c9sc00815b)
Supplement: Supplementary file 1 [file SC-010-C9SC00815B-s001.pdf]

Electronic Supplementary Material (ESI) for Chemical Science.
This journal is © The Royal Society of Chemistry 2019

Electronic Supplementary Information for

Genome mining and biosynthesis of kitacinnamycin as a STING activator

Jing Shi,[a,†] Cheng Li Liu,[a,†] Bo Zhang,[a] Wen Jie Guo,[a] Jiapeng Zhu,[b] Chin-Yuan Chang,[c] Er Juan Zhao,[a] Rui Hua Jiao,[a] Ren Xiang Tan,[*a,b] and Hui Ming Ge[*a]

a. State Key Laboratory of Pharmaceutical Biotechnology, Institute of Functional Biomolecules, School of Life Sciences, Nanjing University 210023, P.R. China
b. State Key Laboratory Cultivation Base for TCM Quality and Efficacy, Nanjing University of Chinese Medicine, Nanjing 210023, P.R.China
c. Department of Biological Science and Technology, National Chiao Tung University, Hsinchu, R.O. China

† Contributed equally to this work.
* Email: rxtan@nju.edu.cn (R.X.T.); hmge@nju.edu.cn(H.M.G.)

Experimental procedures:
General experimental procedures.
Fermentation and isolation of kitacinnamycins.
Physicochemical data of kitacinnamycins.
LC-MS/MS fragmentation of hydrolyzed products of **1** and **2**.
GC/MS analysis of sugar moieties in **1**.
Determination of the absolute configurations of the amino acid residues in **1** and **2**.
Gene disruption in *Kitasatospora* sp. CGMCC 16924
Gene expression and protein purification.
In vitro assay of Kcn27.
In vitro assay of Kcn28.
Sequence similarity network (SSN) analysis.
Genome neighbouring network (GNN) analysis for putative CCNP gene clusters
Protein crystallization, structural elucidation and docking study.
Site-directed mutagenesis of Kcn28.
Cell culture
Immunoblot assay
Immunofluorescence

Table S1. Bacterial plasmids and strains.
Table S2. Primers used in this study.
Table S3. Deduced functions of ORFs in the *kcn* gene cluster.
Table S4. Prediction of A domain substrate specificity.
Table S5. ^{1}H NMR (600 MHz) and ^{13}C NMR (150 MHz) data of **1** in DMSO-d_6.
Table S6. ^{1}H NMR (600 MHz) and ^{13}C NMR (150 MHz) data of **2** in DMSO-d_6.
Table S7. ^{1}H NMR (600 MHz) and ^{13}C NMR (150 MHz) data of **3** in DMSO-d_6.
Table S8. ^{1}H NMR (600 MHz) and ^{13}C NMR (150 MHz) data of **4** in DMSO-d_6.
Table S9. ^{1}H NMR (600 MHz) and ^{13}C NMR (150 MHz) data of **5** in DMSO-d_6.
Table S10. ^{1}H NMR (600 MHz) and ^{13}C NMR (150 MHz) data of **6** in DMSO-d_6.
Table S11. ^{1}H NMR (600 MHz) and ^{13}C NMR (150 MHz) data of **7** in DMSO-d_6.
Table S12. ^{1}H NMR (600 MHz) and ^{13}C NMR (150 MHz) data of **8** in DMSO-d_6.
Table S13. ^{1}H NMR (600 MHz) and ^{13}C NMR (150 MHz) data of **9** in DMSO-d_6.
Table S14. ^{1}H NMR (600 MHz) and ^{13}C NMR (150 MHz) data of **10** in DMSO-d_6.
Table S15. ^{1}H NMR (600 MHz) and ^{13}C NMR (150 MHz) data of **11** in DMSO-d_6.
Table S16. ^{1}H NMR (600 MHz) and ^{13}C NMR (150 MHz) data of **12** in DMSO-d_6.
Table S17. Data collection and refinement statistics (molecular replacement) of Kcn28 and Kcn28 with **9**.

Figure S1. Structures of known CCNPs including skyllamycin A, WS9326A, mohangamide A, coprisamide A.
Figure S2. Proposed biosynthetic pathway for N-terminal cinnamyl residue biosynthesis.
Figure S3. Structures of ishigamide, colabomycin A, and simocyclinone D8.
Figure S4. Biosynthetic gene clusters for putative CCNPs.
Figure S5. MS/MS fragmentation analysis of hydrolized products of **1** (A) and **2** (B).
Figure S6. LC-MS analysis of L-FDAA derivatives of the amino acids in compound **1** and **2**.
Figure S7. GC-MS analysis of the trimethylsilyl derivatives of the hydrolyzed **1**.
Figure S8. Construction of in-frame deletion in CGMCC 16924.
Figure S9. SDS-PAGE analysis of proteins.
Figure S10. LC-MS analysis of Kcn27-catalyzed reaction.
Figure S11. HRESIMS spectra of **13** (A) and **14** (B).
Figure S12. Crystal structure of Kcn28, OleD and CalG3.
Figure S13. Relative activities of Kcn28 and its site-specific mutants on enzymatic reactions.
Figure S14. Sequence alignment of Kcn28 and its homologues.
Figure S15. Compound **8** dose dependently promote IFN-β production induced by poly(dA:dT) and cGAMP.
Figure S16-S22. 1D and 2D NMR spectrum of **1** in DMSO-d_6.
Figure S23-S30. 1D and 2D NMR spectrum of **2** in DMSO-d_6.
Figure S31-S34. 1D and 2D NMR spectrum of **3** in DMSO-d_6.

Figure S35-S41. 1D and 2D NMR spectrum of **4** in DMSO-d_6.
Figure S42-S48. 1D and 2D NMR spectrum of **5** in DMSO-d_6.
Figure S49-S54. 1D and 2D NMR spectrum of **6** in DMSO-d_6.
Figure S55-S61. 1D and 2D NMR spectrum of **7** in DMSO-d_6.
Figure S62-S68. 1D and 2D NMR spectrum of **8** in DMSO-d_6.
Figure S69-S75. 1D and 2D NMR spectrum of **9** in DMSO-d_6.
Figure S76-S82. 1D and 2D NMR spectrum of **10** in DMSO-d_6.
Figure S83-S89. 1D and 2D NMR spectrum of **11** in DMSO-d_6.
Figure S90-S96. 1D and 2D NMR spectrum of **12** in DMSO-d_6.
References

Experimental Procedures

General experimental procedures.

All 1D and 2D NMR spectra were obtained from a Bruker Avance 600 specrometer at 600 MHz for ^{1}H and 150 MHz for ^{13}C nuclei. HRESIMS were run on an Agilent 6530 TOF LC/MS mass coupled with a Agilent 1260 Infinity HPLC equipped with a Poroshell 120 EC-C18 column (4.5 × 50 mm, 2.7 µm, Agilent Techonologies). GC/MS was conduncted on an Agilent 5977A mass coupled with Agilent 7890B GC system using an HP-5 MS column (30 m, 0.25 mm I.D.). UV-vis absorbance was measured on a Nanodrop 2000c spectrometer (Thermo Scientific) with a 10 mm cuvette. Semipreparative RP-HPLC was performed on an Agilent 1200 HPLC with an Eclipse XDB-C18 column (5 µ, 250×9.4 mm, Agilent Techonologies). MPLC fractionation was conducted on Biotage Isolera One using a Biotage SNAP Catridge C18 column (60 g). PCR amplifications were performed on a Bio-Rad S1000™ Thermal Cycler using Phanta Super-Fidelity DNA Polymerase or 2×Rapid Taq Master Mix (Vazyme Co., Ltd) Polymerase. DNA Sequencing was conducted by TsingKe Biological Technology Co. Recombinant proteins were purified on a GE AKTA pure system with a 5 mL Histrap HP column (GE lifesciences).

Human IFN-beta ELISA Kits were bought from MultiSciences Biotech CO., LTD (Hanghzou, China). Poly(dA:dT) (dsDNA naked-complexed with transfection reagent) and 2'3'-cGAMP was bought from InvivoGen (Toulouse, France). Anti-phospho-IRF3 antibody (37829), anti-IRF3 (4302) were purchased from Cell Signaling Technology. Anti-Actin (sc-1616) were purchased from Santa Cruz Biotechnology. Alexa Fluor 488 goat anti-rabbit IgG (A11008) was purchased from Invitrogen. All other chemicals were obtained from Sigma-Aldrich.

Fermentation and isolation of kitacinnamycins.

For compounds **1**−**6** isolation, fresh spores of *Kitasatospora* sp. CGMCC 16924 were innoculated into five 1-L flasks containing 200 mL of TSB medium (17.0 g tryptone, 3.0 g soytone, 2.5 g glucose, 5.0 g sodium chloride, 2.5 g Na_2HPO_4 in 1 L water, pH7.0). After growing at 28 °C and 160 rpm for 2 days, the seed culture were inoculated into 100×1-L flasks each containing 200 mL of SCAS medium (40g soluble starch, 5 g casamino acid, 0.5 g KH_2PO_4, 0.5 g $MgSO_4·7H_2O$, 0.01 g $FeSO_4·7H_2O$ in 1L water.) and shaken at 160 rpm, 28 °C. After 10 days fermentation, the broth was filtered, absorbed with XAD-16 resin. The resin was washed with water and eluted with methanol. The methanol was removed under reduced pressure to afford 9.2 g crude extract for MPLC fractionation. The MPLC fractions with UV absorption at 300 nm were further purified by semipreparative using a 30-min solvent gradient elution system (CH_3CN/H_2O: 40:60-100:0) at a flow rate of 2 ml/ min to afford **1** (20.7 mg), **2** (35.1 mg), **3** (2.3 mg), **4** (2.1 mg), **5** (2.9 mg), and **6** (2.5 mg).

Compounds **7** (12 mg) and **8** (15 mg) were purified through HPLC from the extract of a large scale fermentation (4 L) of Δ*Kcn27* mutant strain using the same fermentation condition mentioned above.

For compounds **9**−**12** isolation, the wild-type strain of *Kitasatospora* sp. CGMCC 16924 were fermented in 20-L scale for 4 days. The obtained crude extract (7.1 g) was fractionated by MPLC. The MPLC fraction was further analyzed by LC-MS. The fractions with target molecular weight were purified by semipreparative HPLC using a 45-min solvent system, from 40% MeCN to 90% MeCN, to afford **9** (1.1 mg), **10** (4.7 mg), **11** (0.9 mg), **12** (5.6 mg).

Physicochemical data of kitacinnamycins

Kitacinnamycin A (**1**): light yellow amorphous powder; NMR data see Table S5; HRESIMS *m/z* 1447.6492 $[M+Na]^+$ (calcd for $[C_{67}H_{96}N_{10}O_{24}Na]^+$, 1447.6491); $[\alpha]_D^{20}$ = +6.0 (*c* 0.2, MeOH); IR (KBr) ν_{max} 3325, 2963, 2931, 2875, 1671, 1536, 1206, 1140, 1079, 980, 722 cm^{-1}; UV (MeOH): λ_{max} (logε) = 266 (4.43), 296 nm (4.39).

Kitacinnamycin B (**2**): light yellow amorphous powder; NMR data see Table S6; HRESIMS *m/z* 1461.6642 $[M+Na]^+$ (calcd for $[C_{68}H_{98}N_{10}O_{24}Na]^+$, 1461.6648); $[\alpha]_D^{20}$ = +11.0 (*c* 0.2, MeOH); IR (KBr) ν_{max} 3338, 2968, 2937, 2879, 1662, 1538, 1215, 1174, 1078, 978, 702 cm^{-1}; UV (MeOH): λ_{max} (logε) =266 (4.44), 296 nm (4.16).

Kitacinnamycin C (**3**): light yellow amorphous powder; NMR data see Table S7; HRESIMS *m/z* 1312.6447 $[M+Na]^+$ (calcd for $[C_{63}H_{91}N_{11}O_{18}Na]^+$, 1312.6436) UV (MeOH): λ_{max} (logε) =260 (4.36), 288 nm (4.35).

Kitacinnamycin D (**4**): light yellow amorphous powder; NMR data see Table S8; HRESIMS *m/z* 1326.6580 $[M+Na]^+$ (calcd for $[C_{64}H_{93}N_{11}O_{18}Na]^+$, 1326.6592) UV (MeOH): λ_{max} (logε) =260 (4.32), 288 nm (4.29).

Kitacinnamycin E (**5**): light yellow amorphous powder; NMR data see Table S9; HRESIMS *m/z* 1123.5435 $[M+Na]^+$ (calcd for $[C_{55}H_{76}N_{10}O_{14}Na]^+$, 1123.5435); $[\alpha]_D^{20}$ = -4.0 (*c* 0.2, MeOH); IR (KBr) ν_{max} 3397, 2966, 2876, 1661, 1534, 1207, 1179, 1074, 978, 702 cm^{-1}; UV (MeOH): λ_{max} (log ε) =262 (4.20), 296 nm (4.16).

Kitacinnamycin F (**6**): light yellow amorphous powder; NMR data see Table S10; HRESIMS *m/z* 1137.5588 $[M+Na]^+$ (calcd for $[C_{56}H_{78}N_{10}O_{14}Na]^+$, 1137.5591); $[\alpha]_D^{20}$ = -6.0 (*c* 0.2, MeOH); IR (KBr) ν_{max} 3307, 2965, 2876, 1662, 1532, 1206, 1073, 978, 701 cm^{-1}; UV (MeOH): λ_{max} (log ε) =262 (4.11), 296 nm (4.07).

Kitacinnamycin G (**7**): light yellow amorphous powder; NMR data see Table S11; HRESIMS *m/z* 1093.5688 $[M+Na]^+$ (calcd for $[C_{55}H_{78}N_{10}O_{12}Na]^+$, 1093.5693); $[\alpha]_D^{20}$ = +4.0 (*c* 0.2, MeOH); IR (KBr) ν_{max} 3305, 2965, 2875, 1659, 1532, 1209, 1176, 1073, 972, 701 cm^{-1}; UV (MeOH): λ_{max} (log ε) =248 (4.27), 288 nm (4.28).

Kitacinnamycin H (**8**): light yellow amorphous powder; NMR data see Table S12; HRESIMS *m/z* 1107.5845 $[M+Na]^+$ (calcd for $[C_{56}H_{80}N_{10}O_{12}Na]^+$, 1107.5849); $[\alpha]_D^{20}$ = +9.0 (*c* 0.2, MeOH); IR (KBr) ν_{max} 3311, 2967, 2877, 1660, 1534, 1208, 1175, 1074, 970, 701 cm^{-1}; UV (MeOH): λ_{max} (log ε) =248 (4.16), 288 nm (4.15).

Kitacinnamycin I (**9**): light yellow amorphous powder; NMR data see Table S13; HRESIMS *m/z* 1109.5656 $[M+Na]^+$ (calcd for $[C_{55}H_{78}N_{10}O_{13}Na]^+$, 1109.5642); UV (MeOH): λ_{max} (log ε) =253 (2.91), 310 nm (3.07).

Kitacinnamycin J (**10**): light yellow amorphous powder; NMR data see Table S14; HRESIMS *m/z* 1123.5792 $[M+Na]^+$ (calcd for $[C_{56}H_{80}N_{10}O_{13}Na]^+$, 1123.5799); UV (MeOH): λ_{max} (log ε) =248 (3.97), 288 nm (3.98).

Kitacinnamycin K (**11**): light yellow amorphous powder; NMR data see Table S15; HRESIMS *m/z* 1085.5670 $[M+Na]^+$ (calcd for $[C_{55}H_{76}N_{10}O_{13}Na]^+$, 1085.5666); UV (MeOH): λ_{max} (log ε) =273 (4.15), 296 nm (4.11).

Kitacinnamycin L (**12**): light yellow amorphous powder; NMR data see Table S16; HRESIMS *m/z* 1121.5640 $[M+Na]^+$ (calcd for $[C_{56}H_{78}N_{10}O_{13}Na]^+$, 1121.5642); UV (MeOH): λ_{max} (log ε)=273 (4.23), 296 nm (4.24).

LC-MS/MS fragmentation of hydrolyzed products of 1 and 2.[1]

The MeCN solution (0.25 mL) of compound **1** (1 mg) was treated with H_2O (0.25 mL) and concentrated NH_4OH (10 μL) at room temperature for 4 h to break the ester bond in **1**. After the mixture was dried in vacuo, it was redissolved in MeOH and analyzed by LC-ESI-MS/MS. The LC-MS/MS analysis was performed using a 18 min solvent gradient from 10% to 90% (0–15 min) and 100% (15–18 min) MeCN in water supplied with 0.1 TFA at a flow rate of 0.5 mL/min. Compound **2** was treated with the same manner, and then analyzed by LC-MS/MS (Figure S5).

GC/MS analysis of sugar moieties in 1.

The dried hydrolyzed product of **1** obtained above was treated with 1-(trimethylsilyl)imidazole in pyridine 1:4 (v/v), and headted at 60 °C for 30 minutes. The afforded derivative was injected to GC/MS for analysis. The GC/MS was performed on an Agilent 7890B gas chromatograph with 5977A MSD mass spectrum using an HP-5 MS column (30 m, 0.25 mm I.D.). GC/MC method: 100 method starts at 100°C holds the oven at this temperature for 1 minute, then ramp of 50 °C/min till 250°C and hold the oven at this temperature for 3 minutes. The carried gas (helium) flow was 1.5 mL/min. See Figure S7.

Determination of the absolute configurations of the amino acid residues in 1 and 2.[2]

Compound **1** (1 mg) was hydrolyzed using 0.5 mL of 6 N HCl for 2 h at 115 °C in a sealed tube. The reaction vial was then cooled in ice-water for 3 min, and was then evaporated in vacuo. The remaining trace amount of HCl was further removed by adding 2 mL of water and evaporating the solvent two times. The obained hydrolysate was then lyophilized. The hydrolysate containing the free amino acids was divided into two 8-mL vials and then re-dissolved in 100 μL of 1 N $NaHCO_3$. To one of the vials containing the dissolved free amino acids, 50 μL of 10 mg/mL L-FDAA (1-fluoro-2,4-dinitrophenyl-5-L-alanine amide) in acetone was added. The reaction mixtures were incubated at 80 °C for 10 min. A 50-μL aliquot of 2 N HCl was added to neutralize the reaction, followed by the addition of 300 μL of aqueous 50 % CH_3CN. An aliquot of each reaction mixture was analyzed by LC/MS using a gradient solvent system (flow rate: 0.5 mL/min, 10% to 90% MeCN in water (0–15 min) and 100% MeCN in water (15–18 min), supplied with 0.1 TFA). The absolute configurations of amino acid residues were determined through comparison of corresponding amino acid standard.

Using the same process mentioned above, the absolute configuration of the amino acid resiudes in compound **1** and **2** were determined (See Figure S6).

Gene disruption in *Kitasatospora* sp. CGMCC 16924

Double cross-over homologous recombination was used for the gene (*kcn7*, *kcn14*, *kcn22*, *kcn27* and *kcn28*) disruption. Briefly, the upstream and downstream homology arms were amplified with primers up-F/R and down-F/R (Table S2) using genomic DNA of CGMCC 16924 as template, respectively. The purified PCR products were ligated to *Hin*dIII and *Eco*RI linearized pKC1139 to generate mutation carring plasmids (pHG5001−pHG5005). After confirmation by DNA sequencing, the individual plasmid (pHG5001−pHG5005) carring mutation were transformed into *E. coli* ET12567/pUZ8002 and further conjugated into *Kitasatospora* sp. CGMCC 16924, respectively, following the standard procedure.[3] After 5 days cultivation, the apramycin resistant colonies were picked and streaked onto ISP4 plates supplied with apramycin antibiotics at final concentration of 50 μg/mL and cultured at 30 °C. The positive colonies showing apramycin sensitive phenotype were picked as candidate for double cross-over homologous recombination. The genotypes of these candidate clones were confirmed by diagnostic PCR analysis using the primers listed in Table S2.

Gene expression and protein purification.

DNA fragment of *kcn27* was amplified from genomic DNA of CGMCC 16924 with primers listed in Table S2. The purified PCR products ligated with linearized pET22b (treated with *Nde*I and *HindIII*) to afford pHG5006. And DNA fragments of *kcn28*, ferredoxin gene (ctg1_4402) and ferredoxin reductase gene (ctg1_1556) were individually amplified from genomic DNA of *CGMCC* 16924 with primers listed in Table S2. The purified PCR products were ligated with linearized pET28a (treated by *Nde*I and *Hin*dIII) to afford pHG5007-pHG5009, respectively. The obtained pHG5006-pHG5009 were further introduced into *E. coli* BL21(DE3), respectively. The transformants were cultivated in 400 mL LB medium supplemented with 50 μg/mL Ampicillin (pHG5006) or 50 μg/mL Kanamycin (pHG5007−pHG5009) at 37 °C (220 rpm) until OD_{600} value reached around 0.6. The culture was cooled to 4 °C and induced with 0.1 mM IPTG, and continued to cultivate at 16 °C (220 rpm) for 18 h. After harvesting the cells by centrifugation at 4000 g for 10 min, the pellet were resuspended in lysis buffer (100 mM Tris, pH 8.0, 15 mM imidazole, 300 mM NaCl, 10 % glycerol), lysed on ice by sonication, and centrifuged at 15,000 rpm for 30 min at 4 °C. The supernatant containing overproduced protein was filtered and purified by ÄKTA FPLC system equipped with a 5 mL Histrap HP column (GE lifesciences).The proteins were pooled and desalted by a PD10 column (GE Healthcare) with 100 mM phosphate buffer (pH 7.0) and 10% glycerol and stored at -80°C.

***In vitro* assay of Kcn27.**

The Kcn27-catalyzed reaction was carried out in a 100 μL reaction system containing 50 mM MES buffer (pH 5.8), 100 μM substrate, 5 mM NADPH, 2 μM FDR, 2 μM FDX and 5 μM Kcn27. After incubation at 30°C for 1 h, the reaction was quenched by adding 100 μL methanol. The reaction mixture was then centrifuged at 15,000 g for 10 min at room temperature, and the afforded supernatant was analyzed by LC-MS. LC-MS analysis was conducted using a 18 min solvent gradient from 10% to 90% (0–15 min) and 100% (15–18 min) MeCN in water supplied with 0.1 TFA at a flow rate of 0.5 mL/min.

***In vitro* assay of Kcn28.**

The Kcn28 reaction solution (100 µL) was performed in 100 mM phosphate buffer (pH 6.8) containing 5 µM Kcn28, 100 µM substrate, 1 mM UDP-glucose/UDP-GlcNAc. Reaction was incubated at 30 °C for 1 h, and terminated by adding 100 µL methanol. The mixture was centrifuged at 15,000 g for 10 min, and the supernatant was analyzed by LC-MS. LC-MS analysis was performed using a 18 min solvent gradient from 10% to 90% (0–15 min) and 100% (15–18 min) MeCN in water supplied with 0.1 TFA at a flow rate of 0.5 mL/min. UDP-glucose and UDP-GlcNAc were obtained from Sigma-Aldrich.

Sequence similarity network (SSN) analysis.[4]

The KS protein sequences for the SSN analysis include representative sequences from **polyene type II PKSs** (Cal30, ALG65306.1, *Streptomyces calvus*; Cal31, ALG65305.1, *Streptomyces calvus*; Cal32, ALG65304.1, *Streptomyces calvus*; Cal33, ALG65303.1, *Streptomyces calvus*; Sky17, AEA30260.1, *Streptomyces* sp. Acta 2897; Sky18, AEA30261.1, *Streptomyces* sp. Acta 2897; Sky19, AEA30262.1, *Streptomyces* sp. Acta 2897; Sky22, AEA30265.1, *Streptomyces* sp. Acta 2897; Iga11, BAX64252.1, *Streptomyces* sp. MSC090213JE08; Iga12, BAX64253.1, *Streptomyces* sp. MSC090213JE08; ColC3, AIL50165.1, *Streptomyces aureus*; ColC4, AIL50166.1, *Streptomyces aureus*; ColC13, AIL50179.1, *Streptomyces aureus*; ColC14, AIL50180.1, *Streptomyces aureus*; AsuC13, ADI58650.1, *Streptomyces nodosus* subsp. *Asukaensis*; AsuC14, ADI58649.1, *Streptomyces nodosus* subsp. *Asukaensis*; Sim-ORF2, AEU17884.1, *Streptomyces antibioticus*; Sim-ORF3, AEU17885.1, *Streptomyces antibioticus*; SmcKSII, ALT05934.1, *Kitasatospora* sp. 152608; SmcX5, ALT05939.1, *Kitasatospora* sp. 152608); **aromatic type II PKSs** (BenA, CAM58798.1, *Streptomyces* sp. A2991200; BenB, CAM58799.1, *Streptomyces* sp. A2991200; JadA, AAB36562.1, *Streptomyces venezuelae* ATCC 10712; JadB, AAB36563.1, *Streptomyces venezuelae* ATCC 10712; DpsA, AAA65206.1, *Streptomyces peucetius*; DpsB, AAA65207.1, *Streptomyces peucetius*; MtmP, CAA61989.1, *Streptomyces argillaceus*; MtmK, CAA61990.1, *Streptomyces argillaceus*; OxyA, AAZ78325.1, *Streptomyces rimosus*; OxyB, AAZ78326.1, *Streptomyces rimosus*; ActI, CAC44200.1, *Streptomyces coelicolor* A3 (2); AknB, AAF70106.1, *Streptomyces galilaeus*; WhiE1, CAB45606.1, *Streptomyces coelicolor* A3 (2); WhiE2, CAB45607.1, *Streptomyces coelicolor* A3 (2); TcmK, CCK26894.1, *Streptomyces davaonensis* JCM 4913; TcmL, CCK26893.1, *Streptomyces davaonensis* JCM 4913; ZhuA, AAG30188.1, *Streptomyces* sp.R1128; ZhuB, AAG30189.1, *Streptomyces* sp.R1128; Snoa1, CAA12017.1, *Streptomyces nogalater*; Snoa2, CAA12018.1, *Streptomyces nogalater*; OxyD, AAZ78328.1, *Streptomyces rimosus*; ZhuH, AAG30195.1, *Streptomyces* sp. R1128; CmmP, CAE17527.1, *Streptomyces griseus*; LanA, AAD13536.1, *Streptomyces cyanogenus*; UrdA, Q54173, *Streptomyces fradiae*; LanB, AAD13537.1, *Streptomyces cyanogenus*; AknC, AAF70107.1, *Streptomyces galilaeus*; EncA, AIN46688.1, *Streptomyces qinglanensis*; EncB, AIN46689.1, *Streptomyces qinglanensis*); **FASs** (EC-FabF, EGT67882.1, *Escherichia coli* 0104; Ec-FabH, CDL30502.1, *Escherichia coli* ISC7; Ec-FabB, EIQ69853.1, *Escherichia coli* EPEC C342-62; Sc-FabH, CAB62720.1, *Streptomyces coelicolor* A3 (2)), and **KS domains from type I PKSs** (ChlA3, AAZ77696.1, *Streptomyces antibioticus*; ChlB1, AAZ77673.1, *Streptomyces antibioticus*; AviM, AAK83194.1, *Streptomyces viridochromogenes* Tue57; FscC, AAQ82564.1, *Streptomyces* sp. FR-008; FscD, AAQ82568.1, *Streptomyces* sp. FR-008; CalO5, AAM70355.1, *Micromonospora echinospora*; CalE8, AAM94794.1, *Micromonospora echinospora*; DynE8, ACB47048.1, *Micromonospora chersina*; SgcE, Q8GME1, *Streptomyces globisporus*; AmphC, AJE44524.1, *Streptomyces nodosus*; GdmAIII, AA006918.1, *Streptomyces hygroscopicus*).

The KR proteins for the SSN analysis include representative sequences from **polyene type II PKSs** (Sky26, AEA30269.1, *Streptomyces* sp.Acta 2897; Cal21, ALG65315.1, *Streptomyces calvus*; Cal37, ALG65299.1, *Streptomyces calvus*; SmcC6, ALT05968.1, *Kitasatospora* sp. 152608; ColC7, AIL50182.1, *Streptomyces aureus*; ColC10, AIL50168.1, *Streptomyces aureus*; Iga13, BAX64254.1, *Streptomyces* sp. MSC090213JE08; SimA6, AAK06787.1, *Streptpmyces antibioticus*; SimJ2, AAL15605.1, *Streptpmyces antibioticus*;), **aromatic type II PKSs** (Erd5, ACX83621.1, uncultures soil bacterium V167; Act_KR, CAC44199.1, *Streptomyces coelicolor* A3 (2); Med_ORF6, BAC79042.1, *Streptomyces* sp. AM-7161; Hed_KR, AGK78907.1, *Streptomyces fulvissimus* DSM 4053); **FASs** (Sv_FabG, CCA54193.1, *Streptomyces venezuelae* ATCC 10712; Sc_FabG, NP_625631.1, *Streptomyces coelicolor* A3 (2); Mp_FabG, RDY06442.1, *Mucuna pruriens*; Cc_FabG, AVH88979.1, *Corynebacterium camporealensis*; Li_FabG, CAC97151.1, *Listeria innocua* Clip11262; Kp_FabG, CDO13934.1, *Klebsiella pneumonia*; Bs_FabG, AHN48141.1, *Brucella suis* bv.1 str. S2), and **KR domains from type I PKSs** (RapB, CAA60459.1, *Streptomyces rapamycinicus* NRRL 5491; Lip Pks2, ABB05103,1, *Kitasatospora aureofaciens*; AmphC, AJE44524.1, *Streptomyces nodosus*; NysC, AAF71776.1, *Streptomyces noursei* ATCC 11455; Tyl, AAB66508.1, *Streptomyces fradiae*; PimS2, CAC20921.1, *Streptomyces natalensis*; PikIII, WP_055641629.1, *Streptomyces venezuelae*; NysB, AAF71775.1, *Streptomyces noursei* ATCC 11455; AveA1, BAC68648.1, *Streptomyces avermitilis* MA 4680; EryAI, AAV51820.1, *Saccharopolyspora erythraes*).

The DH proteins for the SSN analysis include representative sequences from **polyene type II PKSs** (Sky24, AEA30267.1, *Streptomyces* sp.Acta 2897; Sky25, AEA30268.1, *Streptomyces* sp.Acta 2897; Cal35, ALG65297.1, *Streptomyces calvus*; Cal36, ALG65298.1, *Streptomyces calvus*; ColC8, AIL50173.1, *Streptomyces aureus*; ColC9, AIL50172.1, *Streptomyces aureus*; Iga16, BAX64257.1, *Streptomyces* sp. MSC090213JE08; SimA5, AAK06788.1, *Streptpmyces antibioticus*; AsuC8, ADI58642.1, *Streptomyces nodosus* subsp. *Asukaensis*; AsuC9, ADI58641.1, *Streptomyces nodosus* subsp. *Asukaensis*;), **aromatic type II PKSs** (SnogH, CAA12009.1, *Streptomyces nogalater*; LipDig5, ABB05111.1, *Kitasatpspora aureofaciens*; JadH, AAB36566.1, *Streptomyces venezuelae* ATCC 10712; UrdQ, AAF72550.1, *Streptomyces fradiae*; UrdS, AAF72552.1, *Streptomyces fradiae*;), **FASs** (Ac_FabZ, SCD16274.1, *Acinetobacter calcoaceticus*; Ba_FabZ, AAA96790.1, *Brucella abortus*; Bv_FabZ, ABS75686.1, *Bacillus velezensis* FZB42; Ec_FabZ, ATZ31741.1, *Escherichia coli*), and **DH domains from type I PKSs** (MerA, ABJ97437.1, *Streptomyces violaceusniger*; MerB, ABJ97438.1, *Streptomyces violaceusniger*; MerC, ABJ97439.1, *Streptomyces violaceusniger*; NidA1, AAC46024.1, *Streptomyces caelestis*; NidA2, AAC46025.1, *Streptomyces caelestis*; NidA3, AAC46026.1, *Streptomyces caelestis*; NysA, AAF71774.1, *Streptomyces noursei* ATCC 11455; NysC, AAF71776.1, *Streptomyces noursei* ATCC 11455; PokS2, ACN64841.1, *Streptomyces diastatochromogenes*; PokS3, ACN64829.1, *Streptomyces diastatochromogenes*; PokS5, ACN64825.1, *Streptomyces diastatochromogenes*).

For SSN analysis, an initial *E* value of 10^{-10} was used from the local blast analysis (all *vs* all). The *E* values were converted into intergers using –log(*E* value), and the *E* value of 0 was manually assigned as 200. Cytoscape 3.7.0 were used for network visualization. Both self loops and duplicate loops were deleted. Finally, the *E* values of 10^{-70}, 10^{-55}, and 10^{-15} were chosen for KS, KR, and DH figure generation, respectively.

Genome neighbouring network (GNN) analysis for putative CCNP gene clusters

For GNN analysis, all genes within the putative CCNP gene clusters were collected and translated. The total 3196 proteins from 53 BGCs (51 new identified BGCs plus two known BGCs (*sky* and *cal*)) were used for all-*vs*-all BLAST analysis with an initial *E* value of 10^{-5}. The obtained *E* value from all-*vs*-all BLAST analysis were converted to intergers using –log(*E* value), and the *E* value of 0 was manually assigned as 200. Cytoscape 3.7.0 were used for newwork visualization. The self loops and duplicate loops were deleted. The *E* value of 10^{-30} was chosen for figure generation.

Protein crystallization, structural elucidation and docking study.

The purified Kcn28 was incubated with thrombin to remove the N-terminal His tag. Crystals were grown at 4 °C with the sitting-drop vapor-diffusion method. Drops consisted of 1:1 ratio of proteins (10 mg/ml, 50 mM NaCl, 20 mM Tris, pH8.0) and crystallization buffer (1.6 M Ammonium sulfate, 0.1 M MES monohydrate pH 6.5, 10% v/v 1,4-Dioxane). Meanwhile, Kcn28-substrates complex was also achieved by crystallizing Kcn28 with different substrates at up to 1:10 ratio. Crystals of Kcn28 and Kcn28-**9** complex were directly flash frozen in liquid nitrogen.

An single wavelength anomalous diffraction data of the Kcn28-**9** complex was collected at BL18U1 beamline at the Shanghai Synchrotron Radiation Facility (SSRF) at wavelengths of 0.97930 Å, while the data of Kcn28 was collected at BL18U1 beamline at SSRF at wavelengths of 0.97894 Å. All diffraction datasets were processed and scaled using imosflm.[5] The phase problem of the Kcn28 and complex was solved by the molecular replacement method using the structure of the OleD protein (PDB ID: 2IYF) as the search model with PHASER,[6] and further autobuilded and refined by PHENIX,[7] COOT was used for manually model rebuilding and adjustments.[8] Finally, additional TLS refinement was performed in PHENIX. The final refinement statistics are listed in Table S17. Structural diagrams were prepared using the program PyMOL (http:// www.pymol.org/). The UDP was docked into the UDP binding pocket by using Autodock Vina.[9]

Site-directed mutagenesis of Kcn28

Mutated fragments were amplified with primers listed in Table S2 by using plasmid pHG5007 as template. The purified PCR products were incubated with DpnI, T4 polynucleotide kinase and T4 DNA ligase, according to the standard procedure of Q5® Site-Directed Mutagenesis Kit purchased from NEB (USA). Each mutation was confirmed by sequencing. The recombined plasmids were expressed in *E. coli* BL21(DE) and purified as described above for native protein.

Cell culture

Human monocytic THP-1 cell line was purchased from Shanghai Institute of Cell Biology (Shanghai, China) and cultured at 37 °C in a 5% (v/v) CO_2 atmosphere. Before further stimulation, THP-1 cells were treated with PMA (500 nM) for 3 h.

Immunoblot assay

Immunoblot assay was performed as described previously.[10] Briefly, proteins were extracted in lysis buffer. The proteins were then separated by SDS–polyacrylamide gel electrophoresis (PAGE) and electrophoretically transferred onto polyvinylidene difluoride membranes. The membranes were probed with antibodies overnight at 4 °C, and then incubated with a horseradish peroxidase-coupled secondary antibody. Detection was performed using a LumiGLO chemiluminescent substrate system.

Immunofluorescence

PMA-differentiated THP1 cells (adhered to coverslips) were treated with either poly(dA:dT) or 30 μM compound 6 for 3 h. Then cells were fixed with 4% paraformaldehyde (30 min, room temperature), stained with anti-p-IRF3 antibody (1:100), and detected with a secondary antibody (Alexa Fluor 488 goat anti-rabbit IgG, 1:250). The coverslips were counterstained with 0.1 µg/ml DAPI and imaged by fluorescence microscopy.

Table S1. Bacterial plasmids and strains.

Plasmid/Strain	Relevant characteristics	Source
**Plasmid**		
pKC1139	*E.coli*-Streptomyces shuttle plasmid used for gene disruption, temperature sensitive	Ref. 11
pET28a(+)	Protein expression vector used in *E.coli*, encoding N-terminal His-tag, kanamycin resistance	Novagen
pET22b(+)	Protein expression vector used in *E.coli*, encoding N-terminal His-tag, Ampicillin resistance	Novagen
pHG5001	pKC1139 derived plasmid for disruption of *Kcn7*	This study
pHG5002	pKC1139 derived plasmid for disruption of *Kcn14*	This study
pHG5003	pKC1139 derived plasmid for disruption of *Kcn22*	This study
pHG5004	pKC1139 derived plasmid for disruption of *Kcn27*	This study
pHG5005	pKC1139 derived plasmid for disruption of *Kcn28*	This study
pHG5006	pET22b(+) derived plasmid for expressing N-terminal His-tag Kcn27	This study
pHG5007	pET28a(+) derived plasmid for expressing N-terminal His-tag Kcn28	This study
pHG5008	pET28a(+) derived plasmid for expressing N-terminal His-tag Ctg1_1556	This study
pHG5009	pET28a(+) derived plasmid for expressing N-terminal His-tag Ctg1_4402	This study
***E. coli* strains**		
DH5α	General cloning host	Ref. 12
BL21 (DE3)	Heterologous host for protein expression	NEB
ET12567 (pUZ8002)	Methylation-deficient host used for *E. coli-Streptomyces* intergeneric conjugation	Ref. 11
***Streptomyces* strains**		
CGMCC 16924	Wild type strain for kitacinnamycins production	This study
HG5001	*Δkcn7*, in-frame deletion mutant strain	This study
HG5002	*Δkcn14*, in-frame deletion mutant strain	This study
HG5003	*Δkcn22*, in-frame deletion mutant strain	This study
HG5004	*Δkcn27*, in-frame deletion mutant strain	This study
HG5005	*Δkcn28*, in-frame deletion mutant strain	This study

Table S2. Primers used in this study.

Name	Sequence
kcn7-up-F	AACGACGGCCAGTGCCAAGCTTCCGCGACCTCGACTTCAT
kcn7-up-R	GCACTGGAGGACGCCGAGGGTCCCGCTCACCACGAC
kcn7-down-F	GTCGTGGTGAGCGGGACCCTCGGCGTCCTCCAGTGC
kcn7-down-R	AGCTATGACATGATTACGAATTCTCATGTCAGCTCCGTTGTTCTC
kcn14-up-F	AACGACGGCCAGTGCCAAGCTTAGAACAACGGAGCTGACATGAG
kcn14-up-R	GATGATCAGGGTGTCGCCCAGCATCGGGAAGCGGTG
kcn14-down-F	CACCGCTTCCCGATGCTGGGCGACACCCTGATCATC
kcn14-down-R	AGCTATGACATGATTACGAATTCCTTTCAGCACGGTCAGGAA
kcn22-up-F	AACGACGGCCAGTGCCAAGCTTTGTGCGCCACCATGAGTTC
kcn22-up-R	CAACCGCAACGCACCGTCGTTGGCGTGCCGGTGGAT
kcn22-down-F	ATCCACCGGCACGCCAACGACGGTGCGTTGCGGTTG
kcn22-down-R	AGCTATGACATGATTACGAATTCGTCCGTGCTGAACTCGATGAA
kcn27-up-F	AACGACGGCCAGTGCCAAGCTTTGCCAATGCTCGGTGAATCT
kcn27-up-R	CCGGTACTGGAGCTCCTGGGCAGTGTTGGTCTCGCT
kcn27-down-F	AGCGAGACCAACACTGCCCAGGAGCTCCAGTACCGG
kcn27-down-R	AGCTATGACATGATTACGAATTCGACATTCGCCCAATTCCCTT
kcn28-up-F	AACGACGGCCAGTGCCAAGCTTGCGACTACCACGACTTCATCAT
kcn28-up-R	GTCTCAGGCGGGCAGTTGGACGCTGACCACGGCGAT
kcn28-down-F	ATCGCCATGAGCGTCCAACTGCCCGCCTGAGAC
kcn28-down-R	AGCTATGACATGATTACGAATTCGACCATGACCGTTTCCATCCA
kcn7-PO-F	GACGCGTACCACGTGAC
kcn7-PO-R	GATGCCGTACTCGGTGAAG
kcn7-NE-F	TGGTGGTCAGCTCCAACTA
kcn7-NE-R	CAGGAAGGCGTCCCAGA
kcn14-PO-F	TCGCGCTGGAGAAGAAGTA
kcn14-PO-R	TCGTTGGACTGGTAGCAGAA
kcn14-NE-F	AAGCCGGACAAGGAGGA
kcn14-NE-R	ACCAGGACTCCATCAGCA
kcn22-PO-F	GACAGCATCATCTCCATCCAA
kcn22-PO-R	CGACGGCACCATGTACTC
kcn22-NE-F	CACTACCCGATCACCTTCTTC
kcn22-NE-R	ACGTCCTCCTCTTCGATCA
kcn27-PO-F	TTCCTATCCTCGGCGTTCAT
kcn27-PO-R	TGAGATCTGGATCGACGGAATC
kcn27-NE-F	CTGACGCCGGACTTCATC
kcn27-NE-R	GATCGGTTCGGTGGTGTAG
kcn28-PO-F	GAGACCCTCCGGTACGA
kcn28-PO-R	GTTCCACCAGAACCTGGAA
kcn28-NE-F	GTCACGTACGCCAACGAC
kcn28-NE-R	AACAGGCCCTCCTGGAT
Kcn27-pET 22b-F	AAGAAGGAGATATACATATGAGCGAGACCAACACT
Kcn27-pET 22b-R	CTCGAGTGCGGCCGCAAGCTTGAGCACGACCGGCAGCGC
1556-pET 28a-F	GGTGCCGCGCGGCAGCCATATGGACACCGGGATCGTG
1556-pET 28a-R	GCTCGAGTGCGGCCGCAAGCTTTCAGAGTTCGTCCAGCGG
4402-pET 28a-F	GGTGCCGCGCGGCAGCCATATGGTGACCTACGTCATCGCG
4402-pET 28a-R	GCTCGAGTGCGGCCGCAAGCTTTCAGTGCTCGGCGTTCTG
Kcn28-pET 28a-F	GGTGCCGCGCGGCAGCCATATGCCCCGTCCTGGCCAT
Kcn28-pET 28a-R	GCTCGAGTGCGGCCGCAAGCTTTCAGGCGGGCAGTTGCCG
Kcn28-17-F	GTCCCGCGGCgcaCTCCACCCCAGC
Kcn28-17-R	GGGACGCTGACCATGGCG
Kcn28-74-F	CGTCACGGACgccATCGCCCAGATGGACG
Kcn28-74-R	CGGCCCTCGGTGGTGTCG
Kcn28-77-F	CCAGATCGCCgccATGGACGTCTTCCTCGACGAC
Kcn28-77-R	TCCGTGACGCGGCCCTCG
Kcn28-81-F	GATGGACGTCgccCTCGACGACGC
Kcn28-81-R	TGGGCGATCTGGTCCGTG
Kcn28-108-F	CCTCTACGACgccCTGGCCTACC
Kcn28-108-R	AAGACGTCGGGCCGGTCC
Kcn28-109-F	CTACGACGTCgccGCCTACCCGGCCC

Kcn28-109-R	AGGAAGACGTCGGGCCGG
Kcn28-131-F	CTCACCGACCgccGTCATGCCGG
Kcn28-131-R	ATCTGGATCGACGGAATC
Kcn28-137-F	GCCGGAGAAGttcCGGGAGCGGA
Kcn28-137-R	ATGACCCAGGTCGGTGAGATCTG
Kcn28-158-F	CGCGGCGCACttcCGCCGCTTCG
Kcn28-158-R	CCGCGCGGGTCCTGCTTC
Kcn28-180-F	GGACCTCGTCgccCTGCCGGAGCGCAG
Kcn28-180-F	CCGGCGTCGATCCCGGGG
Kcn28-237-F	CTCCCTGGGCgccCACCTGACCAAC
Kcn28-237-R	ACCAGGGCGACCTTCTCG

Table S3. Deduced functions of ORFs in the *kcn* gene cluster

ORF	Amino acids[a]	Blastp homologue	Identity/coverage [%]	Protein ID[b]
Kcn1	738	YdfJ, membrane protein	57/96	AKJ10629.1
Kcn2	411	BaeS, histidine kinase	64/97	AJC53597.1
Kcn3	223	CitB, LuxR family regulator	75/98	GCB49075.1
Kcn4	82	Cal29, acyl carrier protein	79/81	ALG65307.1
Kcn5	413	Cal30, 3-oxoacyl-ACP synthase	85/100	ALG65306.1
Kcn6	379	Cal31, 3-oxoacyl-ACP synthase	58/99	ALG65305.1
Kcn7	313	Cal32, 3-oxoacyl-ACP synthase	52/98	ALG65304.1
Kcn8	300	Cal25, hydrolase	37/87	ALG65311.1
Kcn9	339	Cal26, thioesterase	49/99	ALG65310.1
Kcn10	369	Cal33, 3-oxoacyl-ACP synthase	56/97	ALG65303.1
Kcn11	92	Cal34 acyl carrier protein	46/80	ALG65302.1
Kcn12	146	Cal35, 3-oxoacyl-ACP dehydratase	56/82	ALG65301.1
Kcn13	171	Cal36, 3-oxoacyl-ACP dehydratase	54/90	ALG65300.1
Kcn14	248	Cal37, 3-oxoacyl-ACP reductase	71/100	ALG65299.1
Kcn15	208	Cal27, isomerase	50/93	ALG65309.1
Kcn16	689	UbiB, putative ABC1 family protein	64/96	BAG20104.1
Kcn17	131	hypothetical protein	62/100	SCF61678.1
Kcn18	135	hypothetical protein	66/98	SCF61683.1
Kcn19	112	hypothetical protein	49/97	SCF61687.1
Kcn20	172	hypothetical protein	48/91	PNG92345.1
Kcn21	5661	LgrD, NRPS	46/94	SPX79649.1
Kcn22	1040	LpmD, NRPS	53/94	AEG64698.1
Kcn23	5017	EntF, NRPS	45/94	AHD20734.1
Kcn24	332	Ehm61, hypothetical protein	31/66	RPI25056.1
Kcn25	341	DrrA, ATP-binding protein	59/91	STX12803.1
Kcn26	262	Iga9, ABC transportor	51/99	BAX64250.1
Kcn27	406	PldB, cytochrome P450	45/97	BAH02272.1
Kcn28	396	OleD, glycosyltransferase	54/99	CUW31969.1
Kcn29	186	OrfB4, putative YbaK/prolyl-tRNAsynthetase	52/86	CDP39163.1
Kcn30	272	CitB, LuxR family transcriptional regulator	41/90	ATL86029.1
Kcn31	249	Orf13, thioesterase TEII family	55/100	AAS79476.1
Kcn32	259	Sky43, IclR family transcriptional regulator	57/88	AEA30286.1
Kcn33	213	Cal27, isomerase	54/98	ALG65309.1
Kcn34	70	Cal4, MbtH-like protein	72/97	ALG65332.1
Kcn35	347	IspA, putative polyprenyl diphosphate synthase	52/100	BAG20120.1
Kcn36	717	ActII, membrane protein	53/98	AKJ15297.1
Kcn37	520	SmcA12, methylmalonyl-CoA carboxyltransferase	87/99	ALT05954.1
Kcn38	642	OmpR, putative AfsR-like transcriptional regulator	47/96	AEA30287.1
Kcn39	49	Unknown function		
Kcn40	142	EchC, limonene-1,2-epoxide hydrolase	63/92	AHN91926.1
Kcn41	331	EchB, NAD-dependent epimerase/dehydratase	79/92	AHN91925.1
Kcn42	919	EchA, peptide synthetase	67/99	AHN91924.1

[a]Numbers are in amino acids. [b]Given in numbers are NCBI accession numbers.

Table S4. Prediction of A domain substrate specificity.

	Active sites residues according to GrsA numbering										Amino acids	
	235	236	239	278	299	301	322	330	331	517	Predicted	In Kitacinnamycins
A1	D	F	W	N	V	G	M	V	H	-	Thr	Thr
A2	D	L	T	K	V	G	E	V	G	-	Asn	Asn
A3	D	A	Y	F	W	G	V	T	F	-	-	Val/Ile
A4	D	A	W	T	V	A	A	V	C	-	Phe	Phe
A5	D	A	Y	W	W	G	G	T	F	-	Val	Val
A6	D	I	L	Q	L	G	V	V	W	-	Gly	Gly
A7	D	I	L	Q	L	G	V	V	W	-	Gly	Gly
A8	D	A	Y	W	W	G	G	T	T	-	Val	Val
A9	D	A	L	L	V	G	A	V	V	-	Leu	Leu

The prediction of the substrate specificity was based on NRPS Predictor2.[13]

Table S5. ^{1}H NMR (600 MHz) and ^{13}C NMR (150 MHz) data of **1** in DMSO-d_6.

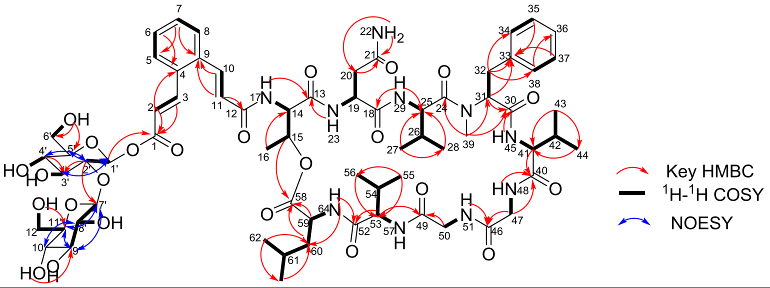

No.	δ_C	δ_H (mult, J in Hz)	No.	δ_C	δ_H (mult, J in Hz)
cinnamic acid			25	54.5	3.65, m
1	164.6		26	29.0	1.99, m
2	121.5	6.56, d (15.7)	27	20.1	0.17, d (6.5)
3	142.5	8.34, d (15.7)	28	18.3	0.54, d (6.5)
4	134.0		29	NH	7.91, d (10.0)
5	129.9	7.38, m	4-N-Me-Phe		
6	127.9	7.82, m	30	170.5	
7	127.8	7.72, m	31	61.6	4.77, m
8	129.3	7.38, m	32	34.2	2.71, m; 3.30, m
9	134.9		33	138.1	
10	134.6	7.79, d (15.5)	34	129.8	7.22, d (7.5)
11	125.7	6.75, d (15.5)	35	129.1	7.33, t (7.5)
12	169.3		36	127.0	7.21, t (7.5)
Glucose			37	129.1	7.34, t (7.5)
1'	93.3	5.64, d (7.9)	38	129.8	7.22, d (7.5)
2'	83.0	3.46, t (7.9)	39 N-Me	30.9	2.60, s
3'	76.0	3.51, m	5-Val		
4'	69.5	3.19, m	40	171.8	
5'	78.3	3.33, m	41	60.3	3.75, m
6'	60.9	3.47, m; 3.69, m	42	29.3	1.80, m
	3' -OH	5.52, d (2.9)	43	19.4	0.81, d (6.7)
	4' -OH	5.22, d (3.3)	44	20.0	0.88, d (6.7)
	6' -OH	4.70, t (6.0)	45	NH	8.40, d (5.9)
7'	105.4	4.38, d (7.8)	6-Gly		
8'	75.1	2.98, m	46	168.7	
9'	76.5	3.14, m	47	42.5	3.43, m; 4.11,m
10'	69.7	3.15, m	48	NH	8.58, t (6.0)
11'	77.2	3.01, m	7-Gly		
12'	60.8	3.35, m; 3.47, m	49	168.6	
	8' -OH	5.23, d (2.0)	50	41.1	3.71, dd (10.6, 6.0)
	9' -OH	4.88, d (4.3)			4.22, m
	11' -OH	4.95, d (4.1)	51	NH	7.88, t (6.0)
	12' -OH	4.32, t (6.0)	8-Val		
1-Thr			52	172.2	
13	168.9		53	56.4	4.39,m
14	60.3	4.21, m	54	32.9	1.98, m
15	69.4	5.07, m	55	17.7	0.46, d (6.7)
16	14.0	1.13, d (6.6)	56	20.2	0.70, d (6.7)
17	NH	8.95, brs	57	NH	6.42, brs
2-Asn			9-Leu		
18	171.1		58	171.9	
19	50.0	4.78, m	59	51.7	4.10, m
20	36.5	1.64, m; 2.70, m	60	39.2	1.40, m; 1.61, m
21	171.5		61	24.4	1.66, m
22	NH_2	6.78, brs; 7.28, brs	62	21.0	0.79, d (6.5)
23	NH	8.22, d (9.0)	63	23.3	0.85, d (6.5)
3-Val			64	NH	8.45, d (6.9)
24	170.7				

Table S6. ^{1}H NMR (600 MHz) and ^{13}C NMR (150 MHz) data of **2** in DMSO-d_6.

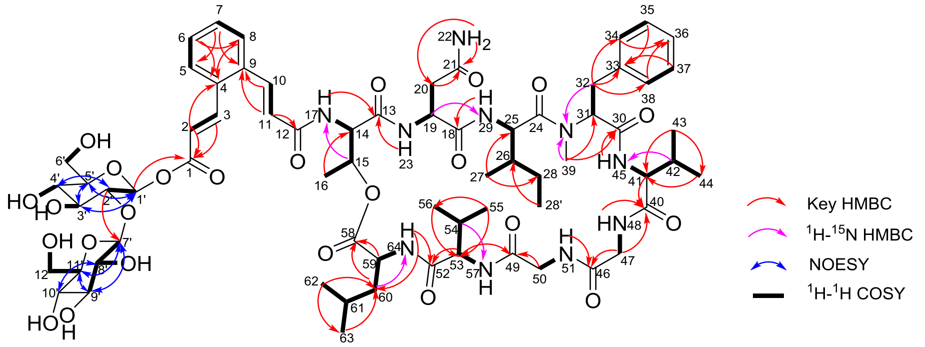

No.	δ_C	δ_H (mult, *J* in Hz)	No.	δ_C	δ_H (mult, *J* in Hz)
cinnamic acid			25	52.3	3.74, m
1	164.1		26	33.9	1.88, m
2	121.1	6.54, d (15.7)	27	15.3	0.12, d (6.4)
3	142.1	8.39, d (15.7)	28	23.0	1.20, m
4	133.6		28'	10.1	0.59, t (7.3)
5	129.9	7.38, m	29	NH	7.89, d (10.2)
6	127.3	7.82, m	4-N-Me-Me		
7	128.2	7.70, m	30	169.9	
8	129.4	7.38, m	31	61.1	4.87, m
9	134.3		32	33.6	2.70, m; 3.30, m
10	134.2	7.81, d (15.5)	33	137.7	
11	125.0	6.75, d (15.5)	34	129.3	7.22, d (7.5)
12	168.7		35	128.5	7.32, t (7.5)
Glucose			36	126.3	7.21, t (7.5)
1'	92.8	5.64, d (8.0)	37	128.5	7.32, t (7.5)
2'	82.5	3.44, t (8.0)	38	129.3	7.22, d (7.5)
3'	75.5	3.51, m	39 N-Me	30.3	2.60, s
4'	69.0	3.15, m	5-Val		
5'	77.8	3.32, m	40	171.3	
6'	60.4	3.47, m; 3.69, m	41	59.7	3.75, m
	3' -OH	5.53, d (2.6)	42	28.8	1.80, m
	4' -OH	5.22, d (5.6)	43	18.9	0.81, d(6.7)
	6' -OH	4.70, t (5.9)	44	19.5	0.88, d (6.7)
7'	104.9	4.37, d (7.7)	45	NH	8.38, d (5.9)
8'	74.5	2.97, t (7.7)	6-Gly		
9'	76.0	3.14, m	46	168.2	
10'	69.1	3.15, m	47	42.0	3.43, m; 4.12, m
11'	76.6	3.00, m	48	NH	8.56, t (6.0)
12'	60.2	3.35, m; 3.47, m	7-Gly		
	8' -OH	5.24, d (2.0)	49	168.1	
	9' -OH	4.86, d (3.2)	50	40.6	3.71, dd (10.6, 6.0)
	10' -OH	4.95, d (2.9)			4.21, m
	12' -OH	4.31, t (6.0)	51	NH	7.86, t (6.0)
1-Thr			8-Val		
13	168.3		52	171.6	
14	59.8	4.20,m	53	55.9	4.38, m
15	68.9	5.07, m	54	32.3	1.97, m
16	13.4	1.12, d (6.5)	55	17.1	0.46, d (6.7)
17	NH	8.93, brs	56	19.7	0.69, d (6.7)
2-Asn			57	NH	6.40, brs
18	170.3		9-Leu		
19	49.4	4.79, m	58	171.4	
20	36.1	1.62, m; 2.71, m	59	51.1	4.10, m
21	171.0		60	38.7	1.40, m; 1.60, m
22	NH_2	6.77, brs; 7.28, brs	61	23.9	1.65, m
23	NH	8.20, d (9.0)	62	20.4	0.79, d (6.5)
3- Ile			63	22.8	0.85, d (6.5)
24	170.2		64	NH	8.44, d (7.0)

Table S7. ^{1}H NMR (600 MHz) and ^{13}C NMR (150 MHz) data of **3** in DMSO-d_6.

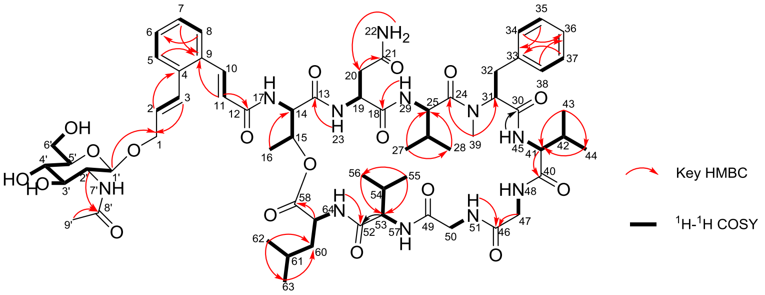

No.	δ_C	δ_H (mult, J in Hz)	No.	δ_C	δ_H (mult, J in Hz)
cinnamic acid			4-N-Me-Phe		
1	67.8	4.35, dd (13.7, 6.0)	30	169.0	
		4.45, dd (13.7, 6.0)	31	60.3	4.80, m
2	128.3	6.24, dt (15.6, 6.0)	32	32.9	2.75, m; 3.29, m
3	133.7	7.24, d (15.6)	33	137.0	
4	135.2		34	128.2	7.24, d (7.4)
5	125.4	7.56, d (7.8)	35	127.6	7.31, t (7.4)
6	127.8	7.28, t (7.8)	36	125.6	7.29, t (7.4)
7	126.1	7.22, t (7.8)	37	127.6	7.31, t (7.4)
8	126.2	7.63, d (7.8)	38	128.2	7.24, t (7.4)
9	131.3		39 N-Me	29.9	2.64, s
10	133.9	7.73, d (15.6)	5-Val		
11	122.9	6.70, d (15.6)	40	170.5	
12	168.4		41	59.0	3.80, m
Glucosamine			42	28.0	1.82, m
1'	99.4	4.43, d (8.5)	43	18.1	0.85, d (6.6)
2'	54.7	3.47, m	44	18.7	0.94, d (6.6)
3'	73.5	3.30, m	45	NH	8.43, d (5.7)
4'	76.3	3.09, m	6-Gly		
5'	69.7	3.10, m	46	167.3	
6'	60.1	3.49, m; 3.72, m	47	41.0	3.38, m; 4.10, m
7'	NH	7.72, d (10.3)	48	NH	8.57, t (6.1)
8'	168.3		7-Gly		
9'	22.2	1.79, s	49	167.2	
	3'-OH	4.93, d (3.9)	50	39.7	3.72, m
	4'-OH	5.02, brs			4.22, dd (16.5, 6.9)
	6'-OH	4.60, m	51	NH	7.81, t (6.0)
1-Thr			8-Val		
13	167.6		52	170.8	
14	59.3	4.15, m	53	54.8	4.38, m
15	68.1	5.07, m	54	31.5	1.88, m
16	12.6	1.12, d (6.5)	55	15.7	0.24, d (6.6)
17	NH	8.92, brs	56	18.8	0.61, d (6.6)
2-Asn			57	NH	6.28, brs
18	169.9		9-Leu		
19	48.6	4.79, m	58	170.4	
20	35.2	1.62, m; 2.71, m	59	50.3	4.10, m
21	170.1		60	37.9	1.40, m; 1.62, m
22	NH_2	6.75,brs; 7.26, brs	61	23.1	1.67, m
23	NH	8.28, d (9.1)	62	19.6	0.79, d (6.4)
3-Val			63	22.0	0.86, d (6.4)
24	169.5		64	NH	8.52, d (7.1)
25	53.3	3.69, m			
26	27.8	2.01, m			
27	18.5	0.15, d (6.5)			
28	17.0	0.55, d (6.5)			
29	NH	7.97, d (10.1)			

Table S8. ^{1}H NMR (600 MHz) and ^{13}C NMR (150 MHz) data of **4** in DMSO-d_6.

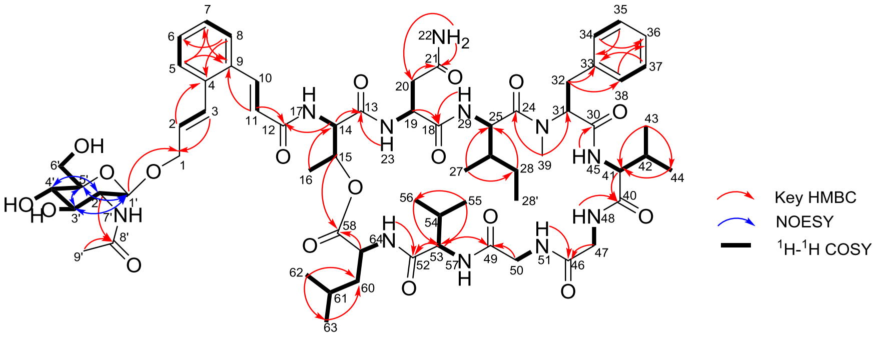

No.	δ_C	δ_H (mult, *J* in Hz)	No.	δ_C	δ_H (mult, *J* in Hz)
cinnamic acid			4-N-Me-Phe		
1	67.5	4.35, dd (13.6,6.0)	30	168.8	
		4.45, dd (13.6, 6.0)	31	59.9	4.89, d (12.3)
2	128.0	6.24, dt (15.6, 6.0)	32	32.6	2.75, m; 3.29, m
3	133.7	7.28, d (15.6)	33	136.7	
4	134.9		34	128.2	7.24, d (7.4)
5	125.2	7.56, d (7.8)	35	127.4	7.30, t (7.4)
6	127.5	7.29, t (7.8)	36	125.4	7.29, t (7.4)
7	126.3	7.22, t (7.8)	37	127.4	7.30, t (7.4)
8	125.9	7.63, d (7.8)	38	128.2	7.24, d (7.4)
9	131.2		39/N-Me	29.7	2.64, s
10	133.8	7.75, d (15.6)	5-Val		
11	122.6	6.69, d (15.6)	40	170.1	
12	168.2		41	58.8	3.80, m
Glucosamide			42	27.7	1.82, m
1'	99.2	4.42, d (8.6)	43	17.9	0.84, d (6.4)
2'	54.4	3.47, m	44	18.5	0.94, d (6.4)
3'	73.3	3.30, m	45	NH	8.41, d (6.0)
4'	76.0	3.09, m	6-Gly		
5'	69.5	3.10, m	46	167.1	
6'	60.0	3.49, m; 3.71, m	47	40.8	3.38, m; 4.12, m
7'	NH	7.73, d (9.6)	48	NH	8.54, t (6.1)
8'	168.0		7-Gly		
9'	22.0	1.79, s	49	167.0	
	3'-OH	4.93, d (3.9)	50	39.5	3.71, m
	4'-OH	5.02, brs			4.21, dd (16.3, 6.9)
	6'-OH	4.60, m	51	NH	7.80, t (6.1)
1-Thr			8-Val		
13	167.3		52	170.7	
14	59.0	4.14, m	53	54.6	4.36, m
15	67.9	5.07, m	54	31.3	1.87, m
16	12.3	1.11, d (6.3)	55	15.5	0.24, d (6.2)
17	NH	8.90, brs	56	18.6	0.60, d (6.2)
2-Asn			57	NH	6.26, brs
18	169.6		9-Leu		
19	48.4	4.80, m	58	170.4	
20	35.0	1.61, m; 2.72, m	59	50.2	4.10, m
21	170.0		60	37.7	1.40, m; 1.62, m
22	NH_2	6.74, brs; 7.26, brs	61	22.9	1.67, m
23	NH	8.27, d (9.3)	62	19.4	0.79, d (6.3)
3- Ile			63	21.8	0.86, d (6.3)
24	169.3		64	NH	8.51, d (7.1)
25	51.4	3.79, m			
26	32.9	1.88, m			
27	14.0	0.09, d (6.1)			
28	21.9	1.22, m			
28'	9.0	0.61, t (7.2)			
29	NH	7.93, d (10.0)			

Table S9. ^{1}H NMR (600 MHz) and ^{13}C NMR (150 MHz) data of **5** in DMSO-d_6.

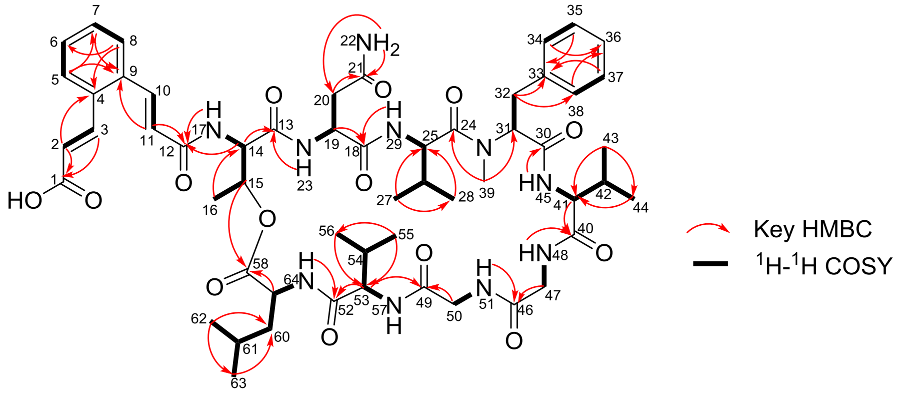

No.	δ_C	δ_H (mult, *J* in Hz)	No.	δ_C	δ_H (mult, *J* in Hz)
cinnamic acid			33	137. 9	
1	167.3		34	129.4	7.21, d (7.5)
2	122.2	6.40, d (15.7)	35	128.6	7.30, t (7.5)
3	140.6	8.11, d (15.7)	36	126.7	7.22, t (7.5)
4	133.7		37	128.6	7.30, t (7.5)
5	127.5	7.78, d (7.3)	38	129.4	7.21, d (7.5)
6	129.8	7.36, m	39 N-Me	30.4	2.60, s
7	129.5	7.36, m	5-Val		
8	127.3	7.70, m	40	171.4	
9	134.4		41	60.0	3.76, m
10	134.2	7.75, d (15.5)	42	29.0	1.81, m
11	125.4	6.74, d (15.5)	43	19.0	0.82, d (6.7)
12	169.0		44	19.6	0.89, d (6.7)
1-Thr			45	NH	8.38, d (6.0)
13	168.6		6-Gly		
14	59.9	4.21, m	46	168.5	
15	69.2	5.08, m	47	42.0	3.44, m; 4.10, m
16	13.6	1.13, d (6.5)	48	NH	8.53, t (5.7)
17	NH	8.93, brs	7-Gly		
2-Asn			49	168.4	
18	170.8		50	40.8	3.70, m; 4.23, m
19	49.7	4.79, m	51	NH	7.89, t (5.7)
20	36.2	1.67, m	8-Val		
		2.70, dd (15.8, 3.5)	52	171.9	
21	171.2		53	56.1	4.38, dd (8.0, 3.9)
22	NH_2	6.78, brs; 7.29, brs	54	32.5	1.94, m
23	NH	8.26, d (9.0)	55	17.2	0.42, d (6.6)
3-Val			56	19.8	0.69, d (6.6)
24	170.2		57	NH	6.42, brs
25	54.1	3.73, m	9-Leu		
26	28.8	1.93, m	58	171.6	
27	19.4	0.15, d (6.5)	59	51.3	4.11, m
28	17.9	0.56, d (6.5)	60	39.0	1.40, m; 1.60, m
29	NH	7.88, d (10.0)	61	24.1	1.66, m
4-N-Me-Phe			62	20.6	0.79, d (6.4)
30	170.1		63	23.0	0.86, d (6.4)
31	61.1	4.78, dd (11.5, 3.1)	64	NH	8.44, d (7.1)
32	33.9	2.75, m; 3.30, m			

Table S10. ^{1}H NMR (600 MHz) and ^{13}C NMR (150 MHz) data of **6** in DMSO-d_6.

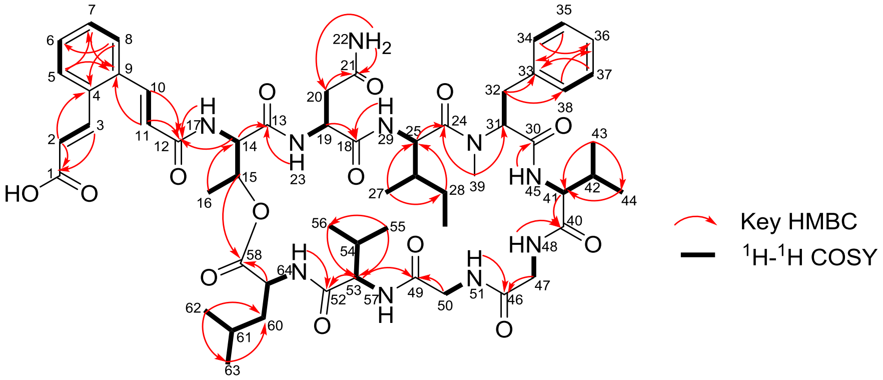

No.	δ_C	δ_H (mult, *J* in Hz)	No.	δ_C	δ_H (mult, *J* in Hz)
cinnamic acid			32	33.6	2.74, m; 3.30, m
1	167.5		33	137.6	
2	123.2	6.39, d (15.7)	34	129.1	7.20, d (7.6)
3	139.6	8.09, d (15.7)	35	128.3	7.29, t (7.6)
4	133.7		36	126.3	7.21, t (7.6)
5	127.2	7.77 , m	37	128.3	7.29, t (7.6)
6	129.2	7.35, m	38	129.1	7.20, d (7.6)
7	128.0	7.35, m	39 N-Me	30.0	2.59, s
8	126.8	7.68, m	5-Val		
9	134.0		40	171.1	
10	134.2	7.76, d (15.5)	41	59.5	3.76, m
11	124.9	6.73, d (15.5)	42	28.7	1.80, m
12	168.5		43	18.7	0.81, d (6.7)
1-Thr			44	19.3	0.88, d (6.7)
13	168.2		45	NH	8.36, d (5.9)
14	59.4	4.22, m	6-Gly		
15	68.9	5.08, m	46	168.1	
16	13.3	1.12, d (6.5)	47	41.7	3.44, m; 4.10, m
17	NH	8.88, brs	48	NH	8.53, t (5.8)
2-Asn			7-Gly		
18	170.3		49	168.0	
19	49.3	4.79, m	50	40.5	3.70, m; 4.20, m
20	36.0	1.67, m	51	NH	7.88, t (5.6)
		2.70, dd (15.8, 3.5)	8-Val		
21	170.9		52	171.4	
22	NH_2	6.77, brs; 7.28, brs	53	55.9	4.36, dd (7.8, 3.9)
23	NH	8.24, d (9.0)	54	32.1	1.95, m
3- Ile			55	16.9	0.44, d (6.6)
24	169.9		56	19.5	0.69, d (6.6)
25	52.1	3.84, m	57	NH	6.44, brs
26	34.0	1.79, m	9-Leu		
27	14.8	0.10, d (6.4)	58	171.3	
28	22.8	1.23, m	59	51.0	4.11, m
28'	10.0	0.60, t (7.4)	60	38.6	1.40, m; 1.60, m
29		7.85, d (10.1)	61	23.8	1.66, m
4-N-Me-Phe			62	20.3	0.79, d (6.5)
30	169.7		63	22.6	0.85, d (6.5)
31	60.8	4.87, dd (11.3, 2.8)	64	NH	8.42, d (7.0)

Table S11. ¹H NMR (600 MHz) and ¹³C NMR (150 MHz) data of **7** in DMSO-d_6.

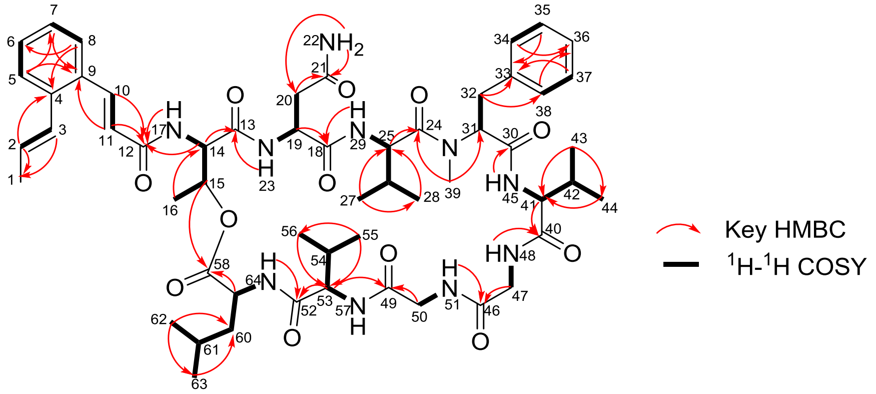

No.	δ_C	δ_H (mult, *J* in Hz)	No.	δ_C	δ_H (mult, *J* in Hz)
cinnamic acid			34	129.2	7.24, d (7.5)
1	18.4	1.95, d (6.0)	35	128.4	7.30, t (7.5)
2	128.1	6.19, dd (15.4, 6.7)	36	126.4	7.23, t (7.5)
3	126.5	7.14, d (15.4)	37	128.4	7.30, t (7.5)
4	136.9		38	129.2	7.24, d (7.5)
5	127.1	7.60, d (7.8)	39 N-Me	30.4	2.69, s
6	128.0	7.16, m	5-Val		
7	128.9	7.26, m	40	171.2	
8	125.8	7.46, d (7.8)	41	59.8	3.80, m
9	131.6		42	28.7	1.83, m
10	135.3	7.75, d (15.6)	43	18.9	0.85, d (6.6)
11	123.3	6.69, d (15.6)	44	19.52	0.93, d (6.6)
12	169.4		45	NH	8.43, d (6.2)
1-Thr			6-Gly		
13	168.4		46	168.1	
14	60.1	4.16, m	47	41.8	3.38, m; 4.13, m
15	68.9	5.07, m	48	NH	8.56, t (6.0)
16	13.3	1.13, d (6.5)	7-Gly		
17	NH	8.91, brs	49	168.0	
2-Asn			50	40.5	3.72, m
18	170.8				4.21, dd (16.3, 6.9)
19	49.5	4.80, m	51	NH	7.80, t (5.8)
20	35.9	1.63, m; 2.72, m	8-Val		
21	170.92		52	171.7	
22	NH_2	6.75, brs; 7.27, brs	53	55.5	4.39, dd (8.2, 3.3)
23	NH	8.30, d (9.2)	54	32.3	1.88, m
3-Val			55	16.5	0.25, d (6.7)
24	170.2		56	19.54	0.61, d (6.7)
25	54.1	3.71, m	57	NH	6.29, d (8.2)
26	28.5	2.02, m	9-Leu		
27	19.3	0.15, d (6.6)	58	171.4	
28	17.7	0.56, d (6.6)	59	51.2	4.11, m
29	NH	7.98, d (10.1)	60	38.7	1.41, m; 1.62, m
4-N-Me-Phe			61	23.9	1.68, m
30	169.9		62	20.4	0.79, d (6.5)
31	60.8	4.82, dd (11.8, 3.2)	63	22.8	0.86, d (6.5)
32	33.6	2.77, m; 3.29, m	64	NH	8.50, d (6.9)
33	137. 6				

Table S12. ^{1}H NMR (600 MHz) and ^{13}C NMR (150 MHz) data of **8** in DMSO-d_6.

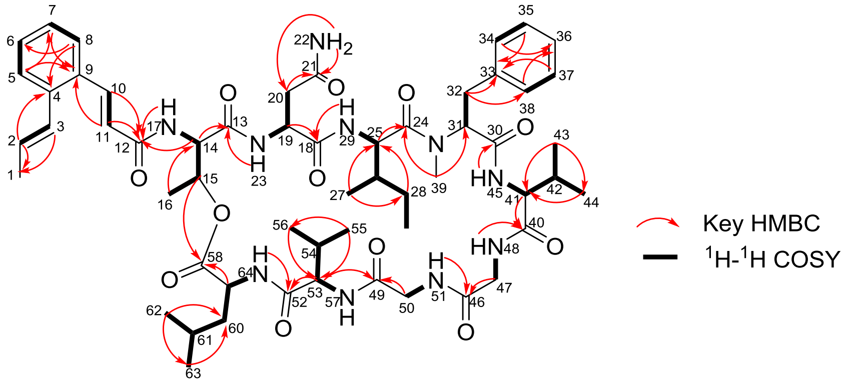

No.	δ_C	δ_H (mult, J in Hz)	No.	δ_C	δ_H (mult, J in Hz)
cinnamic acid			33	137.7	
1	18.4	1.95, dd (6.6, 1.2)	34	129.2	7.24, d (7.5)
2	128.01	6.19, dd (15.4, 6.7)	35	128.4	7.30, t (7.5)
3	126.5	7.17, d (15.4)	36	126.4	7.23, t (7.5)
4	137.0		37	128.4	7.30, t (7.5)
5	127.1	7.60, d (7.8)	38	129.2	7.24, d (7.5)
6	128.02	7.16, m	39/N-Me	30.4	2.69, s
7	128.9	7.26, m	5-Val		
8	125.8	7.47, d (7.8)	40	171.2	
9	131.6		41	59.8	3.80, m
10	135.4	7.77, d (15.7)	42	28.7	1.84, m
11	123.2	6.68, d (15.7)	43	18.9	0.85, d (6.6)
12	169.3		44	19.5	0.93, d (6.6)
1-Thr			45	NH	8.44, d (6.3)
13	168.4		6-Gly		
14	60.0	4.15, m	46	168.1	
15	68.9	5.08, m	47	41.8	3.39, m; 4.13, m
16	13.3	1.12, d (6.6)	48	NH	8.58, t (5.8)
17	NH	8.89, brs	7-Gly		
2-Asn			49	168.0	
18	170.7		50	40.5	3.72, m
19	49.4	4.82, m			4.21, dd (16.4, 4.9)
20	36.0	1.62, m; 2.73, m	51	NH	7.80, t (6.1)
21	171.0		8-Val		
22	NH_2	6.75, brs; 7.28, brs	52	171.7	
23	NH	8.29, d (9.2)	53	55.5	4.38, dd (8.3, 3.6)
3- Ile			54	32.3	1.88, m
24	170.2		55	16.5	0.26, d (6.7)
25	52.4	3.81, m	56	19.5	0.62, d (6.7)
26	33.9	1.89, m	57	NH	6.28, d (8.3)
27	15.0	0.09, d (6.5)	9-Leu		
28	22.9	1.24, m	58	171.4	
28'	10.0	0.62, t (7.4)	59	51.2	4.11, m
29		7.96, d (10.2)	60	38.7	1.41, m; 1.62, m
4-N-Me-Phe			61	23.9	1.67, m
30	169.9		62	20.4	0.79, d (6.5)
31	60.8	4.92, dd (11.5, 2.8)	63	22.8	0.86, d (6.5)
32	33.6	2.77, m; 3.30, m	64	NH	8.50, d (7.0)

Table S13. ^{1}H NMR (600 MHz) and ^{13}C NMR (150 MHz) data of **9** in DMSO-d_6.

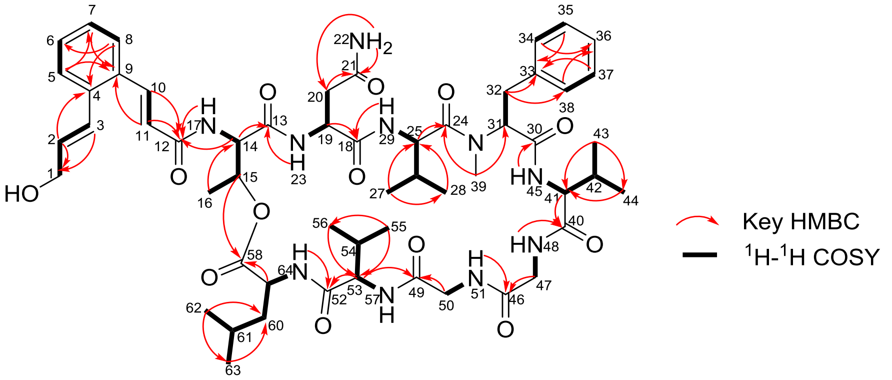

No.	δ_C	δ_H (mult, J in Hz)	No.	δ_C	δ_H (mult, J in Hz)
cinnamic acid			33	137. 7	
1	62.1	4.23, d (5.8)	34	129.4	7.24, d (7.5)
2	133.4	6.27, dt (15.7, 5.8)	35	128.5	7.32, t (7.5)
3	126.2	7.21, d (15.7)	36	126.6	7.22, t (7.5)
4	136.3		37	128.5	7.32, t (7.5)
5	127.0	7.64, d (7.8)	38	129.4	7.24, d (7.5)
6	129.1	7.20, t (7.8)	39 N-Me	30.7	2.65, s
7	127.1	7.28, t (7.8)	5-Val		
8	126.1	7.51, d (7.8)	40	171.3	
9	132.2		41	60.0	3.80, m
10	135.0	7.76, d (15.6)	42	28.8	1.83, m
11	123.5	6.70, d (15.6)	43	19.0	0.84, d (6.5)
12	169.5		44	19.6	0.93, d (6.5)
1-Thr			45	NH	8.42, d (6.2)
13	168.5		6-Gly		
14	60.2	4.17, m	46	168.2	
15	69.0	5.07, m	47	41.9	3.40, m; 4.13, m
16	13.5	1.13, d (6.5)	48	NH	8.54, t (5.8)
17	NH	8.91, brs	7-Gly		
2-Asn			49	168.0	
18	171.0		50	40.6	3.70, m; 4.25, m
19	49.6	4.81, m	51	NH	7.84, t (5.8)
20	36.1	1.63, m	8-Val		
		2.71, dd (15.2, 3.2)	52	171.8	
21	171.0		53	55.7	4.39, dd(8.1, 3.2)
22	NH_2	6.76, brs; 7.27, brs	54	32.4	1.90, m
23	NH	8.29, d (9.1)	55	16.6	0.27, d (6.6)
3-Val			56	19.7	0.62, d (6.6)
24	170.0		57	NH	6.29, brs
25	54.3	3.71, m	9-Leu		
26	28.6	2.01, m	58	171.5	
27	19.4	0.15, d (6.5)	59	51.3	4.11, m
28	17.8	0.56, d (6.5)	60	38.8	1.41, m; 1.62, m
29	NH	7.98, d (10.1)	61	24.0	1.67, m
4-N-Me-Phe			62	20.5	0.79, d (6.3)
30	169.9		63	22.9	0.86, d (6.3)
31	61.0	4.82, dd (12.0, 3.1)	64	NH	8.49, d (6.9)
32	33.7	2.77, m; 3.29, m			

Table S14. ^{1}H NMR (600 MHz) and ^{13}C NMR (150 MHz) data of **10** in DMSO-d_6.

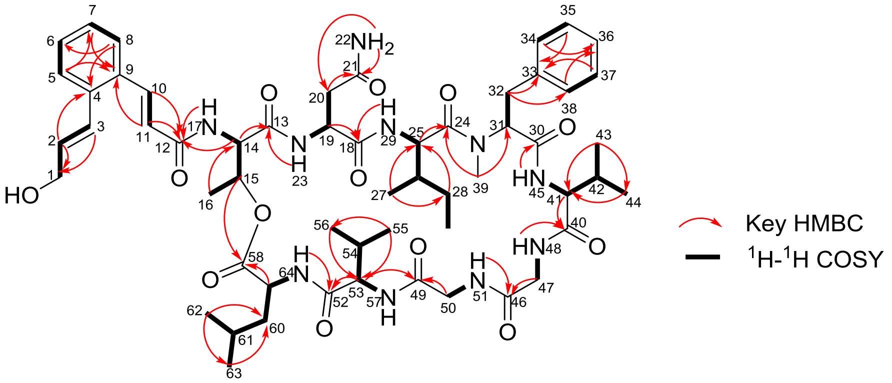

No.	δ_C	δ_H (mult, J in Hz)	No.	δ_C	δ_H (mult, J in Hz)
cinnamic acid			32	33.6	2.76, m; 3.30, m
1	61.9	4.22, d (5.6)	33	137.7	
2	133.2	6.26, dt (15.6, 5.6)	34	129.2	7.24, d (7.5)
3	126.1	7.25, d (15.6)	35	128.4	7.30, t (7.5)
4	136.3		36	126.4	7.23, t (7.5)
5	126.8	7.63, d (7.8)	37	128.4	7.30, t (7.5)
6	129.0	7.20, t (7.8)	38	129.2	7.24, d (7.5)
7	127.0	7.28, t (7.8)	39/N-Me	30.6	2.65, s
8	126.0	7.51, d (7.8)	5-Val		
9	132.0		40	171.1	
10	134.9	7.78, d (15.6)	41	59.8	3.80, m
11	123.4	6.69, d (15.6)	42	28.7	1.83, m
12	169.3		43	18.9	0.84, d (6.6)
1-Thr			44	19.5	0.93, d (6.6)
13	168.3		45	NH	8.42, d (6.4)
14	60.0	4.16, m	6-Gly		
15	68.9	5.07, m	46	168.1	
16	13.3	1.12, d (6.6)	47	41.7	3.39, m; 4.13, m
17	NH	8.89, brs	48	NH	8.54, t (6.0)
2-Asn			7-Gly		
18	170.7		49	168.0	
19	49.4	4.81, m	50	40.5	3.70, m; 4.24, m
20	36.0	1.61, m	51	NH	7.83, t (5.9)
		2.72, dd (15.4, 3.6)	8-Val		
21	171.0		52	171.6	
22	NH_2	6.75, brs; 7.27, brs	53	55.6	4.37, dd (8.3, 3.6)
23	NH	8.28, d (9.2)	54	32.3	1.90, m
3-Ile			55	16.5	0.27, d (6.7)
24	170.4		56	19.6	0.62, d (6.7)
25	52.4	3.81, m	57	NH	6.28, brs
26	33.9	1.88, m	9-Leu		
27	15.0	0.09, d (6.5)	58	171.4	
28	22.9	1.23, m	59	51.1	4.11, m
28'	10.0	0.61, t (7.5)	60	38.7	1.41, m; 1.62, m
29	NH	7.95, d (10.2)	61	23.9	1.67, m
4-N-Me-Phe			62	20.4	0.79, d (6.4)
30	169.9		63	22.8	0.85, d (6.4)
31	60.9	4.91, dd (11.3, 2.8)	64	NH	8.48, d (7.0)

Table S15. ^{1}H NMR (600 MHz) and ^{13}C NMR (150 MHz) data of **11** in DMSO-d_6.

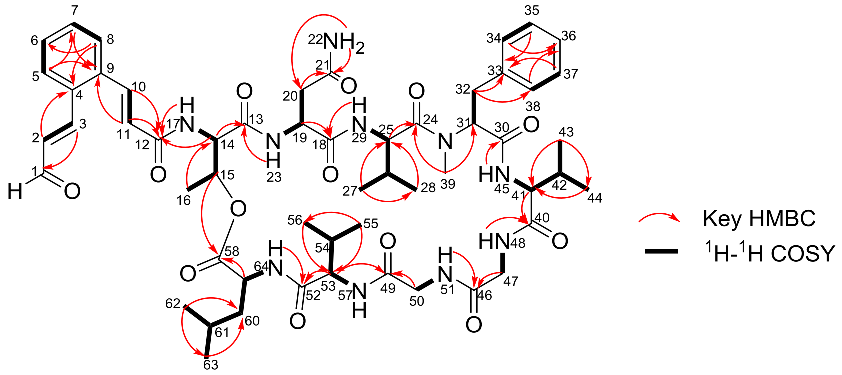

No.	δ_C	δ_H (mult, J in Hz)	No.	δ_C	δ_H (mult, J in Hz)
cinnamic acid			33	137. 8	
1	194.9	9.89, d (7.8)	34	129.4	7.24, d (7.5)
2	130.4	6.82, dd (15.7, 7.8)	35	128.5	7.31, t (7.5)
3	149.9	8.49, d (15.7)	36	126.6	7.23, t (7.5)
4	134.5		37	128.5	7.31, t (7.5)
5	127.3	7.83, d (7.9)	38	129.4	7.24, d (7.5)
6	129.5	7.40, m	39/N-Me	30.9	2.59, s
7	130.9	7.44, m	5-Val		
8	127.7	7.76, d (7.6)	40	171.3	
9	132.9		41	60.0	3.79, m
10	133.9	7.88, d (15.6)	42	28.8	1.83, m
11	125.2	6.77, d (15.6)	43	19.0	0.84, d (6.6)
12	169.4		44	19.7	0.94, d (6.6)
1-Thr			45	NH	8.45, d (6.3)
13	168.5		6-Gly		
14	60.4	4.17, m	46	168.2	
15	69.0	5.08, m	47	41.8	3.41, m; 4.12, m
16	13.5	1.13, d (6.6)	48	NH	8.52, t (5.8)
17	NH	9.03, brs	7-Gly		
2-Asn			49	168.1	
18	171.0		50	40.6	3.67, m; 4.27, m
19	49.7	4.81, m	51	NH	7.89, t (5.7)
20	36.2	1.60, m; 2.71, m	8-Val		
21	171.0		52	171.9	
22	NH_2	6.75, brs; 7.27, brs	53	55.5	4.39, dd (8.0, 3.0)
23	NH	8.26, d (9.4)	54	32.6	1.90, m
3-Val			55	16.3	0.19, d (6.8)
24	170.6		56	19.8	0.58, d (6.8)
25	54.5	3.69, m	57	NH	6.22, d (8.0)
26	28.6	2.09, m	9-Leu		
27	19.3	0.13, d (6.6)	58	171.6	
28	17.9	0.55, d (6.6)	59	51.3	4.11, m
29	NH	7.98, d (10.2)	60	38.9	1.41, m; 1.62, m
4-N-Me-Phe			61	24.0	1.67, m
30	170.3		62	20.5	0.79, d (6.3)
31	61.0	4.87, m	63	23.0	0.86, d (6.3)
32	33.7	2.76, m	64	NH	8.51, d (7.0)
		3.28, d (12.2)			

Table S16. ^{1}H NMR (600 MHz) and ^{13}C NMR (150 MHz) data of **12** in DMSO-d_6.

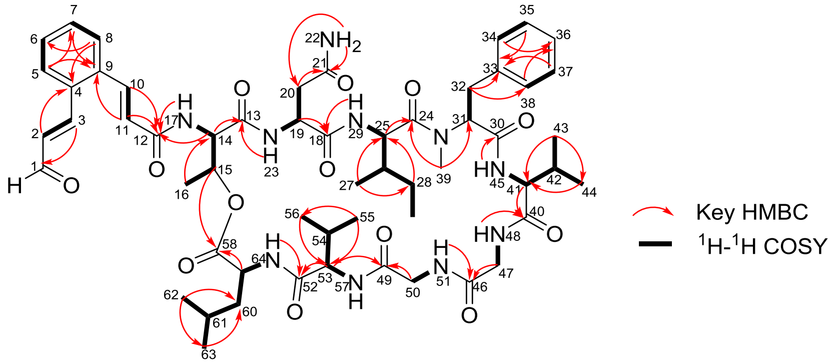

No.	δ_C	δ_H (mult, J in Hz)	No.	δ_C	δ_H (mult, J in Hz)
cinnamic acid			33	137.7	
1	194.7	9.89, d (7.8)	34	129.2	7.24, d (7.6)
2	130.2	6.83, dd (15.6, 7.9)	35	128.4	7.31, t (7.6)
3	149.8	8.54, d (15.6)	36	126.4	7.23, t (7.6)
4	134.3		37	128.4	7.31, t (7.6)
5	127.1	7.84, d (7.8)	38	129.2	7.24, d (7.6)
6	129.3	7.40, m	39 N-Me	30.7	2.59, s
7	130.7	7.45, m	5-Val		
8	127.4	7.77, d (7.8)	40	171.1	
9	132.7		41	59.8	3.80, m
10	133.7	7.91, d (15.5)	42	28.7	1.83, m
11	124.9	6.78, d (15.5)	43	18.9	0.85, d (6.6)
12	169.1		44	19.6	0.95, d (6.6)
1-Thr			45	NH	8.46, d (6.4)
13	168.2		6-Gly		
14	60.2	4.17, m	46	168.0	
15	68.8	5.08, m	47	41.6	3.40, m; 4.14, m
16	13.3	1.13, d (6.5)	48	NH	8.52, t (6.0)
17	NH	9.01, brs	7-Gly		
2-Asn			49	167.9	
18	170.9		50	40.4	3.67, m; 4.27, m
19	49.5	4.83, m	51	NH	7.89, t (5.8)
20	36.1	1.57, m; 2.73, m	8-Val		
21	170.9		52	171.6	
22	NH_2	6.74, brs; 7.28, brs	53	55.4	4.38, dd (8.0, 3.0)
23	NH	8.25, d (9.3)	54	32.4	1.91, m
3- Ile			55	16.1	0.20, d (6.8)
24	170.5		56	19.5	0.59, d (6.8)
25	52.4	3.81, m	57	NH	6.22, d (8.0)
26	33.7	1.98, m	9-Leu		
27	14.9	0.08, d (6.5)	58	171.4	
28	22.9	1.23, m	59	51.2	4.11, m
28'	9.8	0.61, t (7.7)	60	38.7	1.42, m; 1.62, m
29		7.96, d (10.2)	61	23.9	1.68, m
4-N-Me-Phe			62	20.3	0.80, d (6.4)
30	170.1		63	22.8	0.86, d (6.4)
31	60.9	4.97, m	64	NH	8.51, d (7.1)
32	33.5	2.77, m			
		3.29, d (11.8)			

Table S17. Data collection and refinement statistics (molecular replacement) of Kcn28 and Kcn28 in complex with **9**.

	Kcn28	Kcn28-**9** complex
**Data collection**		
Space group	I23	I23
Cell dimensions		
a, *b*, *c* (Å)	242.9, 242.9, 242.9	243.2, 243.2, 243.2
α, β, γ (°)	90, 90, 90	90, 90, 90
Resolution (Å)	99.16-2.50 (2.55-2.50)	39.45-2.24 (2.28-2.24)
R_{merge} (%)	7.5 (91.8)	25.9 (192.1)
I / σ*I*	10.7 (1.6)	8.3 (1.9)
CC1/2 (%)	99.6 (54.5)	99.8 (17.9)
Completeness (%)	99.6 (99.9)	100 (100)
Redundancy	5.7 (5.0)	19.0 (19.8)
**Refinement**		
Resolution (Å)	70.1-2.5 (2.59-2.50)	39.45-2.24 (2.33-2.24)
No. reflections	81367	113311 (11335)
R_{work} / R_{free} (%)	26.88 (35.51) / 28.50 (35.68)	22.57 (37.59) / 24.85 (38.89)
No. atoms	11972	12788
Protein	11805	11910
Ligand/ion	0	312
Water	167	566
B-factors	86.1	56.5
Protein	86.4	56.5
Ligand/ion	0	71.9
Water	60.0	47.8
R.m.s. deviations		
Bond lengths (Å)	0.004	0.004
Bond angles (°)	0.88	1.02
Ramachandran plot (%)		
favored	92.07	93.86
outliers	2.49	1.90

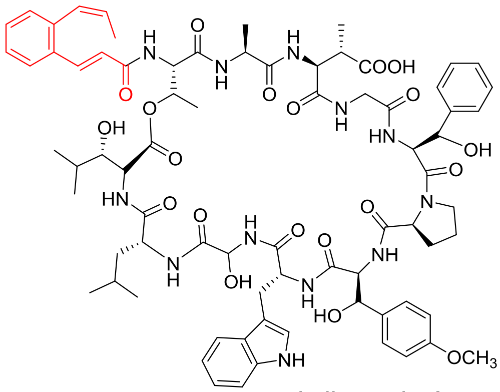

skyllamycin A

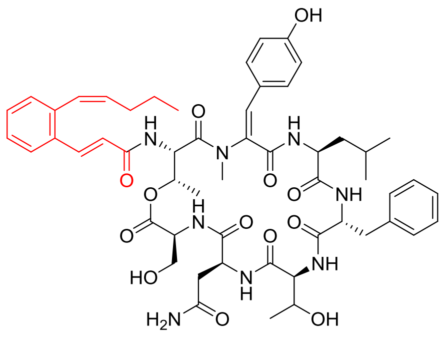

WS9326A

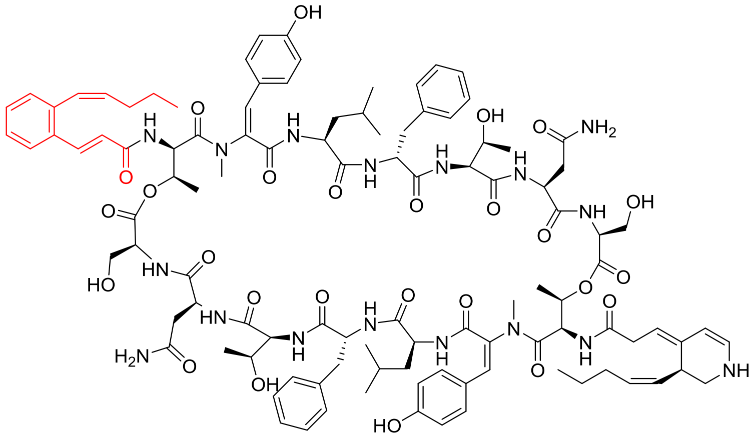

Mohangamide A

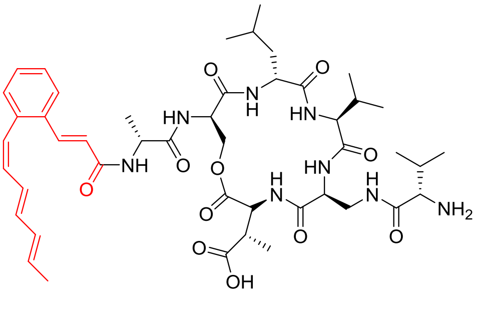

coprisamide A

Figure S1. Structures of known CCNPs including skyllamycin A, WS9326A, mohangamide A, coprisamide A. The substructures highlighted by red color are cinnamoyl containing moiety.

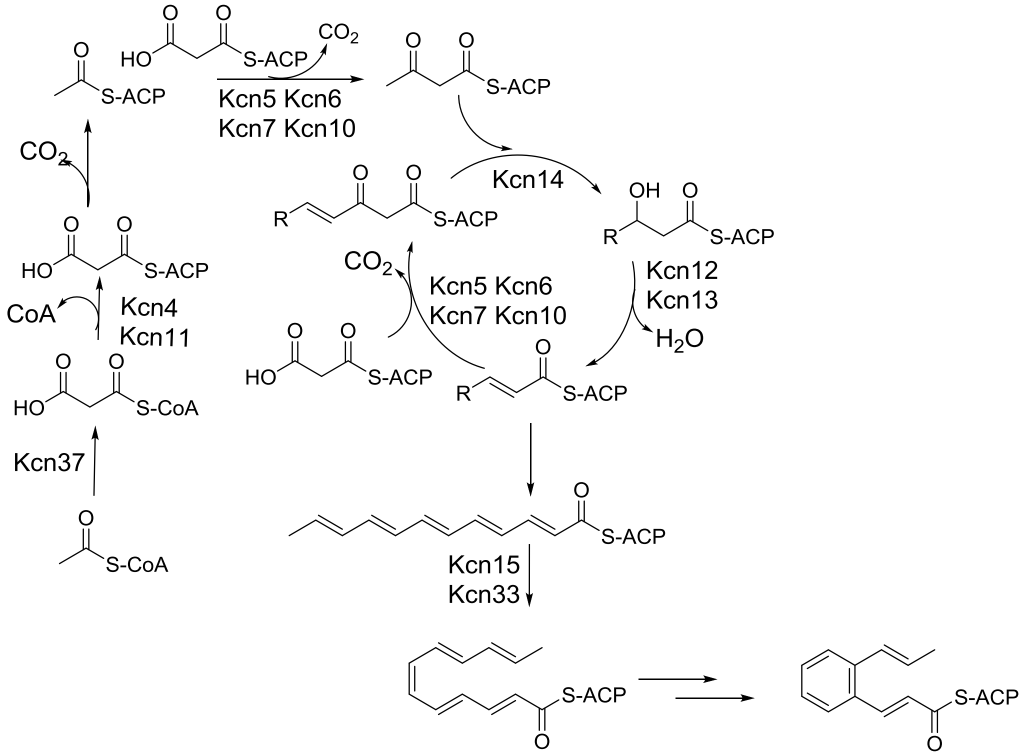

Figure S2. Proposed biosynthetic pathway for N-terminal cinnamyl residue biosynthesis.

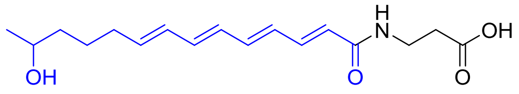

ishigamide

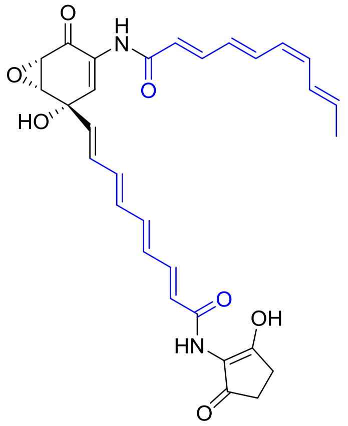

colabomycin A

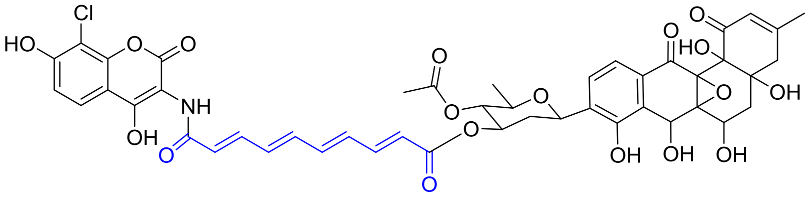

simocyclinone D8

Figure S3. Structures of ishigamide, colabomycin A, and simocyclinone D8. The substructures highlighted by blue color are biosynthesized by type II PKS.

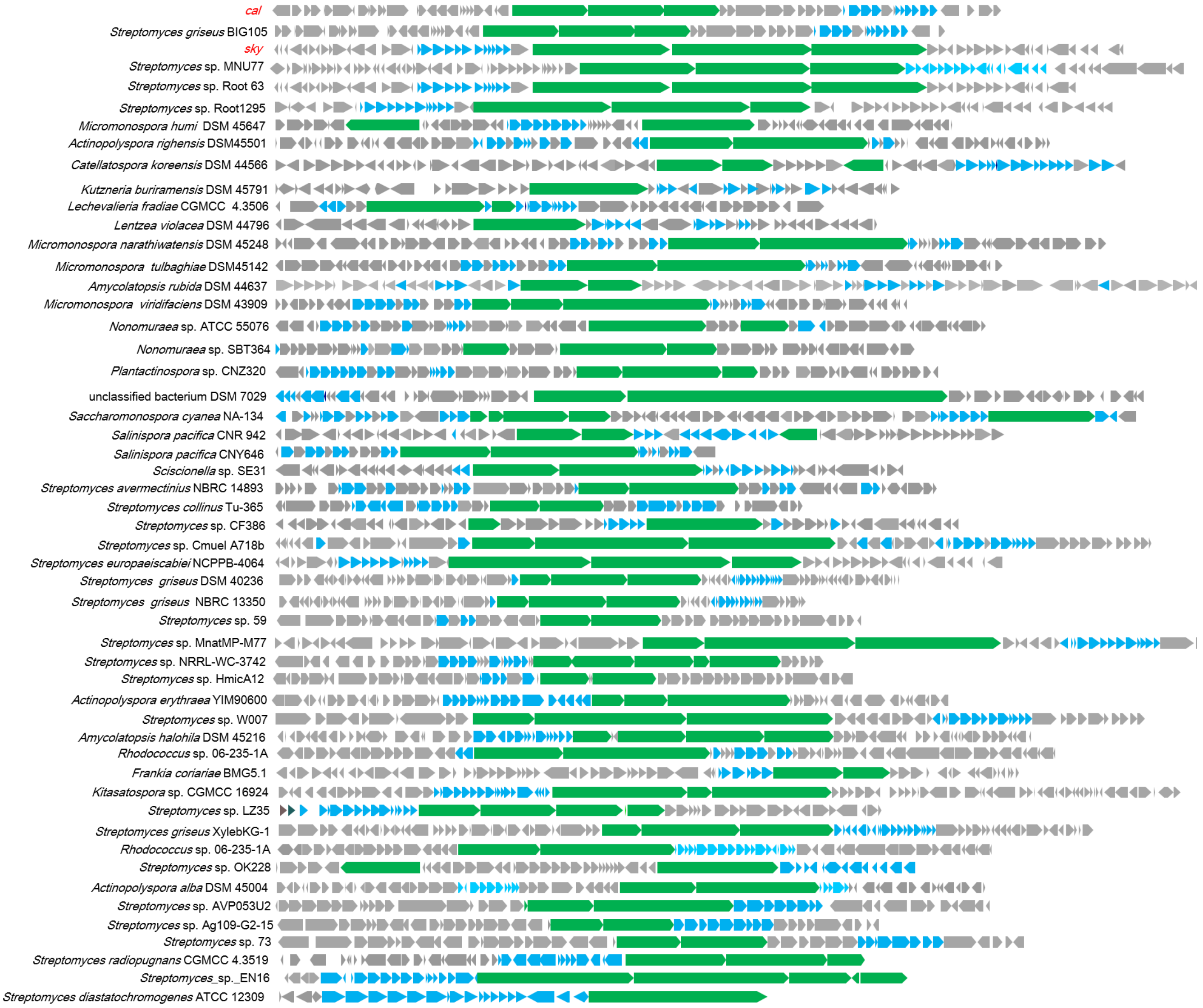

Figure S4. Biosynthetic gene clusters for putative CCNPs. NRPS genes are highlighted in green color, and type II PKS genes are marked in blue color.

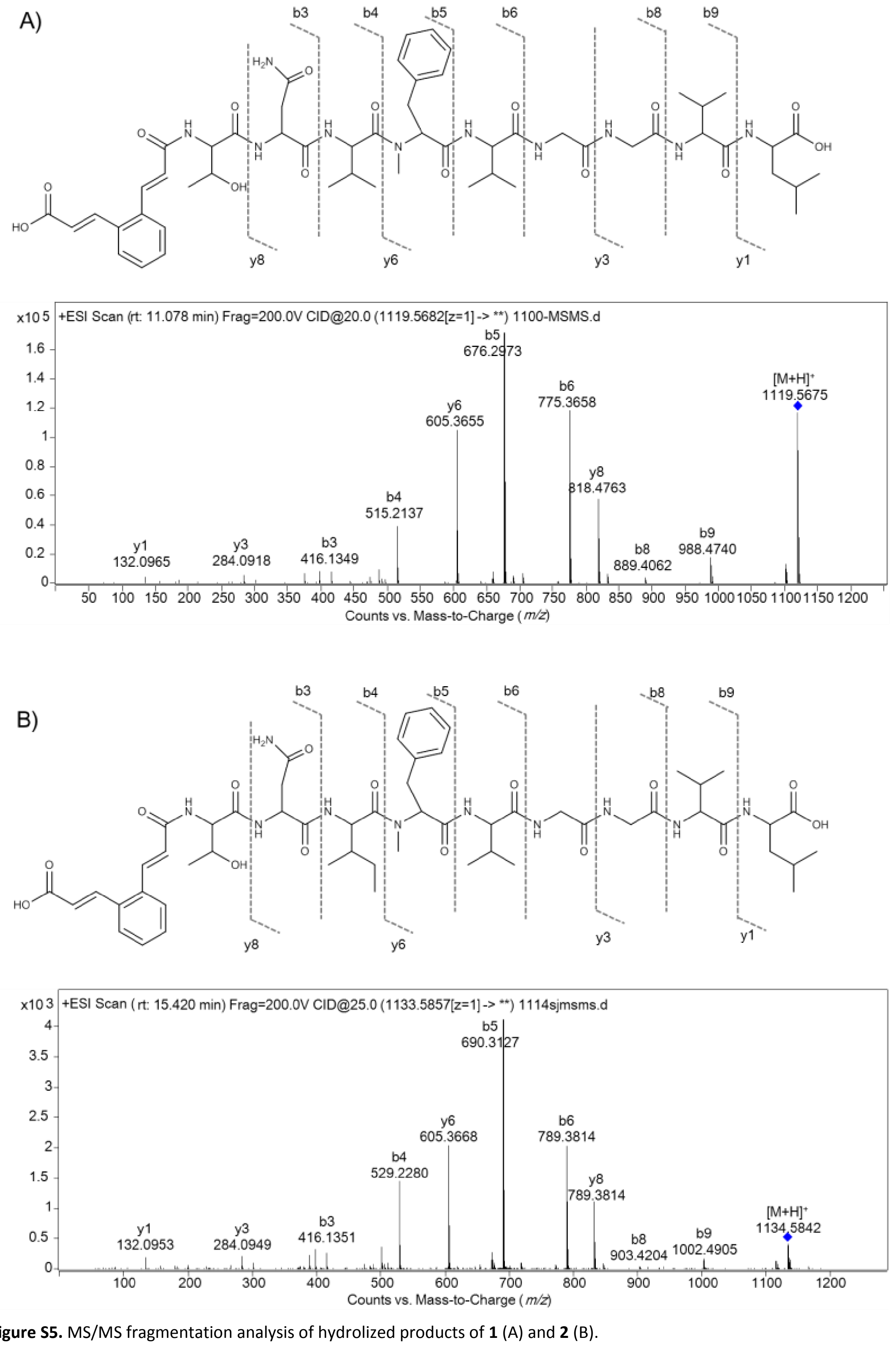

Figure S5. MS/MS fragmentation analysis of hydrolized products of **1** (A) and **2** (B).

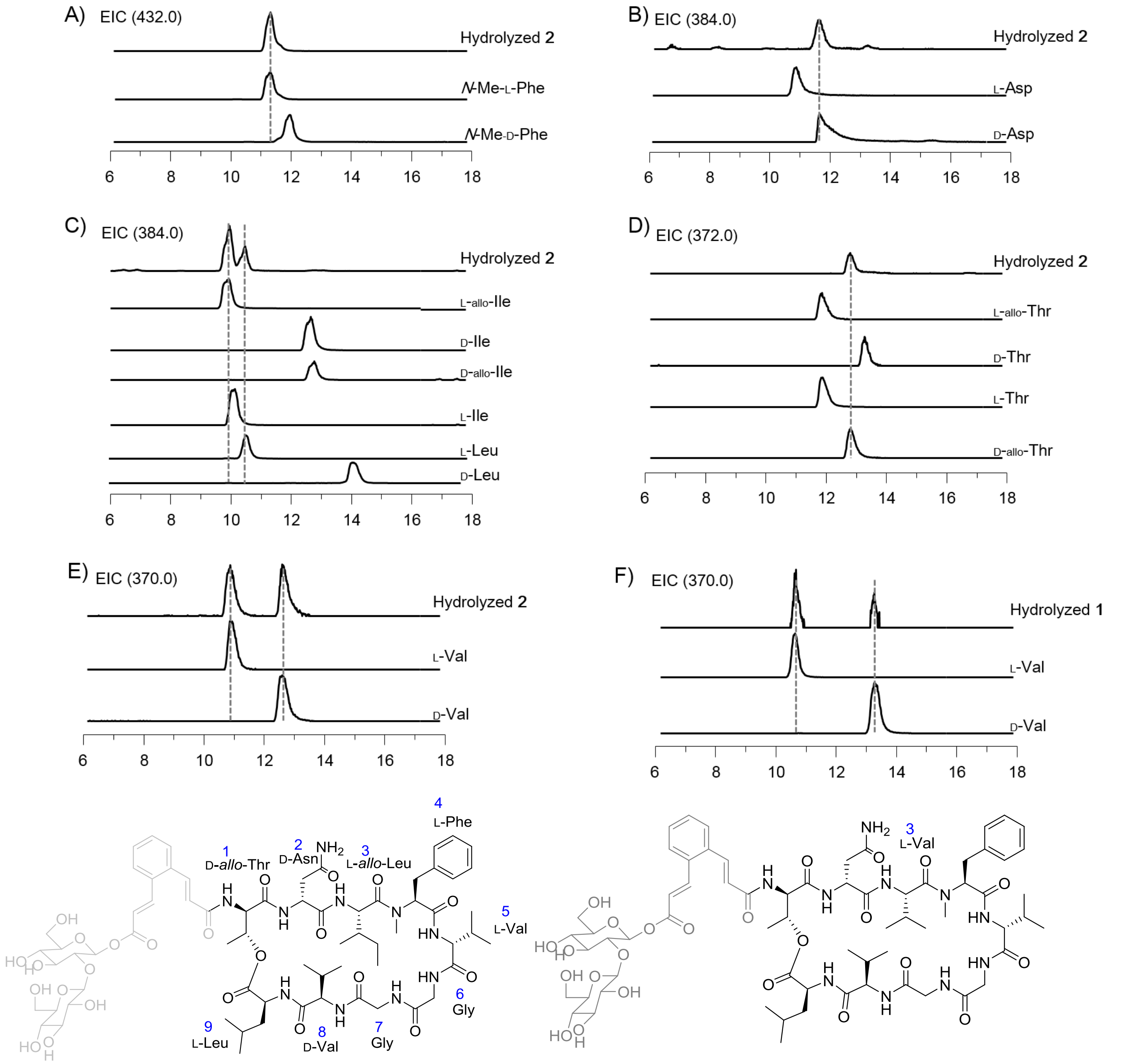

2 **1**

Figure S6. LC-MS analysis of L-FDAA derivatives of the amino acid residues in **1** and **2**. Panel A indicates 4th Phe in **2** is L-type; Panel B indicates 2nd Asn in **2** is D-type,; Panel C indicates 3rd Ile in **2** is L-allo-type, and 9th Leu in **2** is L-type; Panel D indicates 1st Thr in **2** is D-allo-type; Panel E, in combination with the presence of an E domain in module 8, indicates 5th and 8th Val in **2** are L- and D-type, respectively. Panel F has a 2:1 ratio of L-val and D-val, suggesting the additional 3rd Val in **1** is L-type. The deduced D-type configurations in 1st, 2nd, and 8th amino acid residues are consistent with the presence of E domains in their corresponding modules (Figure 1).

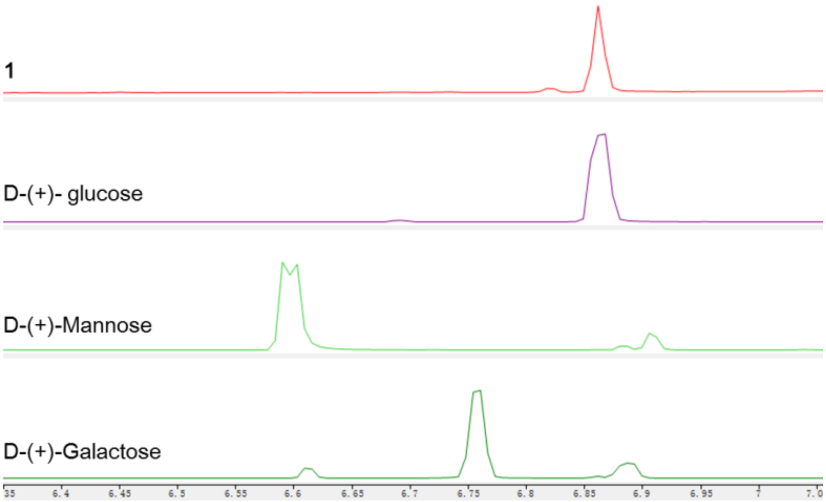

Figure S7. GC-MS analysis of the trimethylsilyl derivatives of the hydrolyzed **1**.

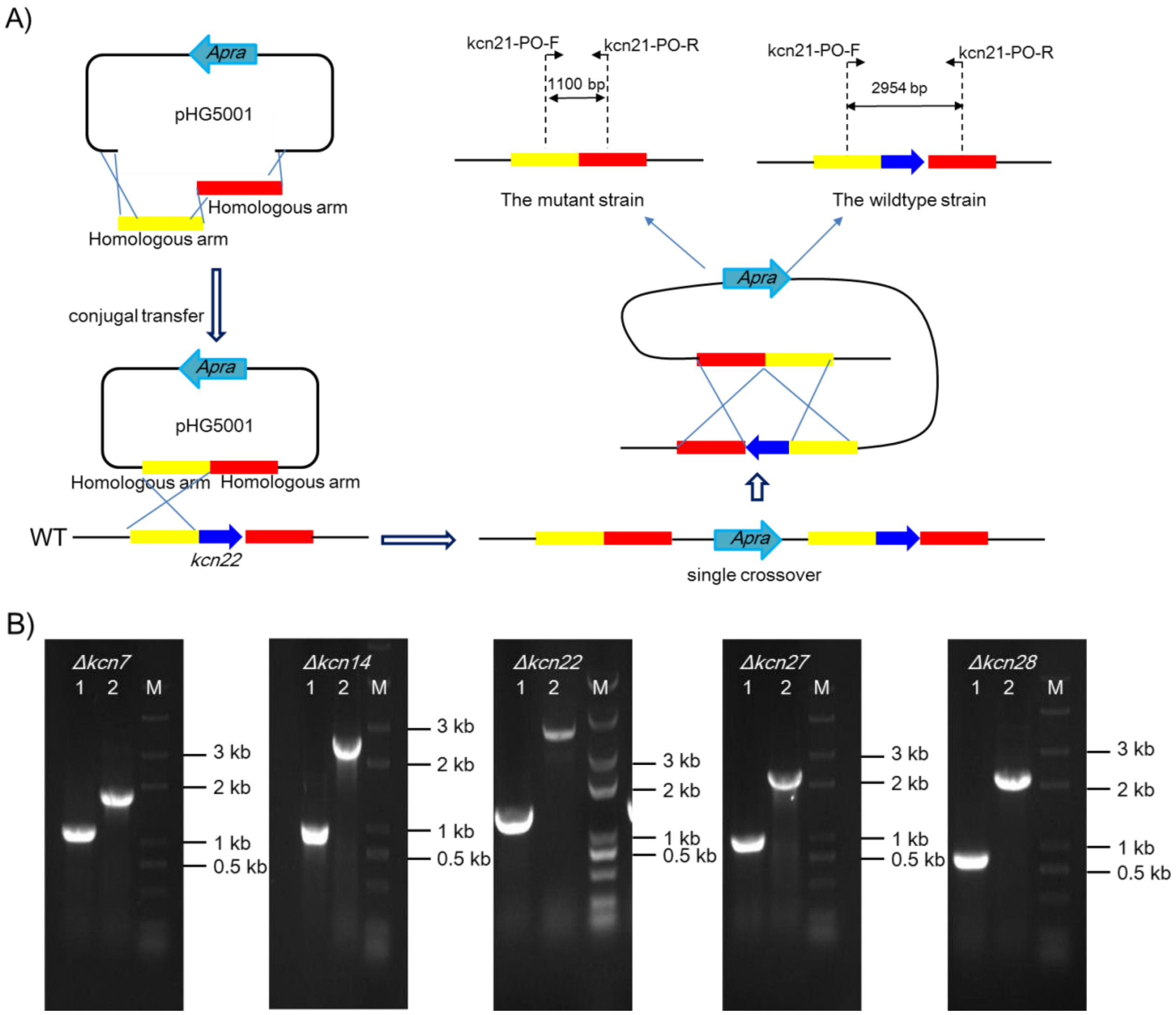

Figure S8. Construction of in-frame deletion in CGMCC 16924. A) Gene disruption with homologous recombination strategies. B) PCR verification of *kcn* mutants: Lane1, amplified with PO-F/R and mutants; Lane 2, amplified with PO-F/R and WT.

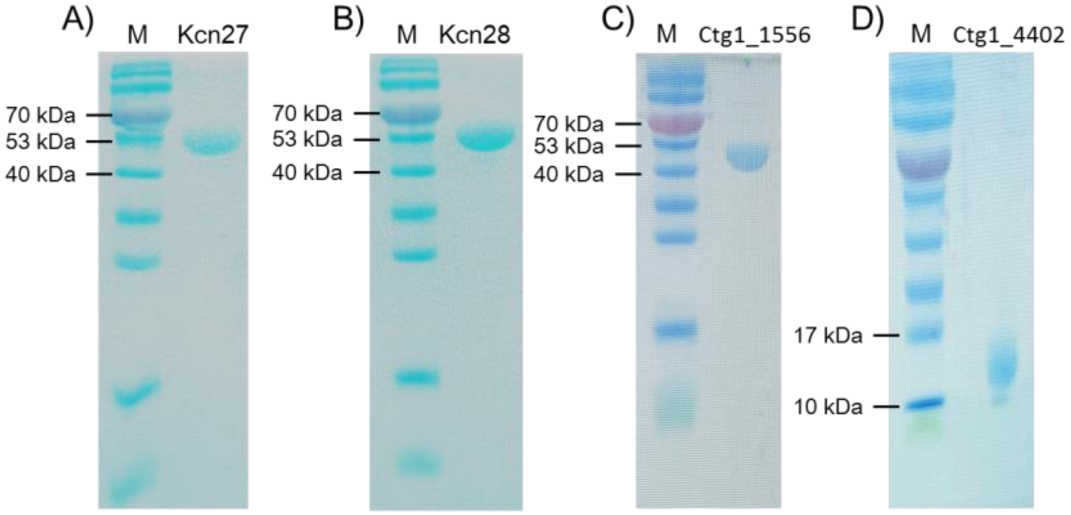

Figure S9. SDS-PAGE analysis of proteins. A) Kcn27 (calculated molecule weight: 44.9 KDa); B) Kcn28 (calculated molecule weight: 45.5 KDa); C) Ctg1_1556 (ferredoxin reductase from CGMCC 16924, calculated molecule weight: 44.6 KDa); D) Ctg1_4402 (ferredoxin from CGMCC 16924, calculated molecule weight: 14.2 KDa).

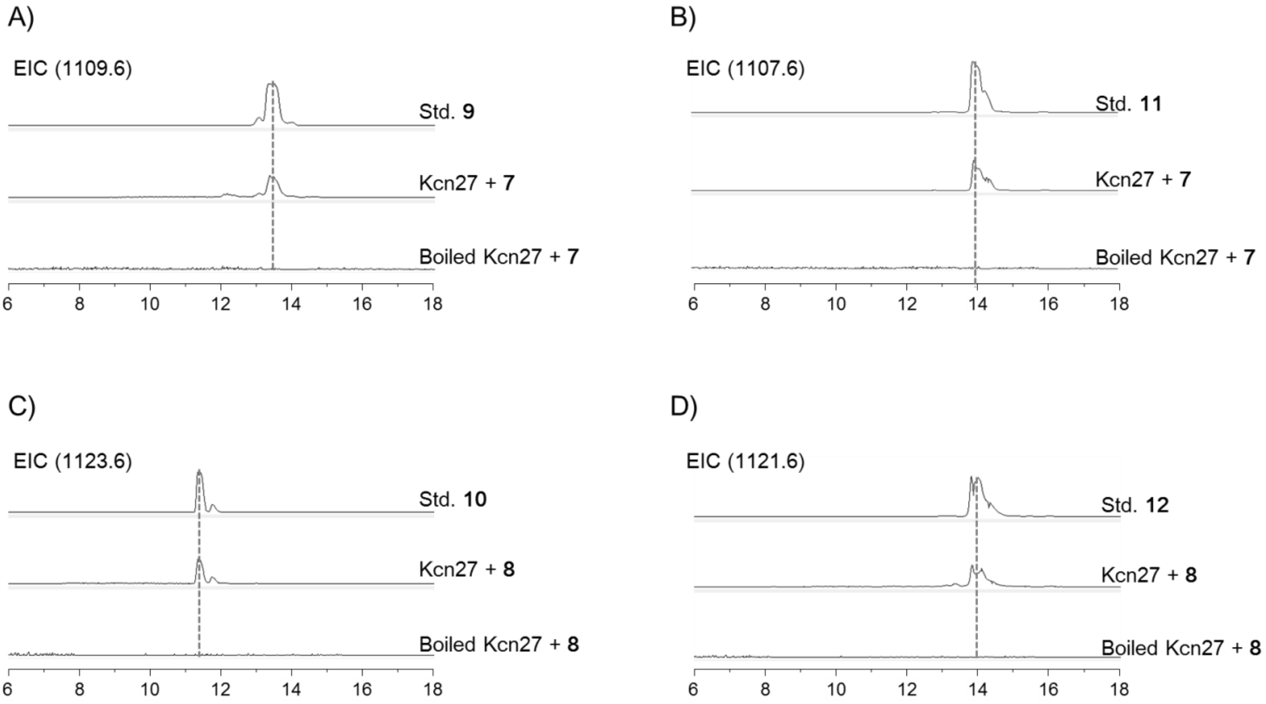

Figure S10. LC-MS analysis of Kcn27-catalyzed reactions.

A)

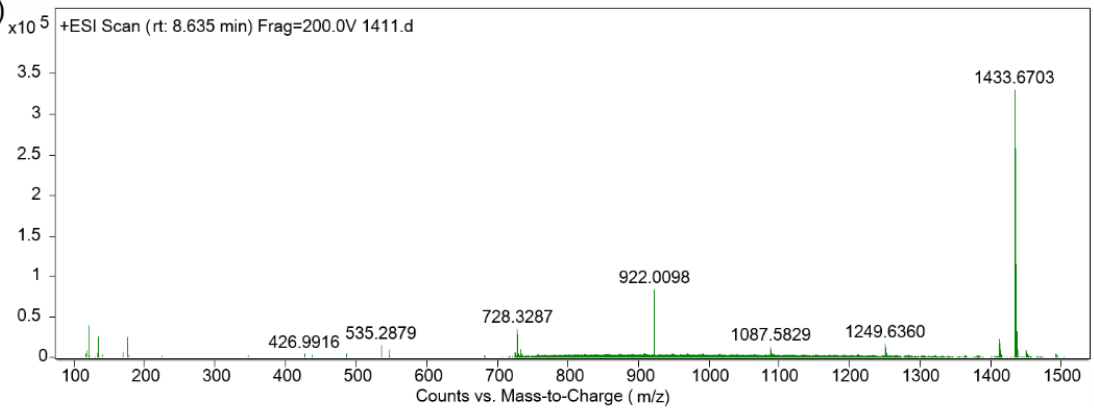

B)

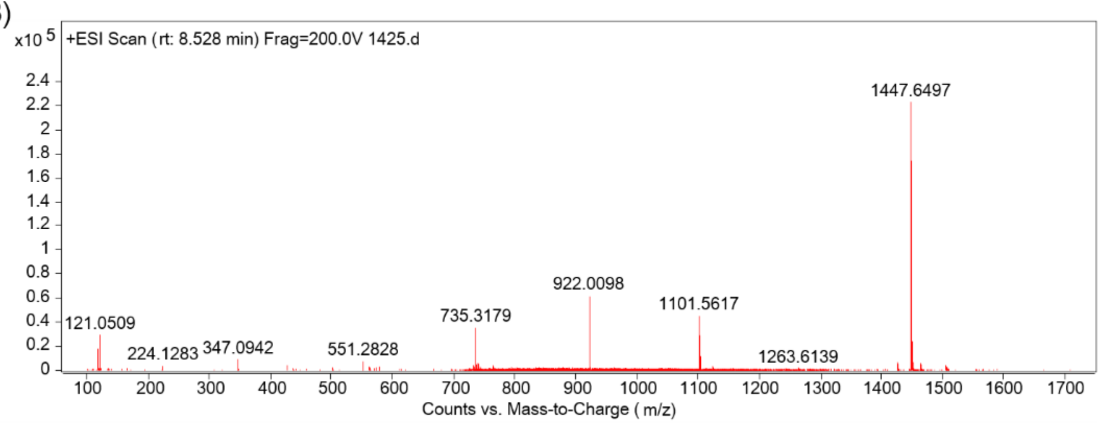

Figure S11. HRESIMS spectra of **13** (A) and **14** (B).

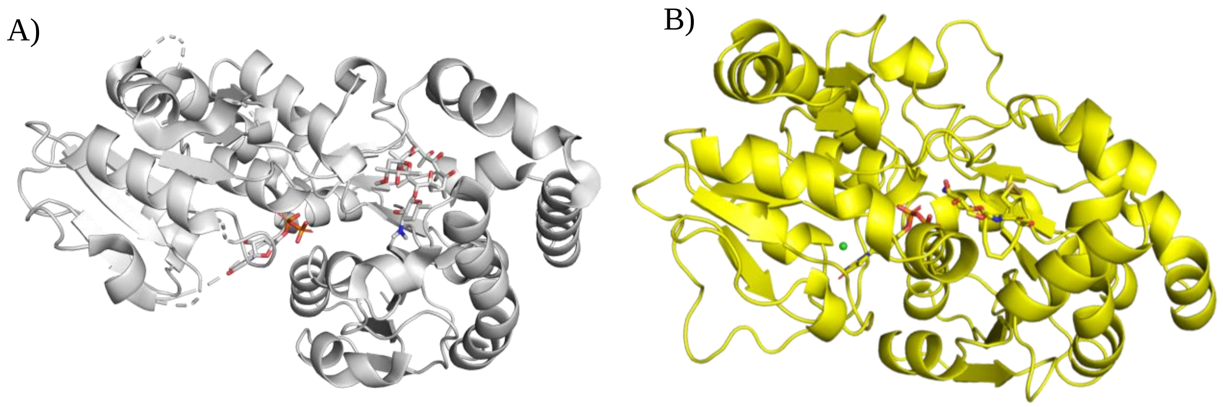

Figure S12. Crystal structure of OleD and CalG3. A) Crystal structure of OleD (PDB: 2IYF). B) Crystal structure of CalG3 (PDB: 3OTI)

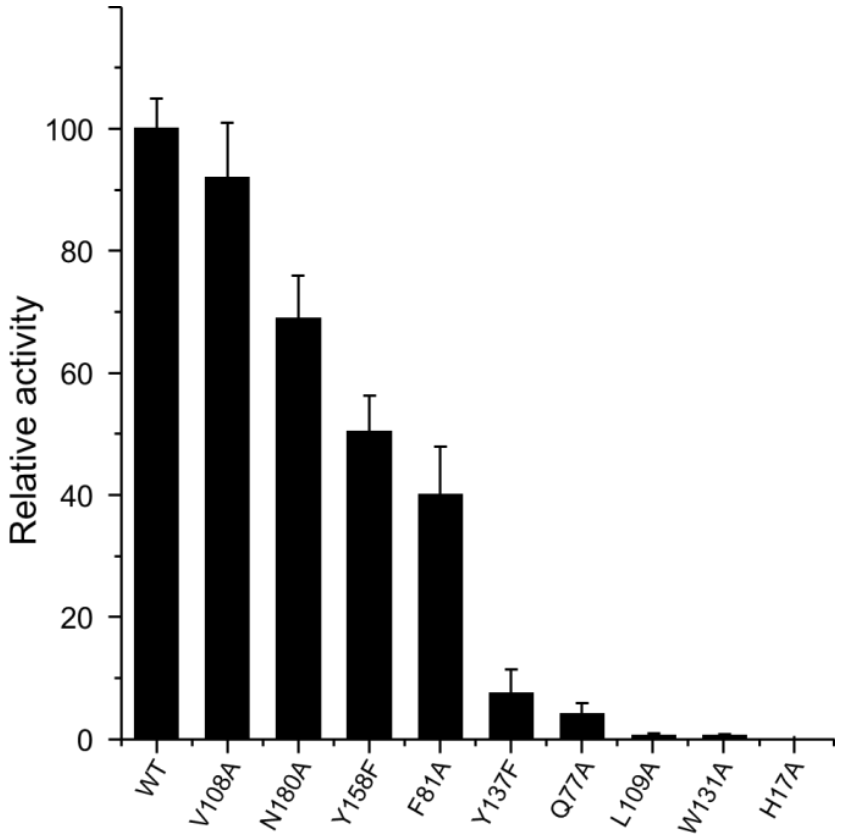

Figure S13. Relative activities of Kcn28 and its site-specific mutants on enzymatic reactions.

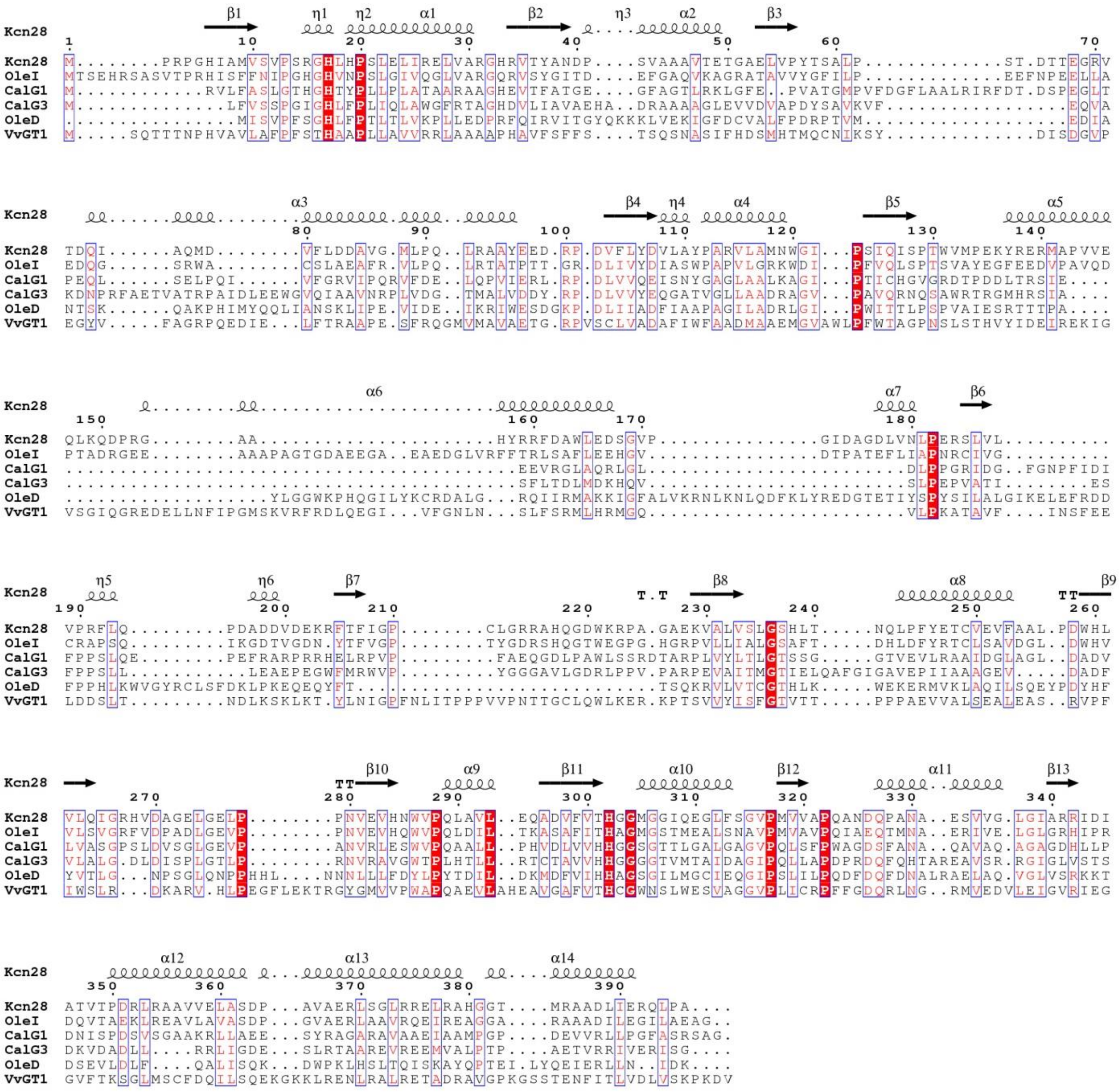

Figure S14. Sequence alignment of Kcn28 and its homologues. The alignment was created with MUSCLE[14] and rendered with ESPript 3.0.[15]

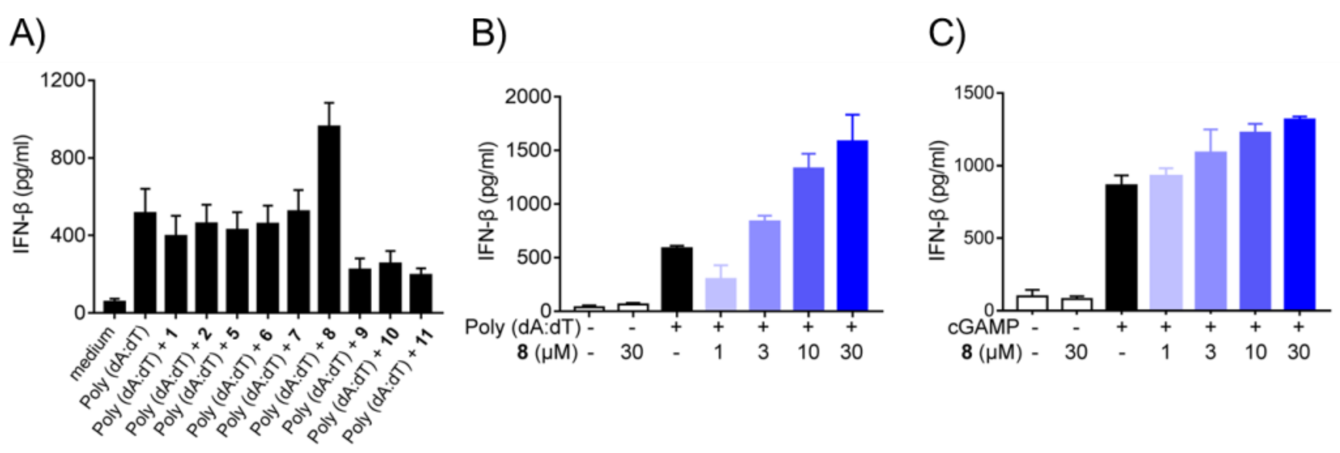

Figure S15. Compound **8** dose dependently promote IFN-β production induced by poly(dA:dT) and cGAMP. A) PMA-differentiated THP1 cells were treated with compounds with or without Poly(dA:dT) (2 μg/ml) for 6 h. Supernatants were collected and subjected for ELISA analysis of IFN-β. B) PMA-differentiated THP1 cells were treated with compound **8** with or without Poly(dA:dT) (2 μg/ml) for 6 h. Supernatants were collected and subjected for ELISA analysis of IFN-β. C) PMA-differentiated THP1 cells were treated with compound **6** with or without cGAMP (1 μg/ml) for 6 h. Supernatants were collected and subjected for ELISA analysis of IFN-β.

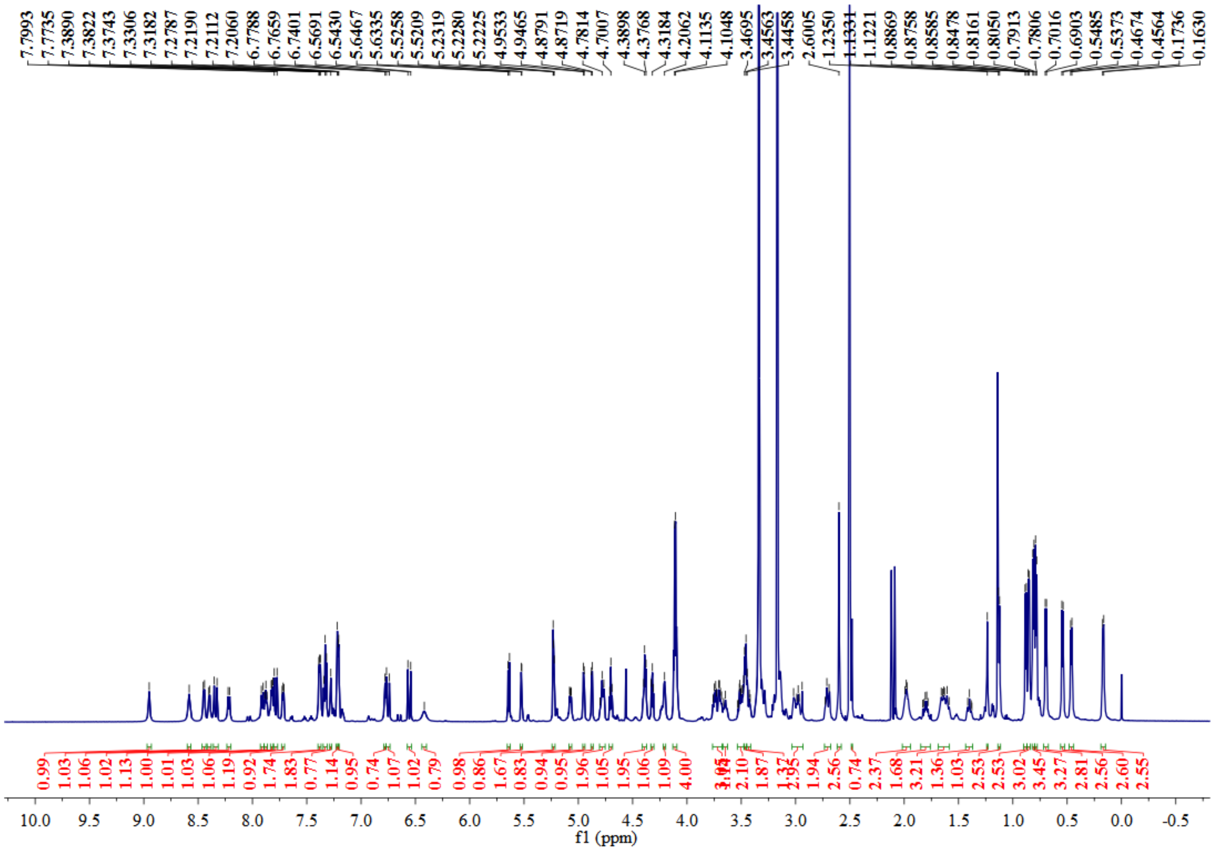

Figure S16. ^{1}H NMR spectrum of **1** in DMSO-d_6 at 600 MHz.

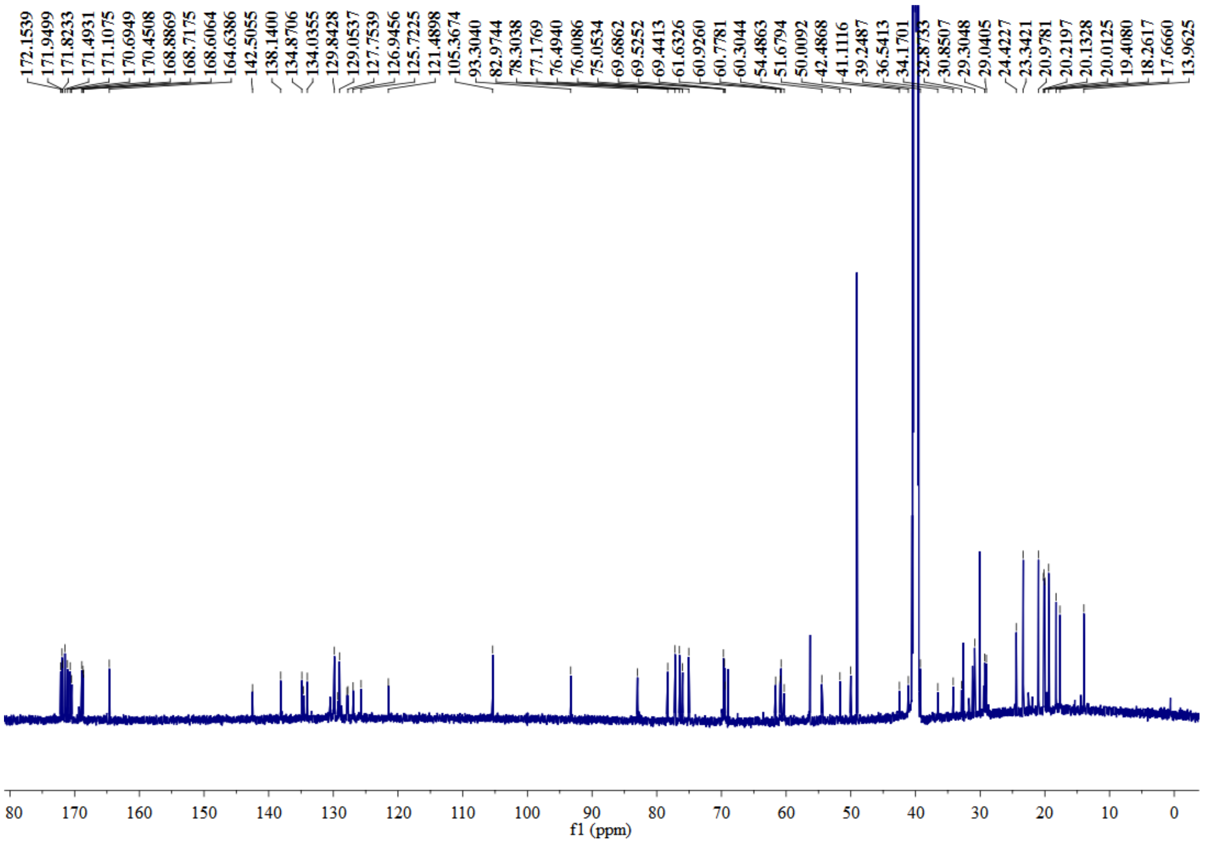

Figure S17. ^{13}C NMR spectrum of 1 in DMSO-d_6 at 150 MHz.

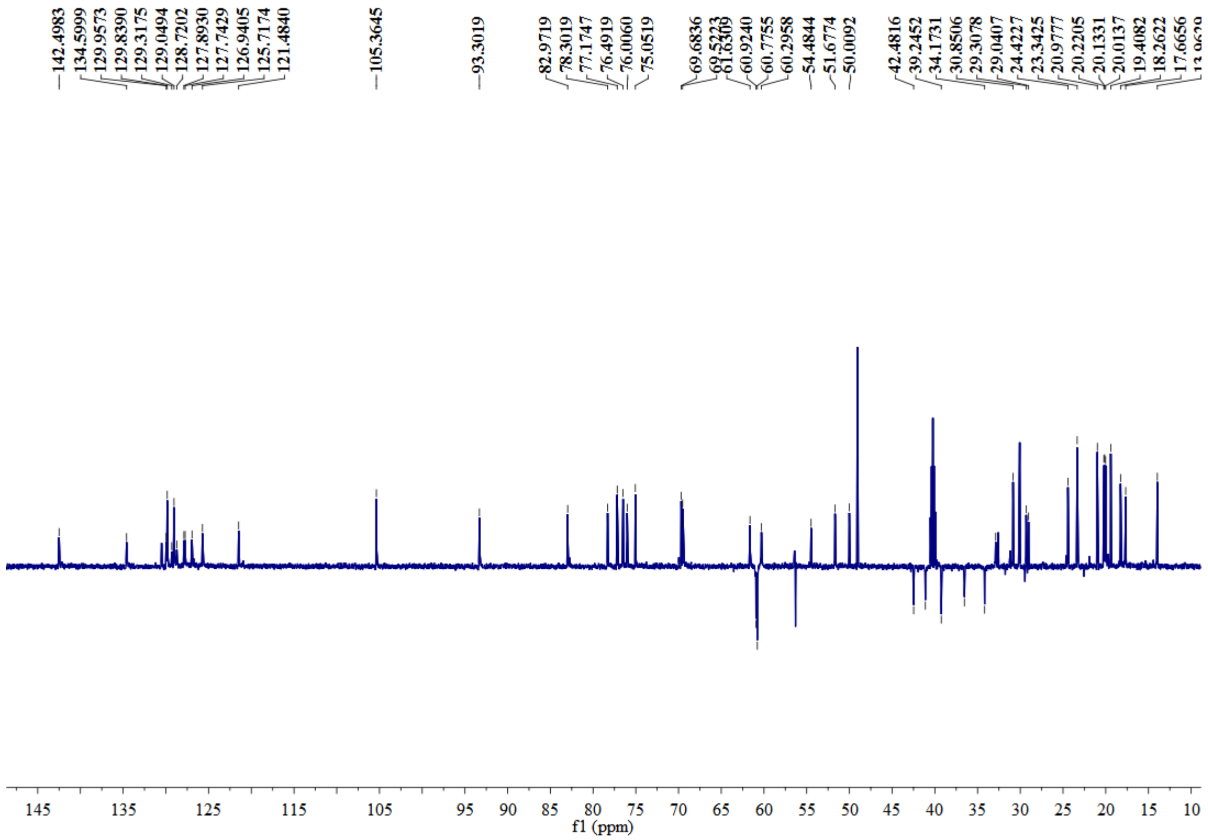

Figure S18. DEPT spectrum of **1** in DMSO-d_6.

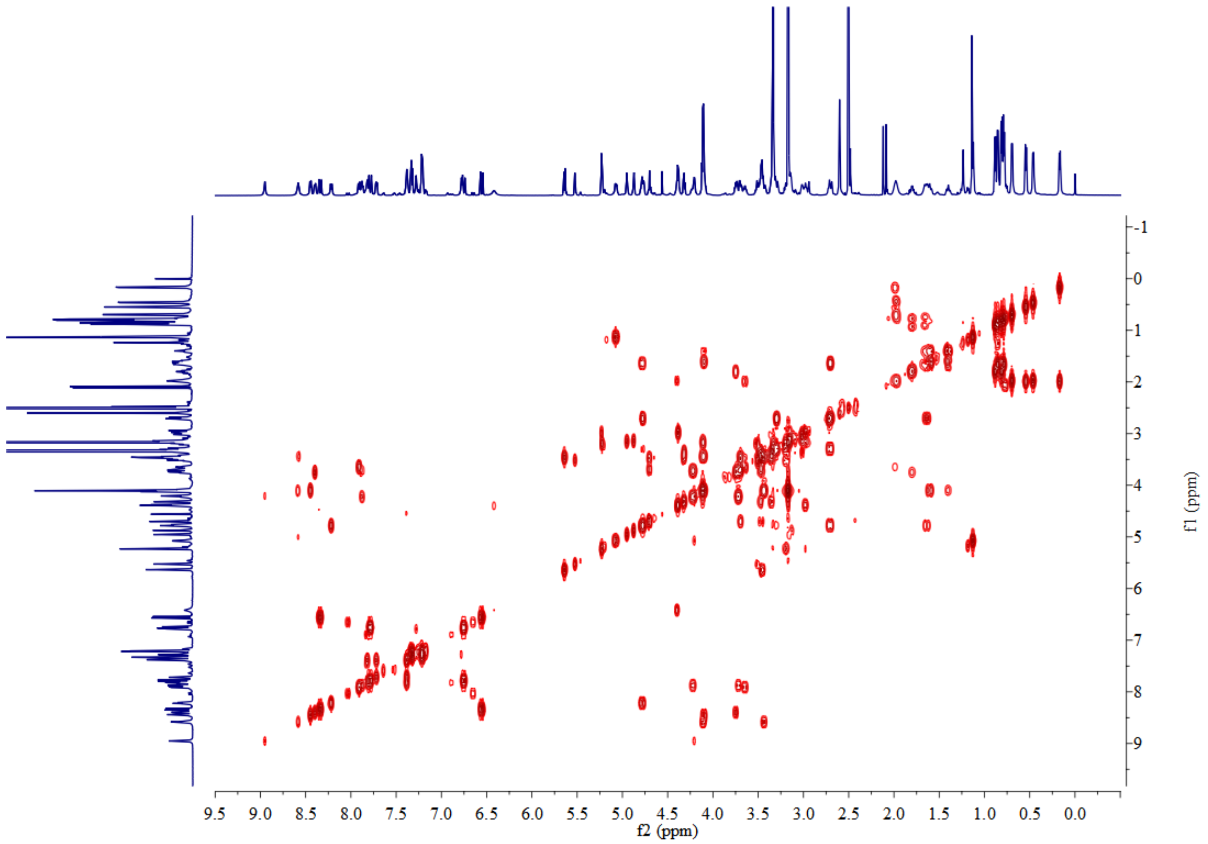

Figure S19. ^{1}H-^{1}H COSY spectrum of **1** in DMSO-d_6.

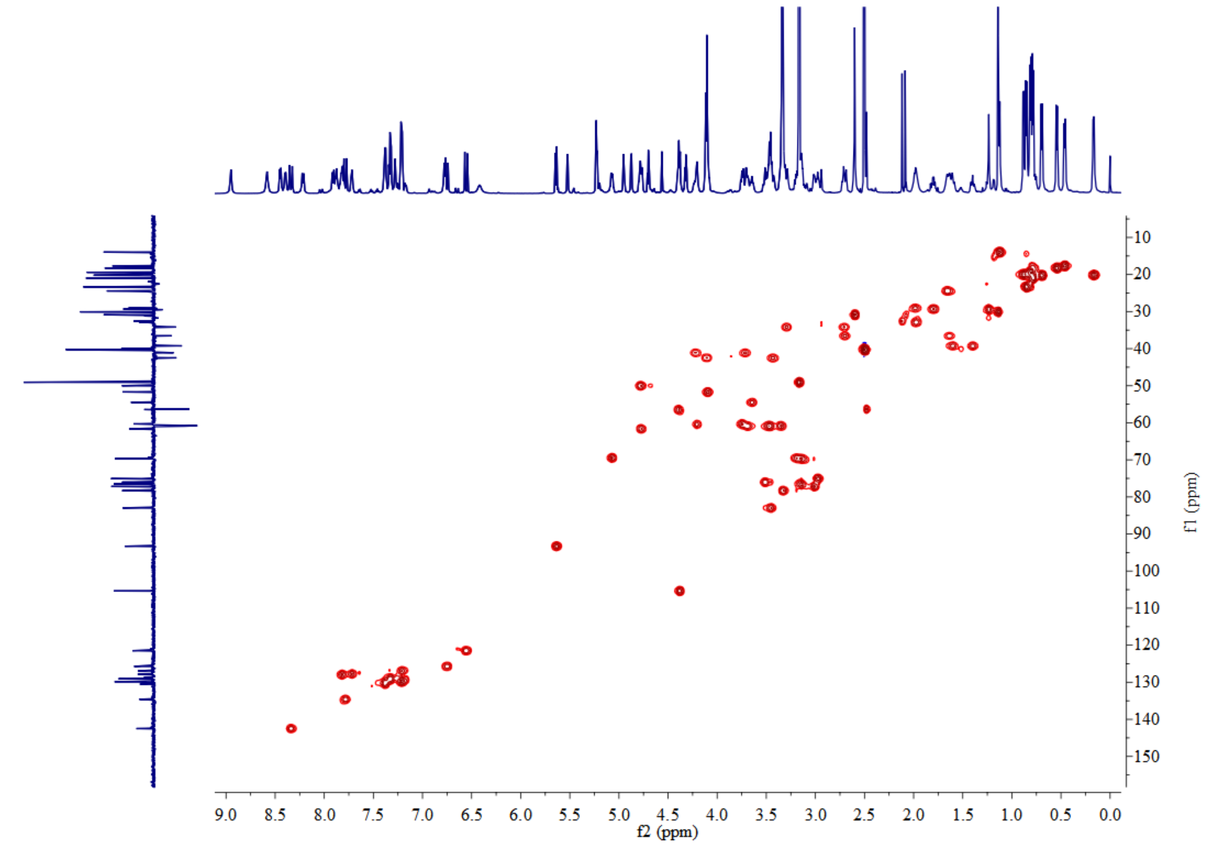

Figure S20. HSQC spectrum of **1** in DMSO-d_6.

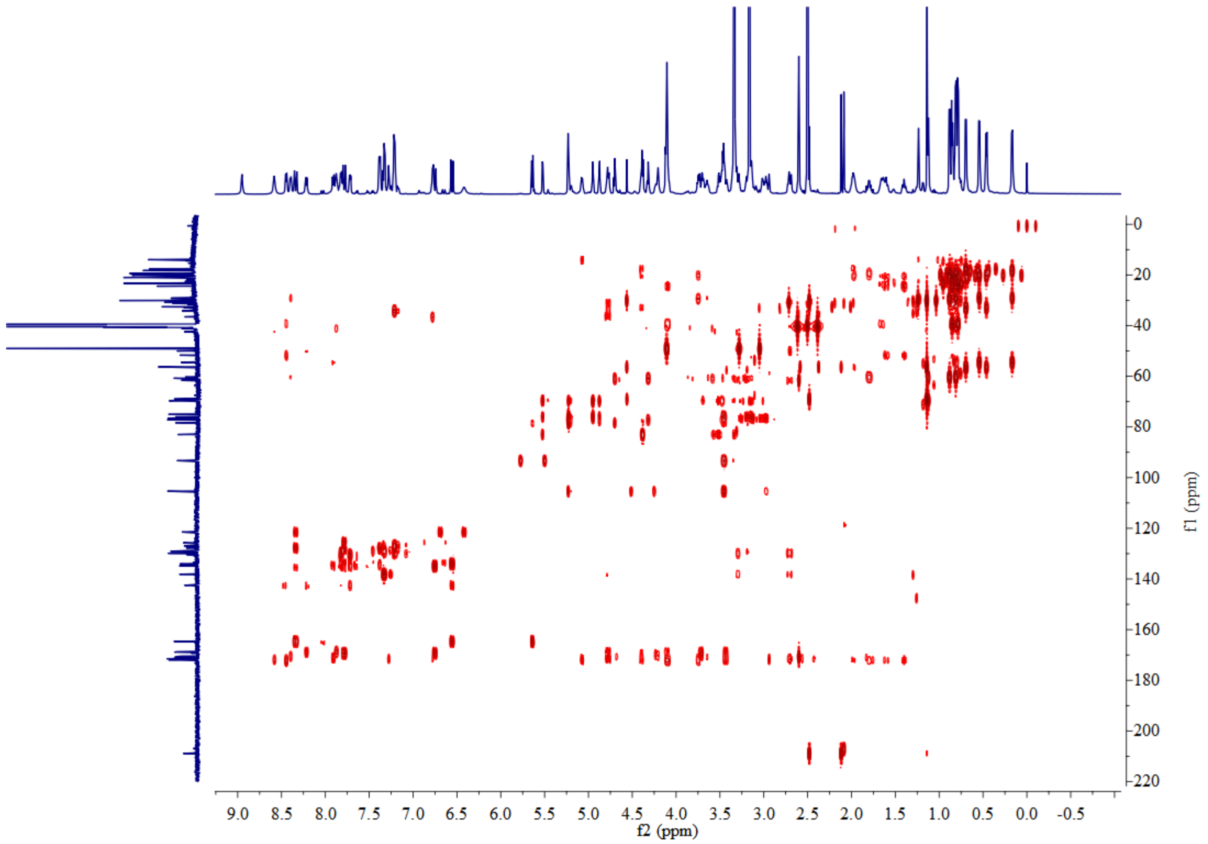

Figure S21. HMBC spectrum of 1 in DMSO-d_6.

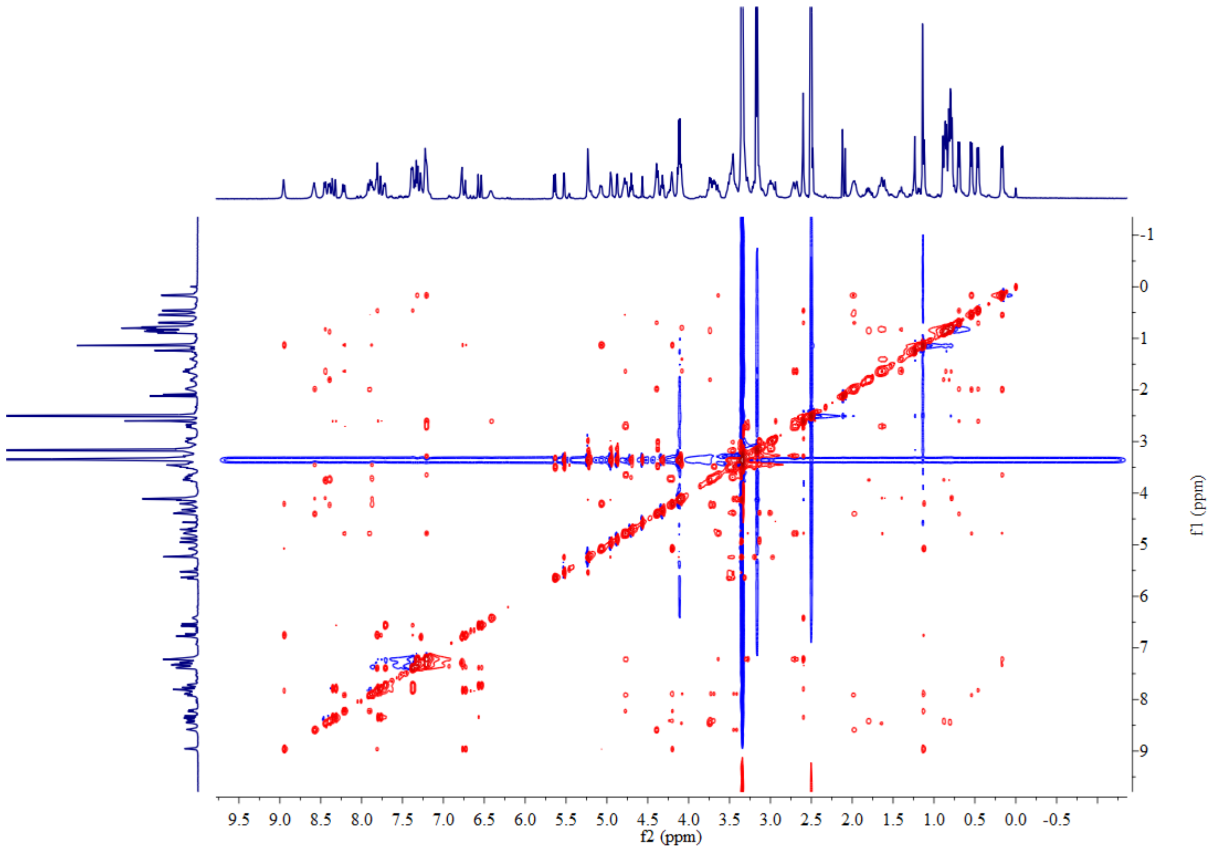

Figure S22. NOESY spectrum of **1** in DMSO-d_6.

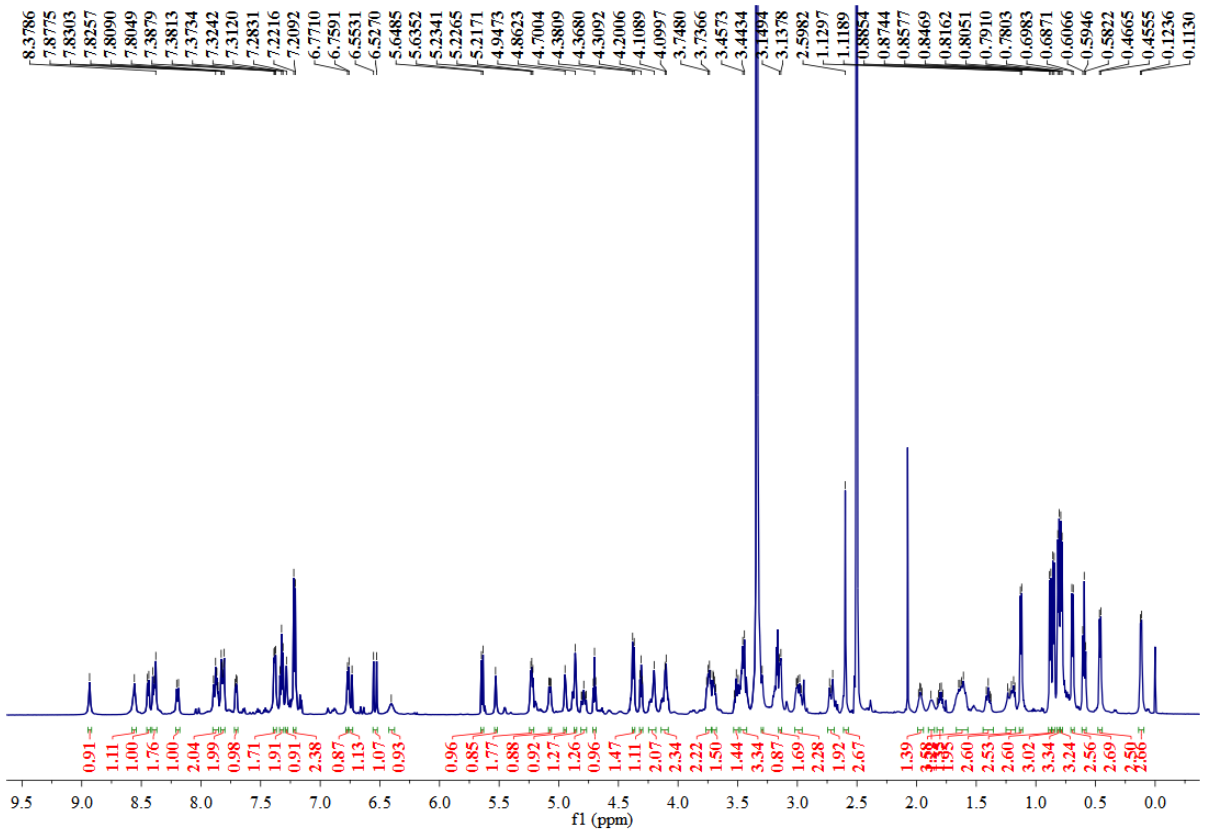

Figure S23. ^1H NMR spectrum of **2** in DMSO-d_6 at 600 MHz.

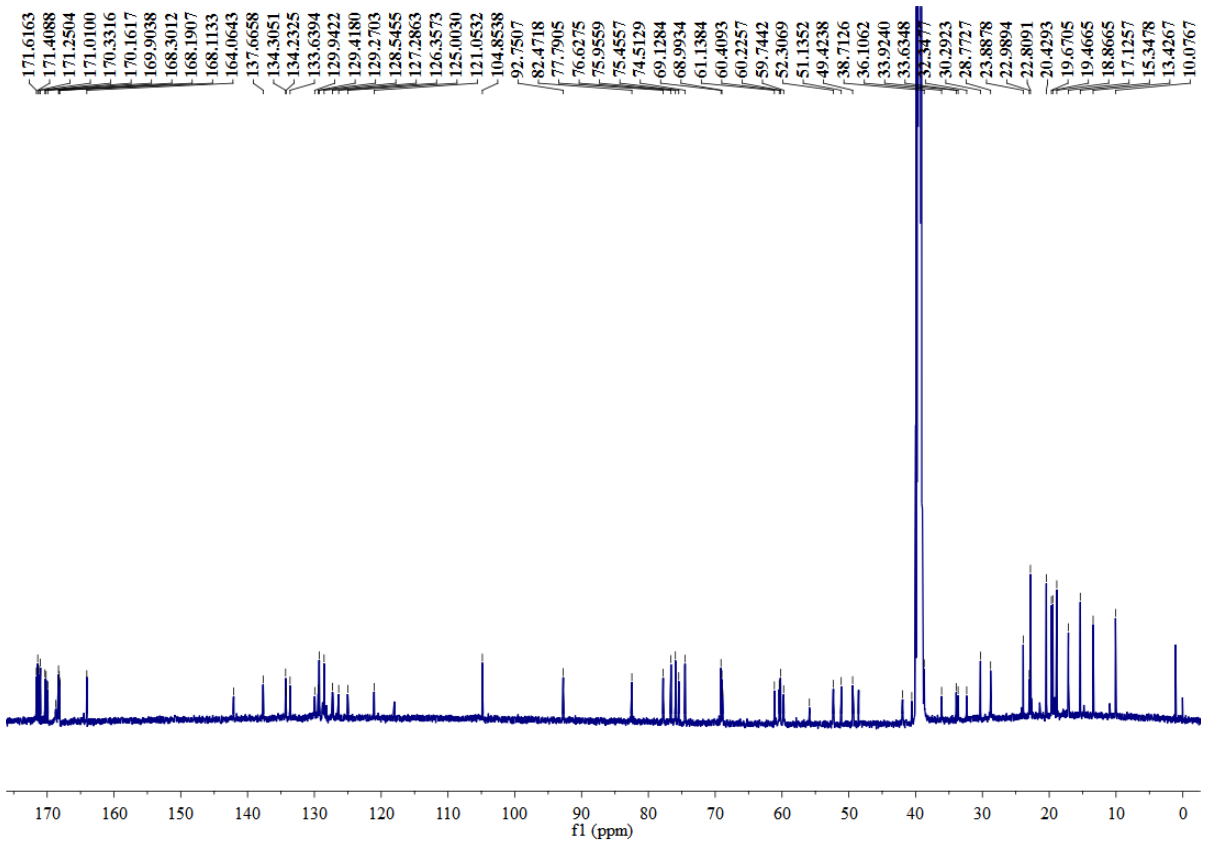

Figure S24. ^{13}C NMR spectrum of **2** in DMSO-d_6 at 150 MHz.

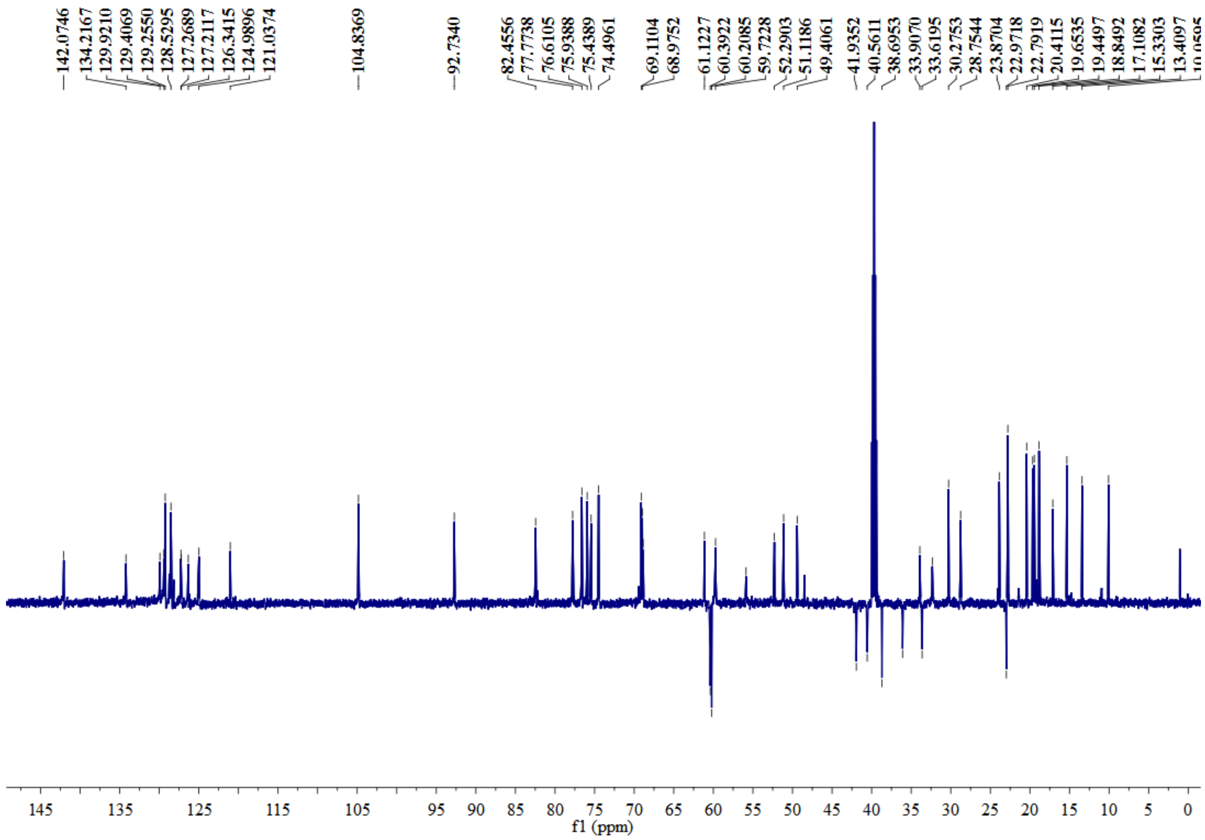

Figure S25. DEPT spectrum of **2** in DMSO-d_6.

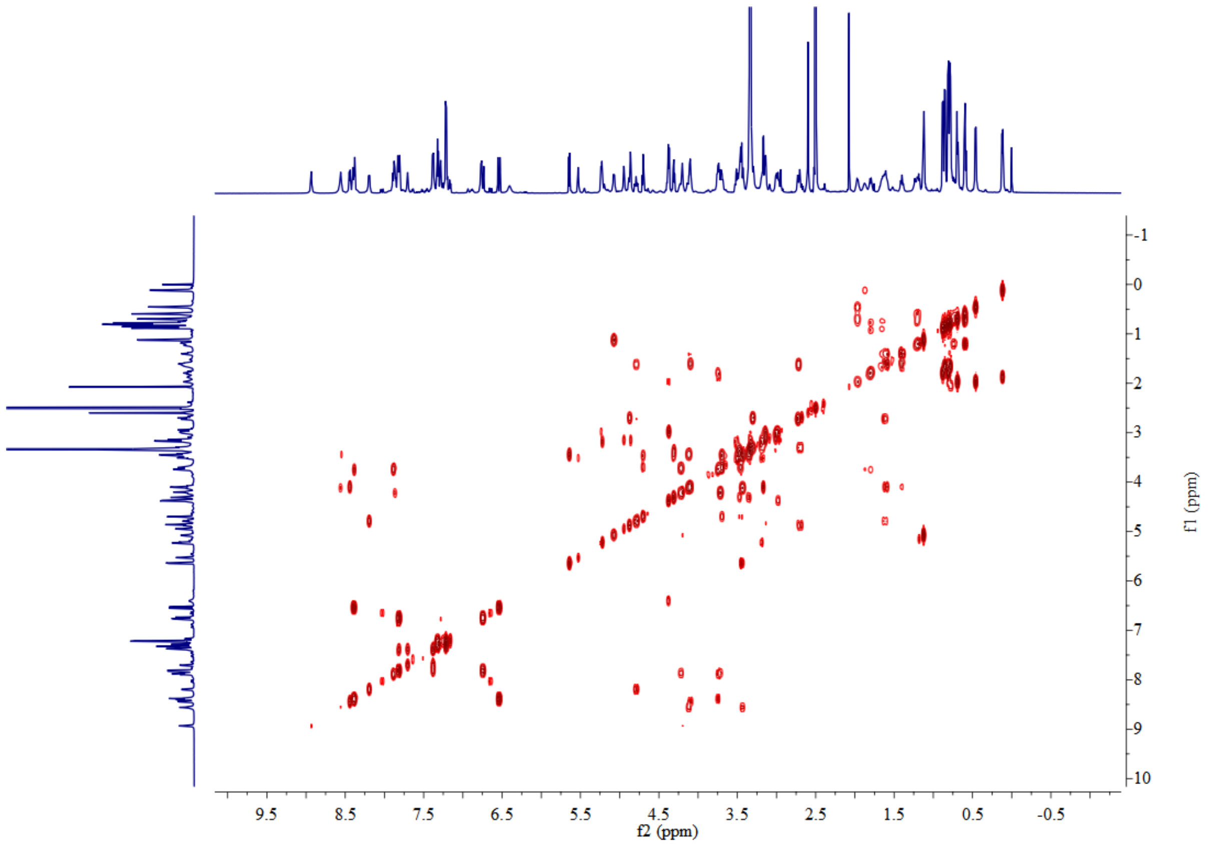

Figure S26. ^{1}H-^{1}H COSY spectrum of **2** in DMSO-d_6.

.

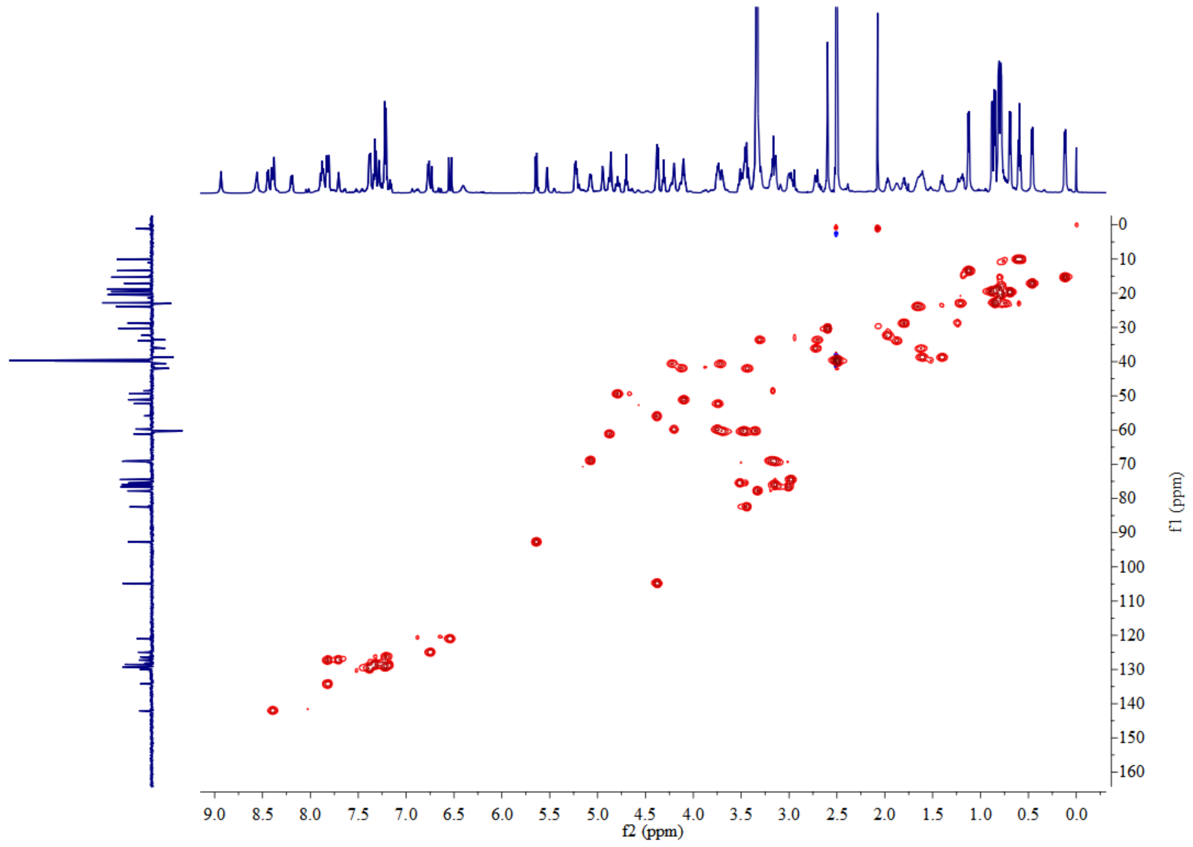

Figure S27. HSQC spectrum of **2** in DMSO-d_6

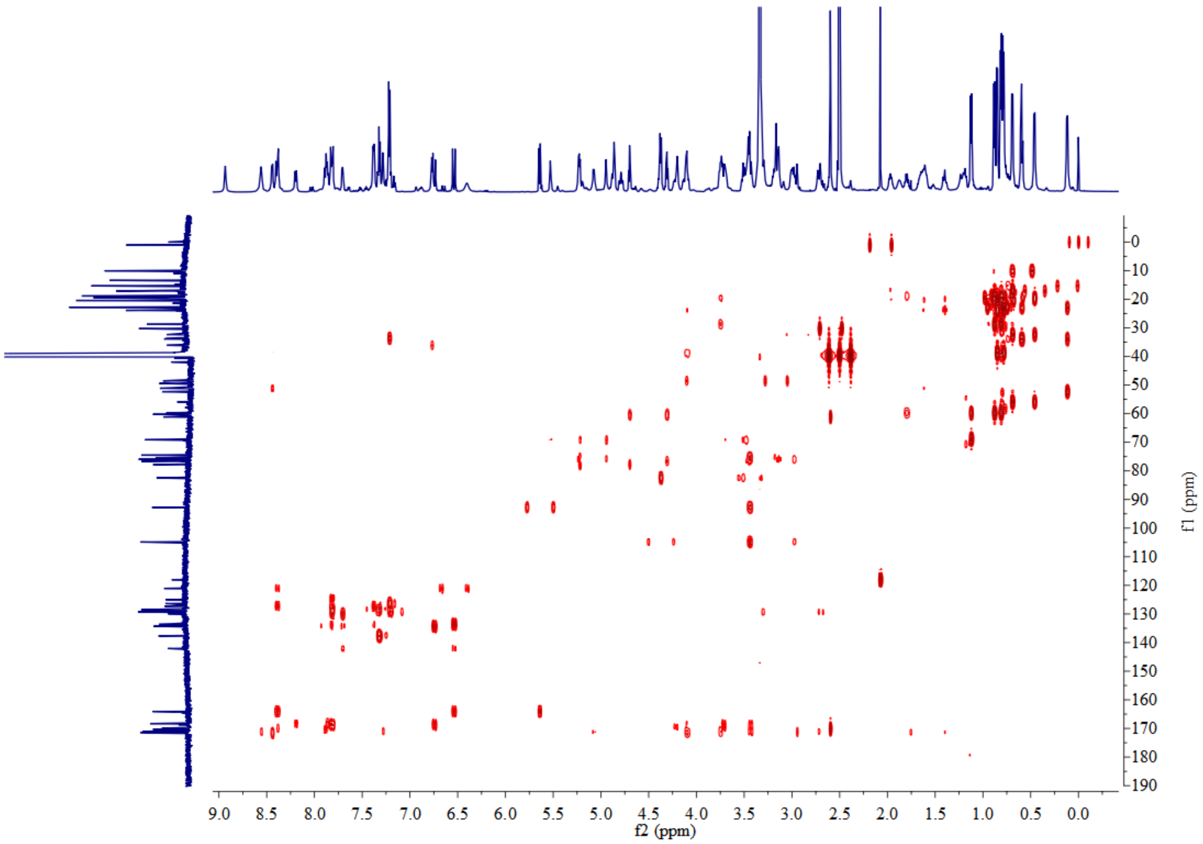

Figure S28. HMBC spectrum of **2** in DMSO-d_6.

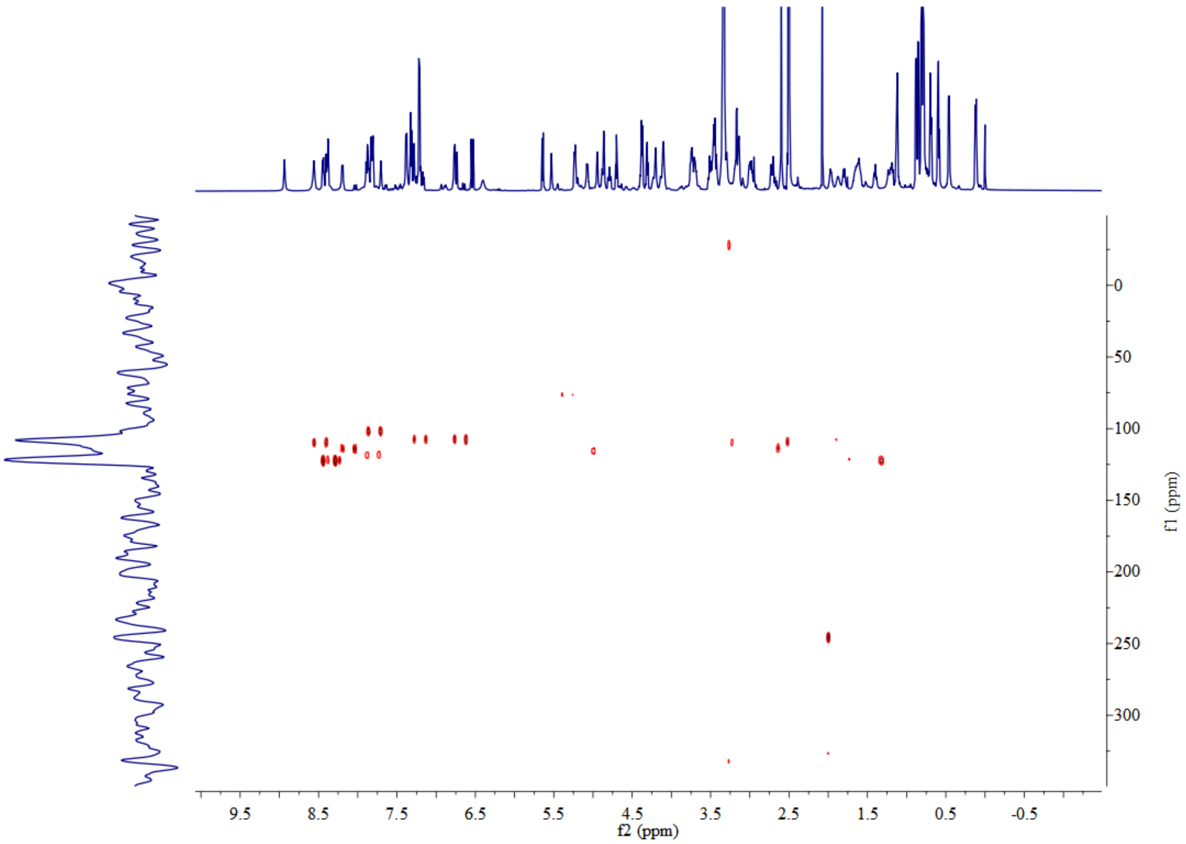

Figure S29. ^{1}H-^{15}N HMBC spectrum of **2** in DMSO-d_6.

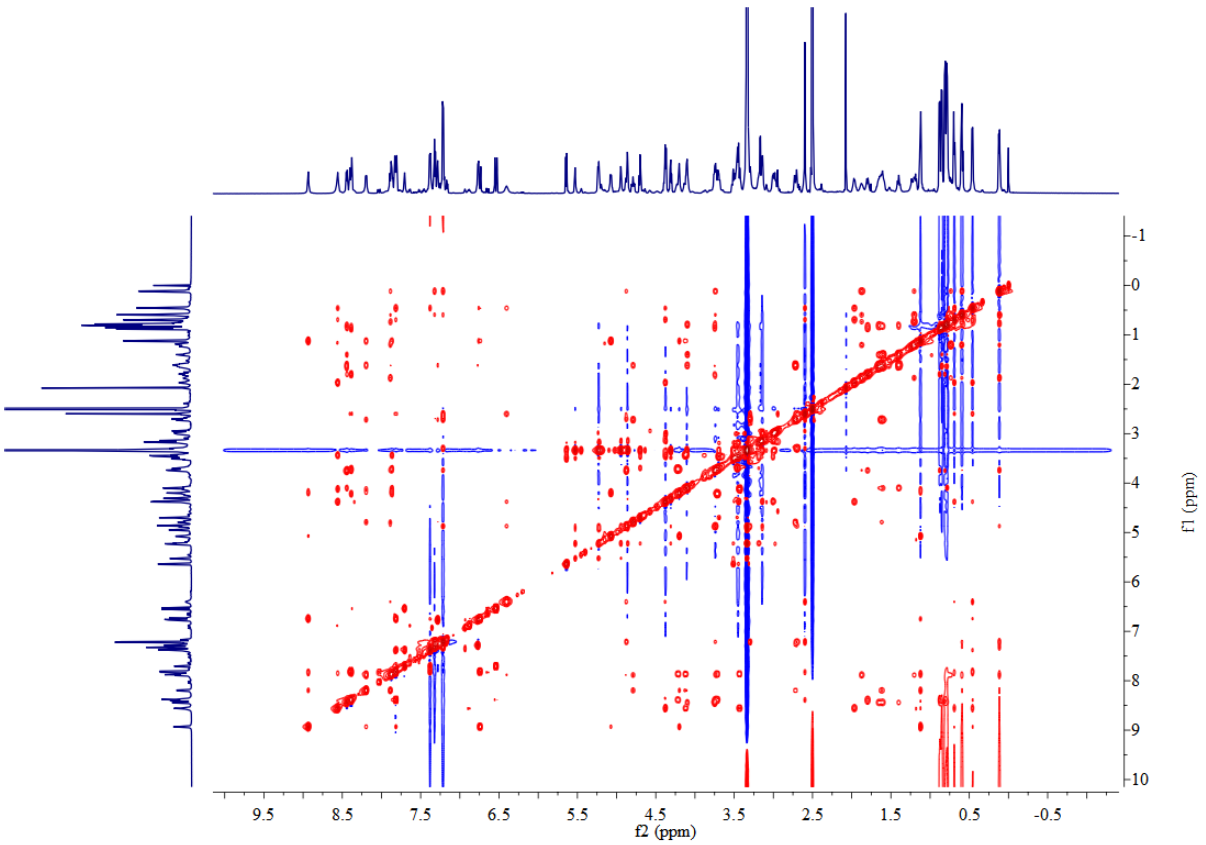

Figure S30. NOESY spectrum of **2** in DMSO-d_6.

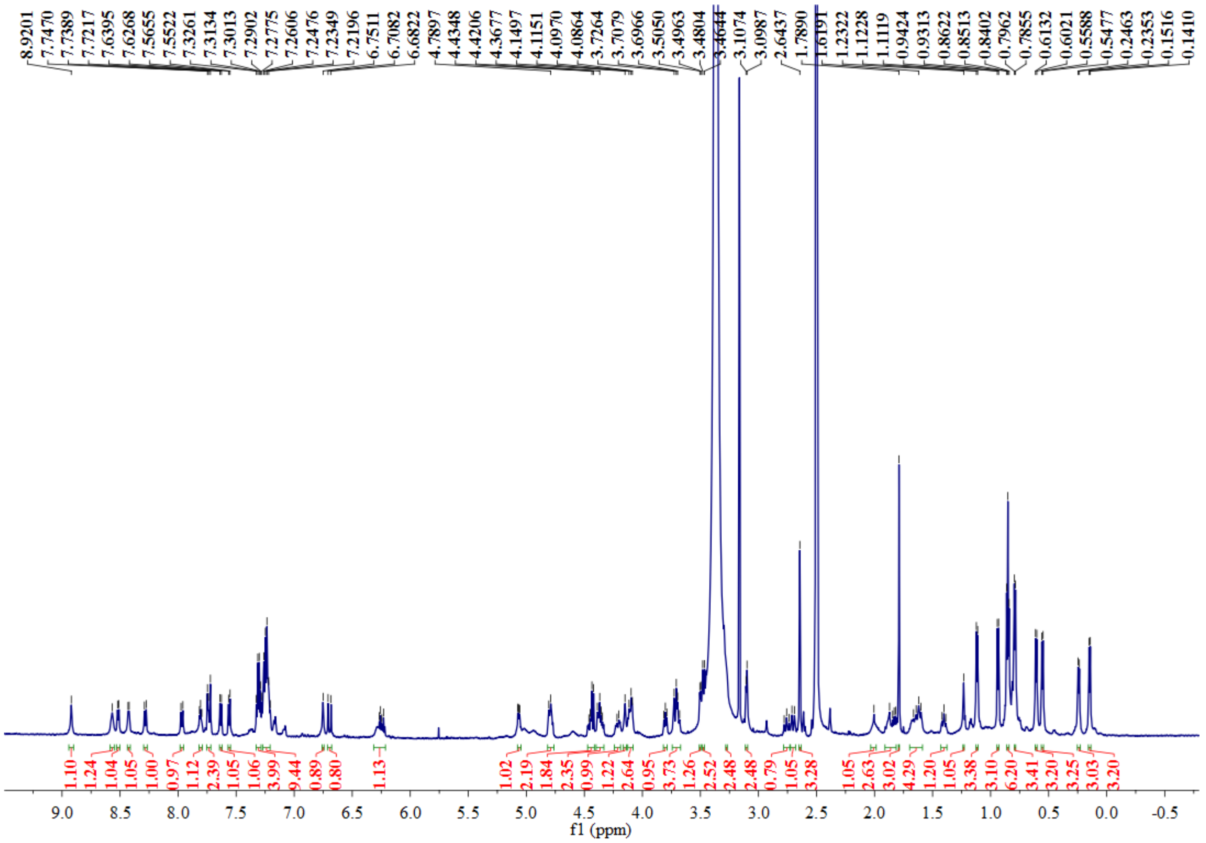

Figure S31. ^{1}H NMR spectrum of **3** in DMSO-d_6 at 600 MHz.

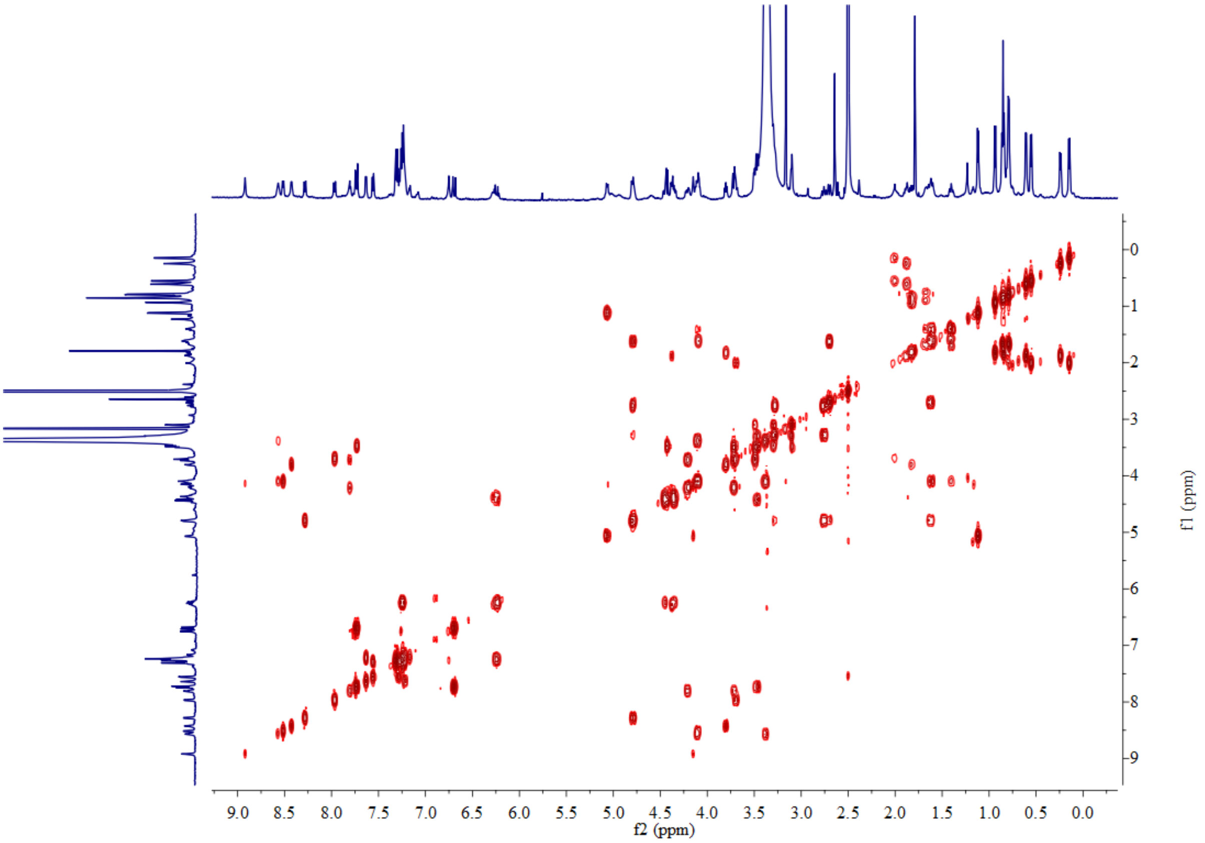

Figure S32. ^1H-^1H COSY spectrum of **3** in DMSO-d_6.

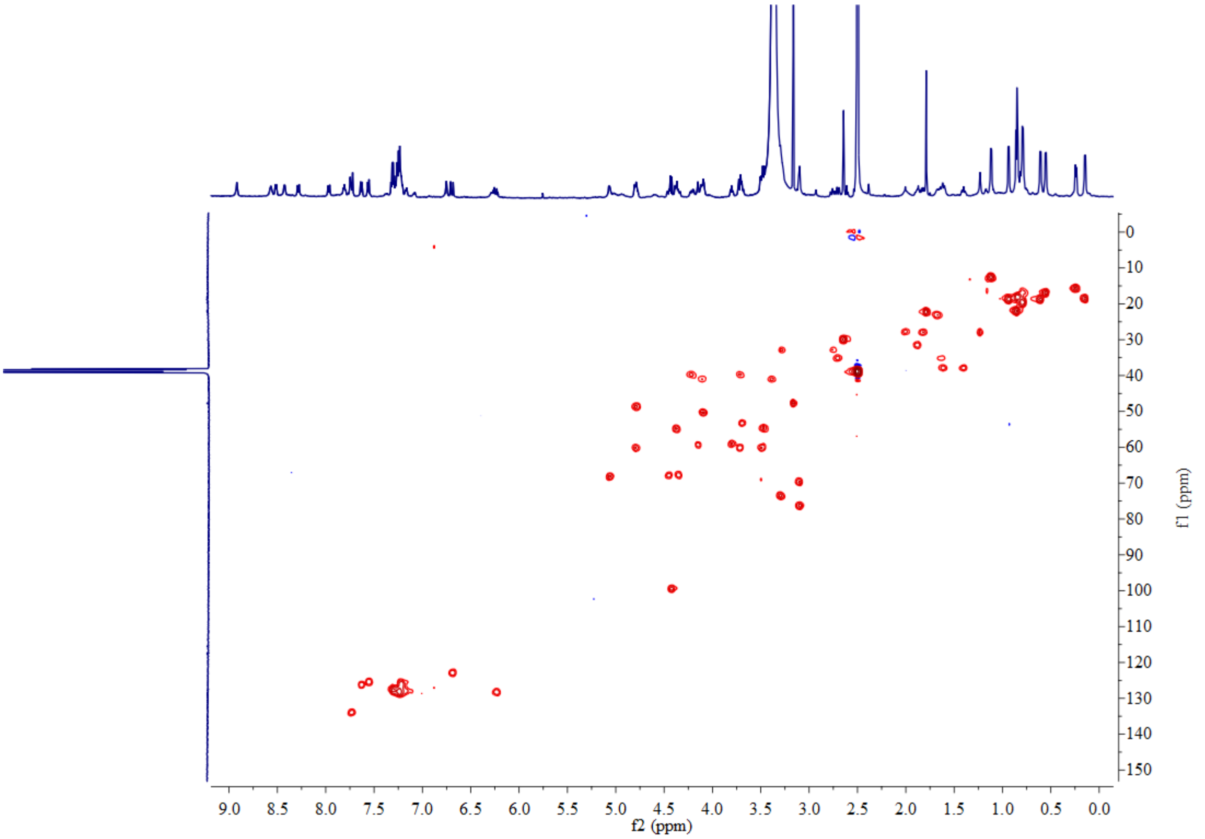

Figure S33. HSQC spectrum of **3** in DMSO-d_6.

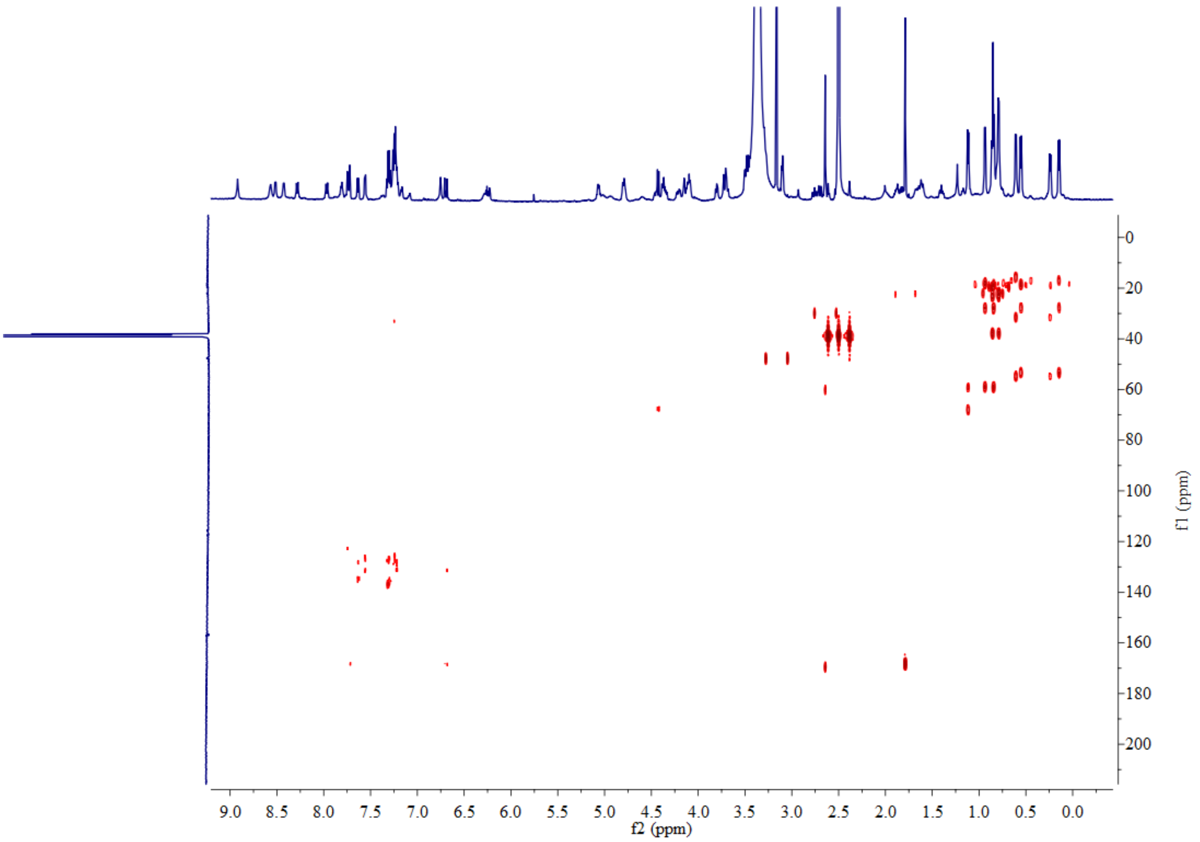

Figure S34. HMBC spectrum of **3** in DMSO-d_6.

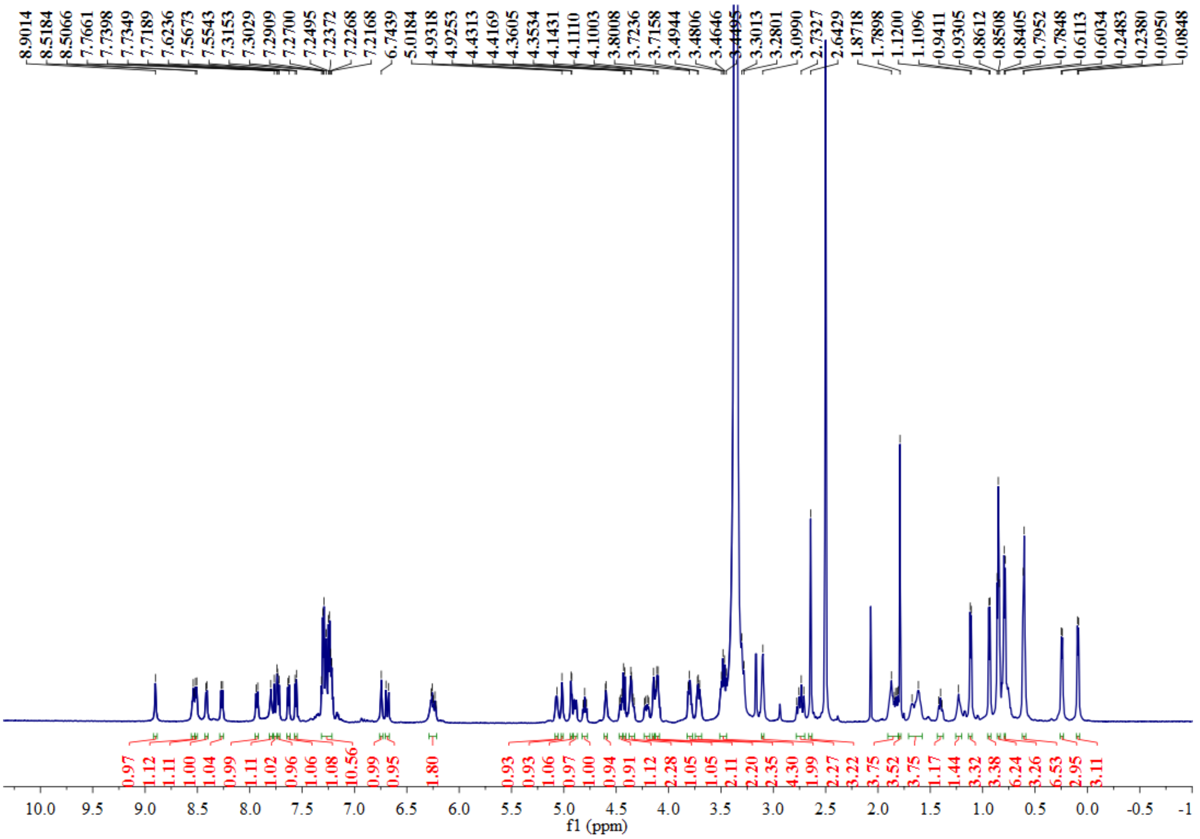

Figure S35. ^{1}H NMR spectrum of **4** in DMSO-d_6 at 600 MHz.

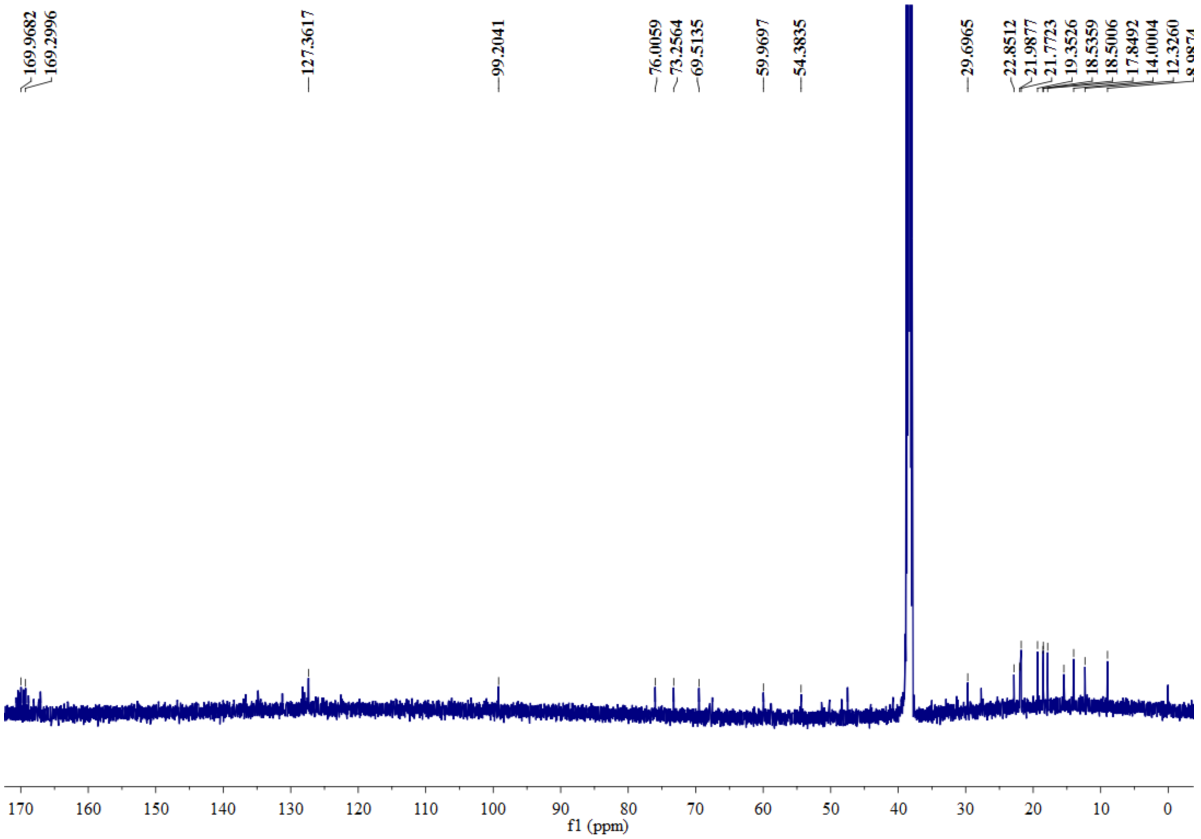

Figure S36. ^{13}C NMR spectrum of **4** in DMSO-d_6 at 150 MHz.

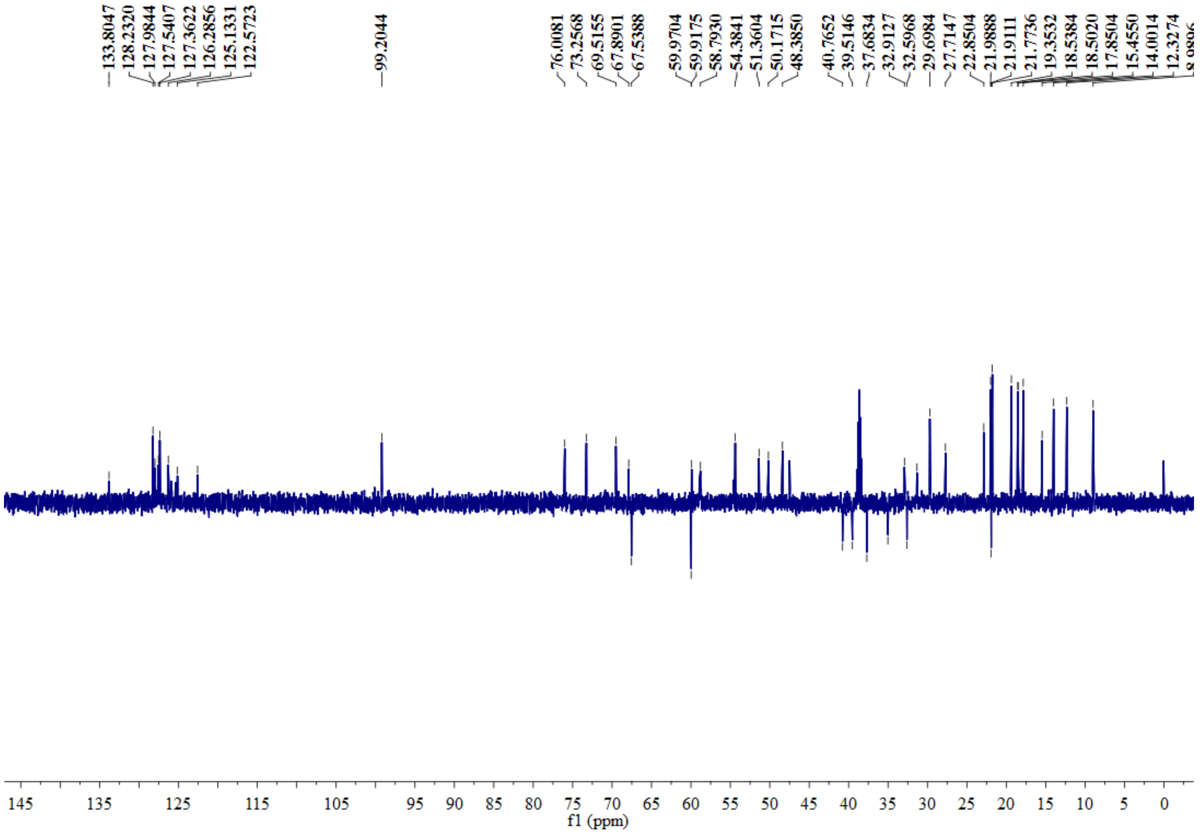

Figure S37. DEPT spectrum of **4** in DMSO-d_6.

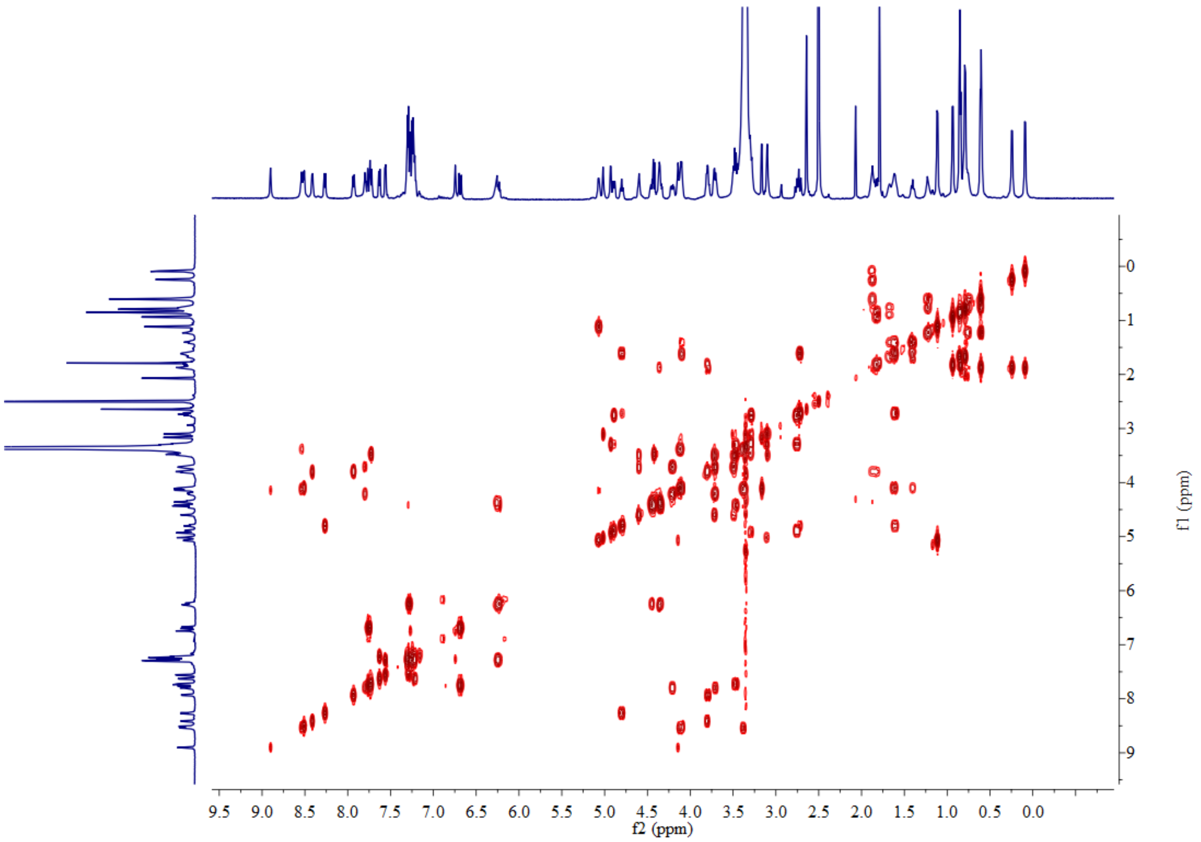

Figure S38. ^{1}H-^{1}H COSY spectrum of **4** in DMSO-d_6.

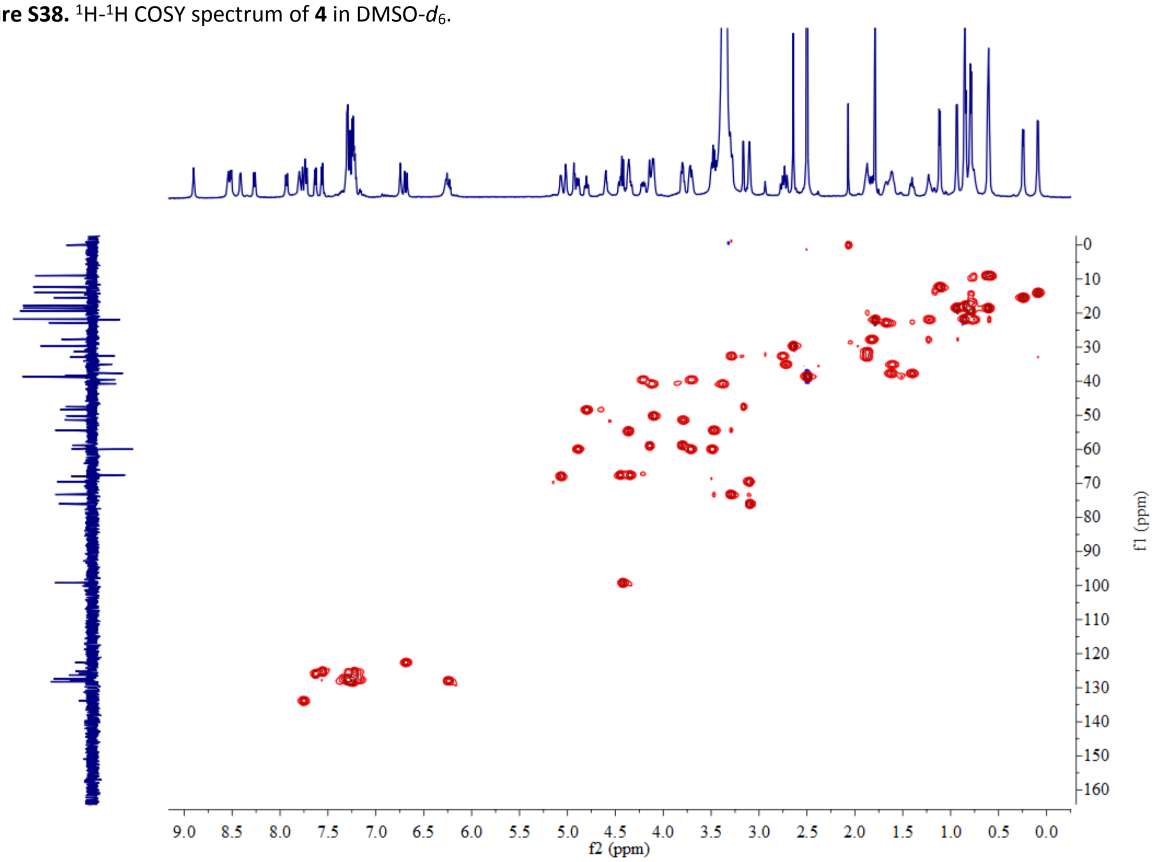

Figure S39. HSQC spectrum of **4** in DMSO-d_6.

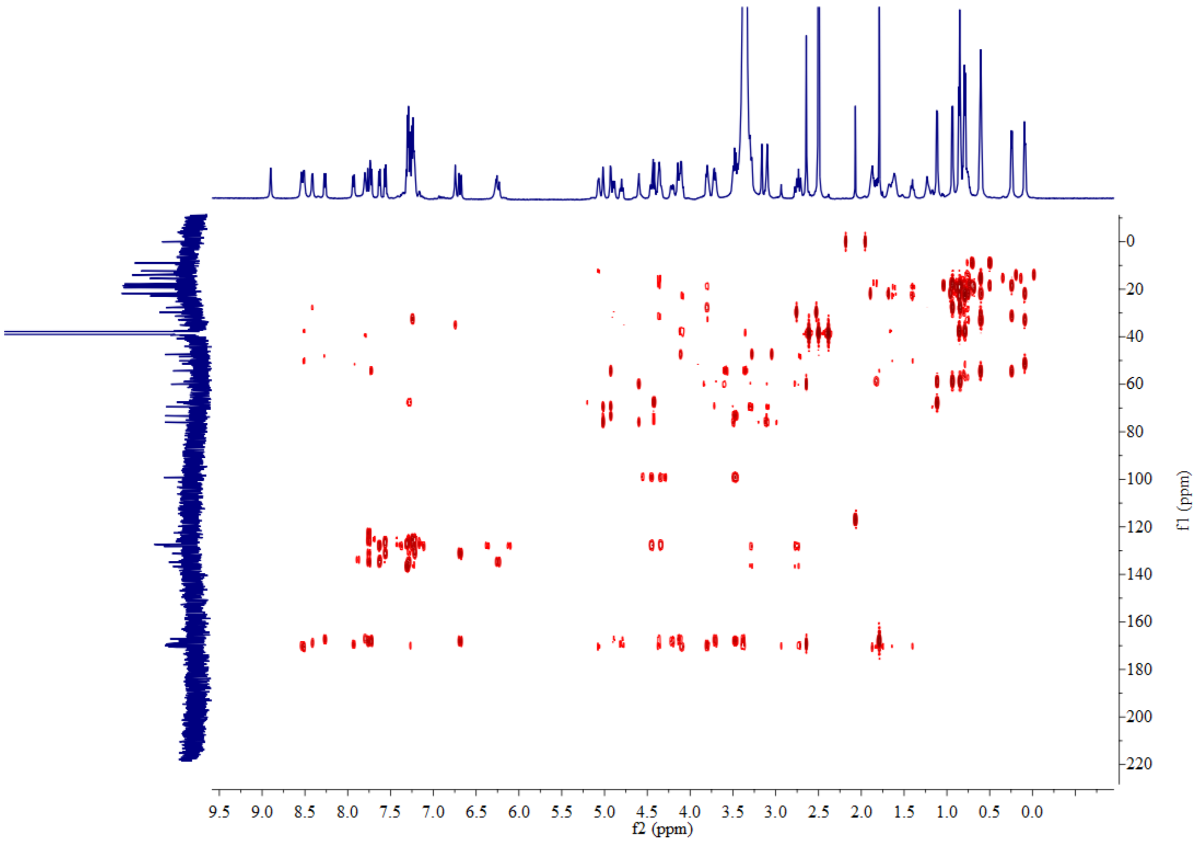

Figure S40. HMBC spectrum of **4** in DMSO-d_6.

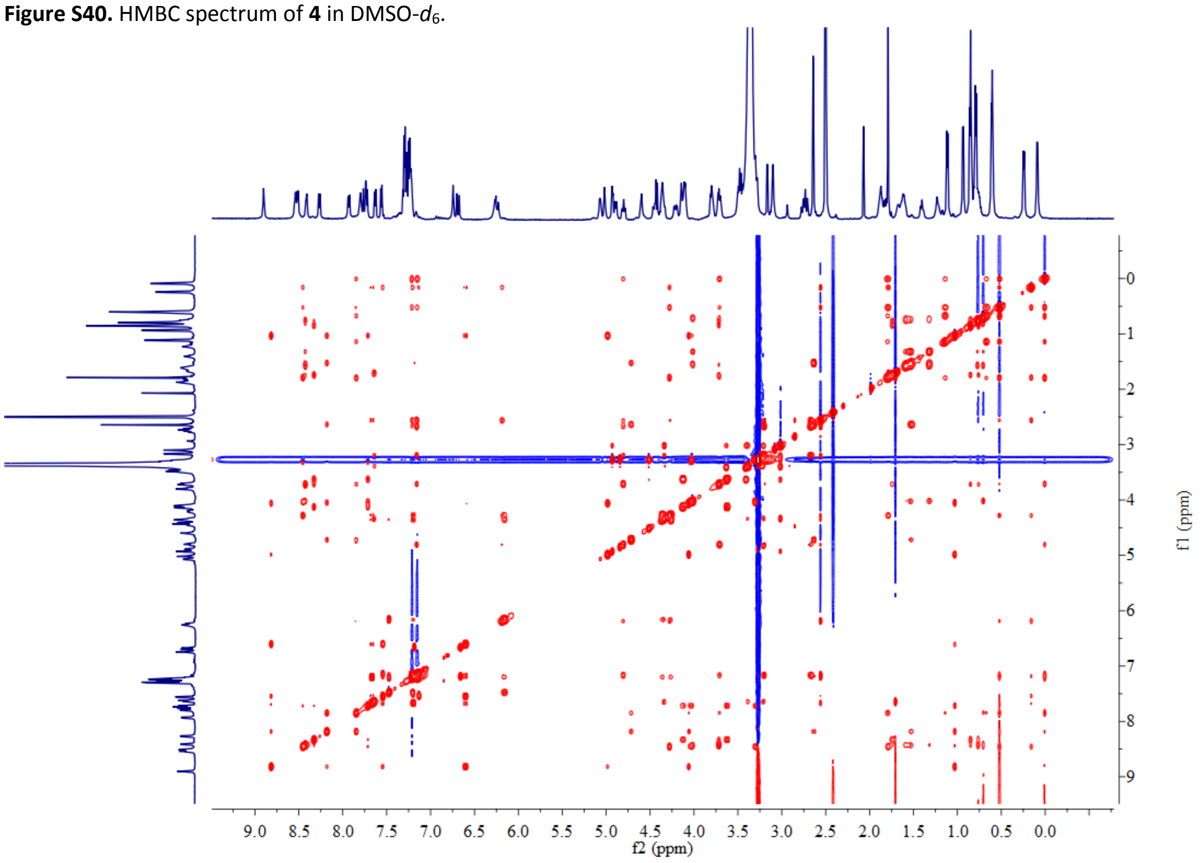

Figure S41. NOESY spectrum of **4** in DMSO-d_6.

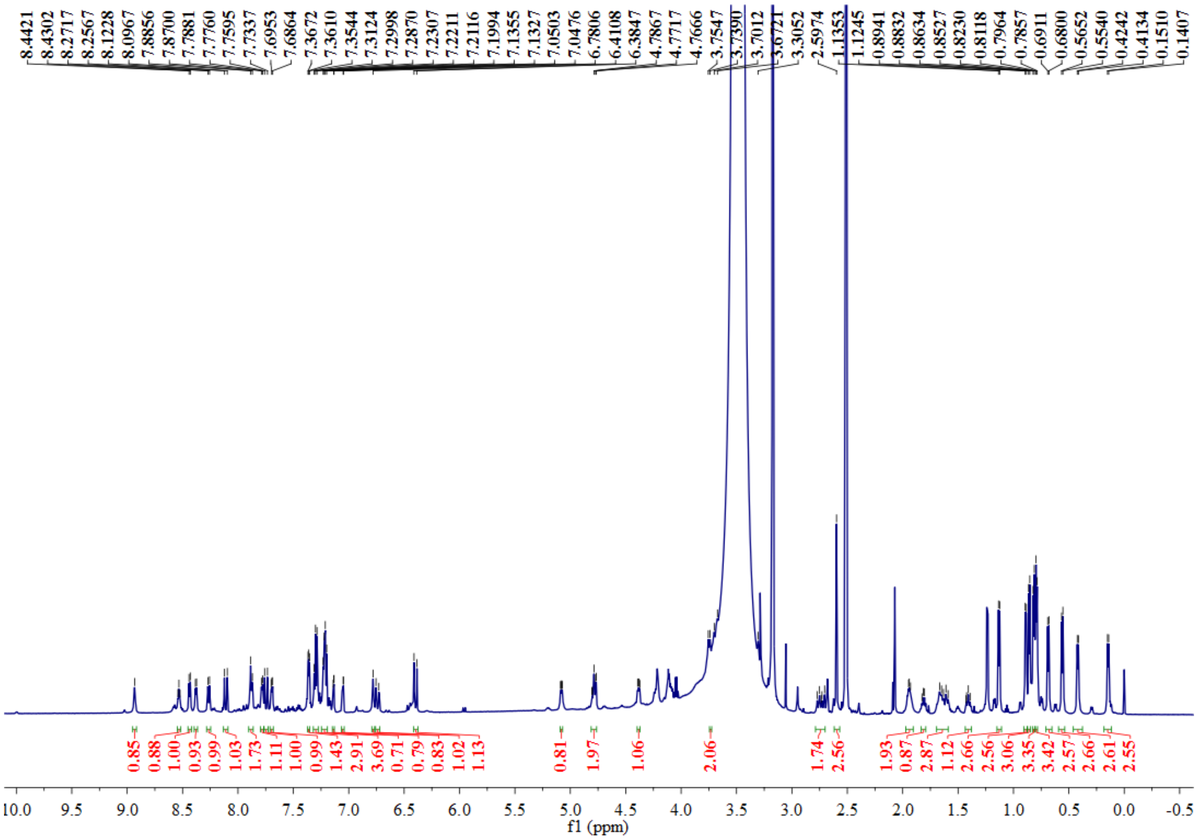

Figure S42. ^{1}H NMR spectrum of **5** in DMSO-d_6 at 600 MHz.

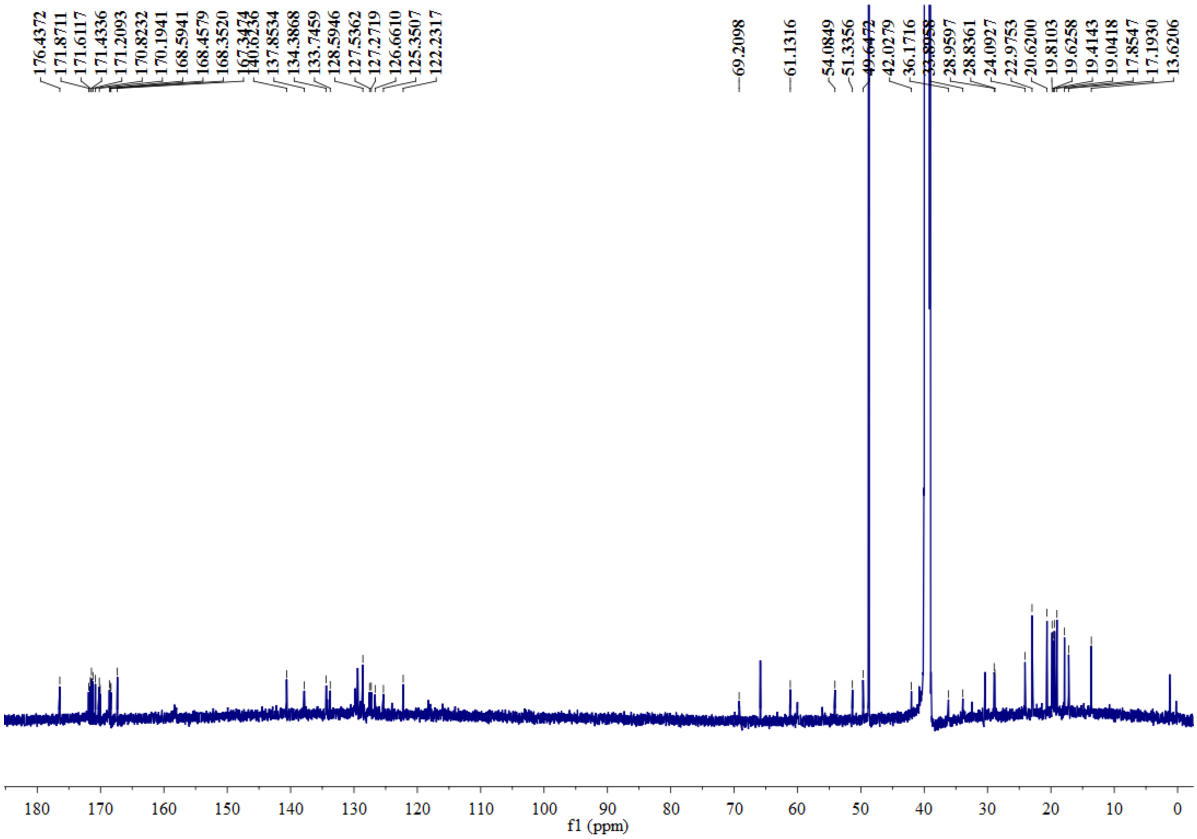

Figure S42. ^{13}C NMR spectrum of **5** in DMSO-d_6 at 150 MHz.

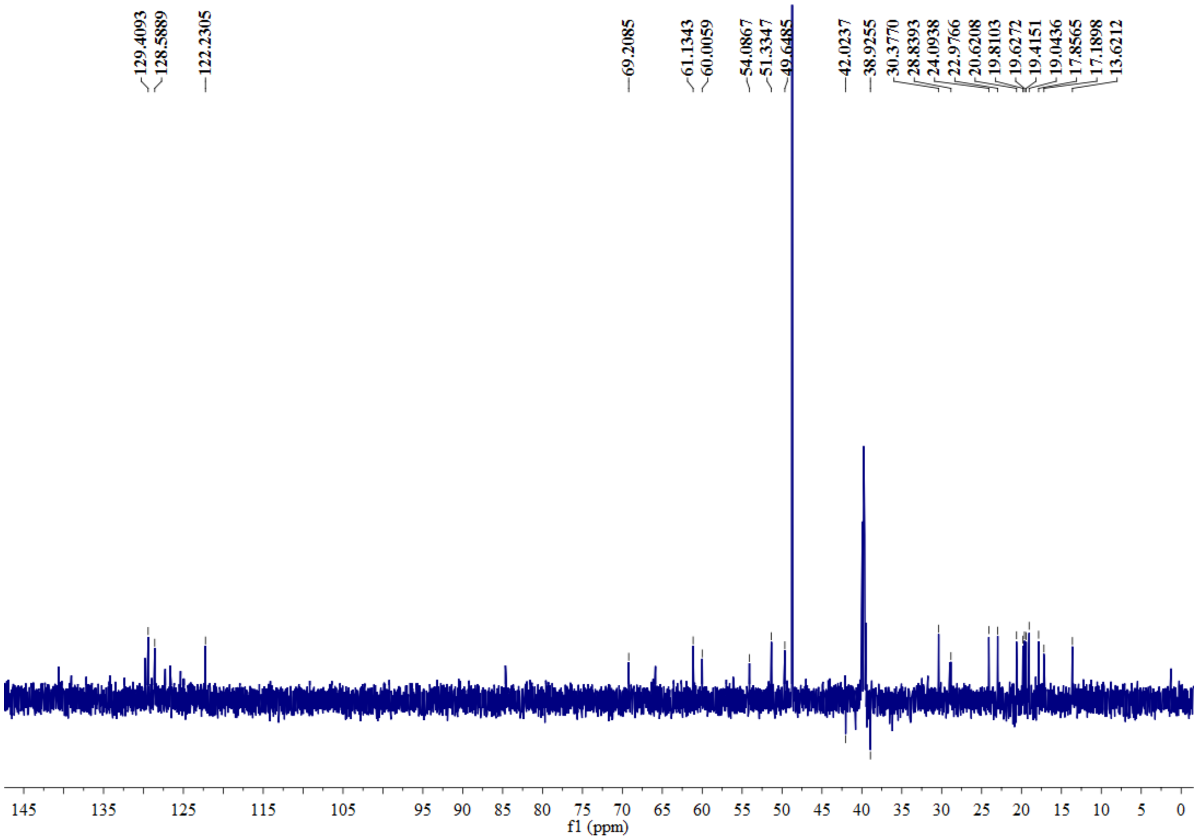

Figure S43. DEPT spectrum of **5** in DMSO-d_6.

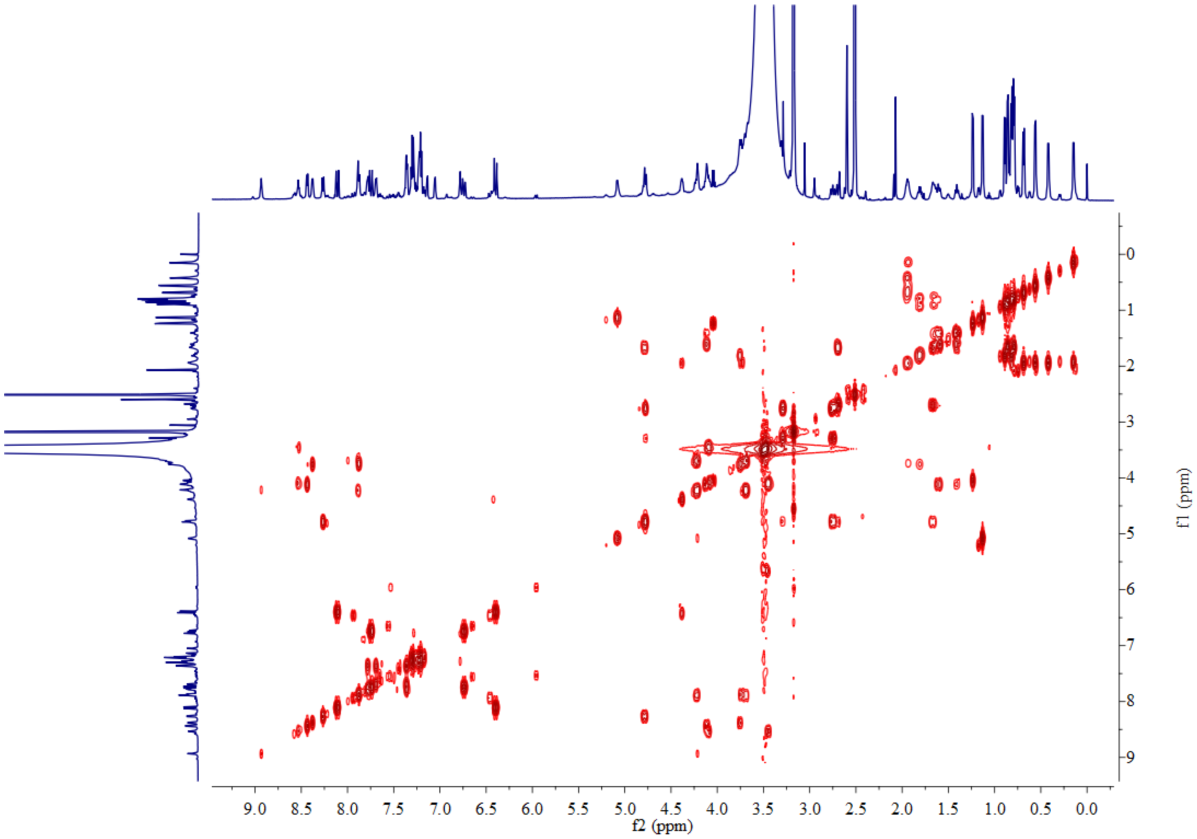

Figure S44. ^1H-^1H COSY spectrum of **5** in DMSO-d_6.

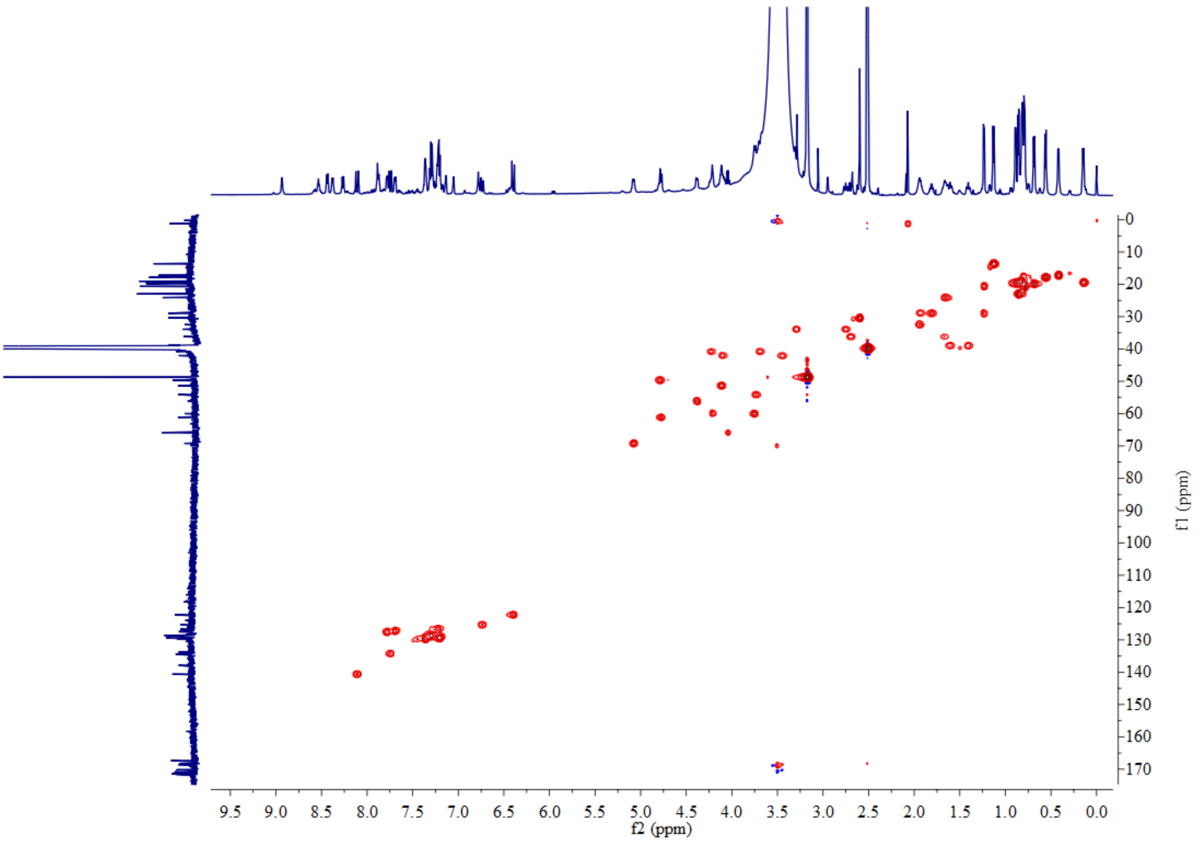

Figure S45. HSQC spectrum of **5** in DMSO-d_6.

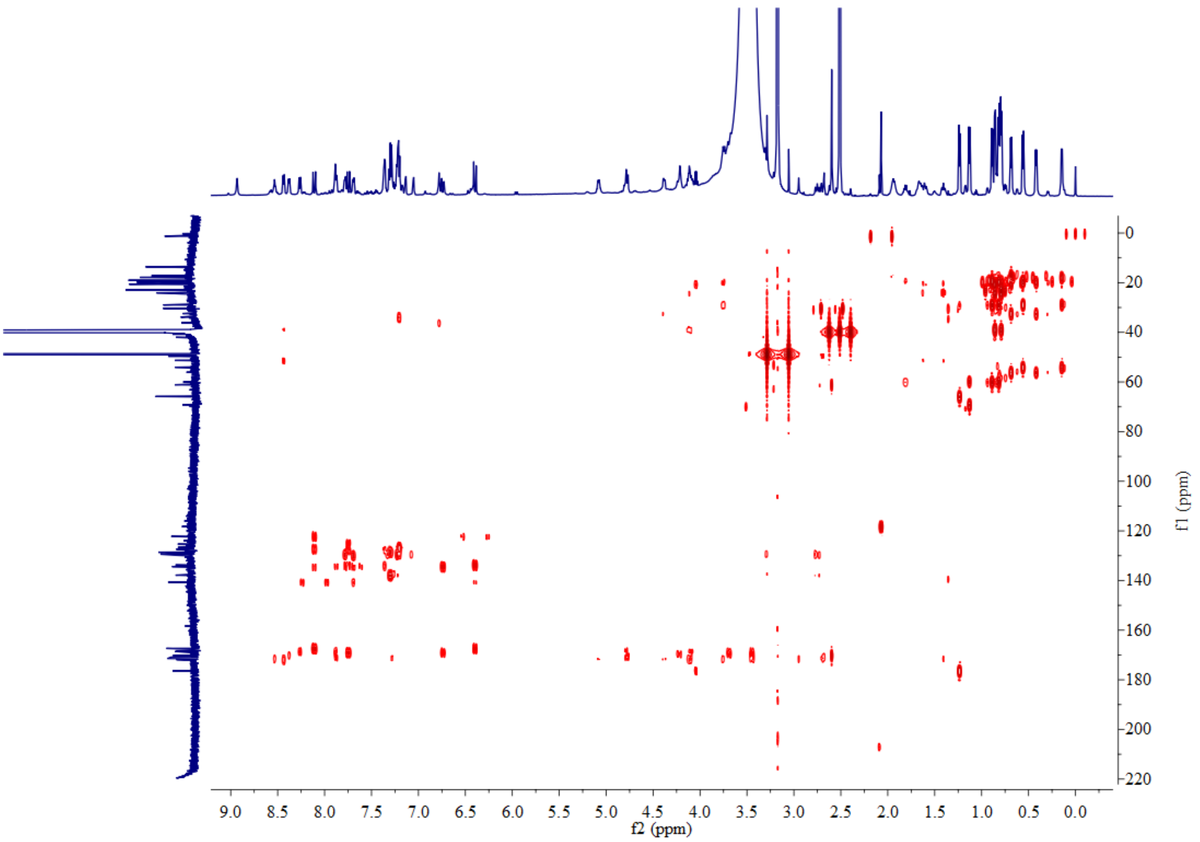

Figure S46. HMBC spectrum of **5** in DMSO-d_6.

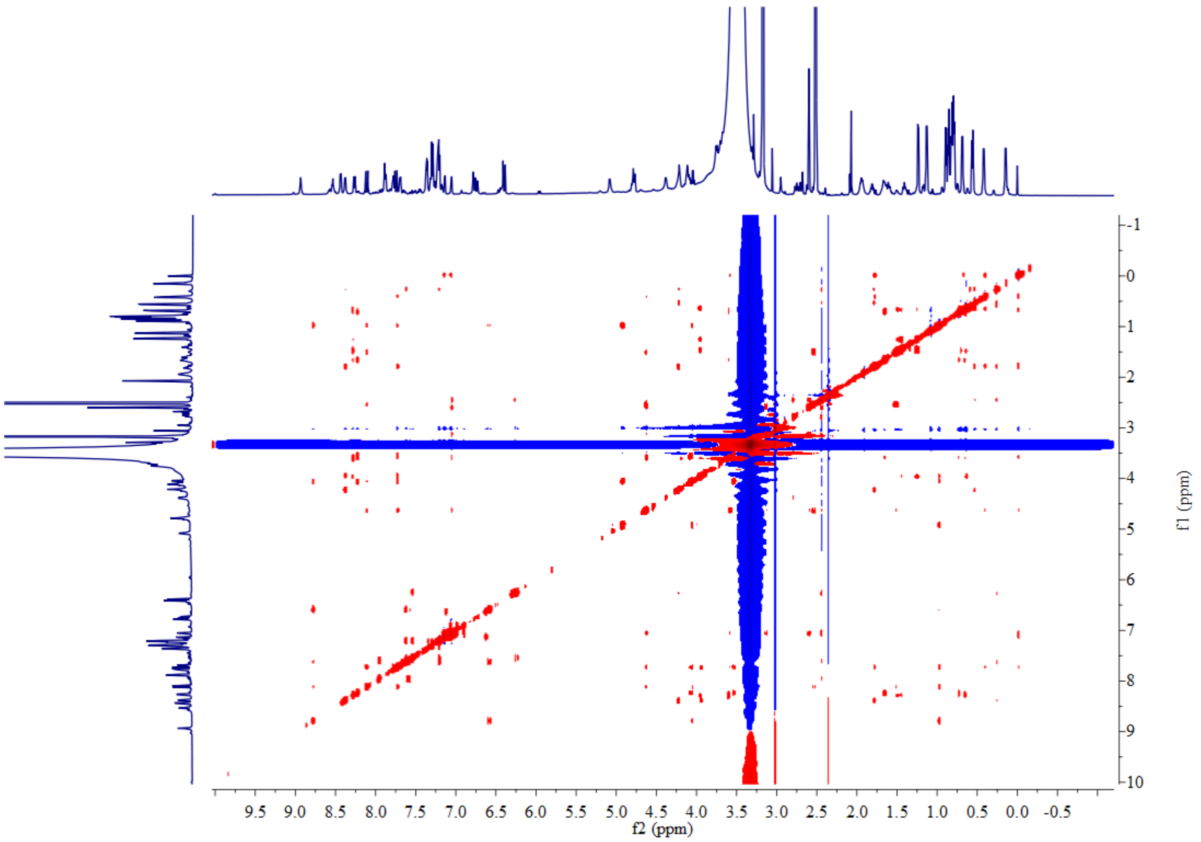

Figure S47. NOESY spectrum of **5** in DMSO-d_6.

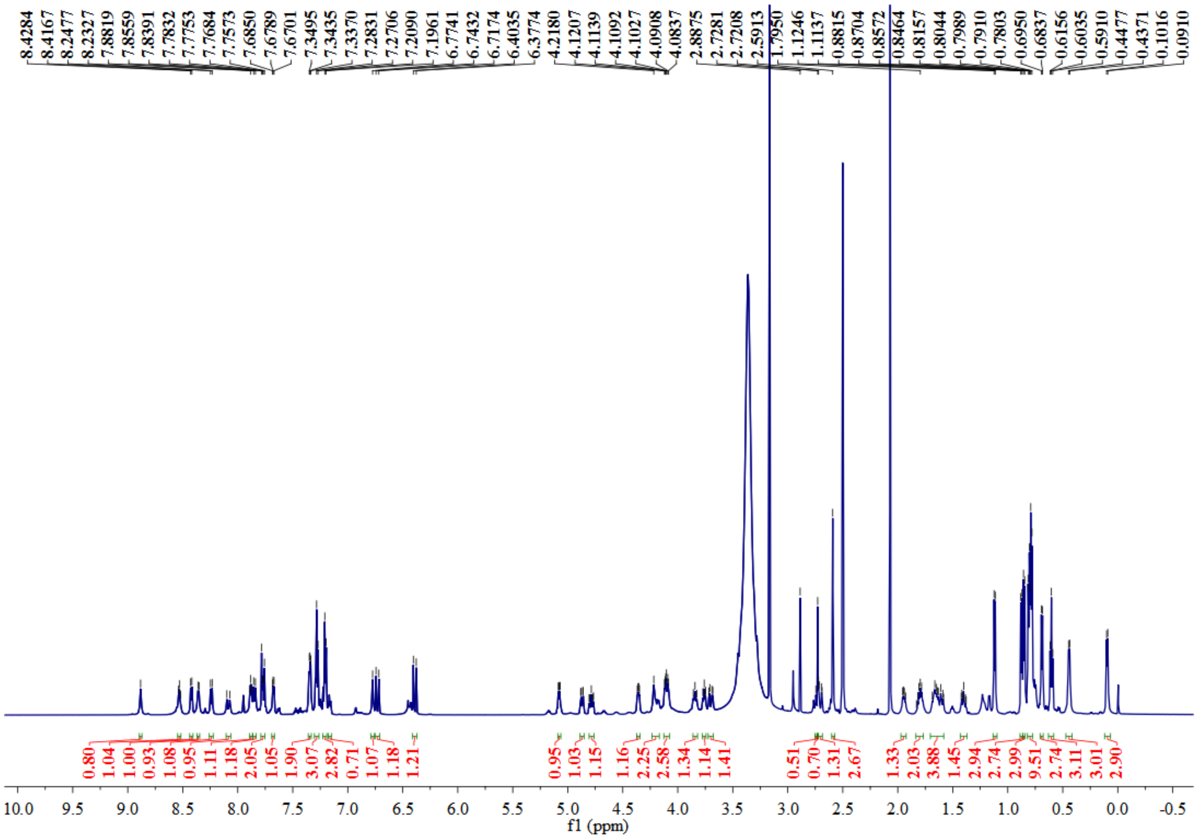

Figure S48. ^{1}H NMR spectrum of **6** in DMSO-d_6 at 600 MHz.

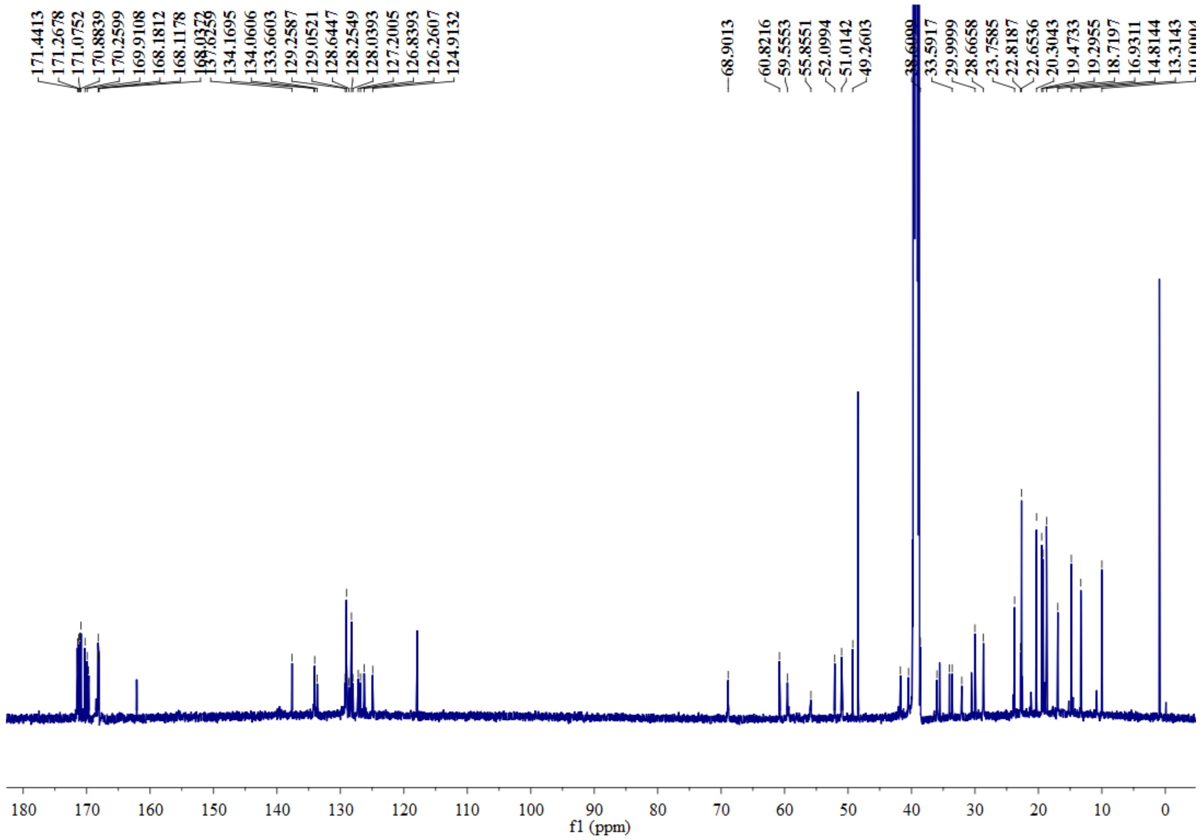

Figure S49. ^{13}C NMR spectrum of **6** in DMSO-d_6 at 150 MHz.

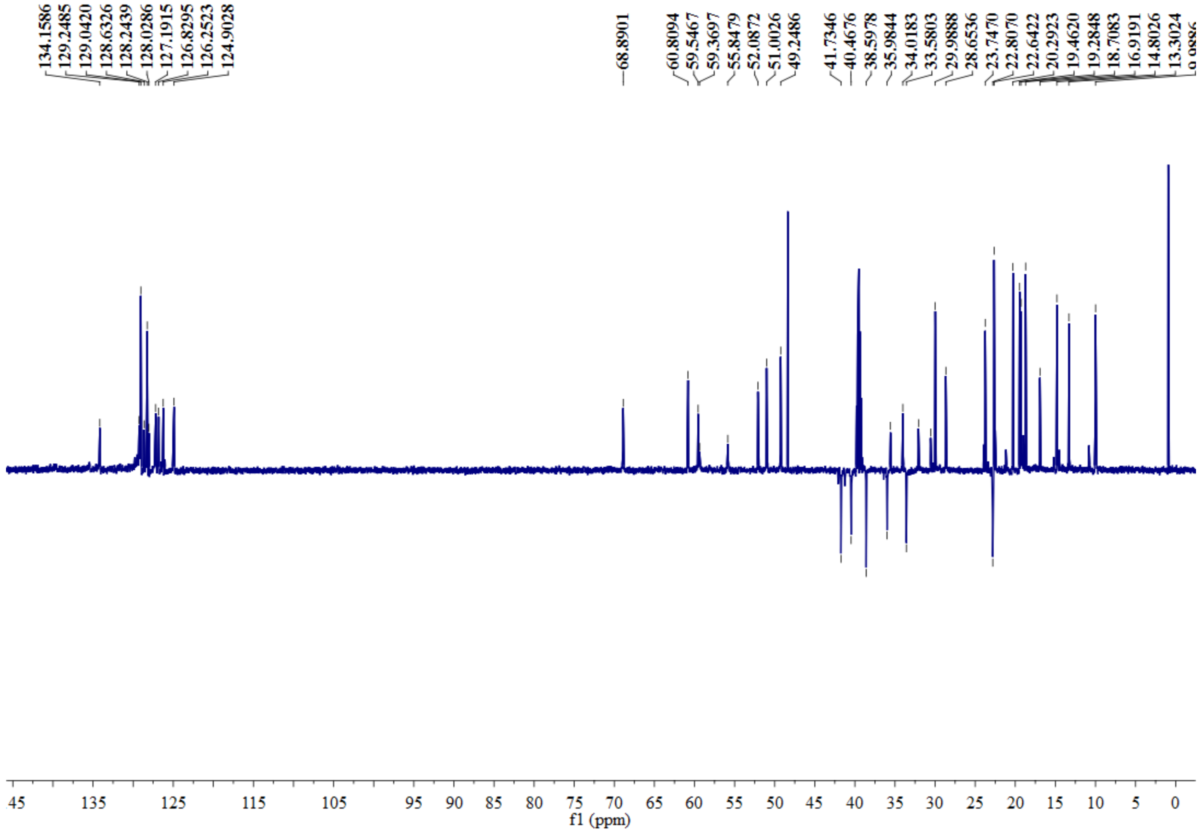

Figure S50. DEPT spectrum of **6** in DMSO-d_6.

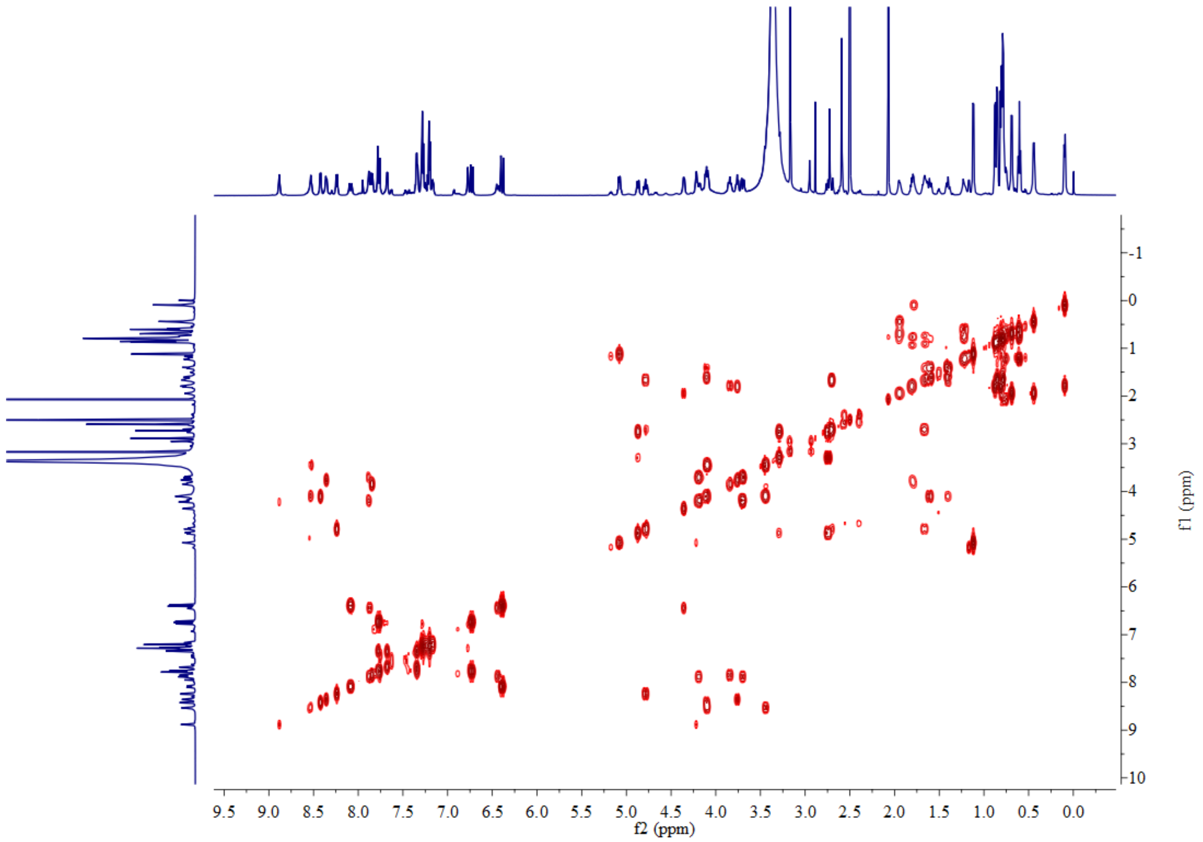

Figure S51. ^{1}H-^{1}H COSY spectrum of **6** in DMSO-d_6.

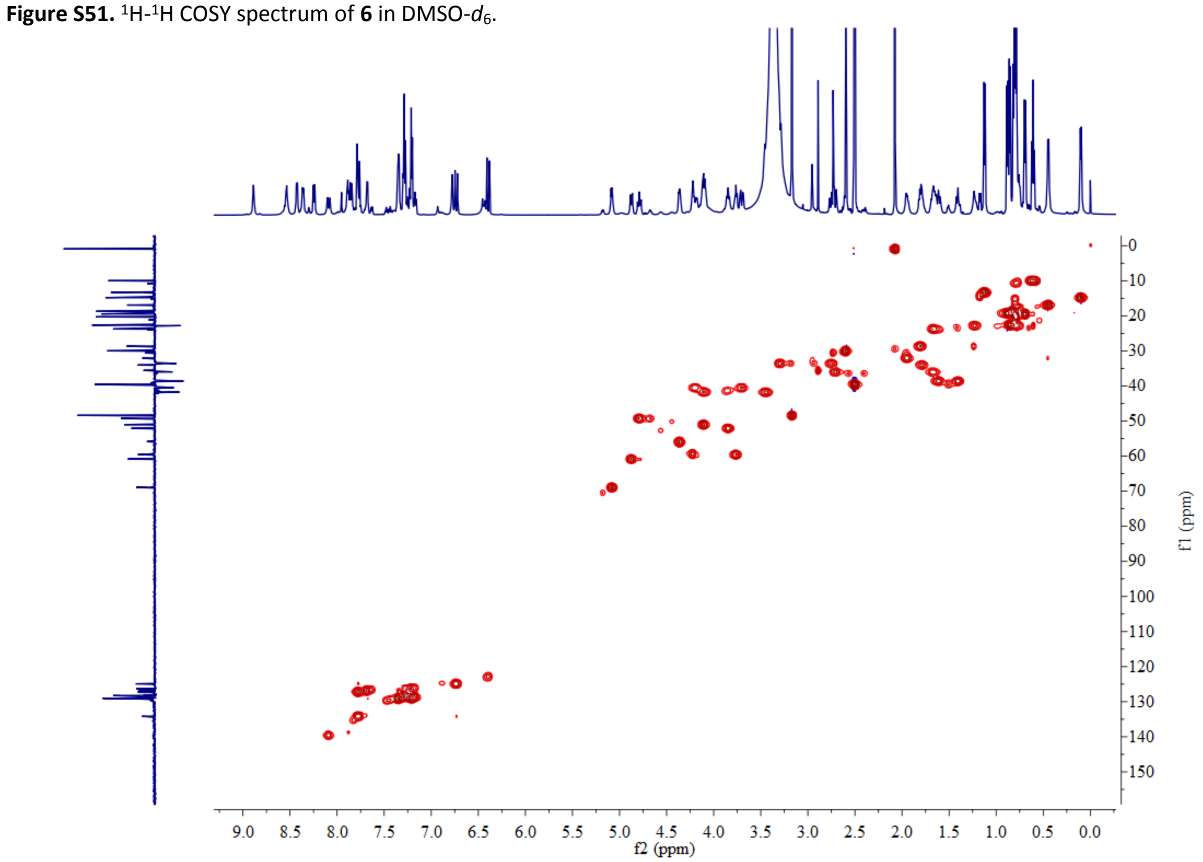

Figure S52. HSQC spectrum of **6** in DMSO-d_6.

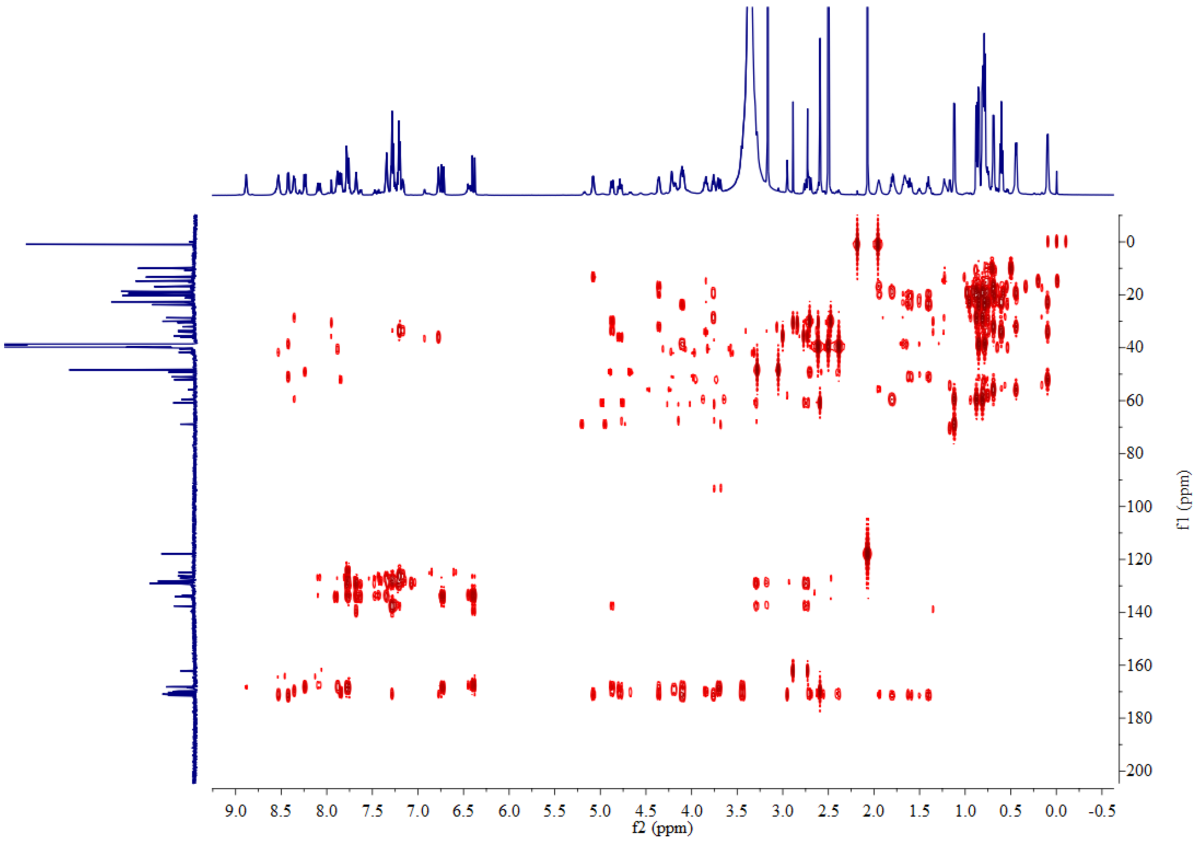

Figure S53. HMBC spectrum of **6** in DMSO-d_6.

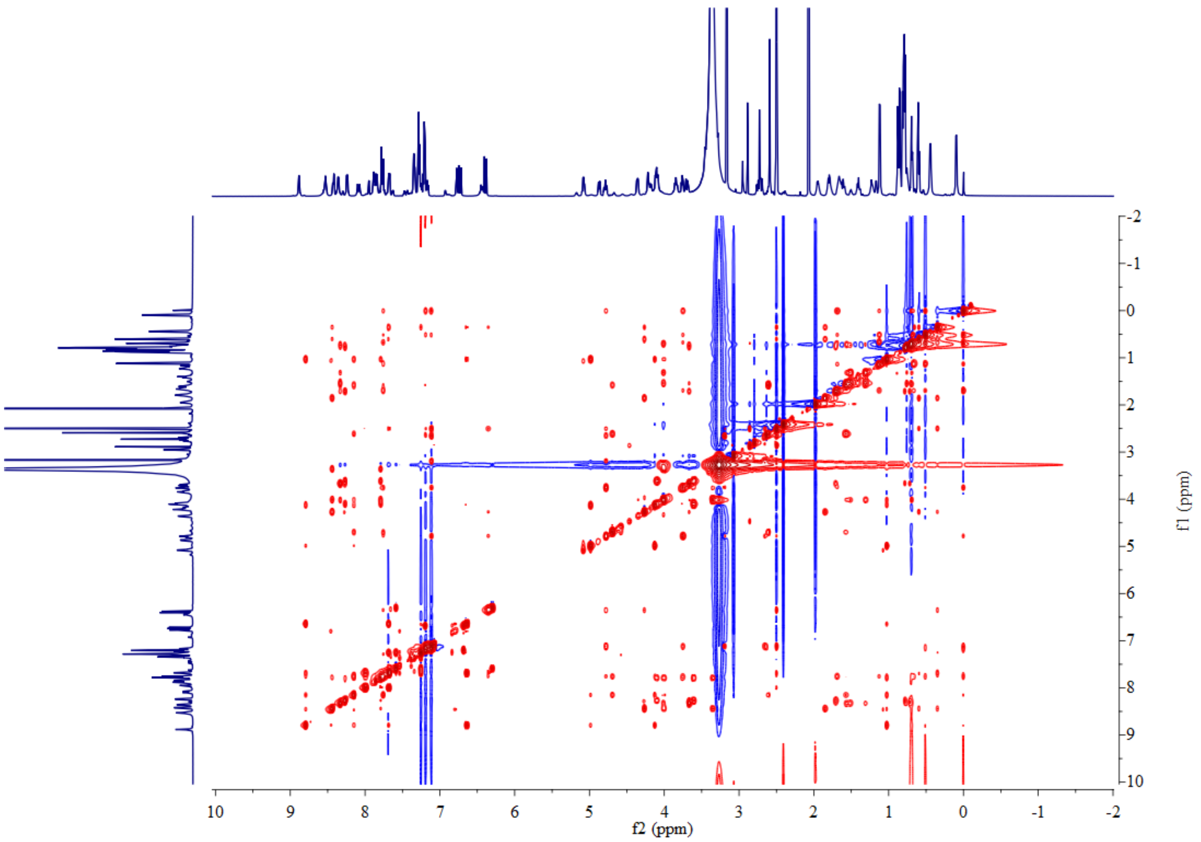

Figure S54. NOESY spectrum of **6** in DMSO-d_6.

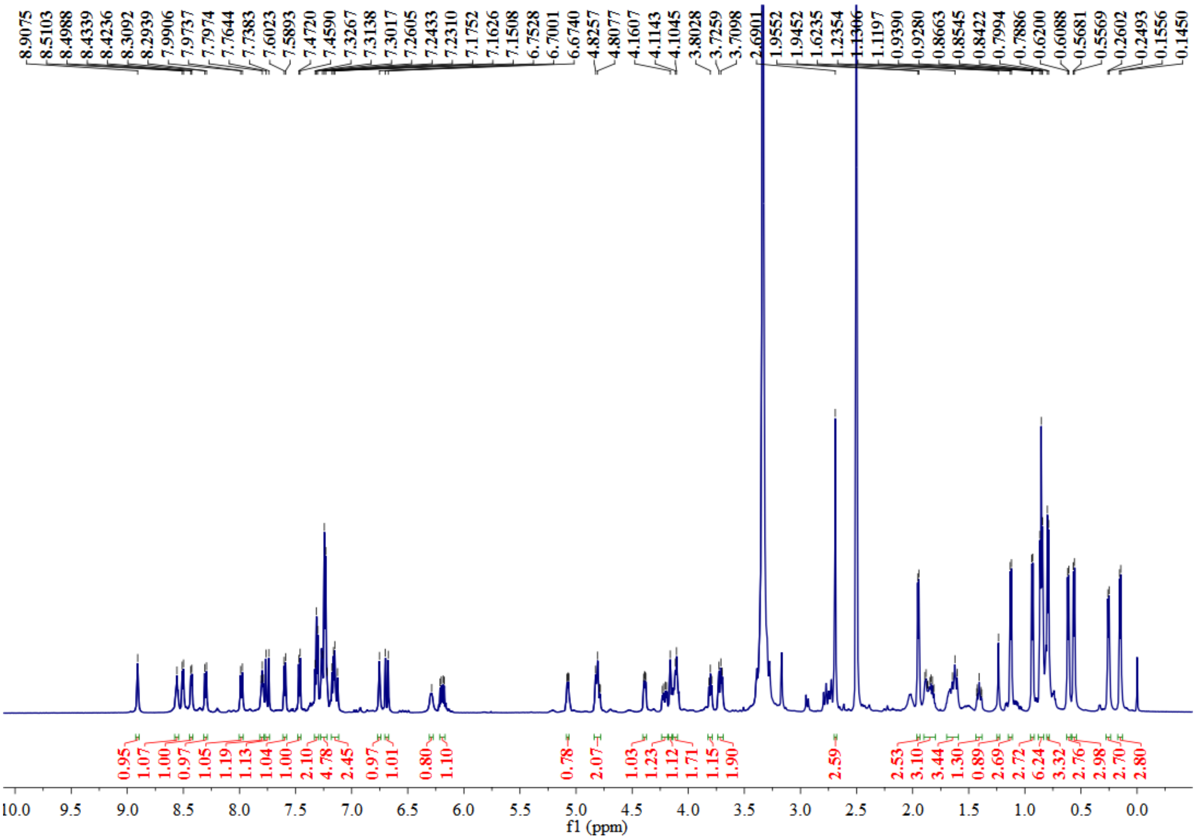

Figure S55. ^{1}H NMR spectrum of **7** in DMSO-d_6 at 600 MHz.

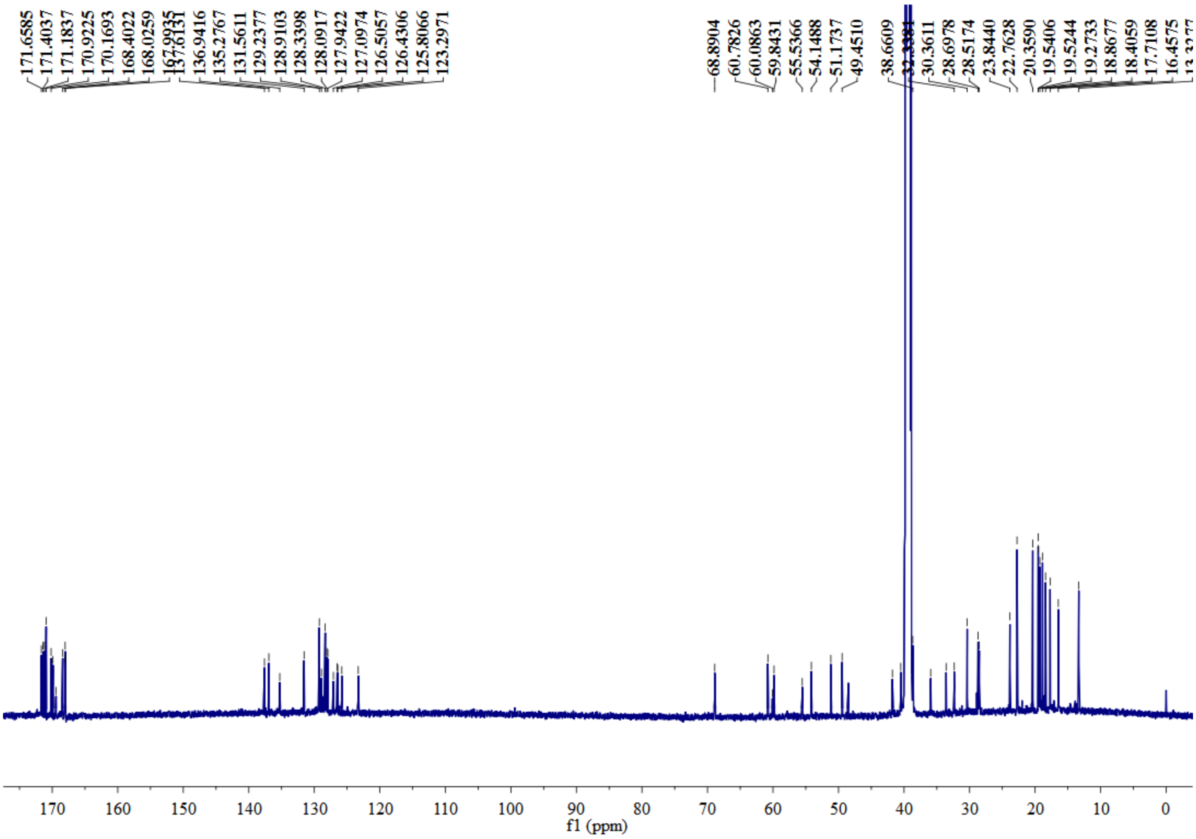

Figure S56. ^{13}C NMR spectrum of **7** in DMSO-d_6 at 150 MHz.

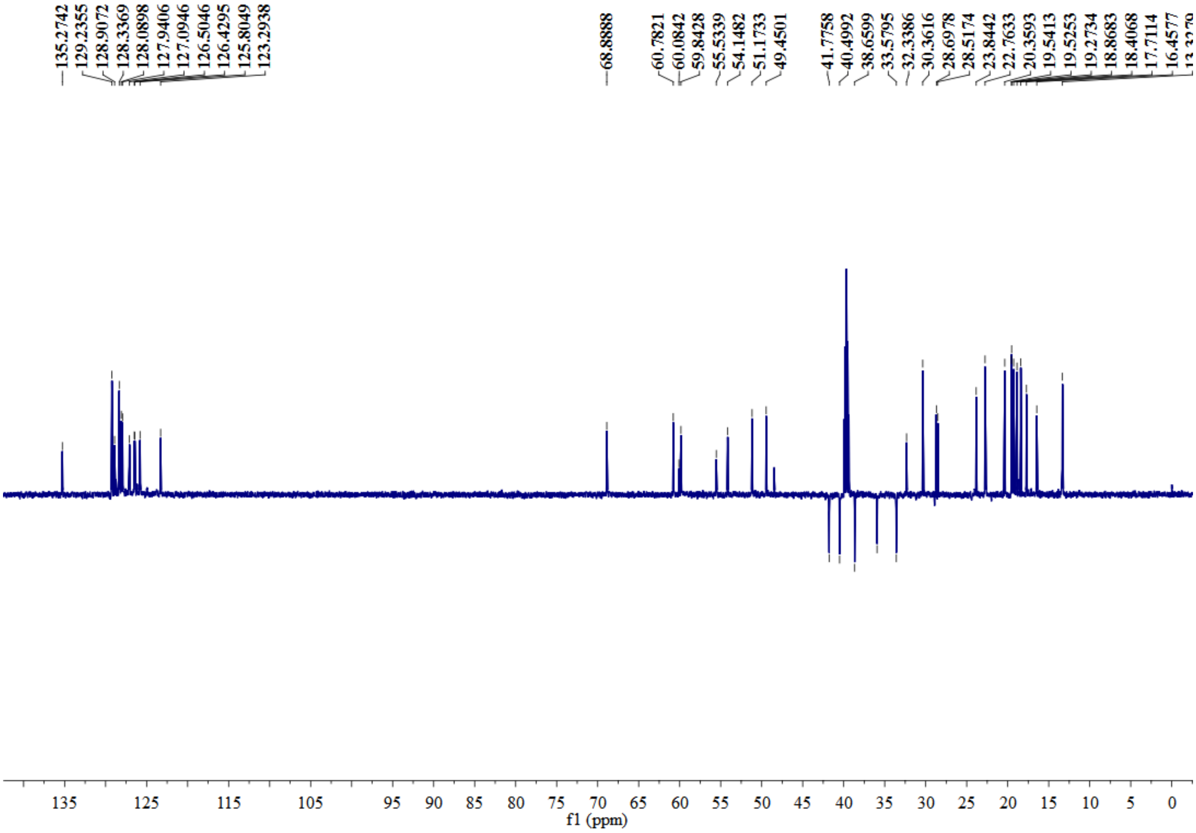

Figure S57. DEPT spectrum of **7** in DMSO-d_6.

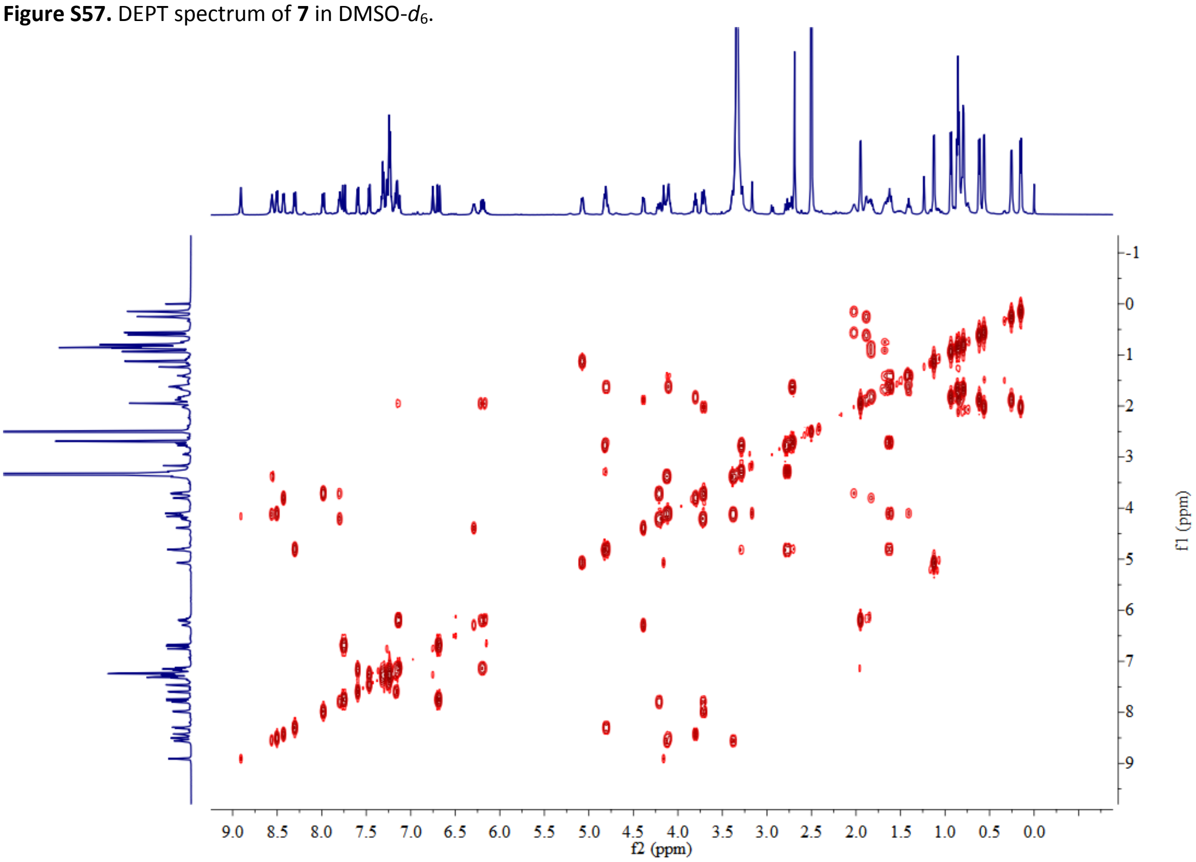

Figure S58. ^{1}H-^{1}H COSY spectrum of **7** in DMSO-d_6.

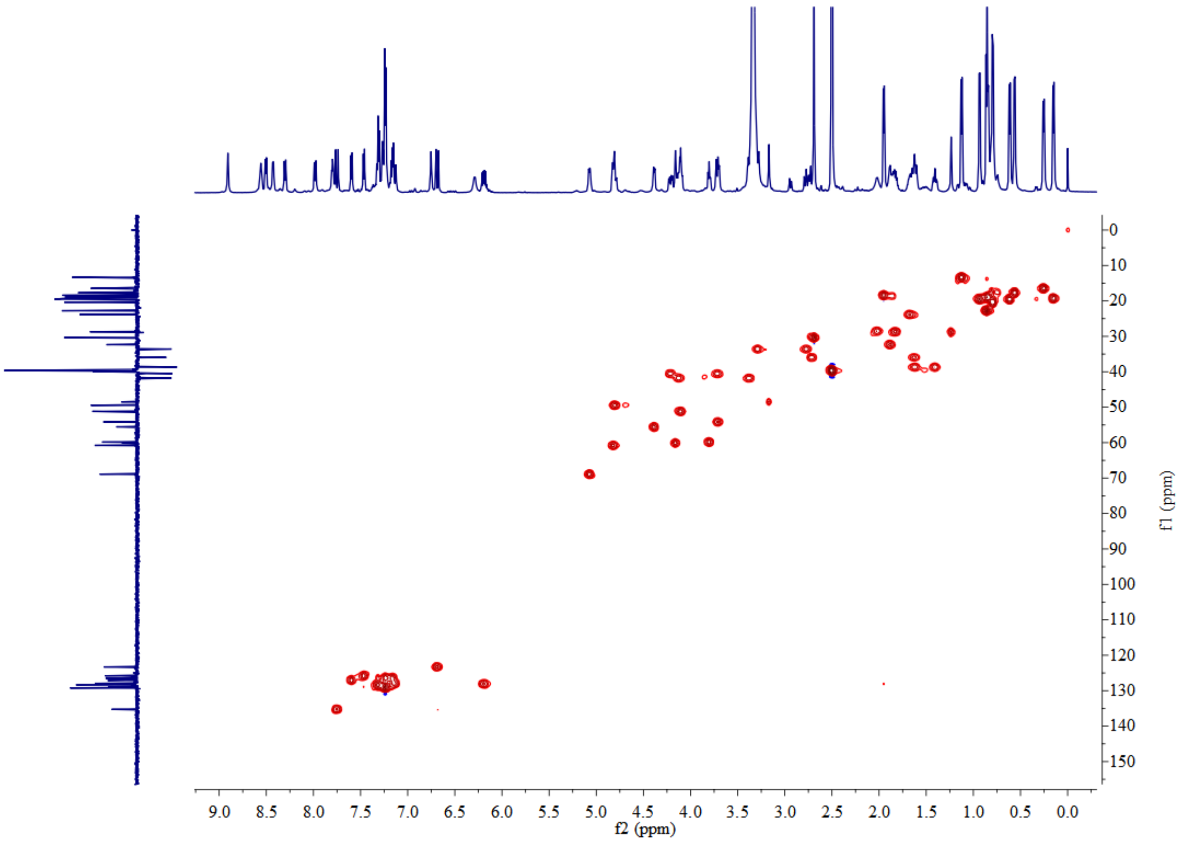

Figure S59. HSQC spectrum of **7** in DMSO-d_6.

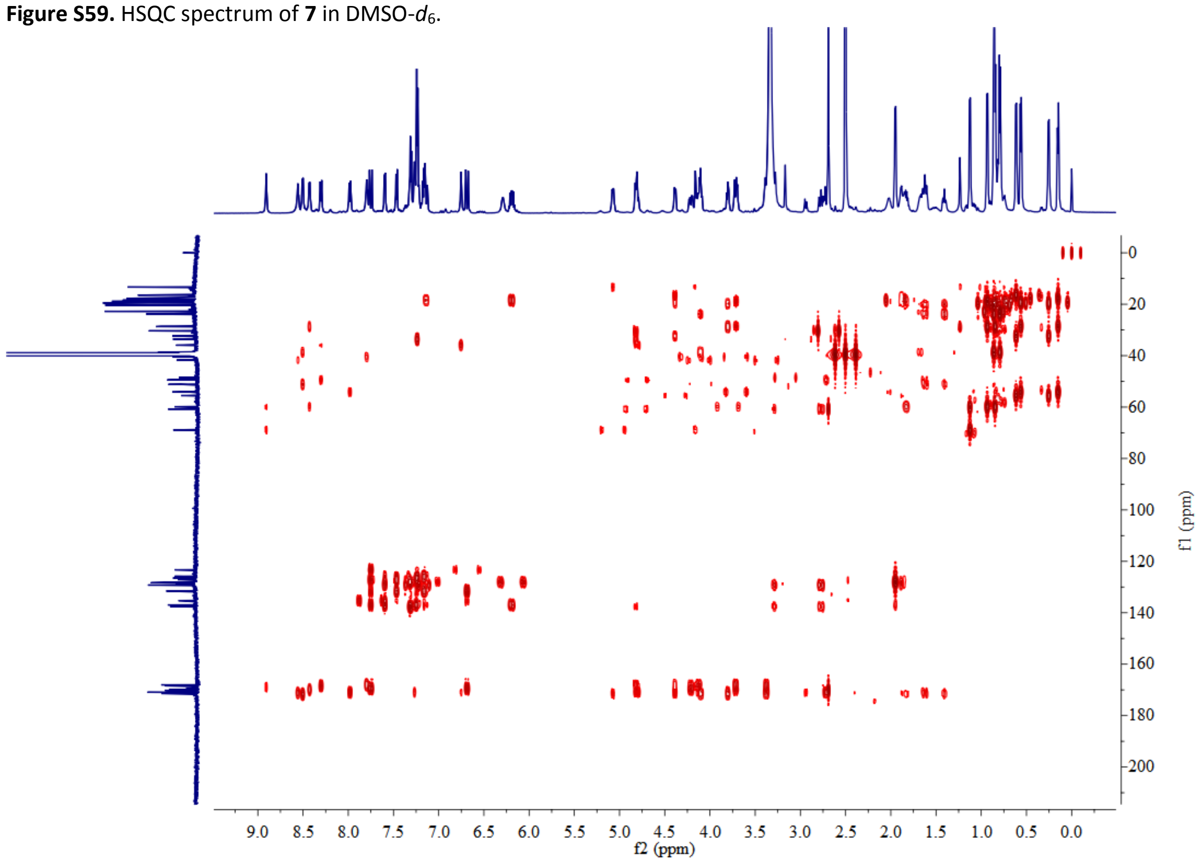

Figure S60. HMBC spectrum of **7** in DMSO-d_6.

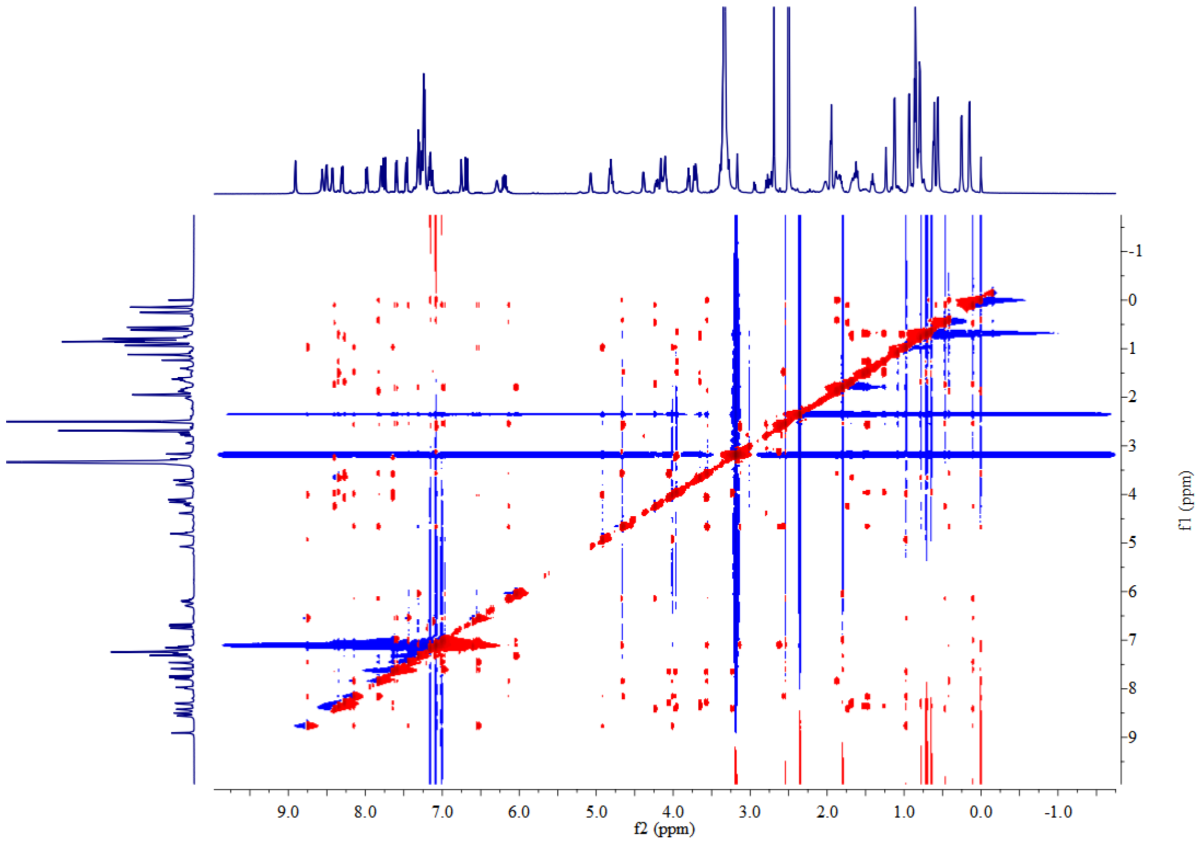

Figure S61. NOESY spectrum of **7** in DMSO-d_6.

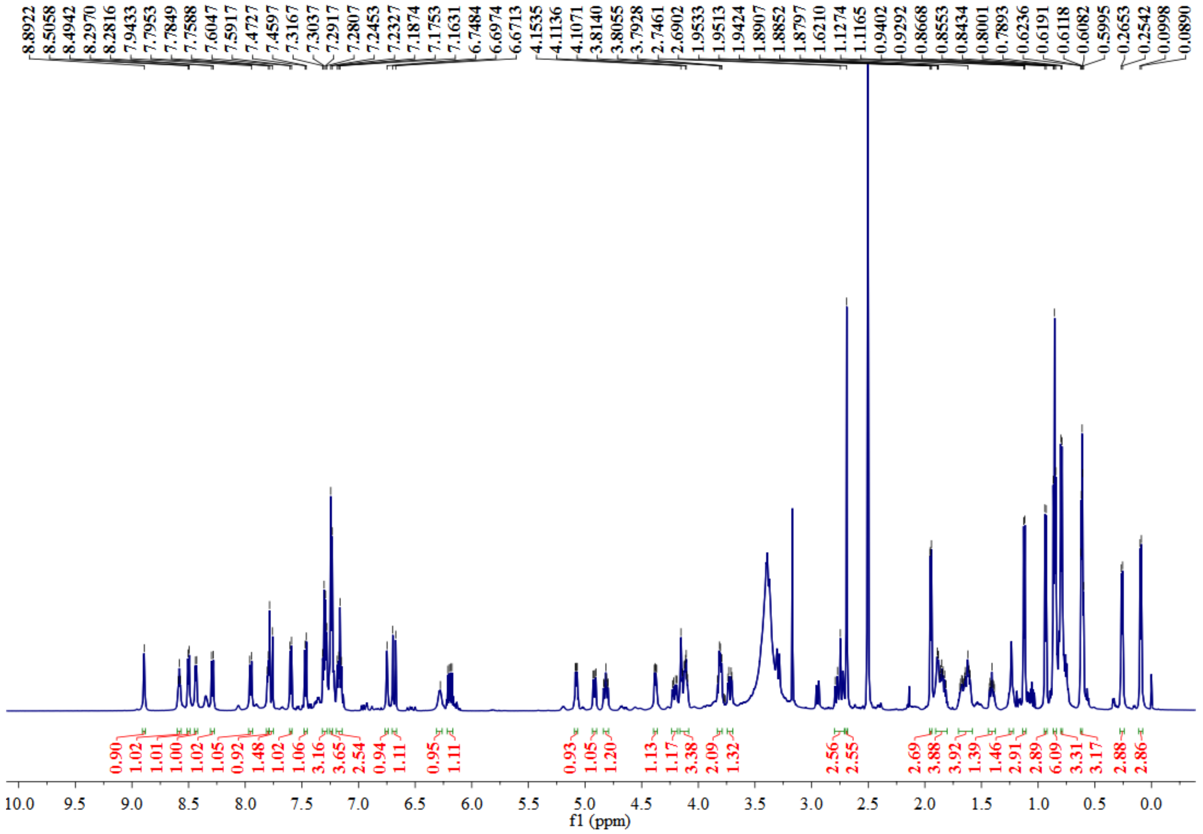

Figure S62. ^{1}H NMR spectrum of **8** in DMSO-d_6 at 600 MHz.

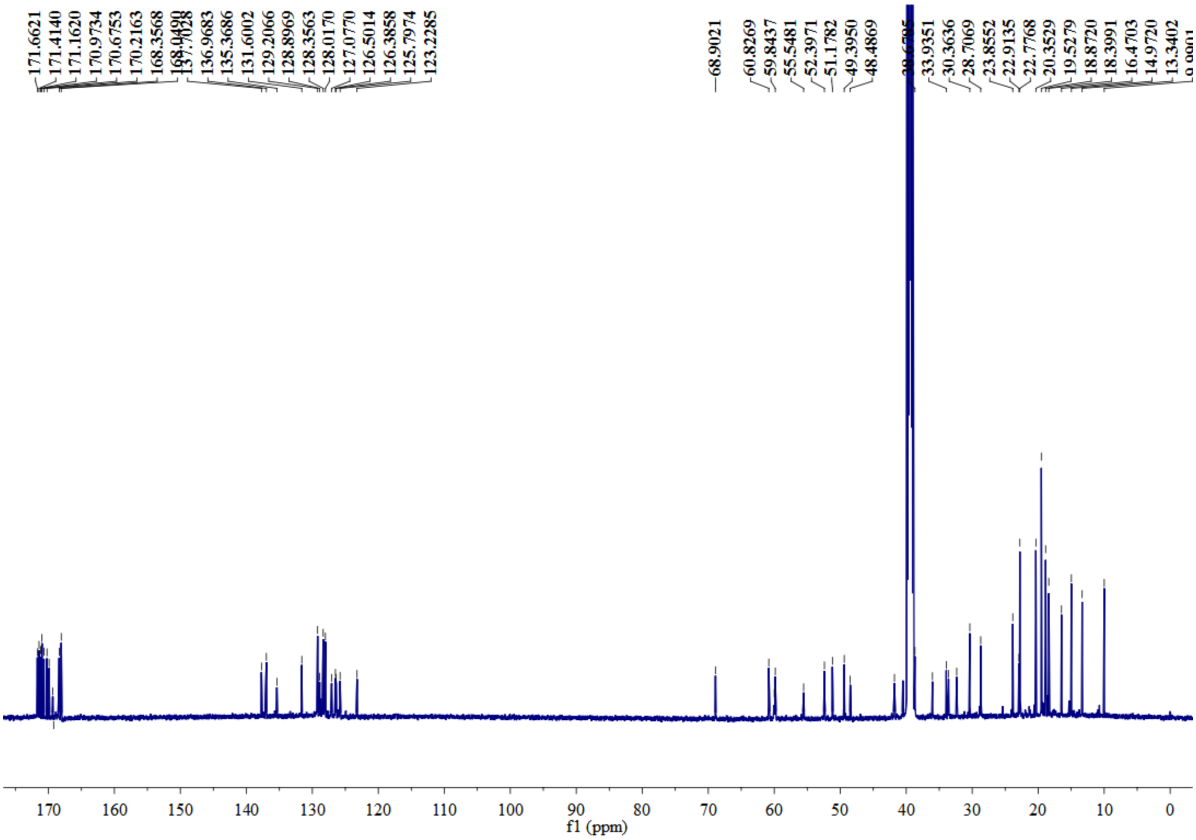

Figure S63. ^{13}C NMR spectrum of **8** in DMSO-d_6 at 150 MHz.

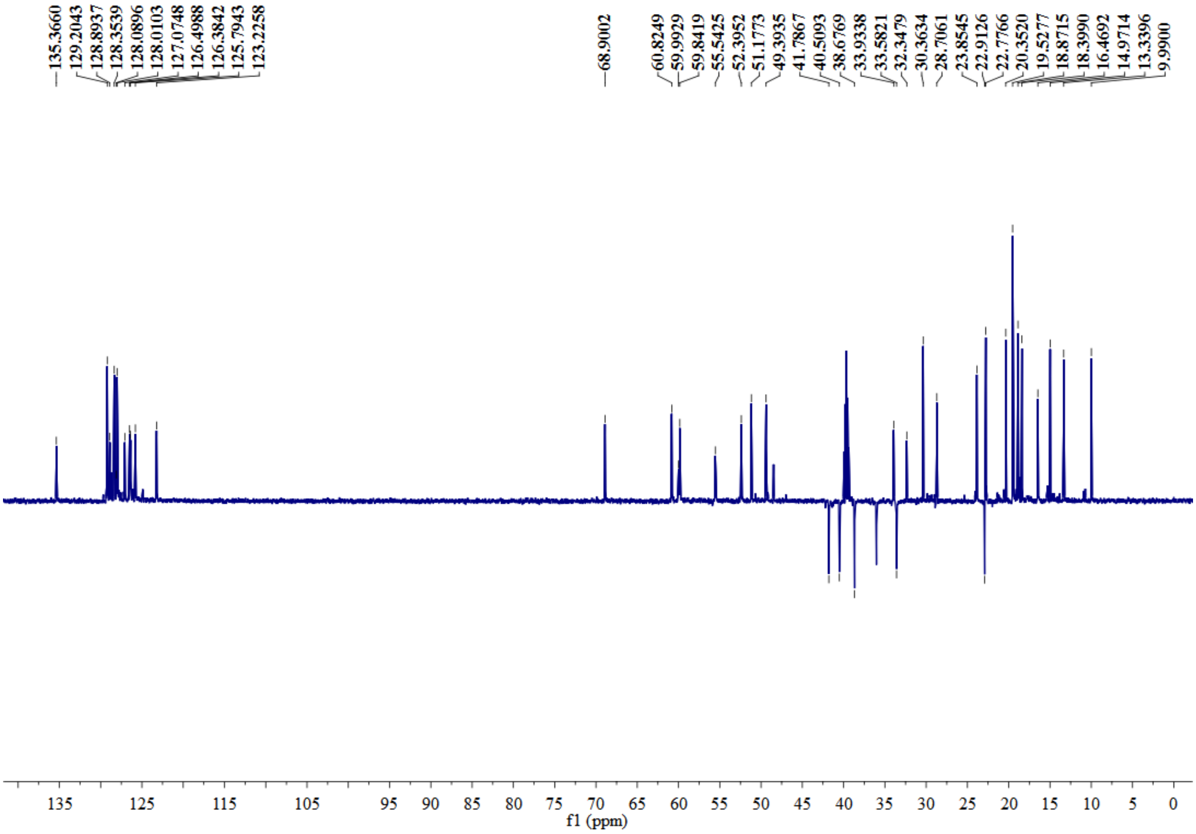

Figure S64. DEPT spectrum of **8** in DMSO-d_6.

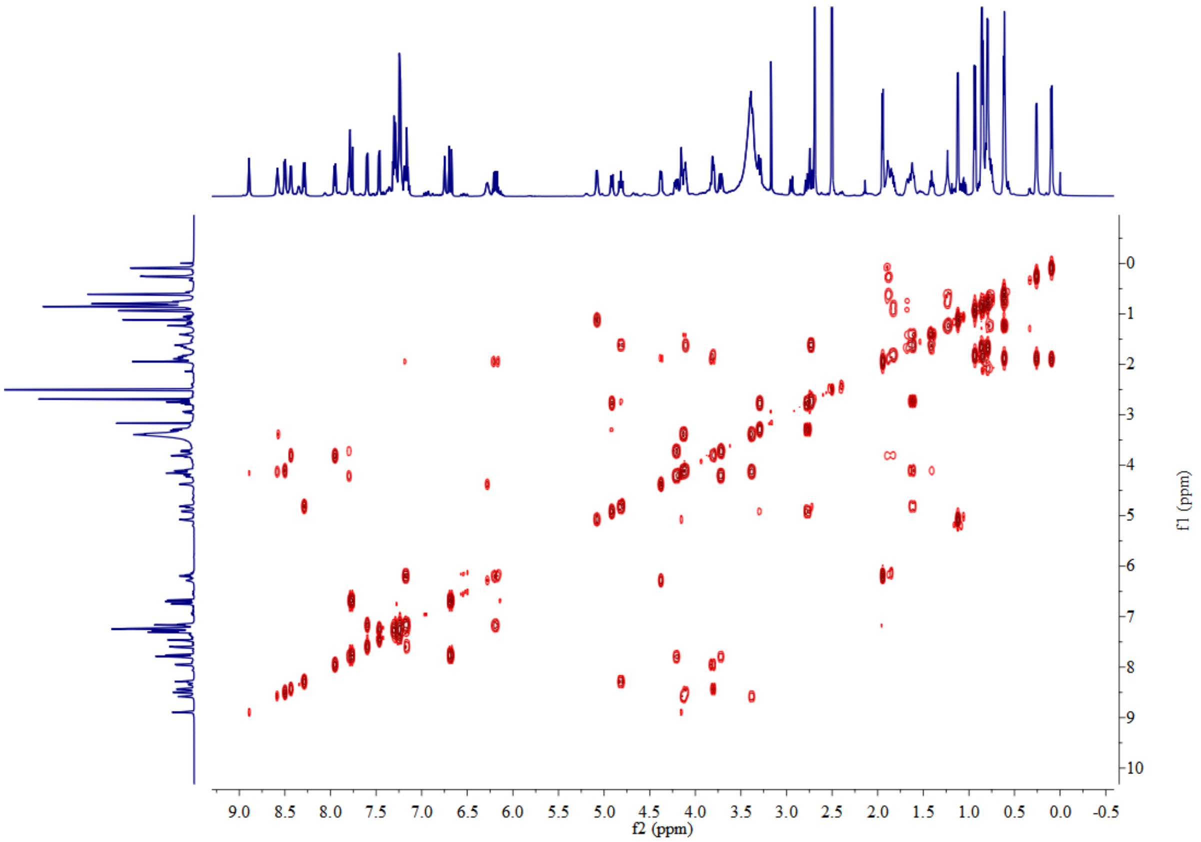

Figure S65. ^{1}H-^{1}H COSY spectrum of **8** in DMSO-d_6.

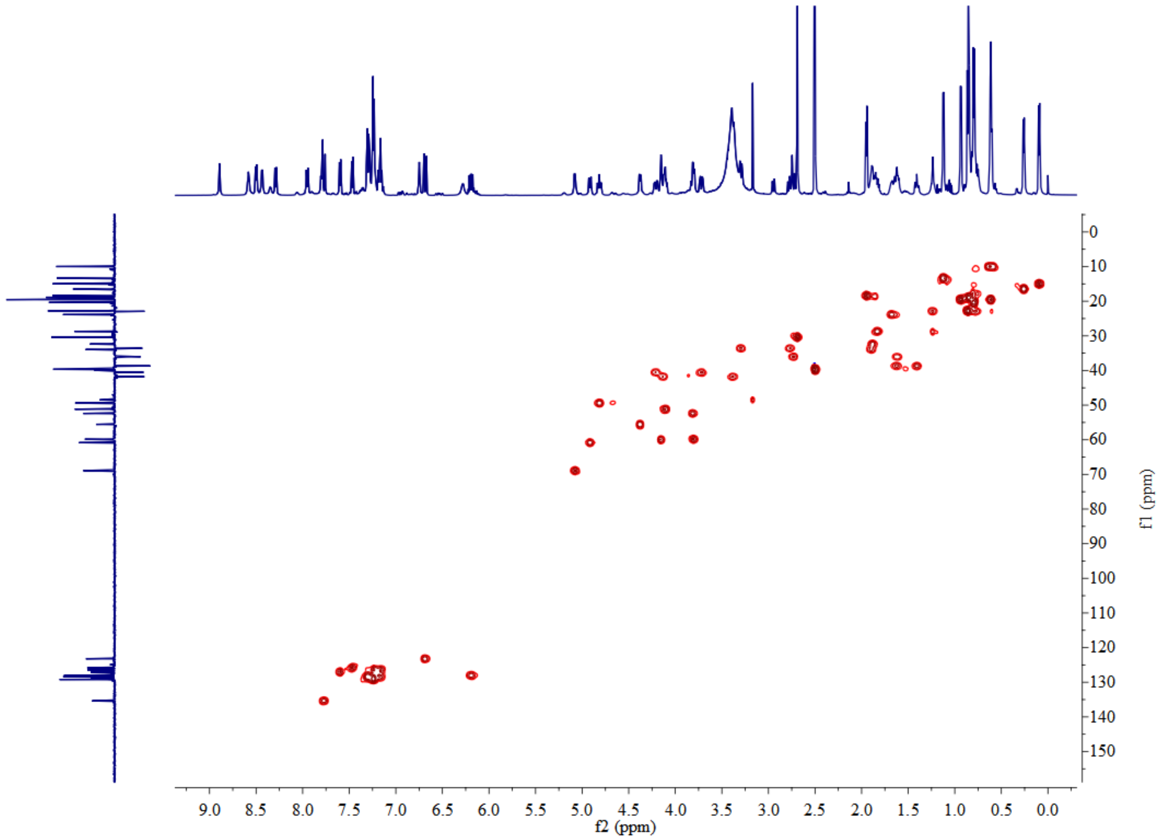

Figure S66. HSQC spectrum of **8** in DMSO-d_6.

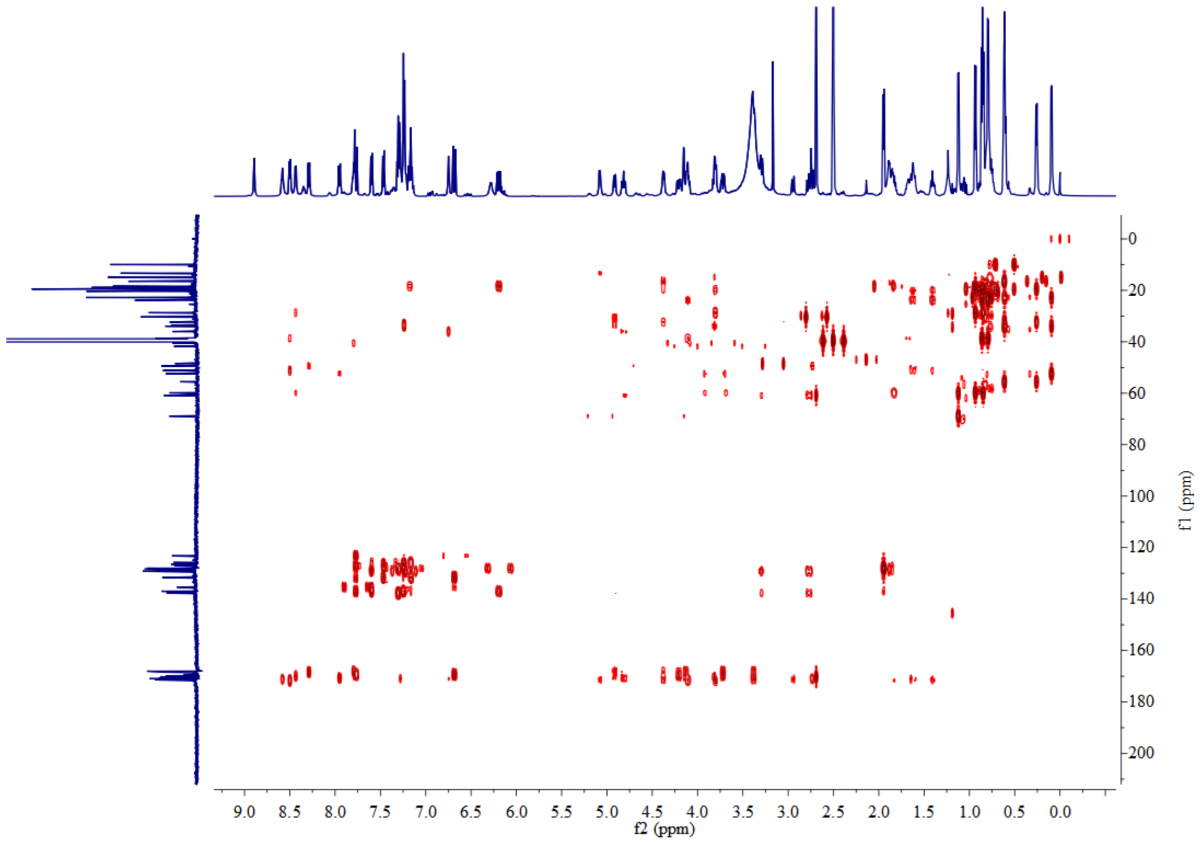

Figure S67. HMBC spectrum of **8** in DMSO-d_6.

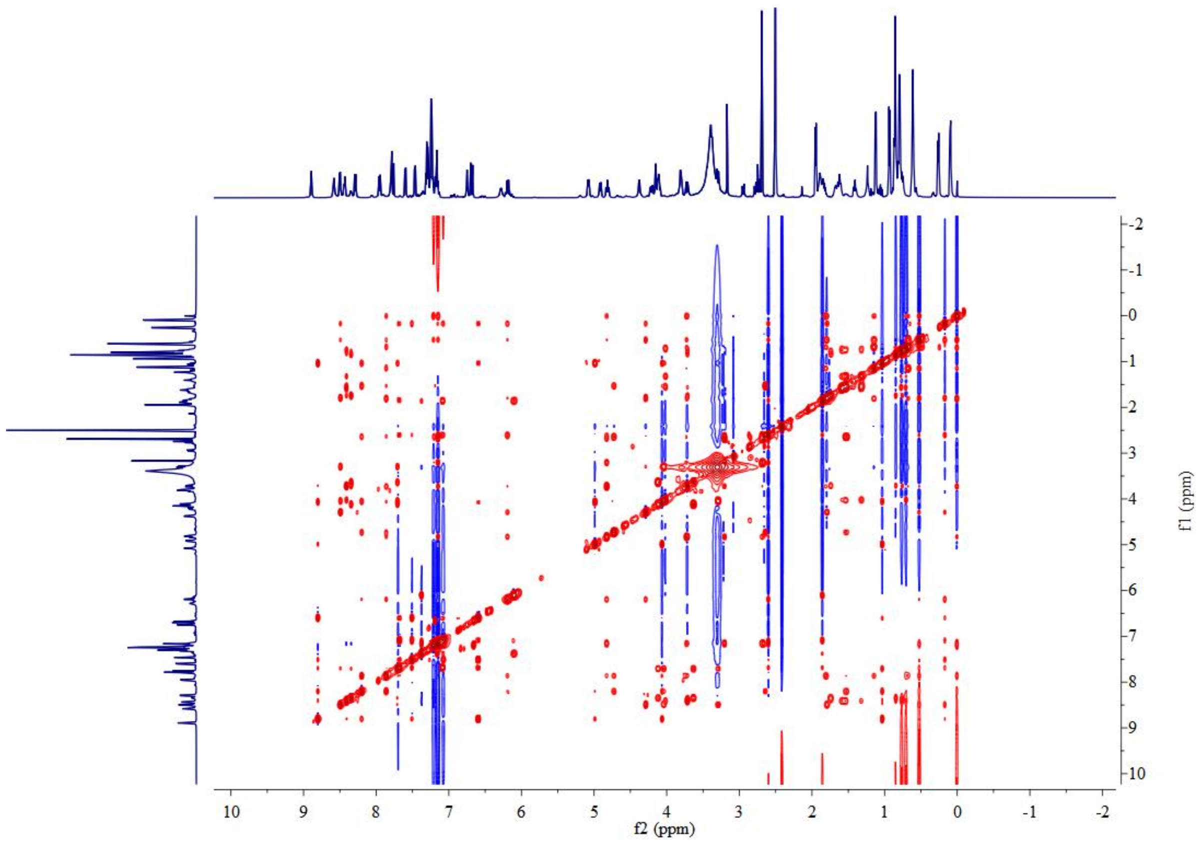

Figure S68. NOESY spectrum of **8** in DMSO-d_6.

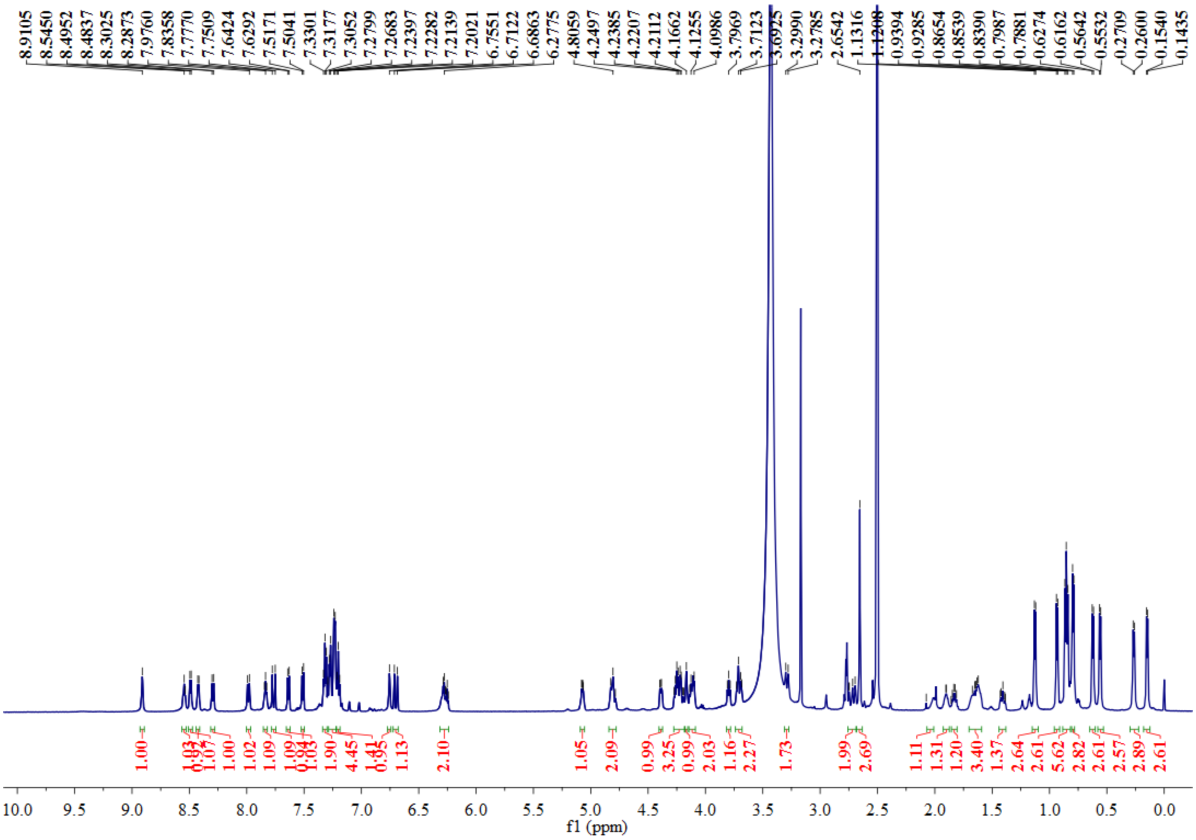

Figure S69. ^{1}H NMR spectrum of **9** in DMSO-d_6 at 600 MHz.

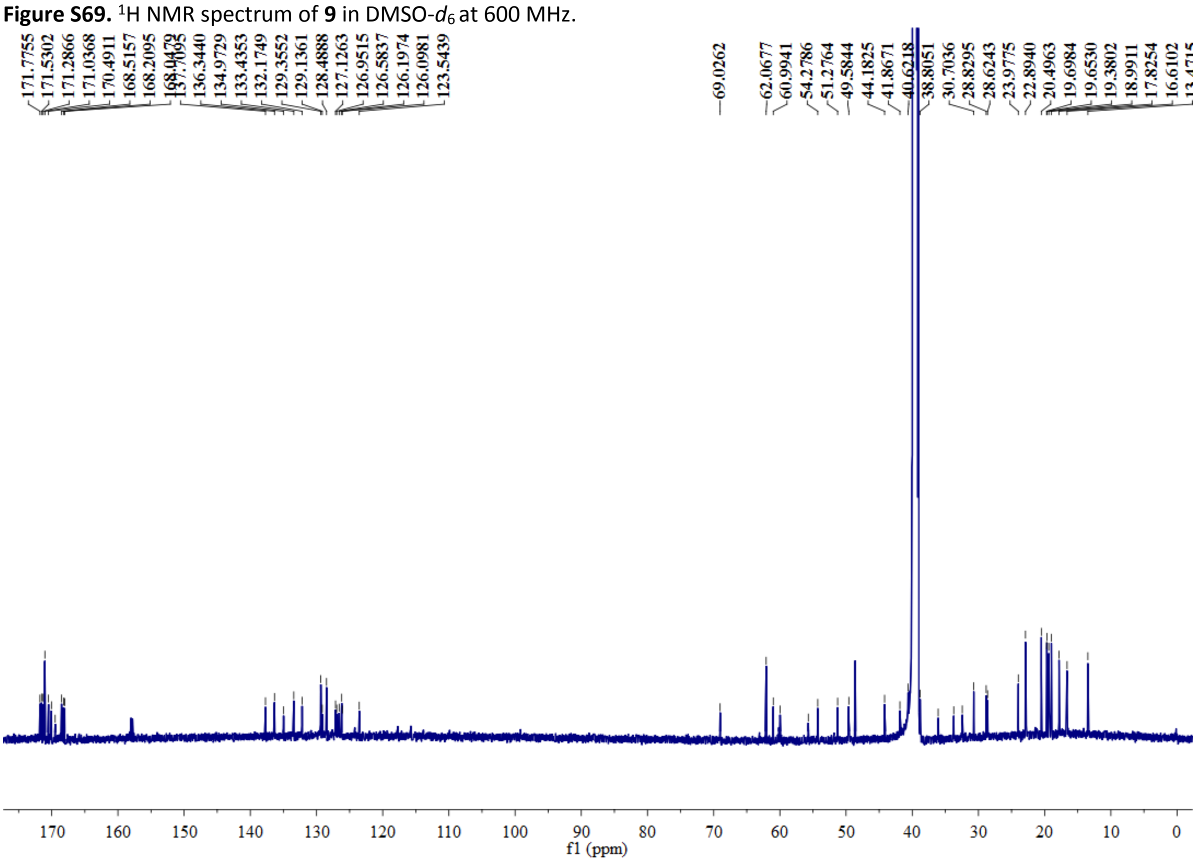

Figure S70. ^{13}C NMR spectrum of **9** in DMSO-d_6 at 150 MHz.

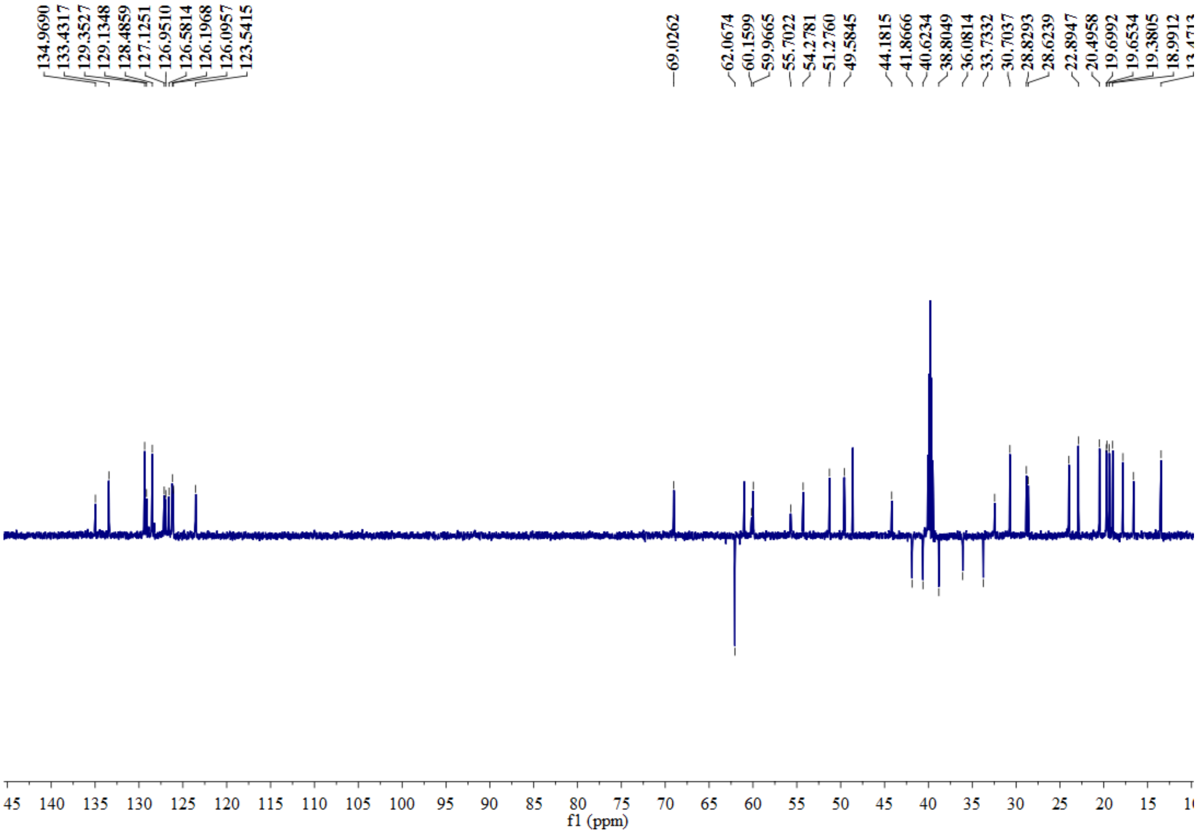

Figure S71. DEPT spectrum of **9** in DMSO-d_6.

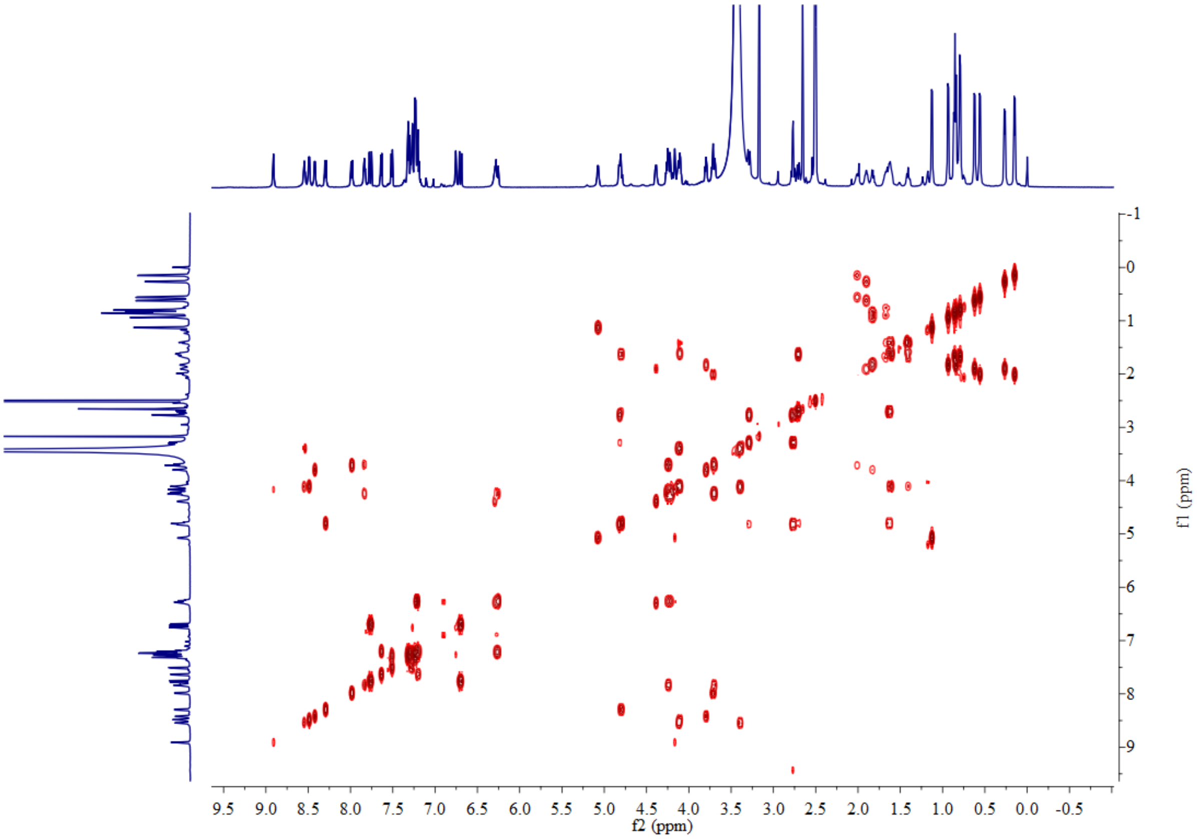

Figure S72. ^{1}H-^{1}H COSY spectrum of **9** in DMSO-d_6.

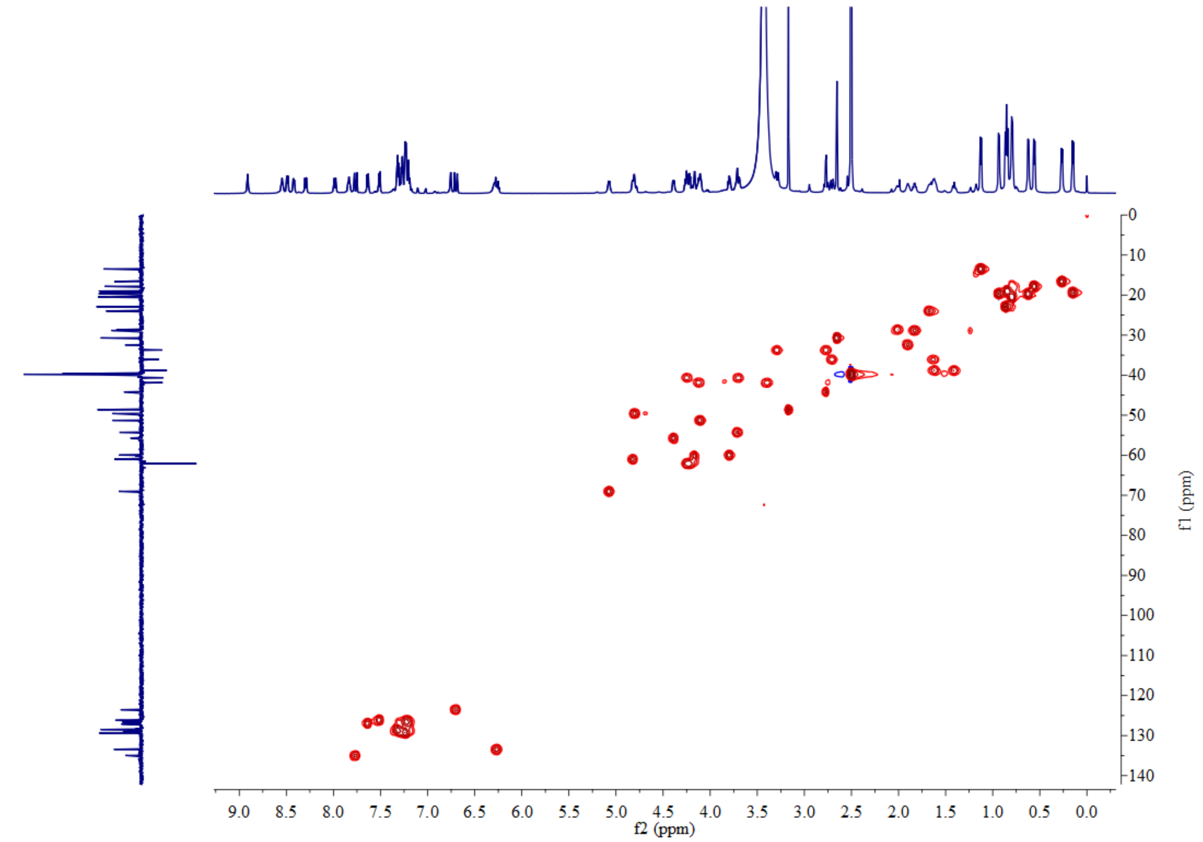

Figure S73. HSQC spectrum of **9** in DMSO-d_6.

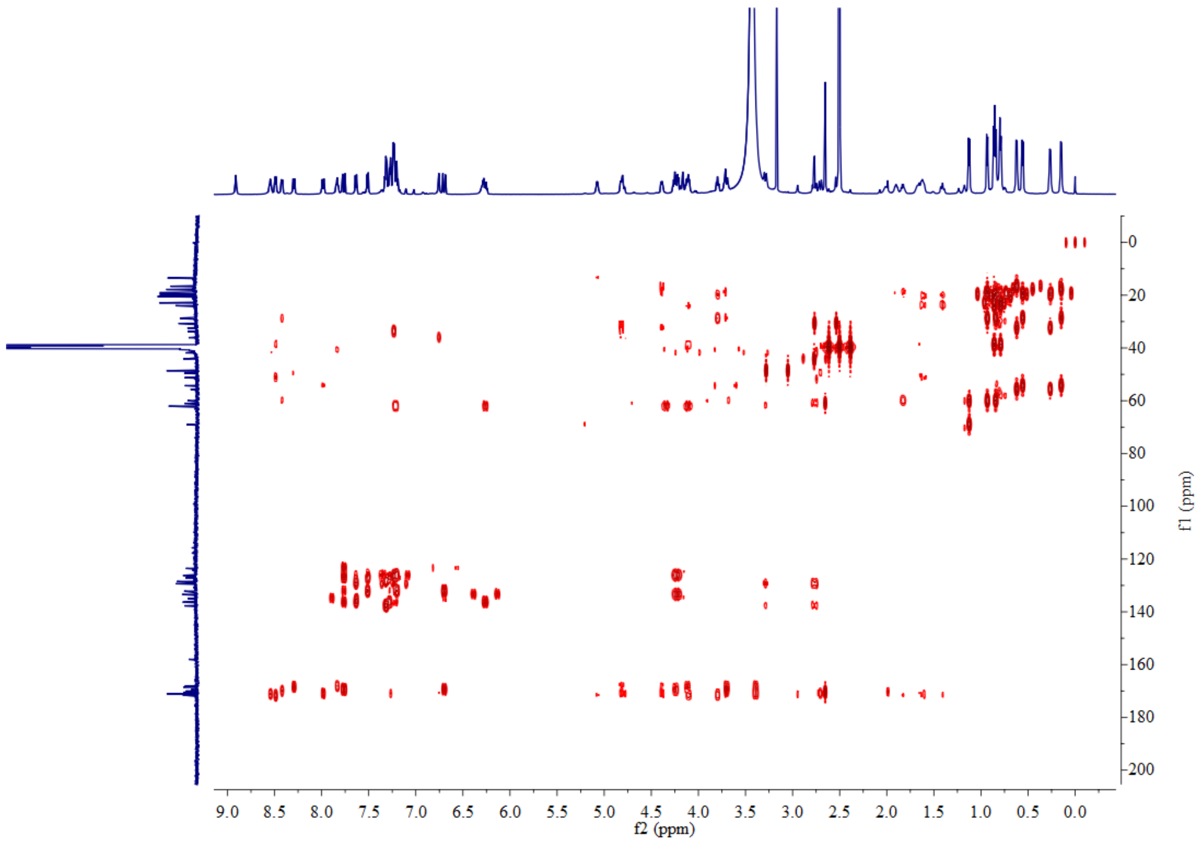

Figure S74. HMBC spectrum of **9** in DMSO-d_6.

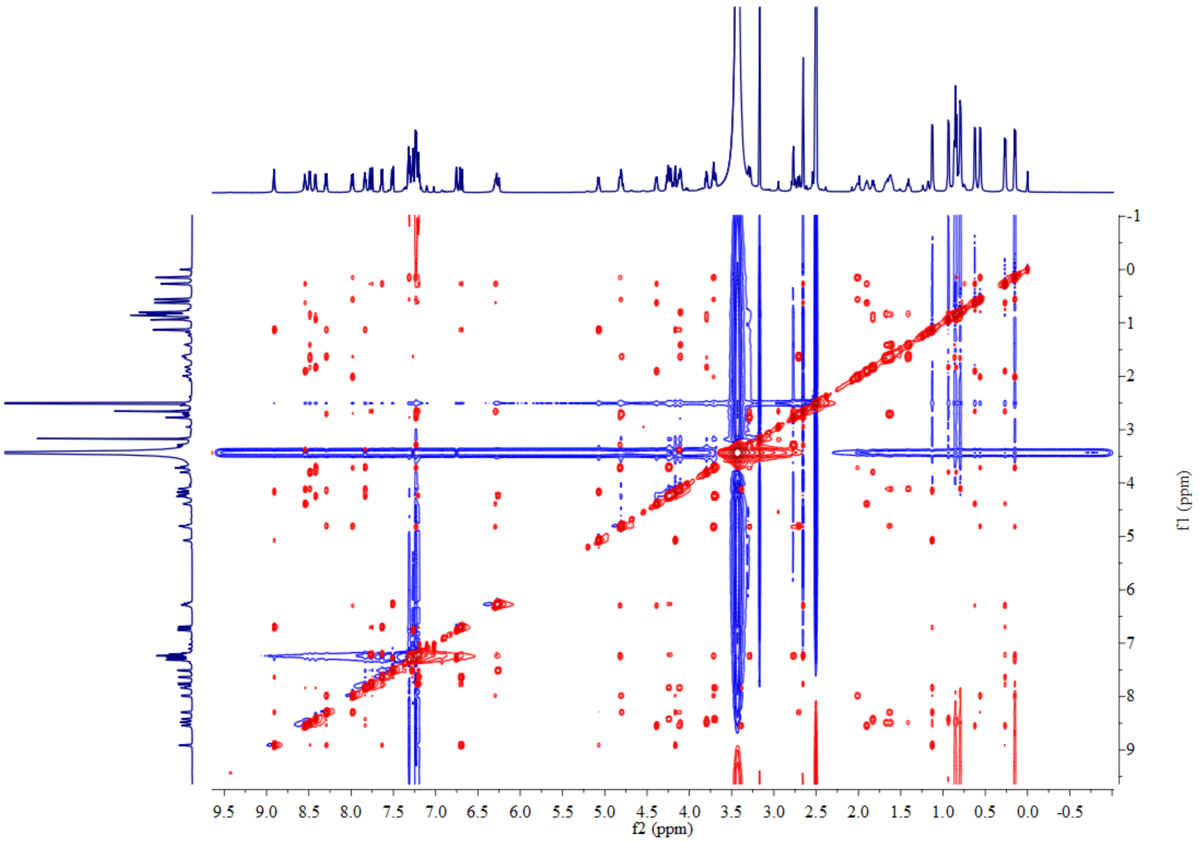

Figure S75. NOESY spectrum of **9** in DMSO-d_6.

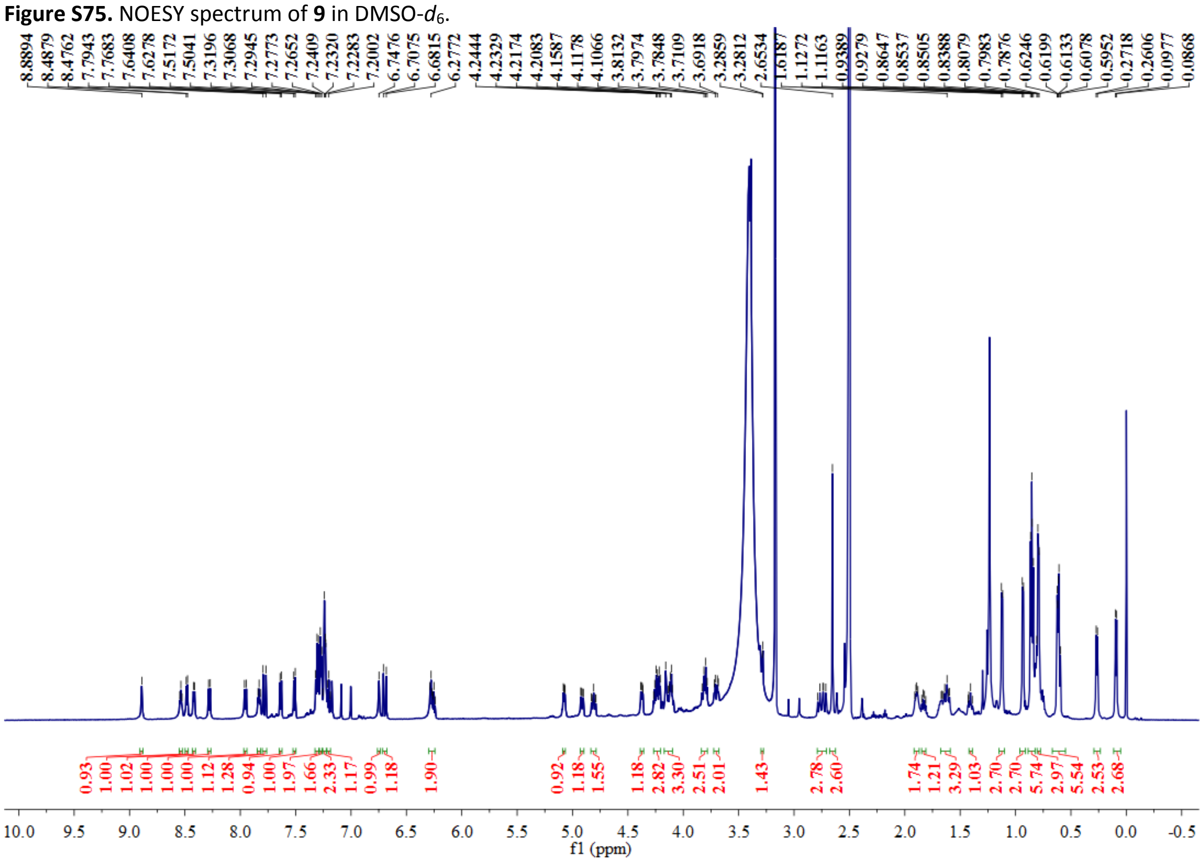

Figure S76. ^{1}H NMR spectrum of **10** in DMSO-d_6 at 600 MHz.

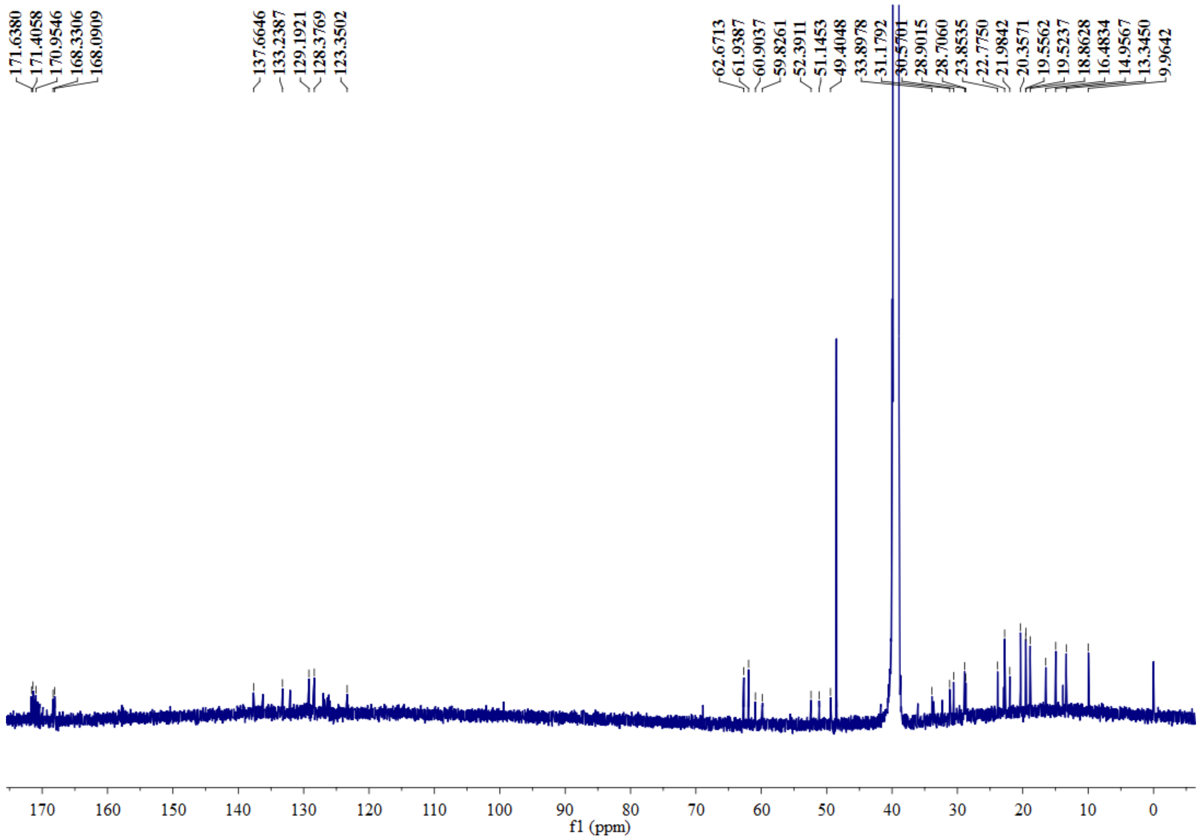

Figure S77. ^{13}C NMR spectrum of **10** in DMSO-d_6 at 150 MHz.

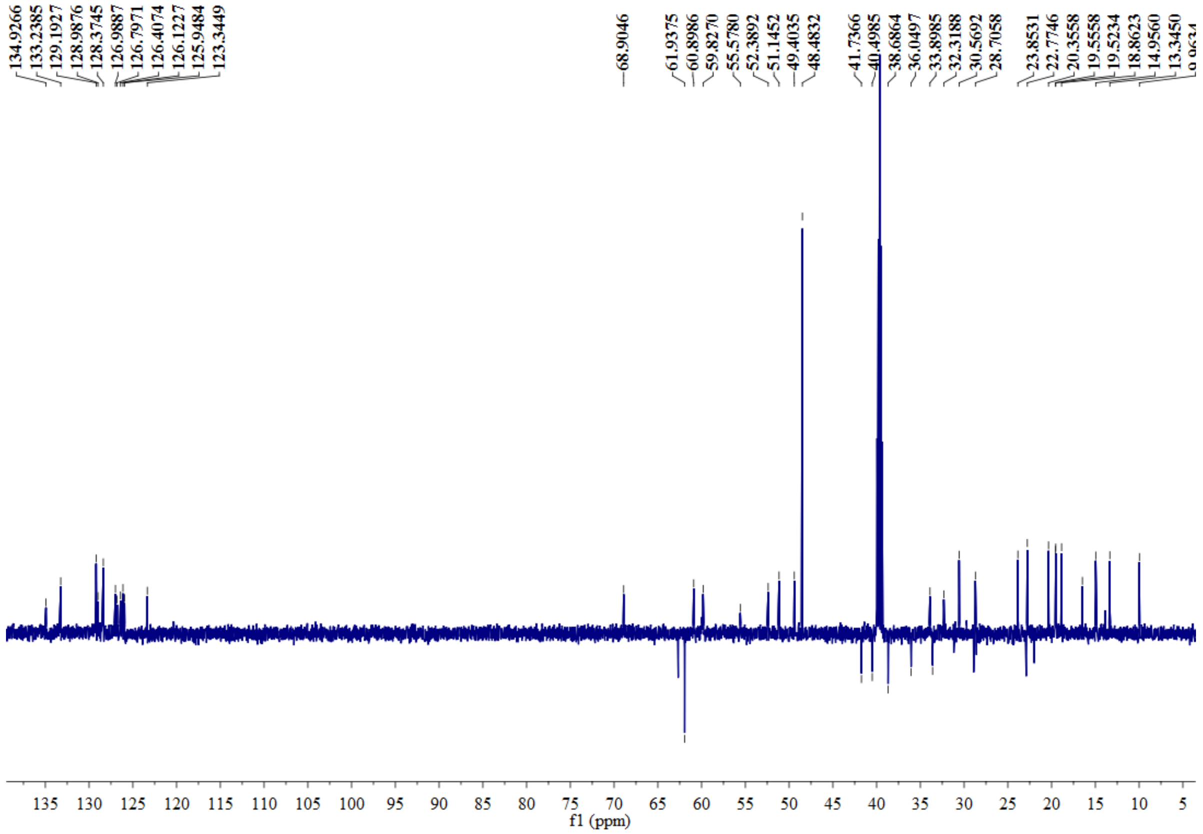

Figure S78. DEPT spectrum of **10** in DMSO-d_6.

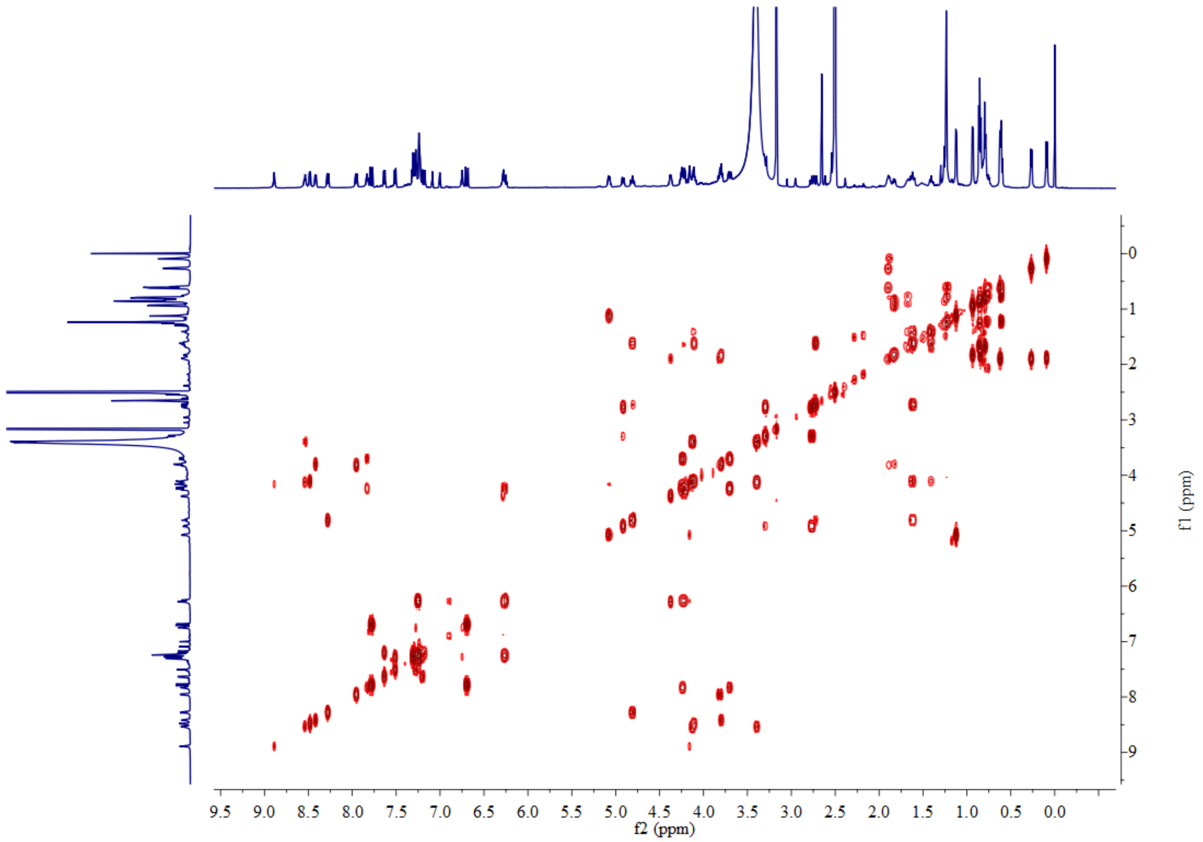

Figure S79. ^{1}H-^{1}H COSY spectrum of **10** in DMSO-d_6.

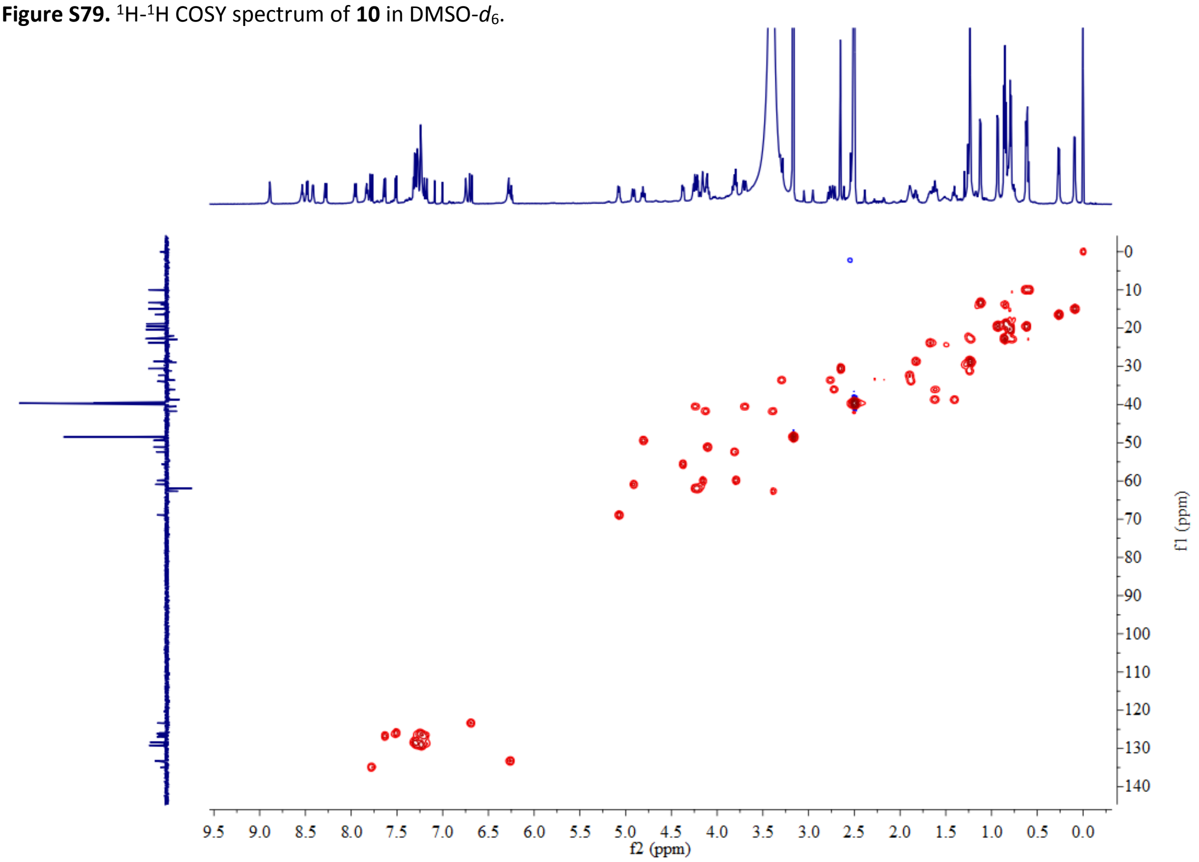

Figure S80. HSQC spectrum of **10** in DMSO-d_6.

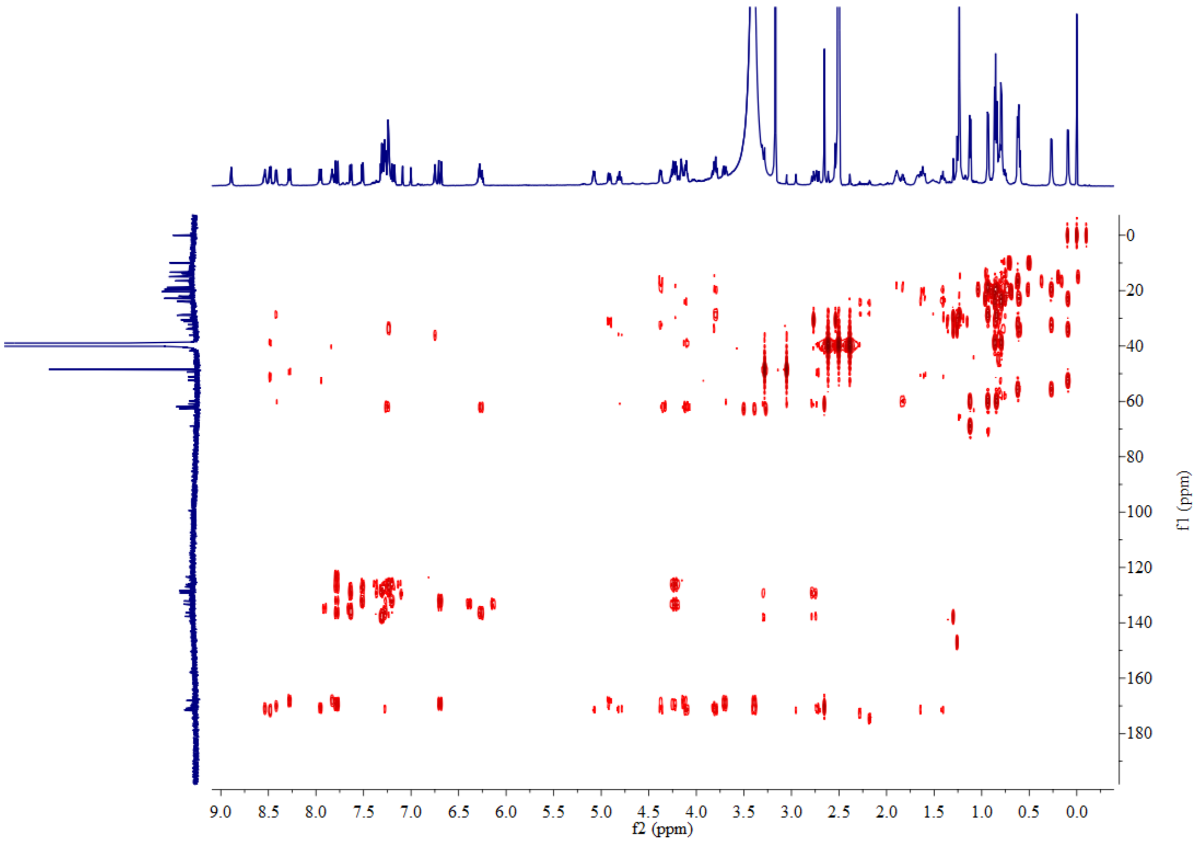

Figure S81. HMBC spectrum of **10** in DMSO-d_6.

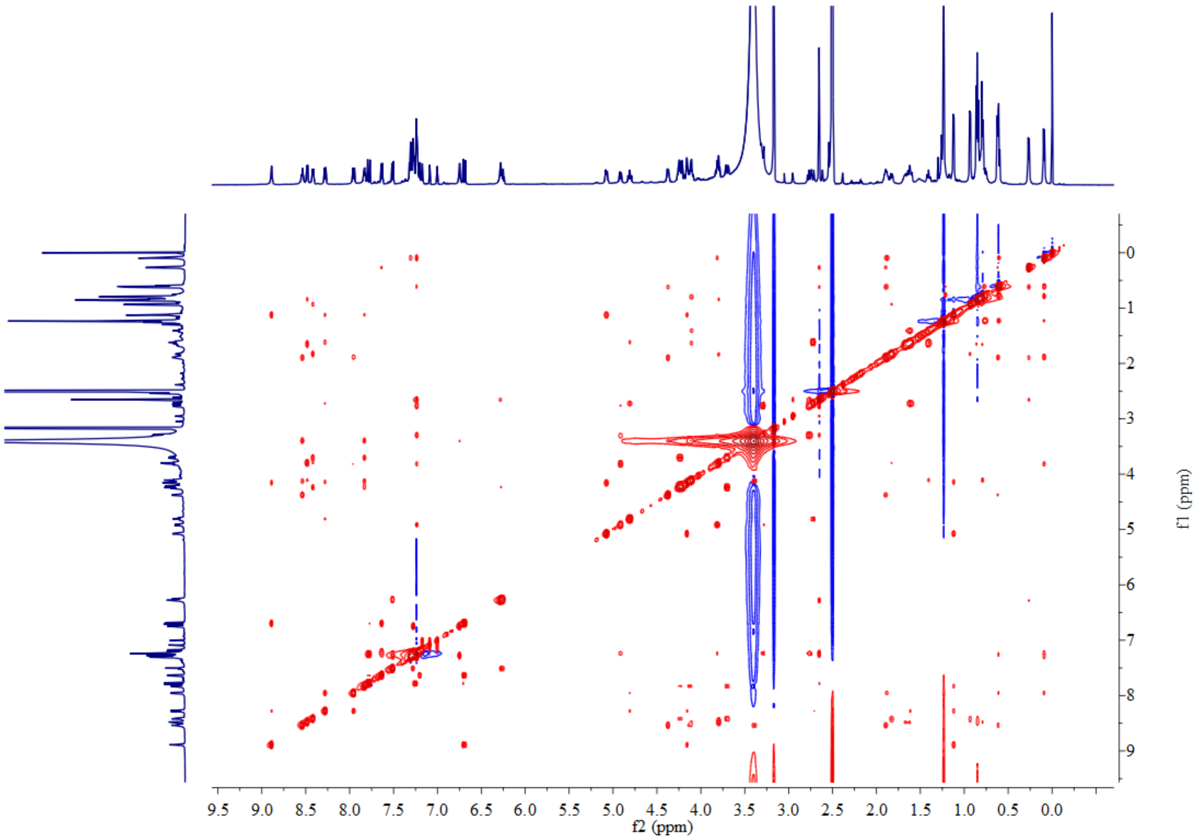

Figure S82. NOESY spectrum of **10** in DMSO-d_6.

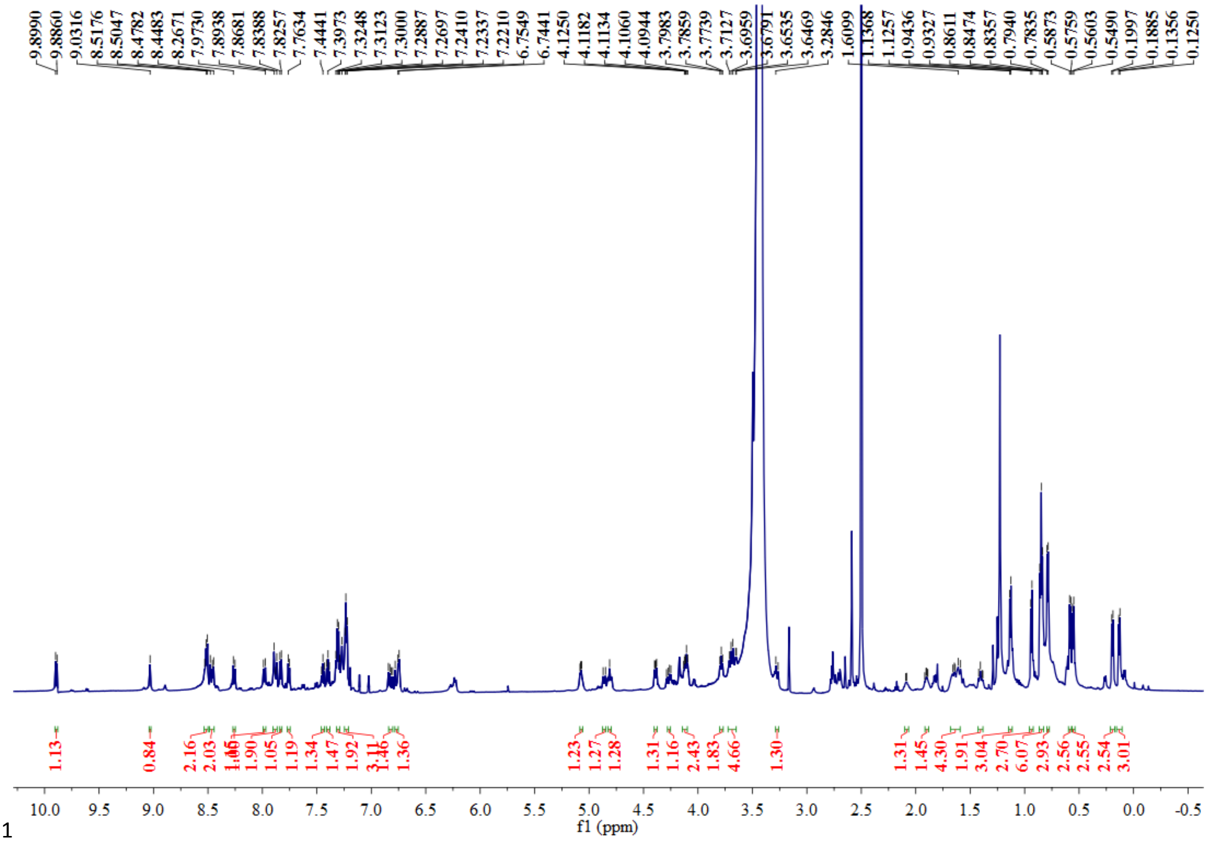

1

Figure S83. ^{1}H NMR spectrum of **11** in DMSO-d_6 at 600 MHz.

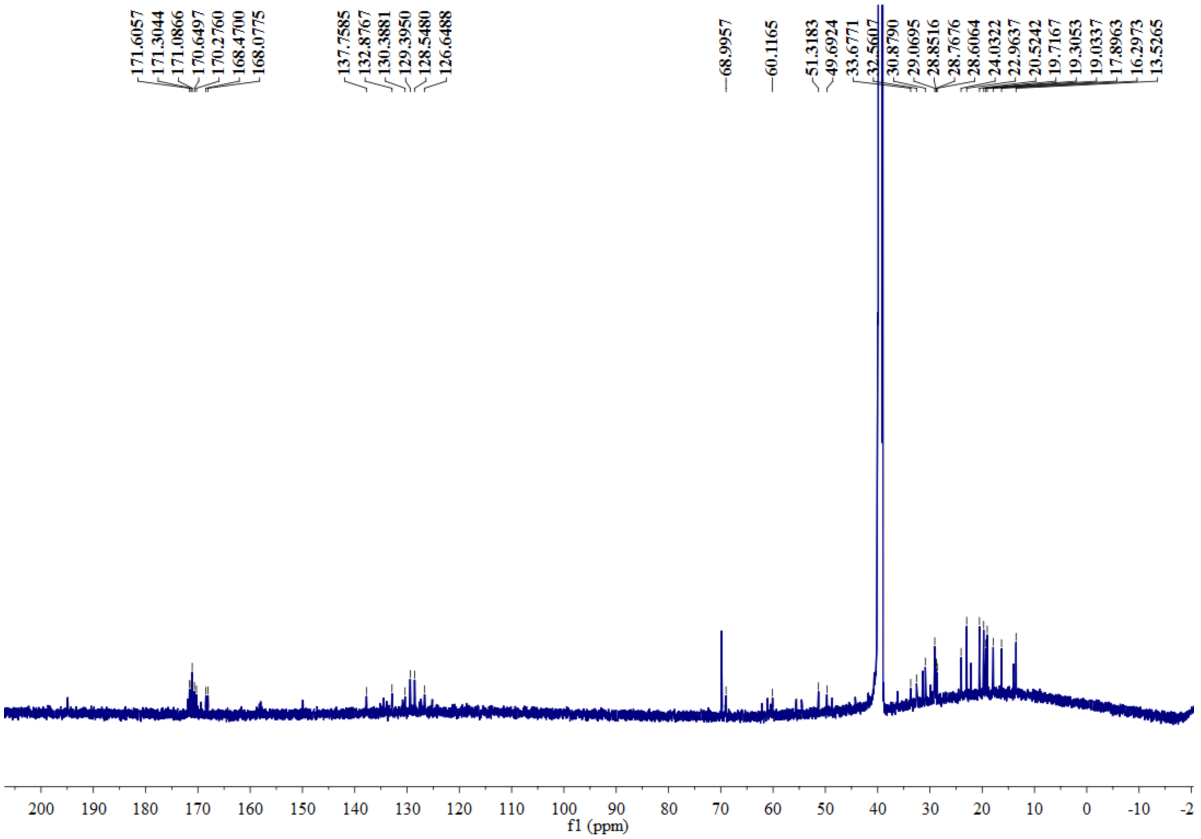

Figure S84. ^{13}C NMR spectrum of **11** in DMSO-d_6 at 150 MHz.

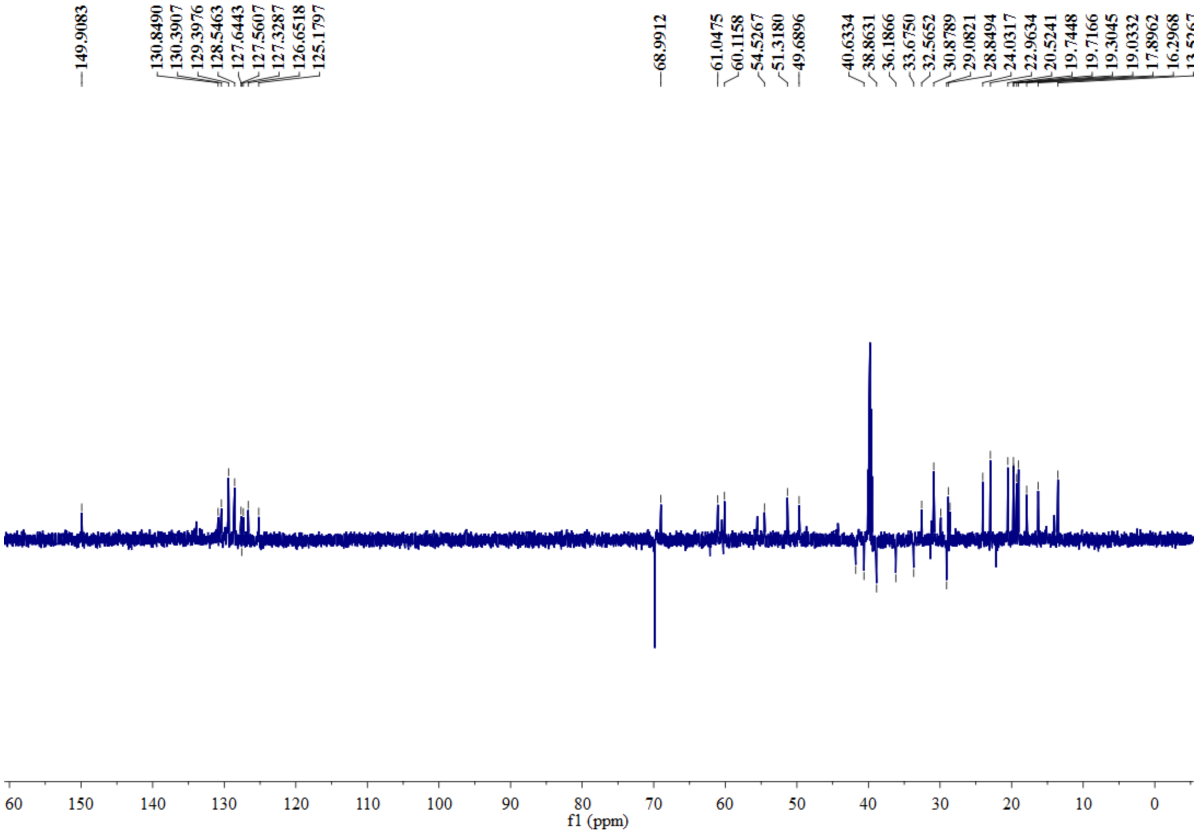

Figure S85. DEPT spectrum of **11** in DMSO-d_6.

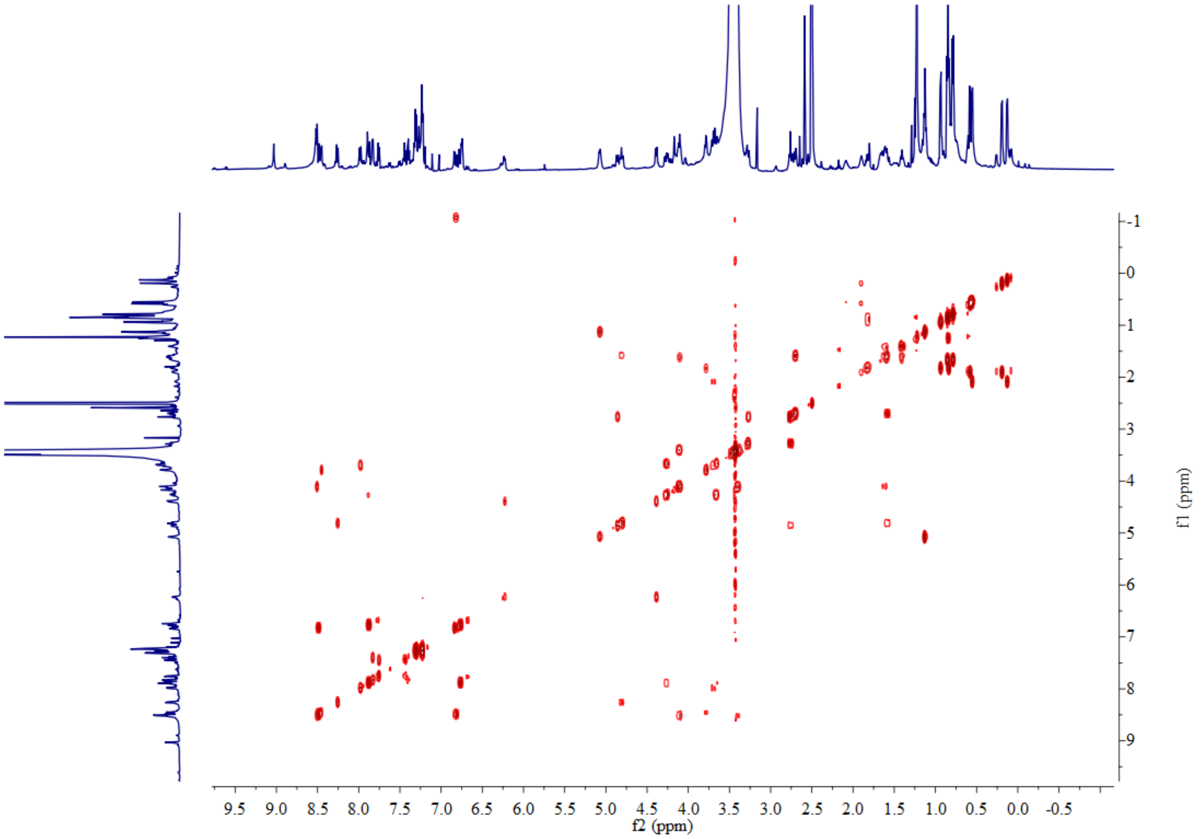

Figure S86. ^{1}H-^{1}H COSY spectrum of **11** in DMSO-d_6.

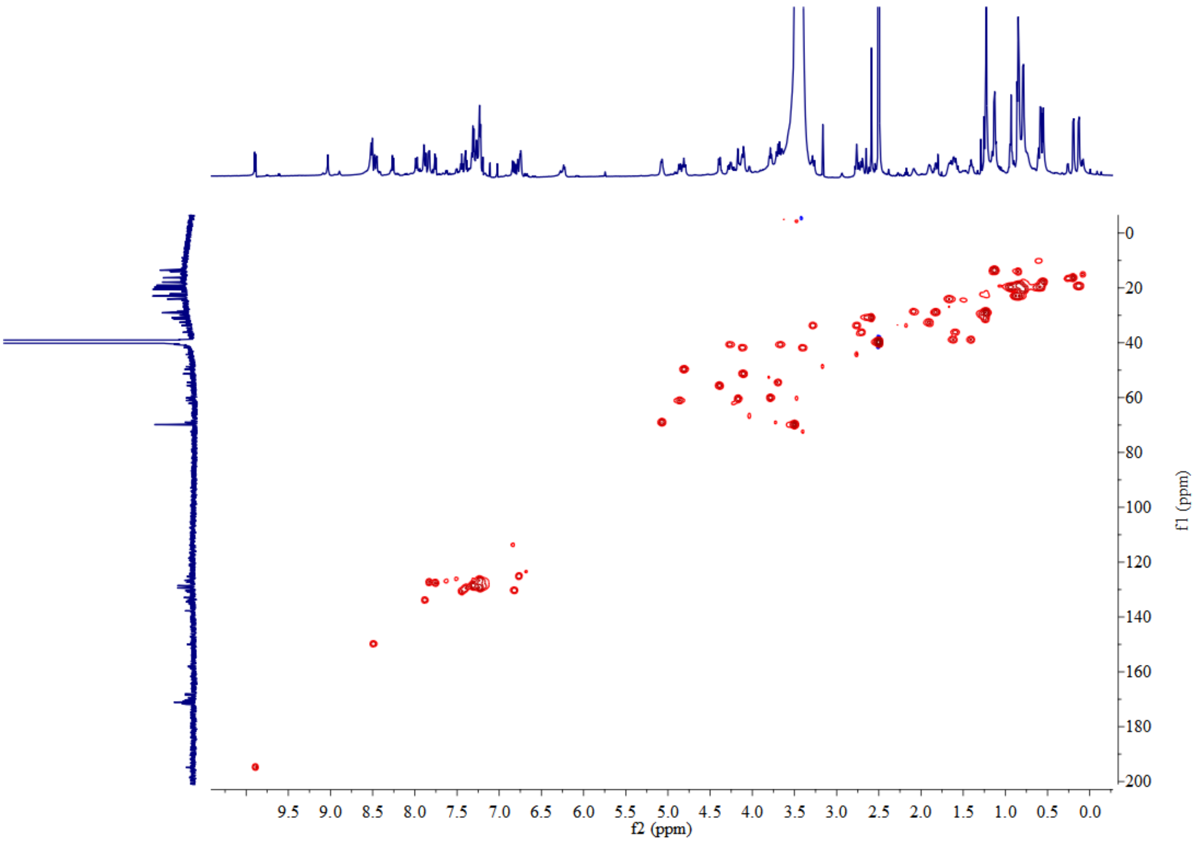

Figure S87. HSQC spectrum of **11** in DMSO-d_6.

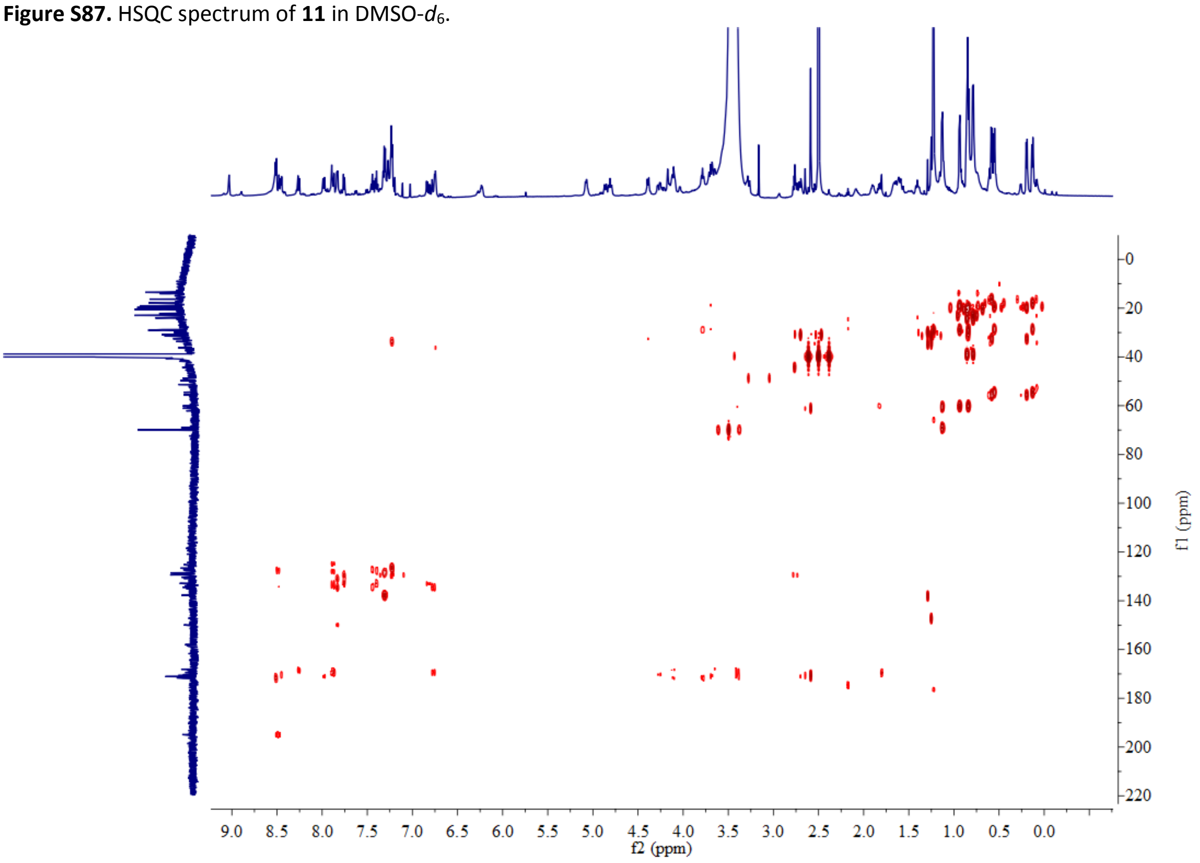

Figure S88. HMBC spectrum of **11** in DMSO-d_6.

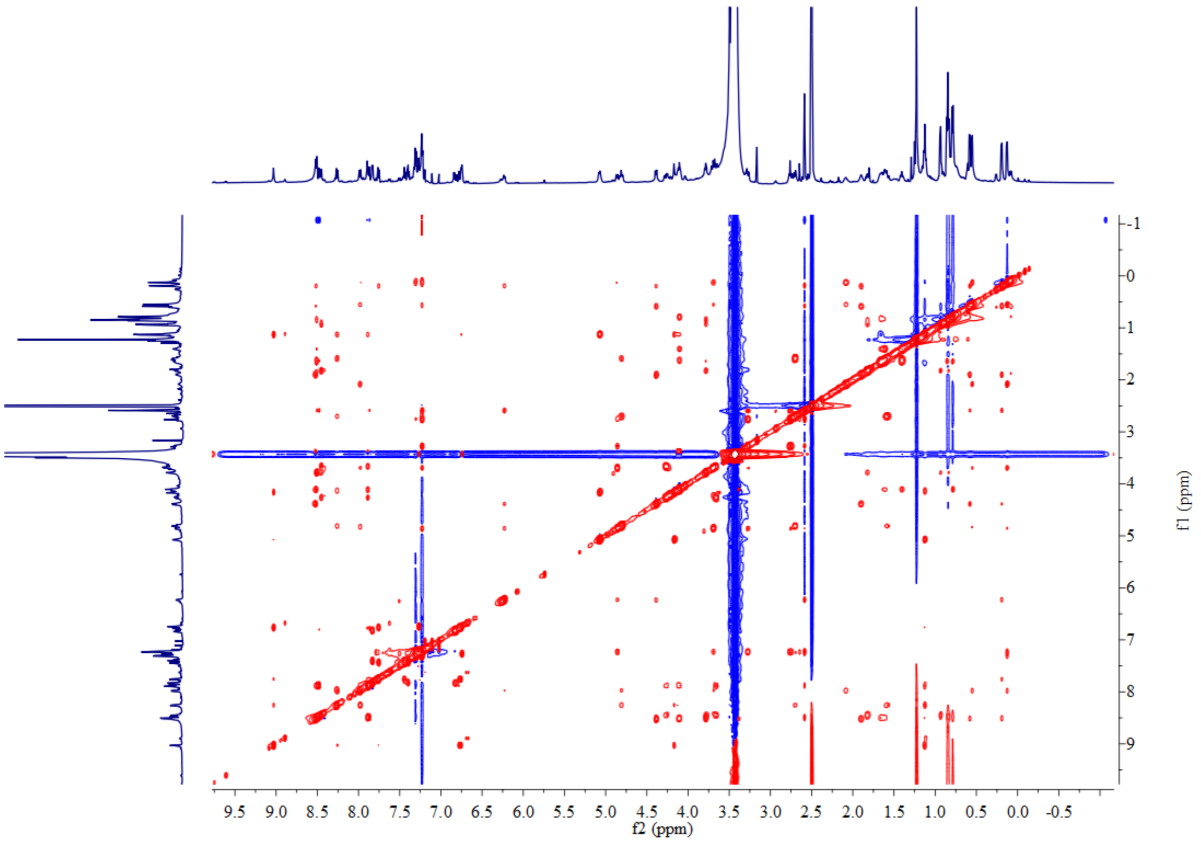

Figure S89. NOESY spectrum of **11** in DMSO-d_6.

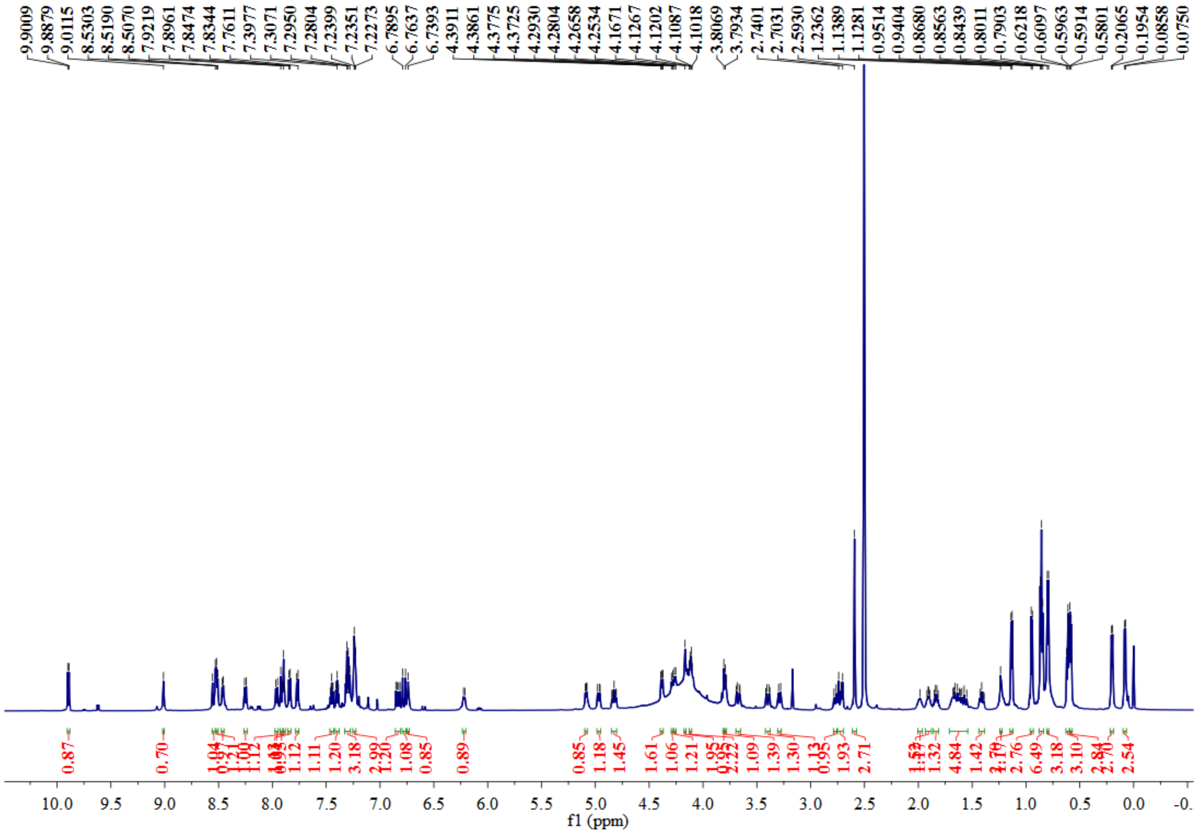

Figure S90. ^{1}H NMR spectrum of **12** in DMSO-d_6 at 600 MHz.

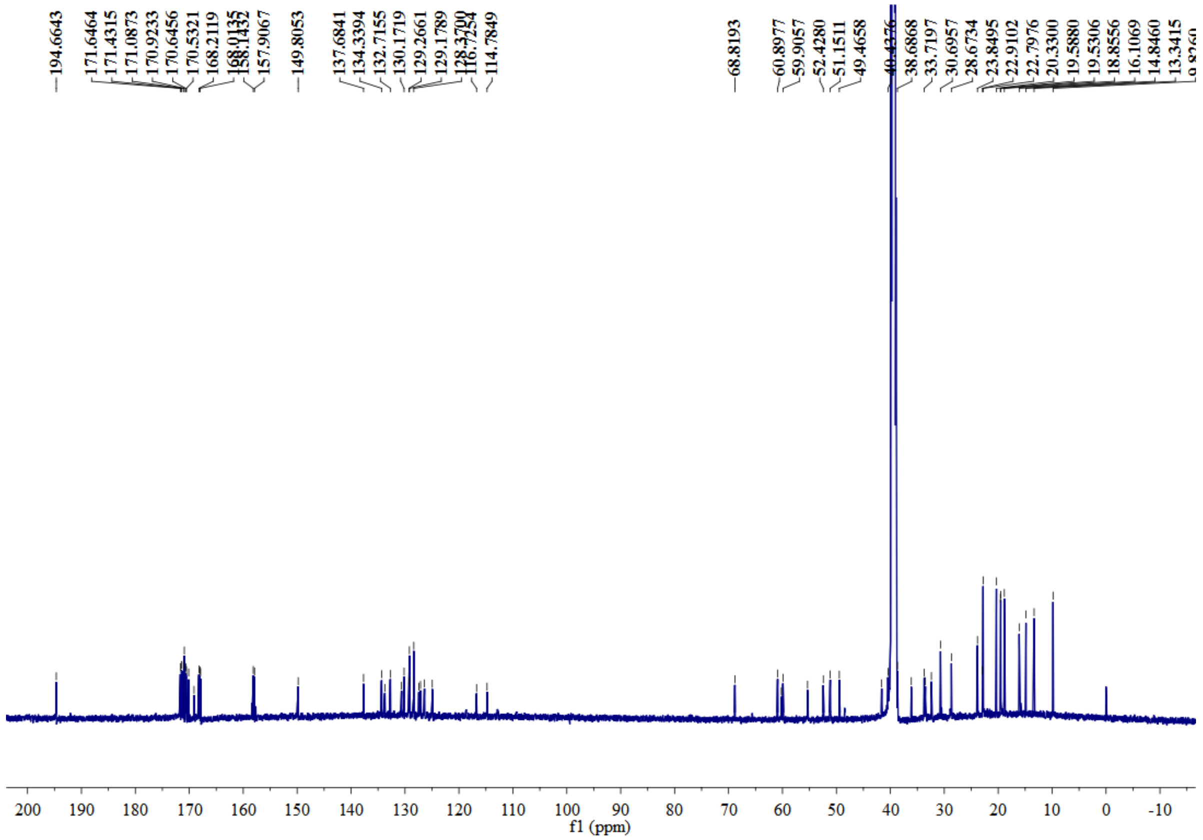

Figure S91. ^{13}C NMR spectrum of **12** in DMSO-d_6 at 150 MHz.

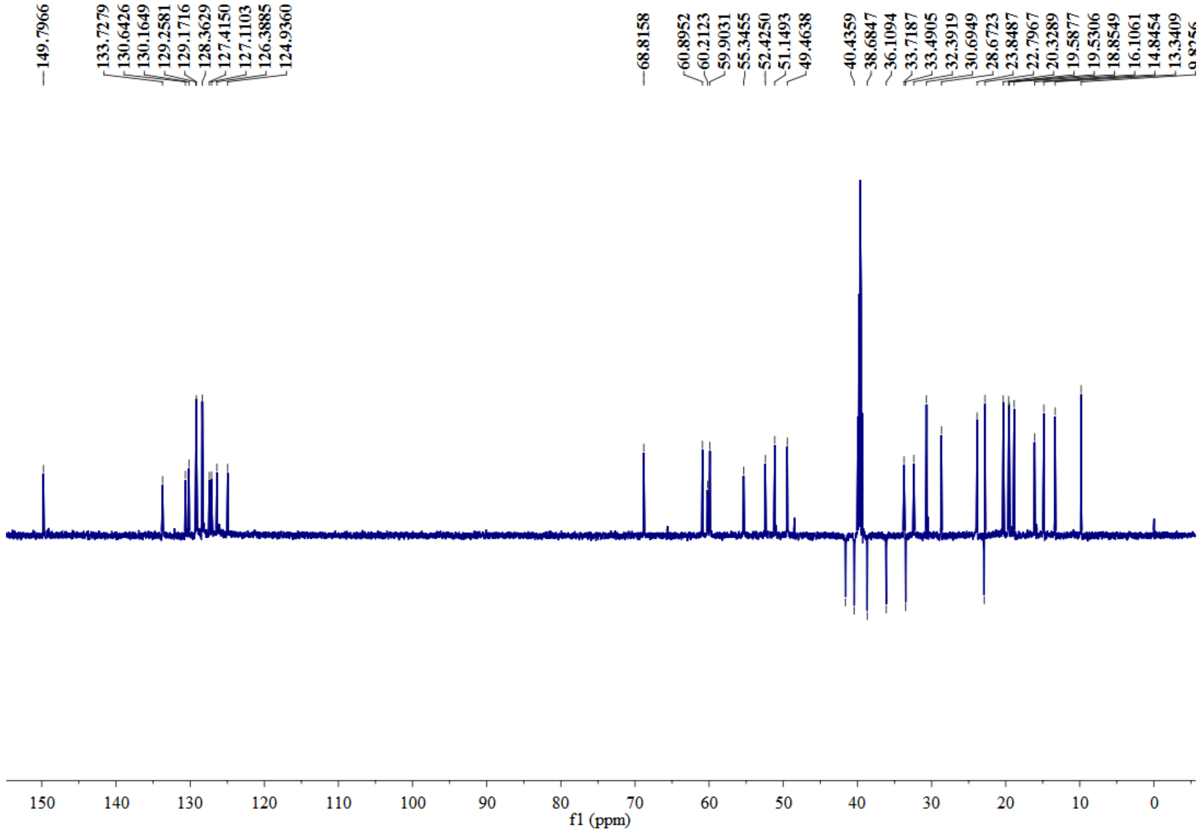

Figure S92. DEPT spectrum of **12** in DMSO-d_6.

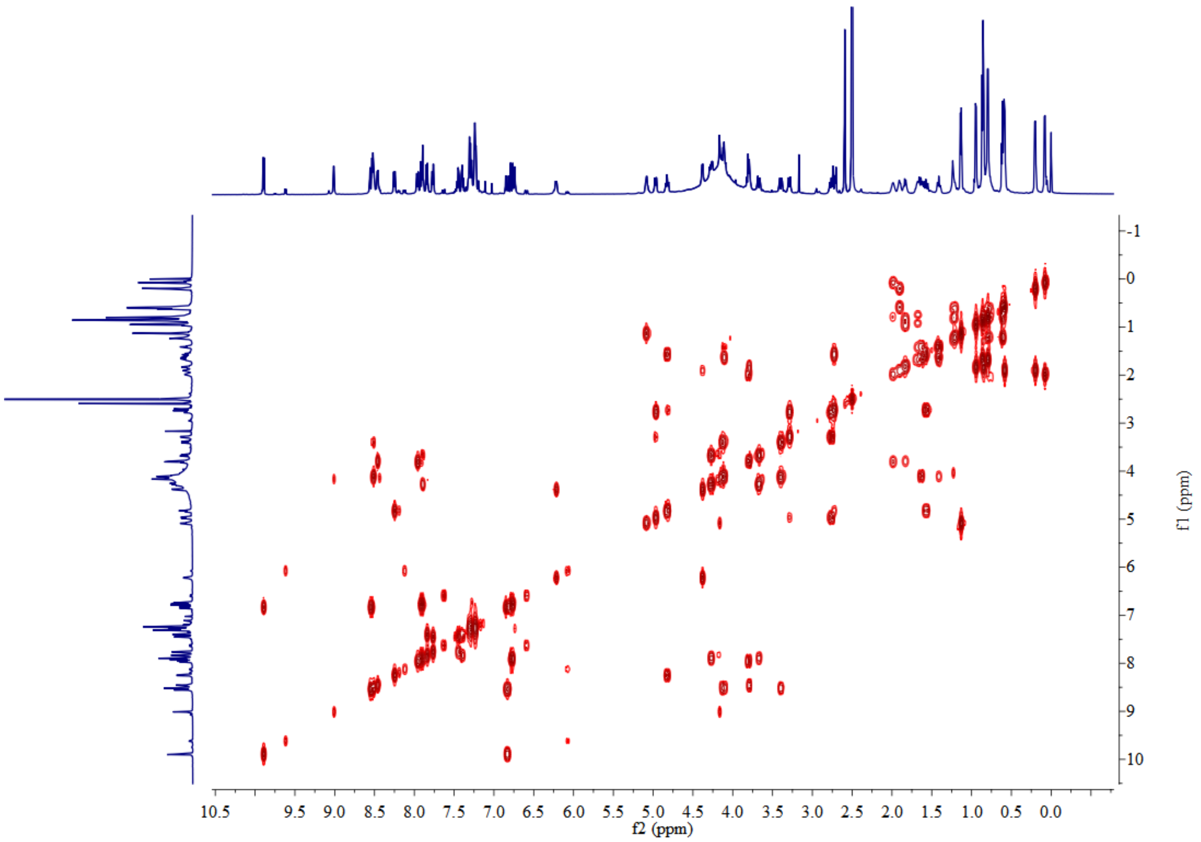

Figure S93. ^{1}H-^{1}H COSY spectrum of **12** in DMSO-d_6.

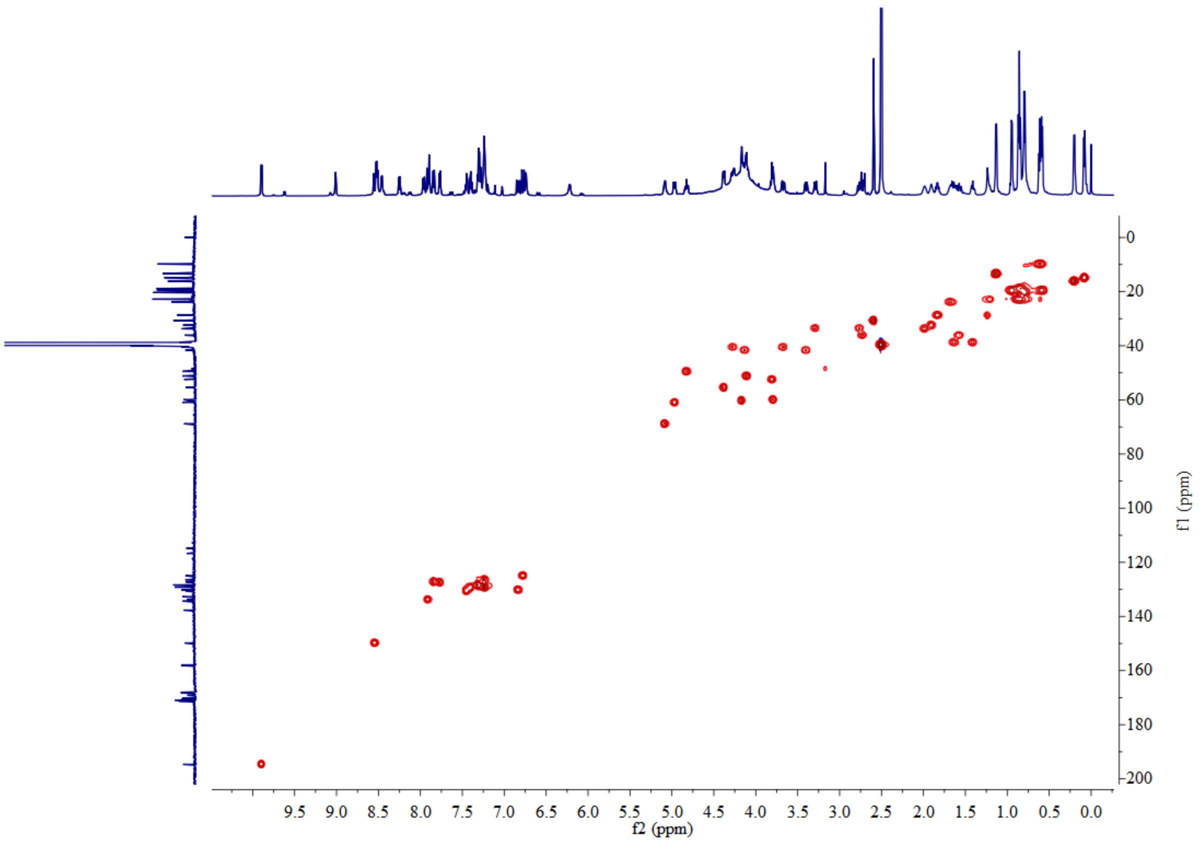

Figure S94. HSQC spectrum of **12** in DMSO-d_6.

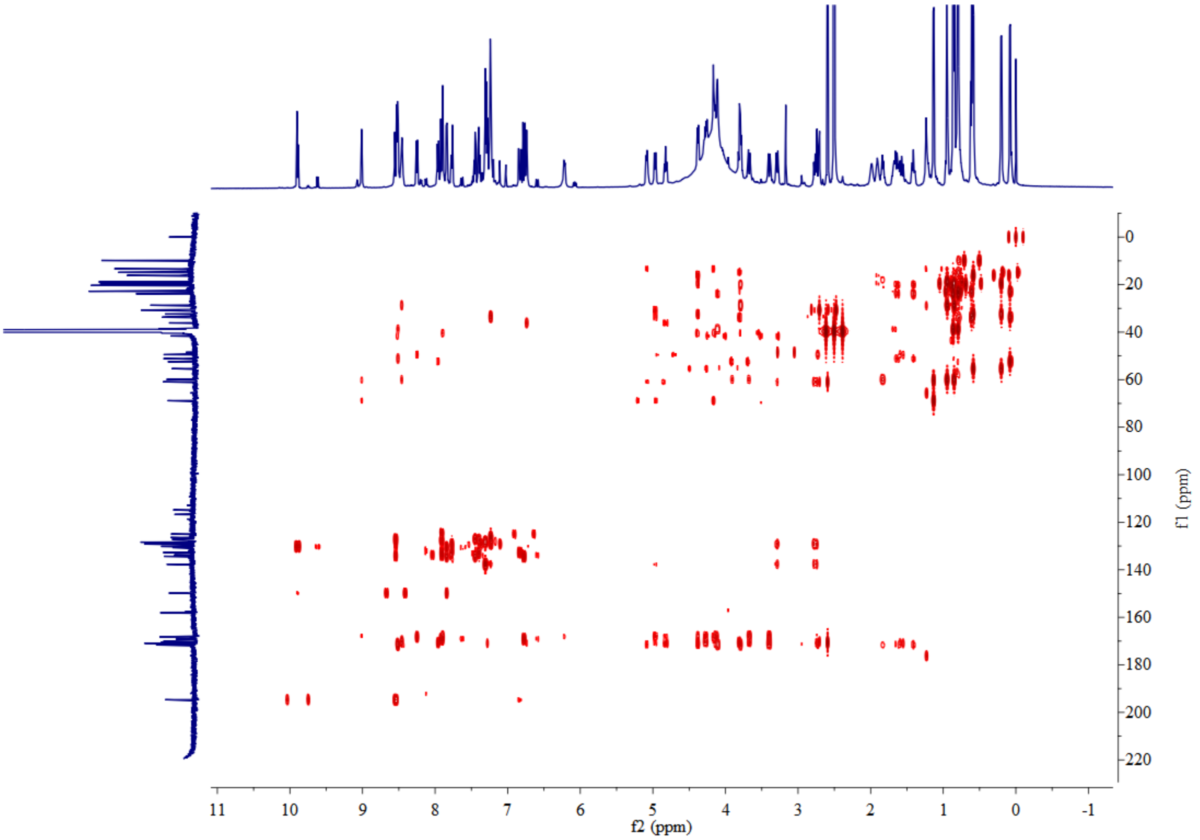

Figure S95. HMBC spectrum of **12** in DMSO-d_6.

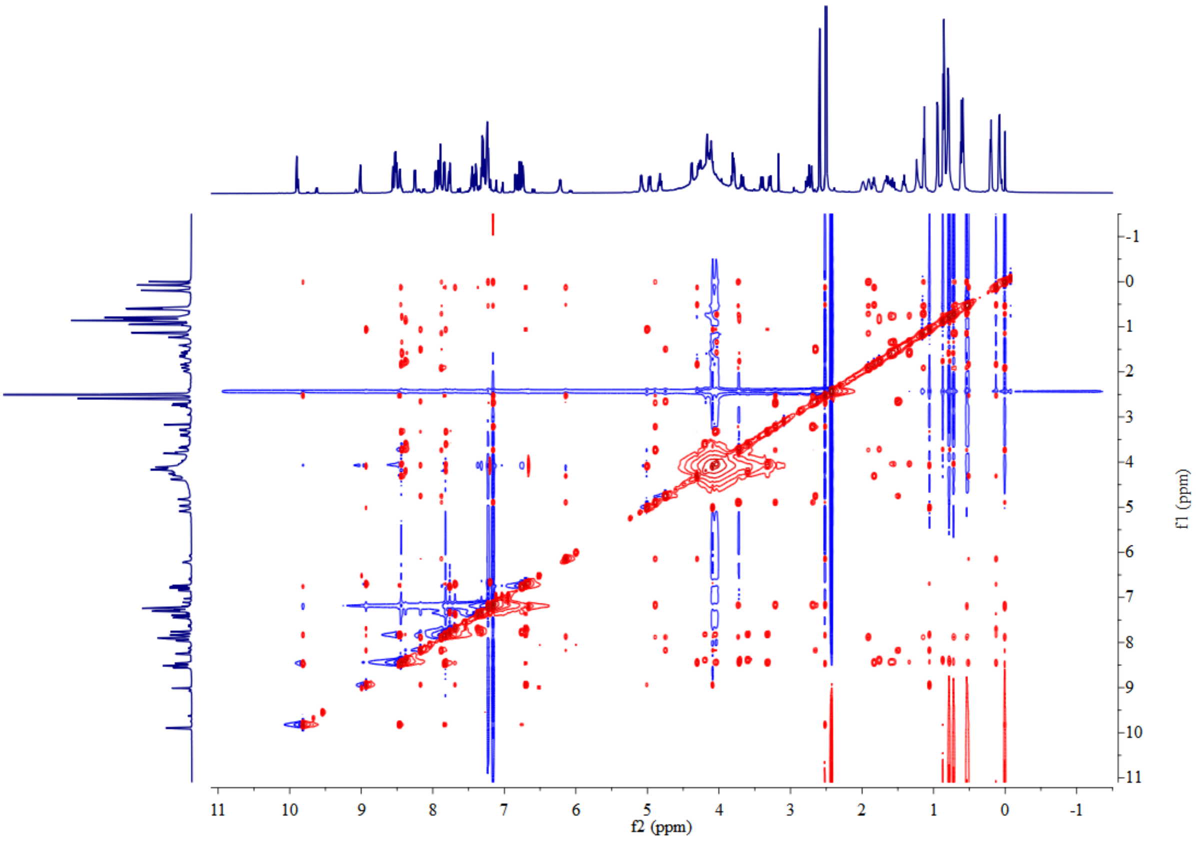

Figure S96. NOESY spectrum of **12** in DMSO-d_6.

Rerences

[1] S. Pohle, C. Appelt, M. Roux, H. P. Fiedler, H. Dou, R. D. Süssmuth, *J. Am. Chem. Soc*. **2011**, *133*, 6194–6205.

[2] S. Um, S. H. Park, J. Kim, H. J. Park, K. Ko, H. S. Bang, S. K. Lee, J. Shin, D. C. Oh, *Org. Lett*. **2015**, *17*, 1272–1275.

[3] D. A. Hopwood, M. J. Bibb, K. F. Chater, T. Kieser, C. J. Bruton, H. M. Kieser, D. J. Lydiate, C. P. Smith, J. M. Ward, H. Schrempf, *Genetic Manipulation of Streptomyces-A Laboratory Manual*, Cold Spring Harbor Laboratory Press, New York **1985**.

[4] J. A. Gerlt, Biochemistry **2017**, *56*, 4293-4308.

[5] T. G. G. Battye, L. Kontogiannis, O. Johnson, H. R. Powell, A. G. W. Leslie, *Acta. Crystallogr. D.* **2011**, *67*, 271–281.

[6] A. J. McCoy, R. W. Grosse-Kunstleve, P. D. Adams, M. D. Winn, L. C. Storoni, R. J. Read, *J. Appl. Crystallogr.* **2007**, *40*, 658–674.

[7] P. D. Adams, M. Mustyakimov, P. V. Afonine, P. Langan, *Acta Crystallogr. D*. **2009**, *65*, 567–573.

[8] P. Emsley, M. Crispin, *Acta Crystallogr. D*. **2018**, *74*, 256–263.

[9] O. Trott, A. J. Olson, *J. Comput. Chem*. *2010*, *31*, 455–461.

[10] W. Guo, W. Liu, Z. Chen, Y. Gu, S. Peng, l. Shen, Y. Shen, S. Wang, G. S. Feng, Y. Sun, Q. Xu, *Nat. Commun.* **2017**, *8*, 2168–2182.

[11] M. Bierman, R. Logan, K. O'Brien, E. T. Seno, R. N. Rao, B. E. Schoner, *Gene* **1992**, *116*, 43–49

[12] M. R. Green, J. Sambrook, Molecular Cloning: A Laboratory Manual 4th ed. Cold Spring Harbor Laboratory Press, **2012.**

[13] M. Röttig, M. H. Medema, K. Blin, T. Weber, C. Rausch, O. Kohlbacher, *Nucleic Acids Res.* **2011**, *39*, 362–367.

[14] R. C. Edgar, *Nucleic Acids Res.* **2004**, *32*, 1792–1797.

[15] X. Robert, P. Gouet, *Nucleic Acids Res.* **2014**, *42*, W320–W324.
